# Integrative bulk and single-cell transcriptomic analysis reveals COL1A2-driven ECM remodeling and focal adhesion signaling associated with the transition from non-muscle-invasive to muscle-invasive bladder cancer

**DOI:** 10.3389/fonc.2025.1716324

**Published:** 2026-01-05

**Authors:** Menglu Li, Xinwei Liu, Yuhui Xue, Yichen Lu, Zhiqiang Chen, Yuwei Zhang, Weiguo Chen, Shan-Chao Zhao, Ke Wang, Ninghan Feng

**Affiliations:** 1 Department of Urology, Jiangnan University Medical Center, Wuxi, China; 2 Institute of Urology, Wuxi School of Medicine, Jiangnan University, Wuxi, China; 3 Department of Urology, Wuxi No. 2 People’s Hospital, Nanjing Medical University, Nanjing, China; 4 Department of Urology, Wuxi Medical Center, Nantong University, Nantong, China; 5 Department of Urology, The First Affiliated Hospital of Soochow University, Suzhou, China; 6 Department of Urology, The Fifth Affiliated Hospital, Southern Medical University, Guangzhou, China; 7 Department of Urology, The Affiliated Hospital of Qingdao University, Qingdao, China

**Keywords:** COL1A2, extracellular matrix, focal adhesion kinase, muscle-invasive bladder cancer, non-muscle-invasive bladder cancer

## Abstract

**Background:**

Bladder cancer (BCA) shows significant prognostic differences between non-muscle-invasive (NMIBC) and muscle-invasive (MIBC) forms. While NMIBC frequently recurs and can progress to invasive disease, reliable biomarkers to monitor this transition are lacking. Extracellular matrix (ECM) remodeling is a critical factor influencing tumor aggressiveness, yet the key regulators of ECM changes across BCA stages remain unclear. In this study, we investigate the role of COL1A2 in ECM-related tumor biology and its potential as a prognostic biomarker for BCA progression.

**Methods:**

We utilized a multi-step bioinformatics pipeline, analyzing RNA-seq data from TCGA and GEO datasets to identify molecular differences between NMIBC and MIBC. Prognostic markers were prioritized via differential expression analysis, Cox regression, and Kaplan–Meier survival analysis. The regulatory network was explored using protein-protein interaction analysis, and ECM-related activity was quantified through ssGSEA. Cell-type-specific insights were gained through single-cell RNA-seq analysis, and intercellular communication was deciphered using CellChat. Functional validation was performed through *in vitro* knockdown experiments in BCA cell lines.

**Results:**

COL1A2 emerged as a key prognostic ECM-related gene associated with MIBC. Single-cell RNA-seq analysis revealed that COL1A2 and ECM components were predominantly enriched in matrix cancer-associated fibroblasts (CAFs), with PTK2 (FAK, focal adhesion kinase) upregulated in epithelial cells undergoing epithelial-mesenchymal transition (EMT). CellChat analysis uncovered a dominant COL1A2-mediated signaling axis from matrix CAFs to EMT epithelial cells via COL1A1/2–SDC1/4 ligand-receptor interactions. Functional assays confirmed that COL1A2 knockdown significantly impaired MIBC cell invasion and migration by suppressing ECM remodeling and EMT.

**Conclusion:**

Our results suggest that the COL1A2–ECM–FAK signaling axis plays a critical role in MIBC progression, and COL1A2 could serve as a potential biomarker and therapeutic target for muscle-invasive bladder cancer.

## 1 Introduction

Bladder cancer (BCA), the second most common urological malignancy, accounts for over 610,000 new cases and 220,000 deaths annually worldwide (1, 2). Clinically classified into non-muscle-invasive bladder cancer (NMIBC) and muscle-invasive bladder cancer (MIBC) subtypes, these entities exhibit stark therapeutic contrasts: NMIBC patients receiving transurethral resection show > 90% 5-year survival, whereas MIBC patients undergoing radical cystectomy face ≤ 50% survival with frequent metastasis (3–5). Although molecular studies reveal higher mutational burden in MIBC (TP53, FGFR3, ERCC3) (6, 7), clinically actionable biomarkers remain scarce. Accordingly, there is a pressing need to systematically elucidate stage-specific drivers to enable the development of targeted therapies that halt invasive progression.

The extracellular matrix (ECM) constitutes a dynamically orchestrated molecular scaffold comprising interconnected protein networks and proteoglycans (8). Proteolytic enzymes, particularly matrix metalloproteinases (MMPs), execute spatiotemporal remodeling of ECM architecture through coordinated degradation and turnover, thereby mediating cell-cell and cell-matrix adhesion dynamics, liberating sequestered growth factors and cytokines, and generating bioactive matrix fragments with pro- or anti-angiogenic functionalities (9–11). Crucially, the ECM serves as a signaling nexus integrating diverse cellular cues—spanning differentiation, proliferation, and migratory programming—to govern tumor evolution, including metastatic dissemination. Dysregulation of ECM composition and biomechanical properties (e.g., aberrant stiffness) disrupts stem cell polarity and asymmetric division while driving epithelial-mesenchymal transition (EMT) in cancer stem cells (12, 13). Pathological ECM remodeling—characterized by collagen hyperdeposition and lysyl oxidase (LOX)-mediated crosslinking—induces tissue stiffening that propagates biomechanical transduction through YAP/TAZ mechanosensitive pathways, ultimately potentiating invasive and metastatic phenotypes (14–21).

Type I collagen (COL1), a pivotal component of the extracellular matrix (ECM), is composed of two α1 chains and one α2 chain encoded by COL1A2 (22). As the most abundant collagen in humans, the COL1A2-encoded α2 chain interacts with diverse matrix proteins to mediate cellular anchorage within the ECM (23, 24). Emerging studies have unveiled the context-dependent roles of COL1A2 in cancer progression and metastasis. For instance, while COL1A2 suppresses fibrosarcoma cell migration, it paradoxically promotes chondrosarcoma cell motility, suggesting its functional divergence through interactions with distinct molecular partners across tumor microenvironments (25). In colorectal cancer (CRC) metastasis, Ma et al. employed integrated proteogenomic profiling of human CRC liver metastases and demonstrated that elevated COL1A2 expression in hepatic metastatic tissues correlates with shorter overall survival and disease-free survival. Functional validation revealed that COL1A2 overexpression significantly enhances CRC cell invasion and migration *in vitro* (26). These findings highlight the critical need to investigate how COL1A2 dysregulation drives invasive and metastatic behaviors in BCA, with particular focus on its stage-specific role in promoting tumor aggressiveness. Given the pivotal role of COL1A2 in shaping the composition and organization of the ECM, elucidating how these ECM alterations are translated into intracellular signaling pathways that drive tumour cell behaviour is also critical.

Building on COL1A2-mediated ECM remodeling, focal adhesion kinase (FAK) - a non-receptor tyrosine kinase - acts as a central signaling mediator that translates extracellular matrix cues into intracellular responses, thereby regulating critical cellular functions including adhesion, migration, and survival (27–29). Consequently, dysregulated ECM remodeling orchestrated by COL1A2 may modulate FAK activation, establishing a mechanistic axis that links extracellular matrix dynamics to intracellular signaling cascades fundamental to tumour progression. Accumulating evidence positions FAK as an upstream regulatory hub in ECM-mediated signaling cascades, coordinating ECM remodeling through integrin-mediated mechanotransduction and cytoskeletal reorganization (27, 29, 30). Pathological dysregulation of ECM remodeling, frequently associated with tumor progression, establishes a permissive microenvironment conducive to cancer cell invasion and metastatic dissemination (30, 31). Functioning as a central signaling integrator, FAK mechanistically converges biomechanical and biochemical cues from the ECM to potentiate tumor cell motility and survival advantages. Specifically, FAK activation facilitates focal adhesion turnover, thereby enhancing dynamic interactions between neoplastic cells and the ECM - a prerequisite for invasive progression and metastatic colonization. Notably, mechanistic studies reveal that FAK cooperates with integrin β1 to drive bone metastasis in breast carcinoma through activation of downstream MAPK and Akt signaling axes (3). In urothelial malignancies, aberrant FAK signaling correlates with enhanced invasiveness and unfavorable prognosis, suggesting its potential involvement in mediating the critical transition from NMIBC to MIBC (27).

**Figure 1:**
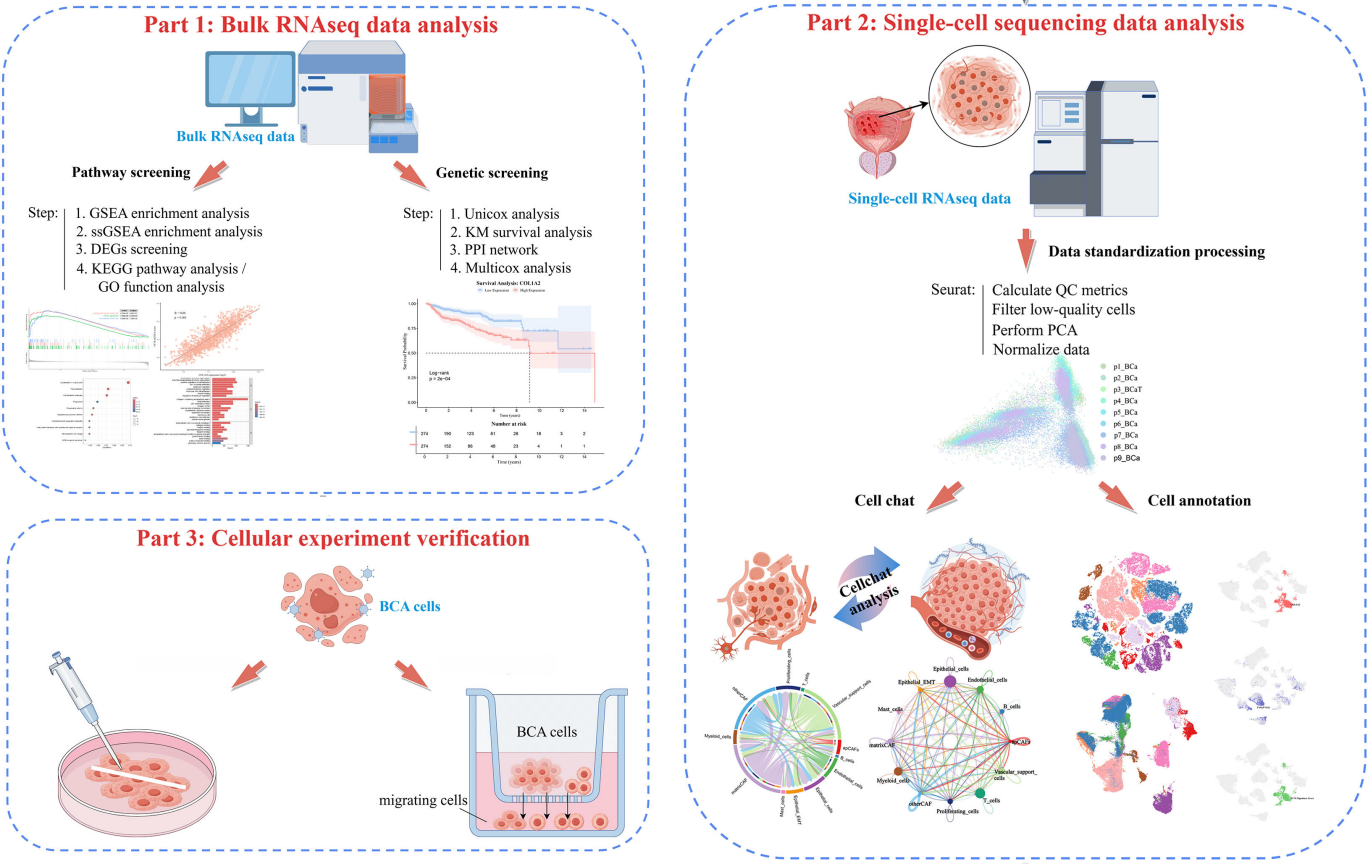
Bulk-RNAseq data analysis flowchart.

In this study, we conducted differential expression analysis on integrated transcriptomic datasets encompassing 421 bladder cancer patients from TCGA and 1,021 cases from GEO (**Figure 1**; **Supplementary Figure S1**). Prognostically relevant differentially expressed genes (DEGs) were refined through univariate Cox regression and Kaplan–Meier survival analyses. Multivariate Cox regression analysis incorporating multiple gene expression levels as covariates, coupled with protein-protein interaction (PPI) network analysis, identified four independent prognostic factors associated with the progression from NMIBC to MIBC. Genome-wide gene set enrichment analysis (GSEA) revealed a significant activation of extracellular matrix (ECM)-related pathways in MIBC. Single-sample GSEA (ssGSEA) further established strong correlations between these four key genes and ECM pathway activity, with COL1A2 emerging as a central orchestrator of stage progression. Mechanistically, COL1A2 modulates extracellular matrix remodeling, thereby triggering integrin–FAK signaling to drive muscle-invasive transformation. Collectively, our integrative multi-omics and experimental findings position COL1A2 as a vital molecular driver of NMIBC-to-MIBC progression via ECM pathway activation, underscoring its central role in bladder cancer stage evolution.

## 2 Materials and methods

### 2.1 Data acquisition and processing

Transcriptomic profiles and clinical annotations of bladder cancer patients were retrieved from TCGA (https://portal.gdc.cancer.gov) cohort and six GEO (https://www.ncbi.nlm.nih.gov/geo/) datasets (GSE13507, GSE48075, GSE32894, GSE31684, GSE83586, GSE37815). After rigorous removal of duplicate samples and cross-platform redundancies, the final cohort comprised 421 TCGA sample data and 1,021 GEO sample data. Raw expression data were harmonized through cross-platform normalization in R (v4.4.2). Batch effects were then corrected using the sva package, and gene identifiers were converted with biomaRt. This integrated dataset was further refined through quantile normalization to ensure analytical comparability across platforms. Single-cell RNA sequencing data were obtained from the GEO database under accession number GSE222315, comprising nine bladder cancer patients. Among these, samples from patients 2, 3, 4, and 5 included both tumor and adjacent normal tissues, whereas the remaining patients provided tumor-only data. Raw Seurat objects were loaded and annotated with unique sample-specific cell barcodes. Quality control involved excluding cells with fewer than 200 or more than 7,500 detected features, as well as those exhibiting over 20% mitochondrial gene content. Mitochondrial genes were annotated using biomaRt with reference to Ensembl gene identifiers. Following filtering, each dataset was normalized, highly variable features were identified, and data were scaled within individual samples. Samples were then merged and batch effects corrected using Harmony integration based on sample origin.

### 2.2 Prognostic screening of differentially expressed genes

Patient stratification was performed based on clinicopathological variables across datasets: NMIBC/MIBC classification in GSE13507 and GSE37815 utilized the “invasiveness” parameter, while “characteristics_T” variable defined disease stage in GSE48075, GSE32894, GSE31684, and GSE83586. For datasets lacking muscularis propria invasion status, TNM staging (“T1/Ta/Tis” vs ≥ T2) served as the stratification criterion, yielding 957 MIBC and 485 NMIBC cases. Differential expression analysis was conducted using the limma package with empirical Bayes moderated t-tests, applying thresholds of |log_2_FC| > 1 and FDR-adjusted *p* < 0.05. Volcano plot visualization was implemented via ggplot2. Prognostic DEG screening employed a two-tiered approach: univariate Cox regression (*p* < 0.05) followed by Kaplan-Meier stratification (log-rank *p* < 0.01; 5-year survival difference > 15%). The Benjamini-Hochberg method controlled false discovery rate (FDR < 0.05), with additional expression magnitude filtering (|log_2_FC| > 0.5). Consensus prognostic DEGs were derived through intersection analysis. PPI network construction utilized STRING (v10.5, confidence score > 0.4) and Cytoscape (v3.9.1) for topology visualization. Multivariate Cox regression (*p* < 0.001) coupled with network centrality metrics identified independent prognostic DEGs with both statistical significance and topological importance. We additionally performed time-dependent ROC analysis (1, 3, 5 years) and 5-fold cross-validation to assess predictive performance and generalizability.

### 2.3 Gene set enrichment analysis

To identify functionally enriched pathways between MIBC and NMIBC subtypes, we performed Gene Set Enrichment Analysis (GSEA). A ranked gene list was generated by sorting all protein-coding genes based on their log_2_-fold change (log_2_FC) from genome-wide differential expression. Curated KEGG pathway gene sets (v2023.2) were acquired from the Molecular Signatures Database (MSigDB) and filtered to retain pathways containing 15–500 genes. Enrichment analysis was executed via the GSEA algorithm implemented in the clusterProfiler package (v4.0) with 5,000 permutations to ensure statistical robustness. Significant pathways were defined using a dual-threshold criterion: false discovery rate (FDR) < 0.05 (Benjamini-Hochberg correction) and absolute normalized enrichment score (NES) > 1. Pathway activation directions were interpreted based on NES polarity, where positive NES indicated MIBC-associated enrichment and negative NES reflected NMIBC-specific patterns. Top enriched pathways (|NES| ranked top 3 per direction) were visualized using gseaplot2 to display enrichment peaks and leading-edge gene distributions.

### 2.4 Single sample GSEA

To investigate the association between core extracellular matrix (ECM)-related genes (CDH11, COL1A2, FLNC, LOX) and ECM pathway activity, we performed single-sample gene set enrichment analysis (ssGSEA). Enrichment scores for each sample were calculated using the MODULE_47 ECM gene set from MSigDB. We then assessed the correlation between the expression levels of each core gene and the corresponding ECM ssGSEA scores using Spearman’s rank correlation, a nonparametric method robust to non-normal distributions. Statistical significance was determined at *p* < 0.05, and results were visualized using scatter plots with regression lines, 95% confidence intervals, and embedded correlation coefficients (R) and p-values to illustrate the relationships. All analyses were conducted in R (v4.4.2) with the GSVA (v1.48.3) and ggplot2 (v3.4.3) packages.

### 2.5 Differential gene expression and functional enrichment analysis

The cohort of 1,442 BCA patients were stratified into COL1A2 high- and low-expression subgroups based on median COL1A2 mRNA levels. Differential gene expression analysis between these subgroups was performed using the limma R package, applying significance thresholds of *p* < 0.05 and absolute log_2_ fold change (|log_2_FC|) > 0.5. This analysis identified 1,083 differentially expressed genes (DEGs), comprising 183 downregulated and 900 upregulated transcripts. These DEGs were then functionally annotated through Kyoto Encyclopedia of Genes and Genomes (KEGG) pathway enrichment analysis and Gene Ontology (GO) classification, covering the molecular function, biological process, and cellular component domains. All analytical results were visualized using the ggplot2 package in R to generate publication-quality graphical representations.

### 2.6 Cell type annotation and clustering analysis of scRNA-seq data

Single-cell RNA-seq data were normalized using Seurat v4.0, followed by identification of 3,000 highly variable genes (HVGs) to construct the feature set. The data were then scaled, and dimensionality reduction was performed via principal component analysis (PCA). Major cell populations were delineated using the FindNeighbors and FindClusters functions with an initial resolution of 1.2, and clustering results were visualized using UMAP and t-SNE embeddings. Cell type annotation integrated cluster-specific differentially expressed genes (identified using FindAllMarkers with thresholds of min.pct = 0.25, log fold change > 0.25, and adjusted *p* < 0.01) alongside curated cell type–specific marker lists. Annotation was assigned when at least two canonical markers exhibited consistent expression patterns, with a mean log-normalized expression > 0.5, detected in over 30% of cells within the cluster, and with a specificity ratio (intra-cluster versus extra-cluster expression) exceeding 2. Fibroblast and EMT-like cell subsets were isolated for further analysis. Within these subsets, 2,000 HVGs were selected for re-normalization and scaling. PCA was performed retaining components accounting for 85% of cumulative variance. Subsequent clustering employed FindNeighbors and FindClusters at a resolution of 0.8, with visualization via UMAP and t-SNE. Subcluster annotation was guided by marker genes curated from literature and validated using the CellMarker 2.0 database (http://bio-bigdata.hrbmu.edu.cn/CellMarker).

### 2.7 Cell–cell communication landscape in the TME

To systematically investigate intercellular communication within the tumor microenvironment (TME), we employed the “CellChat” R package to infer and analyze cell–cell interactions based on a curated database of ligand–receptor pairs. The analysis was performed on 12 transcriptionally defined cell subclusters derived from single-cell RNA sequencing data. Statistically enriched ligand–receptor interactions were identified using the Wilcoxon rank-sum test implemented in CellChat. Subsequently, a global cell–cell interaction network was constructed and quantified from the perspective of communication probability. Communication roles (sender, receiver, mediator, influencer) of each cell subcluster were inferred by calculating various network centrality measures. For pathway-level signaling analysis, signaling networks corresponding to ECM–integrin–FAK related pathways—including COLLAGEN, LAMININ, and FN1—were extracted and analyzed independently. Next, dominant ligand–receptor pairs involved in matrixCAF→Epithelial_EMT communication were identified using built-in functions such as rankNet, and their contribution to pathway-specific communication was evaluated. The spatial pattern of communication was visualized using circle plots, chord diagrams, and bubble plots provided by the CellChat toolkit. To further dissect signaling pattern heterogeneity, we applied the non-negative matrix factorization (NMF) algorithm to identify major incoming and outgoing communication patterns across the 12 cell types. The optimal number of communication patterns was determined using cophenetic correlation and silhouette scores. Finally, associations between identified signaling patterns, specific ligand–receptor pairs, and cell subpopulations were visualized to facilitate interpretation of complex interaction dynamics.

### 2.8 Cell culture and transfection

Human bladder cell lines modeling distinct pathological stages were utilized: SV-HUC-1 (non-malignant urothelium), T24 (muscle-invasive phenotype), and SW1710 (non-muscle-invasive phenotype) (Chinese Academy of Sciences Cell Bank). All lines were maintained at 37 °C/5% CO_2_ in humidified incubators. SW1710 was cultured in RPMI-1640 (Procell), while SV-HUC-1 and T24 were grown in Ham’s F12K (Procell), both supplemented with 10% heat-inactivated FBS (Gibco) and 1% penicillin-streptomycin (Beyotime).

To investigate COL1A2’s stage-specific functions, SW1710 (low-COL1A2) was selected for gain-of-function studies and T24 (high-COL1A2) for loss-of-function analysis. For transient overexpression, SW1710 cells at 70% confluency were transfected with pcDNA3.1-COL1A2 plasmid (GenScript) using InviTranGene^®^ reagent (Polyplus; 1:3 DNA: reagent ratio). For targeted knockdown, T24 cells were reverse-transfected at 60–70% confluency in 6-well plates (2×10^5^ cells/well) with 50 nM COL1A2-specific or non-targeting control siRNA (Lipofectamine 3000, Invitrogen), with complexes formed in Opti-MEM and incubated 15 min prior to application. Post-transfection procedures included medium replacement with complete RPMI-1640/10% FBS at 6 hours. Knockdown efficiency was rigorously assessed at 48h via qRT-PCR. All manipulations adhered to aseptic protocols.

### 2.9 Total RNA isolation and quantitative RT−PCR

Total RNA was extracted from cells using TRIzol reagent (Vazyme, China), followed by cDNA synthesis with reverse transcriptase (PrimeScript RT Kit, Takara). Quantitative PCR (qPCR) was performed in technical triplicates using SYBR Green Master Mix (Applied Biosystems) on a real-time PCR system under standardized cycling conditions. Gene expression levels were normalized to β-actin and calculated using the 2^−ΔΔCt method. Primer sequences are provided in the **Supplementary Table S1**.

### 2.10 Wound healing assay

Cells were seeded in 6-well culture plates at a density of 5×10^5^ cells/well and incubated until 90-95% confluency. Sterile 200 μl pipette tips (Corning) were used to generate uniform linear wounds through the central axis of each well. After three PBS washes to remove detached cells, wounded monolayers were maintained in serum-free medium (Gibco). Phase-contrast images (10× magnification) were captured at 0, 24 and 48 h post-scratching using an inverted microscope (Olympus IX73) equipped with an environmental chamber maintaining 5% CO_2_ and 37°C. Quantification of migration capacity was performed by measuring residual wound area using ImageJ software (NIH) with the following formula: Migration rate (%) = [(A_0_ - A_t_)/A_0_] × 100 (where A_t_ represents initial wound area and A_t_ denotes area at timepoint t).

### 2.11 Transwell-based invasion analysis

Cell migration and invasion assays were performed using 8 μm pore Transwell inserts (Corning 3422) with Matrigel coating, respectively. SW1710 and T24 cells (4–5 × 10^4^ cells per well) were seeded in the upper chamber in serum-free medium. The lower chamber was filled with RPMI-1640 or Ham’ s F12K medium, each supplemented with 10% FBS. After 24 hours incubation at 37 °C, cells that had invaded to the lower membrane surface were fixed with 4% paraformaldehyde, stained with 0.1% crystal violet, and imaged using a Leica light microscope. Invasive capacities were quantified by counting stained cells in four randomly selected fields per insert.

### 2.12 Statistical method

Data are expressed as mean ± standard deviation (SD) from three independent experiments. Graphs were generated using the ggplot2 package (v3.4.4) in R, and statistical analyses were performed with GraphPad Prism (v10.1.2; GraphPad Software, Inc.). Statistical significance was determined by two-sided p-values < 0.05, in accordance with American Statistical Association recommendations for biological research.

## 3 Results

### 3.1 Screening and analysis of DEGs

**Figure 2:**
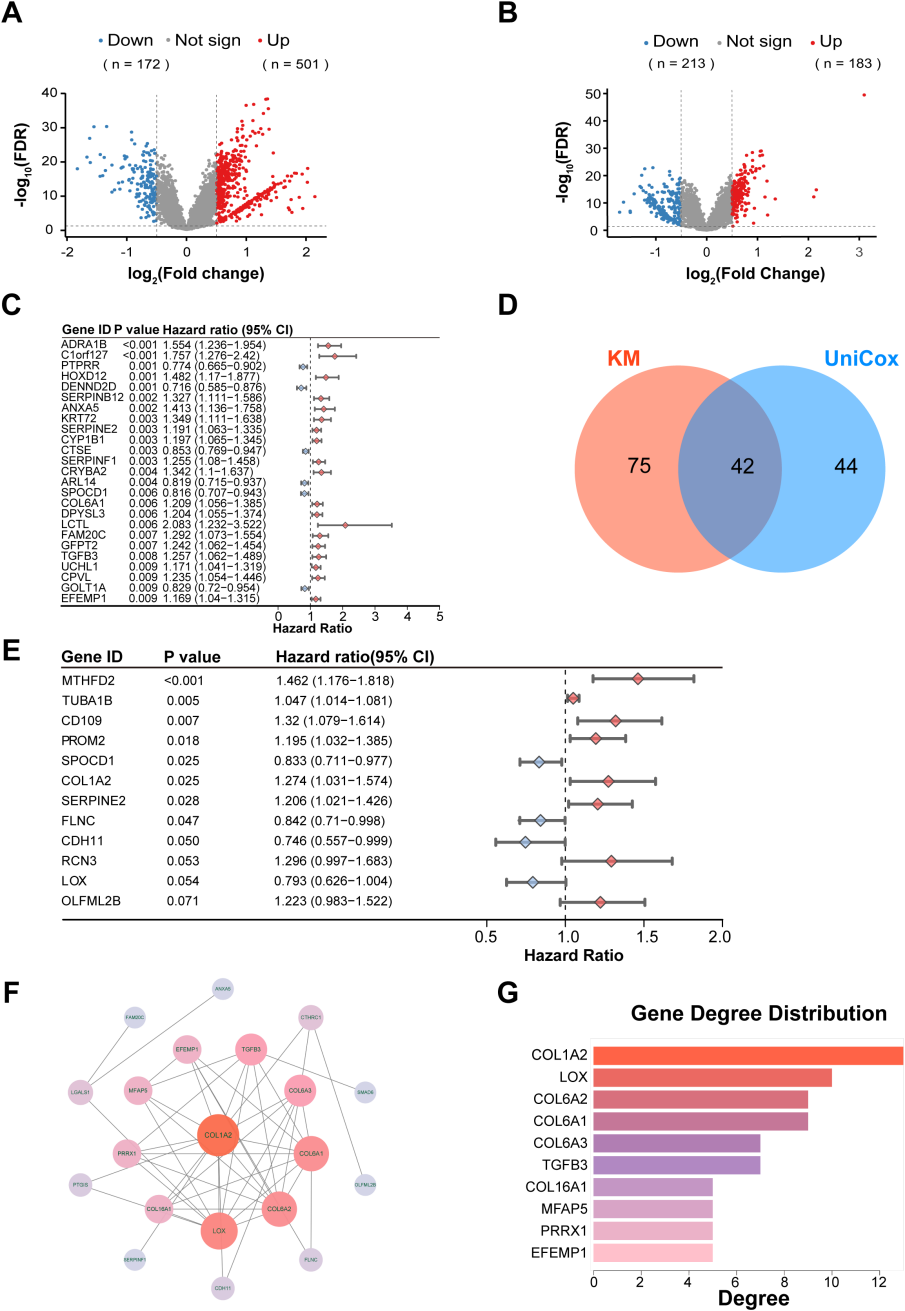
Screening and validation of DEGs in BCA. **(A)** Volcano plot showing upregulated and downregulated DEGs between MIBC and NMIBC groups in the GEO BCA dataset. **(B)** Volcano plot displaying DEGs between MIBC and NMIBC in the combined GEO and TCGA BCA cohorts. **(C)** Forest plot presenting univariate Cox regression results identifying DEGs significantly associated with patient survival. **(D)** Venn diagram illustrating the intersection of DEGs identified by Kaplan–Meier survival and univariate Cox analyses. **(E)** Forest plot showing multivariate Cox regression analysis identifying independent prognostic DEGs. **(F)** Protein-protein interaction network highlighting interactions among DEGs and identifying hub genes. **(G)** Bar plot ranking hub genes by node degree within the PPI network.

**Figure 3:**
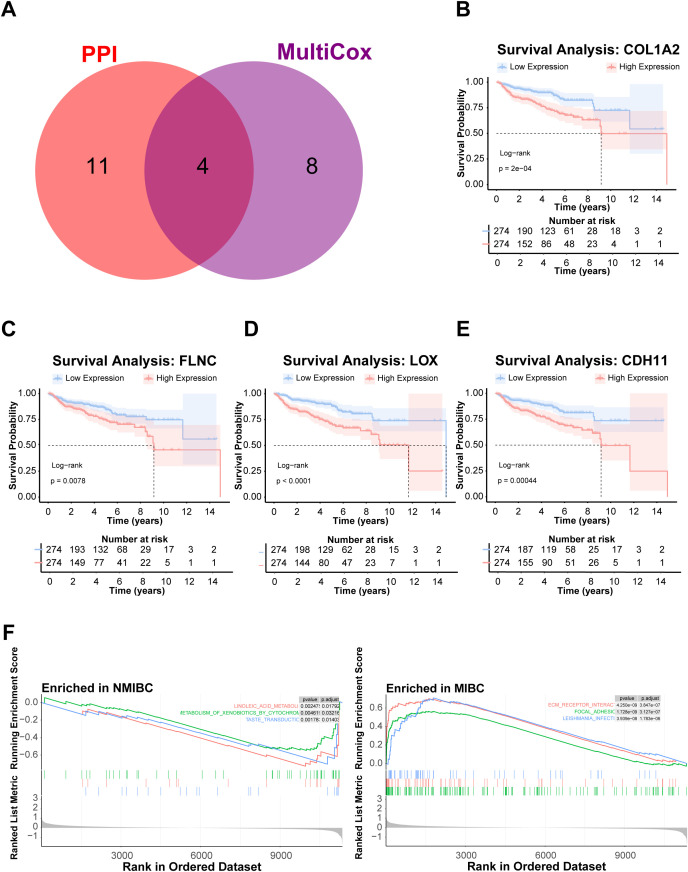
Analysis of prognostic DEGs and pathway enrichment in MIBC. **(A)** Venn diagram showing the intersection of prognostic DEGs identified by multivariate Cox regression and core hub genes from the PPI network. **(B–E)** Kaplan–Meier survival curves depicting overall survival differences stratified by expression levels of COL1A2, FLNC, LOX, and CDH11, respectively. **(F)** GSEA enrichment plot comparing pathway activation between MIBC and NMIBC groups, highlighting extracellular matrix–receptor interaction as the most significantly upregulated pathway in MIBC.

We conducted a comprehensive transcriptomic analysis by first aggregating six independent GEO datasets (**Supplementary Table S2**) after standardized preprocessing. Differential expression analysis within this combined GEO cohort identified 673 significantly dysregulated genes (FDR < 0.05, | log_2_FC | > 0.5), comprising 172 downregulated and 501 upregulated genes in MIBC relative to NMIBC (**Figure 2A**). Subsequently, we integrated these six GEO datasets with the TCGA-BCA cohort (**Supplementary Table S3**), forming a unified dataset of 1,442 bladder cancer patients (957 MIBC and 485 NMIBC). Re-analysis of this expanded cohort revealed 396 significantly dysregulated genes associated with MIBC progression (FDR < 0.05, |log_2_FC| > 0.5), with 213 downregulated and 183 upregulated (**Figure 2B**; **Supplementary Table S4**). This two-tiered approach underscores the robustness of our gene signature identification across diverse datasets and patient populations. To elucidate the prognostic implications of these DEGs, we performed a comprehensive survival analysis framework. Initial univariate Cox regression (**Figure 2C**; **Supplementary Table S5**, *p* < 0.05) followed by Kaplan-Meier evaluation with stringent criteria (**Supplementary Table S6**, *p* < 0.01 and 5-year survival difference > 15%) revealed 42 survival-associated DEGs (**Figure 2D**). Subsequent multivariate Cox regression identified 12 independent prognostic markers (**Figure 2E**; **Supplementary Table S7**), while protein-protein interaction network analysis (STRING database) highlighted 15 hub genes with node degrees > 1 (**Figures 2F, G**; **Supplementary Table S8**). The intersection of these two analytical approaches yielded four pivotal DEGs demonstrating both prognostic significance and network centrality. Among them, four genes, CDH11, COL1A2, FLNC and LOX were identified as prognostic factors for our subsequent study (**Figure 3A**). Show Kaplan-Meier survival curves for the four core genes (**Figures 3B–E**).

### 3.2 ECM remodeling emerges as critical determinant of tumor stage transition

To delineate pathway-level distinctions between MIBC and NMIBC molecular subtypes, we performed genome-wide Gene Set Enrichment Analysis (GSEA) using pre-ranked gene lists based on differential expression log_2_-fold change (log_2_FC) values. The analysis was implemented using the clusterProfiler package (v4.0) with KEGG pathway annotations from the Molecular Signatures Database (MSigDB c2.cp.kegg_legacy.v2023.2.Hs). A ranked list metric incorporating log_2_FC values for all protein-coding genes was subjected to 1,000 permutations using the GSEA function, with significance thresholds set at false discovery rate (FDR) <0.25 and normalized enrichment score (NES) magnitude >1.5. Pathway activation directionality was interpreted through NES polarity, where positive NES indicated MIBC-associated activation and negative NES reflected NMIBC-specific patterns.

The GSEA landscape revealed extracellular matrix (ECM)-receptor interaction as the most prominently enriched pathway (NES = 2.33, *p* = 2.01), demonstrating coordinated upregulation in MIBC specimens (**Figure 3F**; **Supplementary Table S9**). Notably, this pathway exhibited the highest combined ranking score across all analyzed gene sets, underscoring its central role in bladder cancer invasiveness progression.

### 3.3 ssGSEA reveals COL1A2 as a central mediator of ECM remodeling in bladder cancer

**Figure 4:**
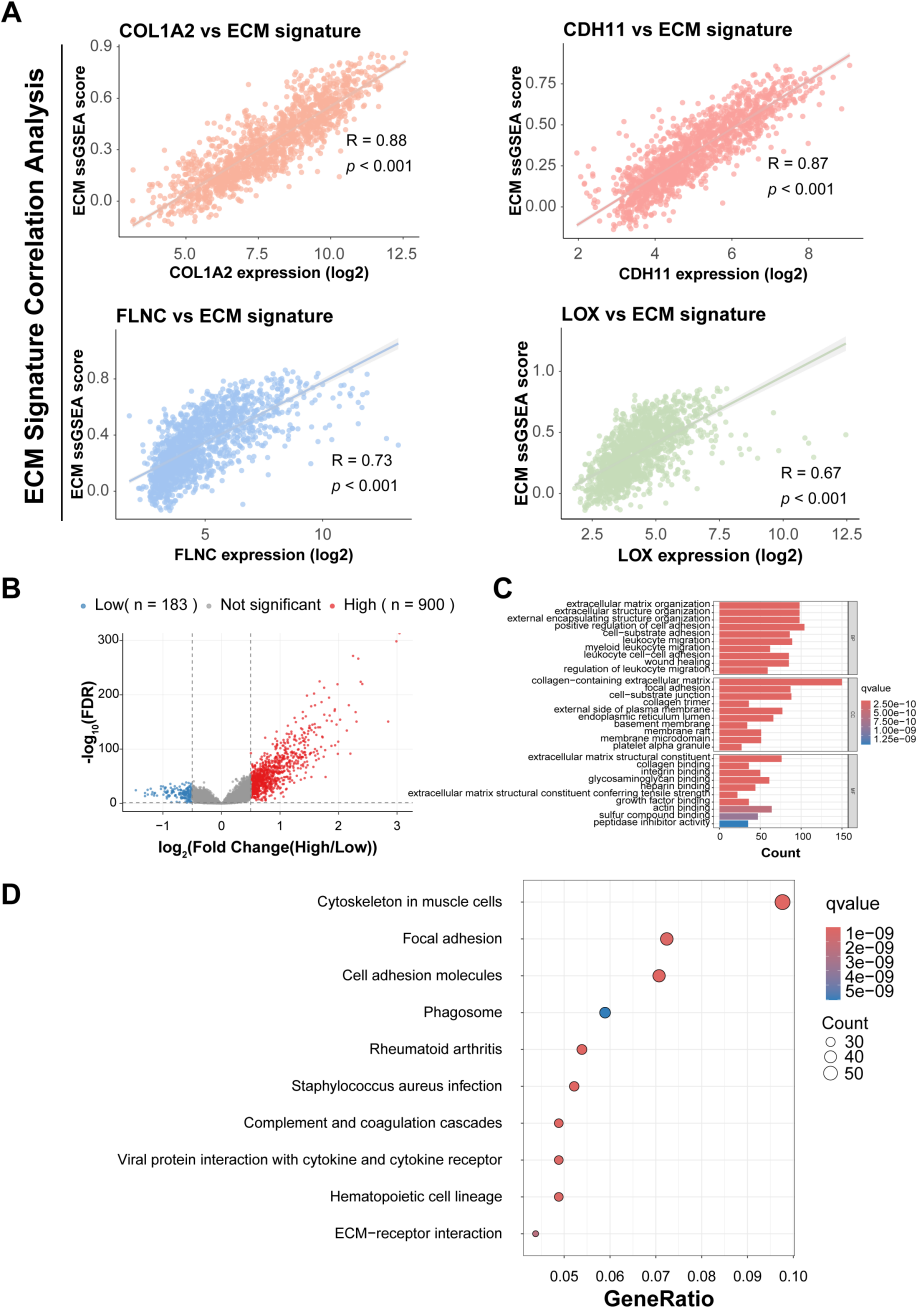
ECM pathway activity and COL1A2-associated transcriptomic profiling in BCA. **(A)** Scatter plots showing correlations between ECM pathway ssGSEA scores and expression levels of prognostic genes CDH11, COL1A2, FLNC, and LOX, highlighting COL1A2 as most strongly correlated. **(B)** Volcano plot depicting differentially expressed genes between high and low COL1A2 expression groups in BCA samples. **(C)** Bar plot illustrating Gene Ontology enrichment analysis of DEGs associated with COL1A2 expression, emphasizing ECM organization and related processes. **(D)** Bubble plot displaying KEGG pathway enrichment results for COL1A2-associated DEGs, with focal adhesion among the top enriched pathways.

To quantify ECM pathway activity in bladder cancer, we applied single-sample gene set enrichment analysis (ssGSEA) using a curated ECM gene set. ECM signature scores exhibited marked inter-sample heterogeneity, indicating differential pathway activation across bladder cancer specimens. Correlation analysis demonstrated that among the four core prognostic genes (CDH11, COL1A2, FLNC, and LOX), COL1A2 expression was most strongly correlated with ECM scores (**Figure 4A**, Spearman’s R = 0.88, *p* < 0.001). This robust correlation positioned COL1A2 as a central regulator of ECM remodeling, justifying its selection for functional validation.

### 3.4 Clinical prognostic relevance of COL1A2 expression in bladder cancer

To elucidate the prognostic and therapeutic significance of COL1A2 in MIBC, we performed an integrative analysis of the TCGA-BCA cohort alongside six independent GEO datasets. Comparative evaluation revealed a marked upregulation of COL1A2 expression in MIBC relative to NMIBC. Kaplan–Meier survival analysis demonstrated a significant difference in overall survival (OS) between patients with high and low COL1A2 expression (log-rank *p* < 0.001). Univariate Cox proportional hazards regression identified COL1A2 expression as significantly associated with OS (hazard ratio [HR] 1.123; 95% confidence interval [CI] 1.006–1.254; *p* = 0.03). Subsequent multivariate Cox analysis, incorporating the expression levels of multiple genes, confirmed COL1A2 as an independent prognostic factor for OS (HR 1.274; 95% CI 1.031–1.574; *p* = 0.025). What’s more, COL1A2 demonstrated consistent predictive accuracy, with time-dependent AUC values of 0.603, 0.630, and 0.647 at 1, 3, and 5 years (**Supplementary Figure S2**). Cross-validation confirmed model stability (**Supplementary Figure S3**). Collectively, these findings position COL1A2 as a promising biomarker for monitoring the progression from NMIBC to MIBC and for predicting invasive tumor behavior. The strong association with clinical outcomes underscores its dual potential as both a prognostic indicator and a therapeutic target in bladder cancer, particularly for intercepting disease progression in high-risk patients.

### 3.5 Focal adhesion as a key downstream effector of COL1A2-mediated ECM remodeling

To elucidate downstream pathways regulated by COL1A2-ECM signaling, bladder cancer samples were stratified into high and low-COL1A2 expression groups. Differential expression analysis using the limma package identified 1,083 significantly dysregulated genes (**Figure 4B**; **Supplementary Table S10**, | log_2_FC | > 0.5, FDR < 0.05), whose distribution was visualized by volcano plot. Functional enrichment analysis of these DEGs revealed significant overrepresentation of Gene Ontology terms related to extracellular matrix organization, cell adhesion, and cytoskeletal remodeling. Complementary Kyoto Encyclopedia of Genes and Genomes pathway analysis further highlighted focal adhesion as a top enriched pathway associated with COL1A2 expression. Supported by stringent statistical thresholds (*p* < 0.05, q < 0.05), these results provide mechanistic insight into how COL1A2 may facilitate bladder cancer progression through modulation of cell–matrix interactions. The enrichment landscape was depicted through barplots and bubble plots illustrating pathway significance and gene ratios, underscoring focal adhesion’s pivotal role downstream of COL1A2-mediated ECM remodeling (**Figures 4C, D**).

### 3.6 Single-cell transcriptomic profiling and cell type annotation

**Figure 5:**
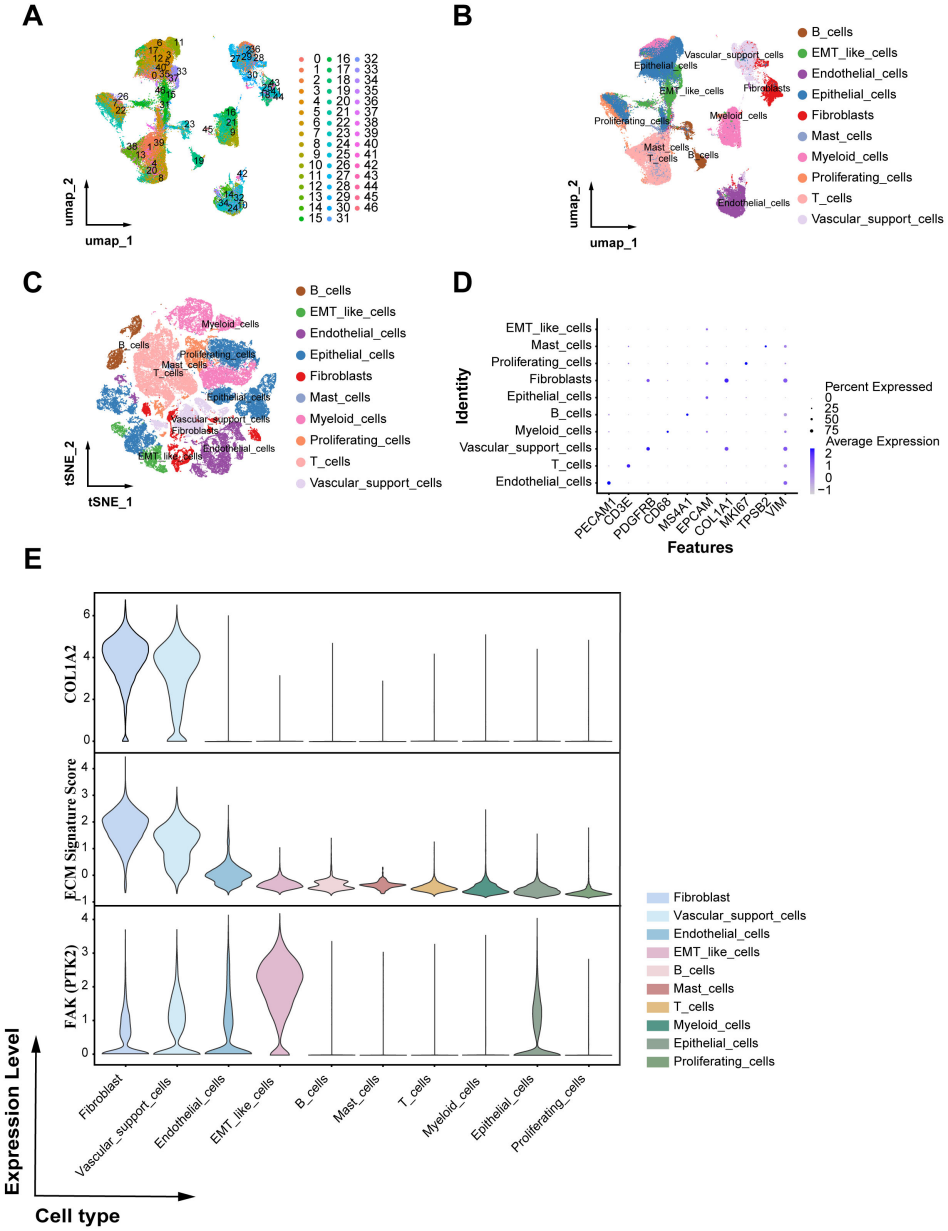
Single-cell transcriptomic profiling and cellular origin of COL1A2 in bladder cancer. **(A)** UMAP clustering displaying 46 distinct cell clusters derived from scRNA-seq data. **(B)** UMAP visualization with cell type annotations identifying 10 major cell populations based on canonical markers. **(C)** t-SNE plot depicting the annotation of nine bladder cancer patient samples into 10 major cell lineages. **(D)** Dot plot showing the expression of lineage-specific marker genes across the annotated cell populations. **(E)** Violin plots illustrating expression levels of COL1A2, ECM signature scores, and PTK2 across the 10 major cell lineages.

To further elucidate the cellular origin of COL1A2 and its role within the tumor microenvironment, we integrated publicly available single-cell RNA sequencing (scRNA-seq) data (GSE222315) comprising nine bladder cancer patient samples. Following rigorous quality control and preprocessing, a total of 74,236 high-quality cells were retained for downstream analyses. After normalization, batch integration, and dimensionality reduction using principal component analysis (PCA), unsupervised clustering identified 46 distinct cell clusters (**Figure 5A**; **Supplementary Table S11**). Based on canonical marker genes and reference-based annotation (**Supplementary Table S12**), these clusters were categorized into 10 major cell lineages, including epithelial cells, T cells, B cells, myeloid cells, fibroblasts, endothelial cells, mast cells, proliferating cells, vascular support cells, and EMT-like cells (**Figures 5B, C**). Given the pivotal role of COL1A2 highlighted in our bulk RNA sequencing (bulk RNA-seq) analysis, we next examined its single-cell expression pattern. Notably, COL1A2 and ECM-related markers showed high and specific expression within the fibroblast population (clusters 18, 25, 28, 35, 36, 41, 43, 44), while FAK-related genes exhibited the highest expression within the EMT-like cell population (**Figures 5D, E**, clusters 15, 33).

**Figure 6:**
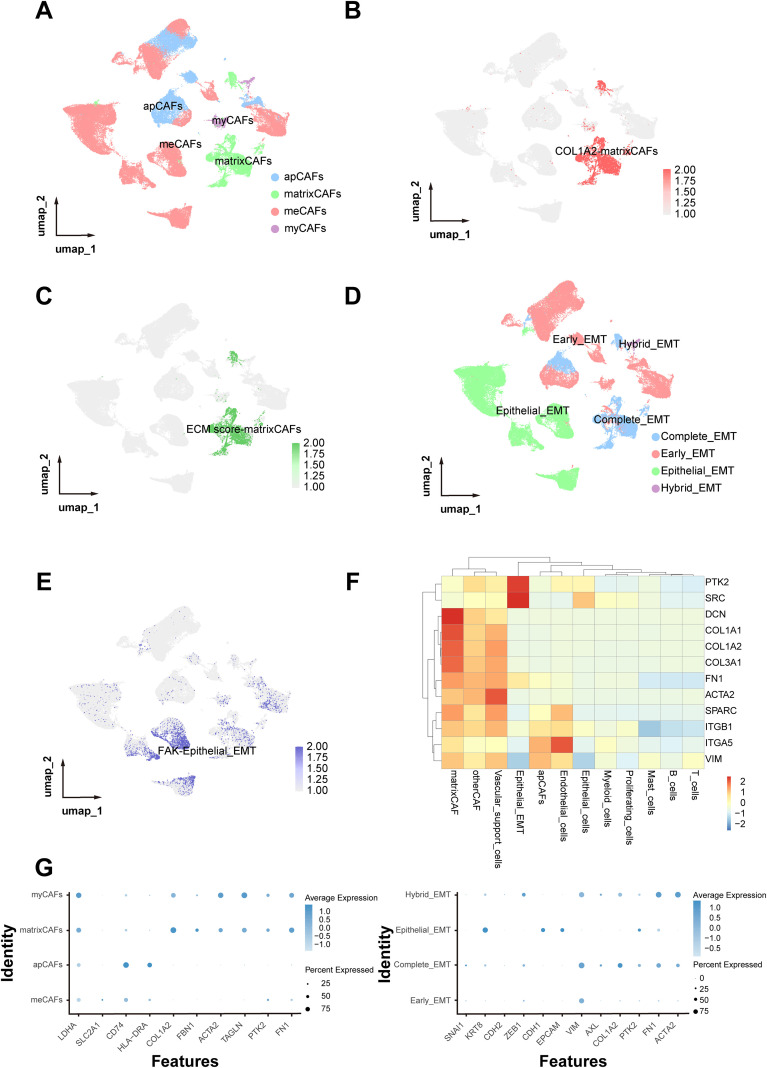
Subclassification of bladder cancer-associated fibroblasts and EMT-like cells. **(A)** UMAP plot showing four distinct cancer-associated fibroblast (CAF) subtypes: antigen-presenting (apCAF), myofibroblastic (myCAF), matrix-producing (matrixCAF), and metabolism-associated CAFs (meCAF). **(B, C)** Feature plots displaying high expression of COL1A2 and ECM-related genes across matrixCAFs. **(D)** UMAP plot depicting four EMT-associated cells subclusters: Complete_EMT, Early_EMT, Epithelial_EMT, and Hybrid_EMT. **(E)** Feature plots displaying high expression of PTK2 (FAK) across Epithelial_EMT cells. **(F)** Heatmap showing the expression of major marker genes for key cell subtypes across CAF and EMT-like populations. **(G)** Dot plot illustrating marker gene expression levels across the CAF subtypes and Epithelial_EMT cells.

To further resolve fibroblast heterogeneity, subclustering of fibroblast cells identified four distinct cancer-associated fibroblast (CAF) subtypes: Antigen-presenting cancer-associated fibroblasts (apCAFs), Myofibroblastic cancer-associated fibroblasts (myCAFs), matrix-producing cancer-associated fibroblasts (matrixCAFs), and Metabolism-associated cancer-associated fibroblasts (meCAFs) (**Figure 6A**). Among these, matrixCAFs displayed the highest expression of COL1A2 and ECM-related genes (**Figures 6B, C**). Similarly, subclustering of EMT-like cells revealed four EMT-associated epithelial subgroups: Complete_EMT, Early_EMT, Epithelial_EMT, and Hybrid_EMT (**Figure 6D**). Notably, FAK (PTK2) was predominantly expressed in the Epithelial_EMT subgroup (**Figure 6E**), suggesting a potential cellular axis linking COL1A2-mediated ECM remodeling and FAK-driven EMT progression. Display the important marker genes of each cell type through heat maps and dot plots (**Figures 6F, G**).

### 3.7 Cell–cell communication analysis between matrixCAFs and epithelial_EMT cells

To elucidate the cellular crosstalk underpinning the COL1A2–ECM–FAK signaling axis identified in bulk RNA-seq, we applied CellChat to dissect intercellular communication networks within the tumor microenvironment. Our analysis focused on the interaction dynamics between COL1A2/ECM-high matrix cancer-associated fibroblasts (matrixCAFs) and PTK2 (FAK)-high Epithelial_EMT cells, hypothesized as critical mediators driving the transition from NMIBC to MIBC.

**Figure 7:**
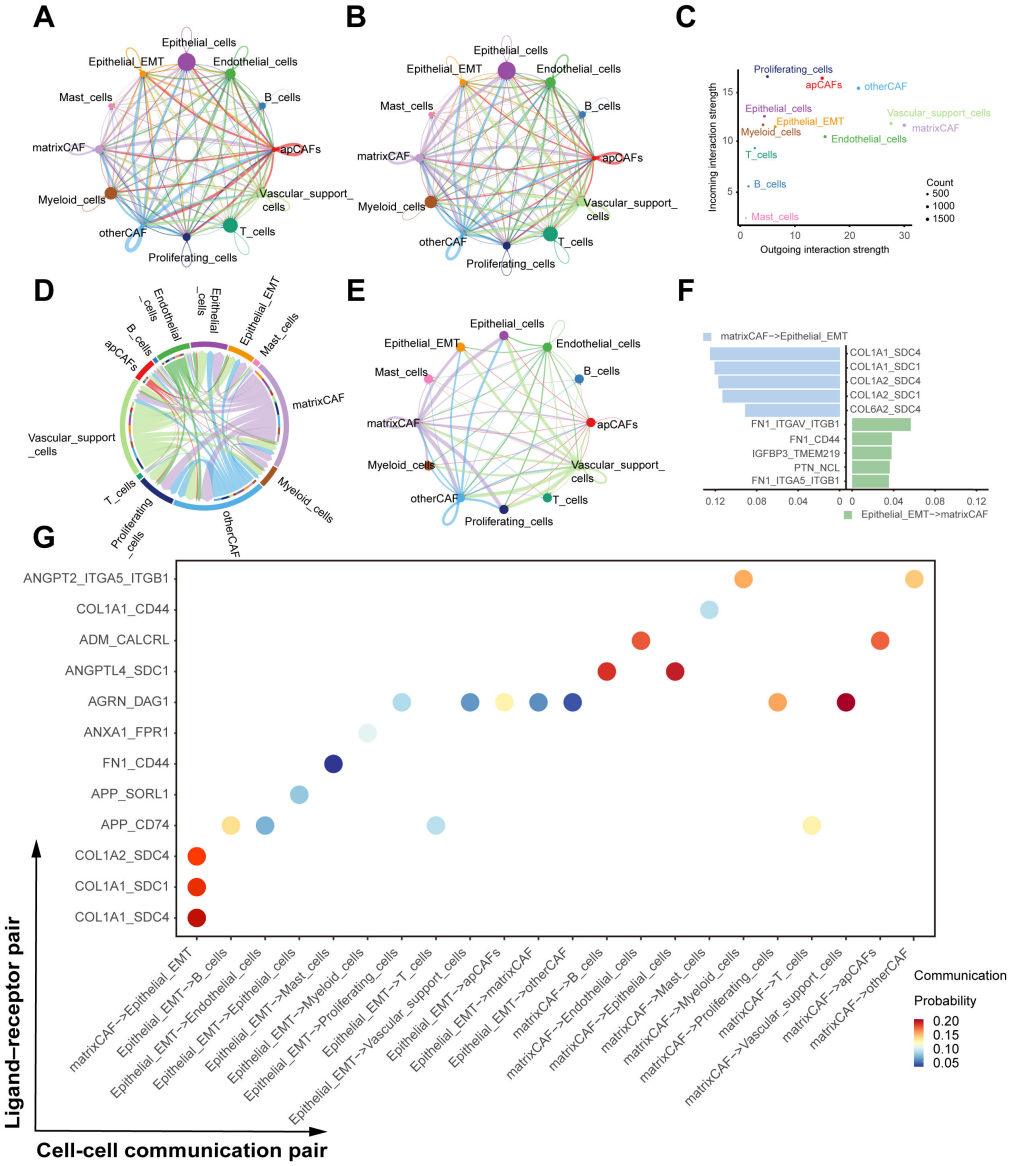
Cell-cell communication analysis highlighting COL1A2 - ECM - FAK axis in bladder cancer. **(A)** The number of interactions in cell-cell communication network. **(B)** The interaction weights/strength in cell-cell communication network. **(C)** Network centrality analysis identifying matrixCAFs as predominant signal senders and Epithelial_EMT cells as principal receivers. **(D)** Chord diagram illustrating collagen pathway-mediated signaling interactions, emphasizing communication from matrixCAFs to Epithelial_EMT cells. **(E)** Circular network visualization of collagen-associated pathways between matrixCAF and Epithelial_EMT populations. **(F)** Bar plot ranking ligand–receptor pairs by communication strength between matrixCAF and Epithelial_EMT populations. **(G)** Bubble plot showing ligand–receptor interaction probabilities between matrixCAF and Epithelial_EMT cells, with COL1A1–SDC4 as the strongest pair.

CellChat revealed frequent and robust communication signals from matrixCAFs to Epithelial_EMT populations (**Figures 7A, B**). Centrality analyses identified matrixCAFs as predominant signal senders, with Epithelial_EMT cells acting as principal signal receivers (**Figure 7C**). Interrogation of specific pathways, particularly through pathway_COLLAGEN visualizations (chord and network diagrams), further substantiated the strength and specificity of ECM-related signaling between these populations (**Figures 7D, E**). The chord diagrams and network graphs highlighted a particularly intense signaling flux from matrixCAFs to Epithelial_EMT cells through collagen-based pathways, reinforcing the functional significance of COL1A2-mediated ECM remodeling in modulating EMT.

Among specific ligand-receptor pairs, COL1A1–SDC4 exhibited the highest communication probability, followed by COL1A2–SDC4 and COL1A1–SDC1, indicating syndecan-mediated receptor engagement as a key downstream effector axis (**Figures 7F, G**). This signaling interaction suggests that ECM components secreted by matrixCAFs may activate syndecan-dependent pathways in Epithelial_EMT cells, thereby facilitating FAK activation and promoting a mesenchymal, invasive phenotype.

Collectively, these cell-cell communication analyses provide mechanistic insight into how matrixCAF-derived ECM signals orchestrate epithelial plasticity and invasive progression in bladder cancer, highlighting a spatial and functional signaling axis essential for NMIBC-to-MIBC transition.

### 3.8 COL1A2-Driven ECM remodeling activates integrin-FAK signaling to promote bladder cancer invasion

**Figure 8:**
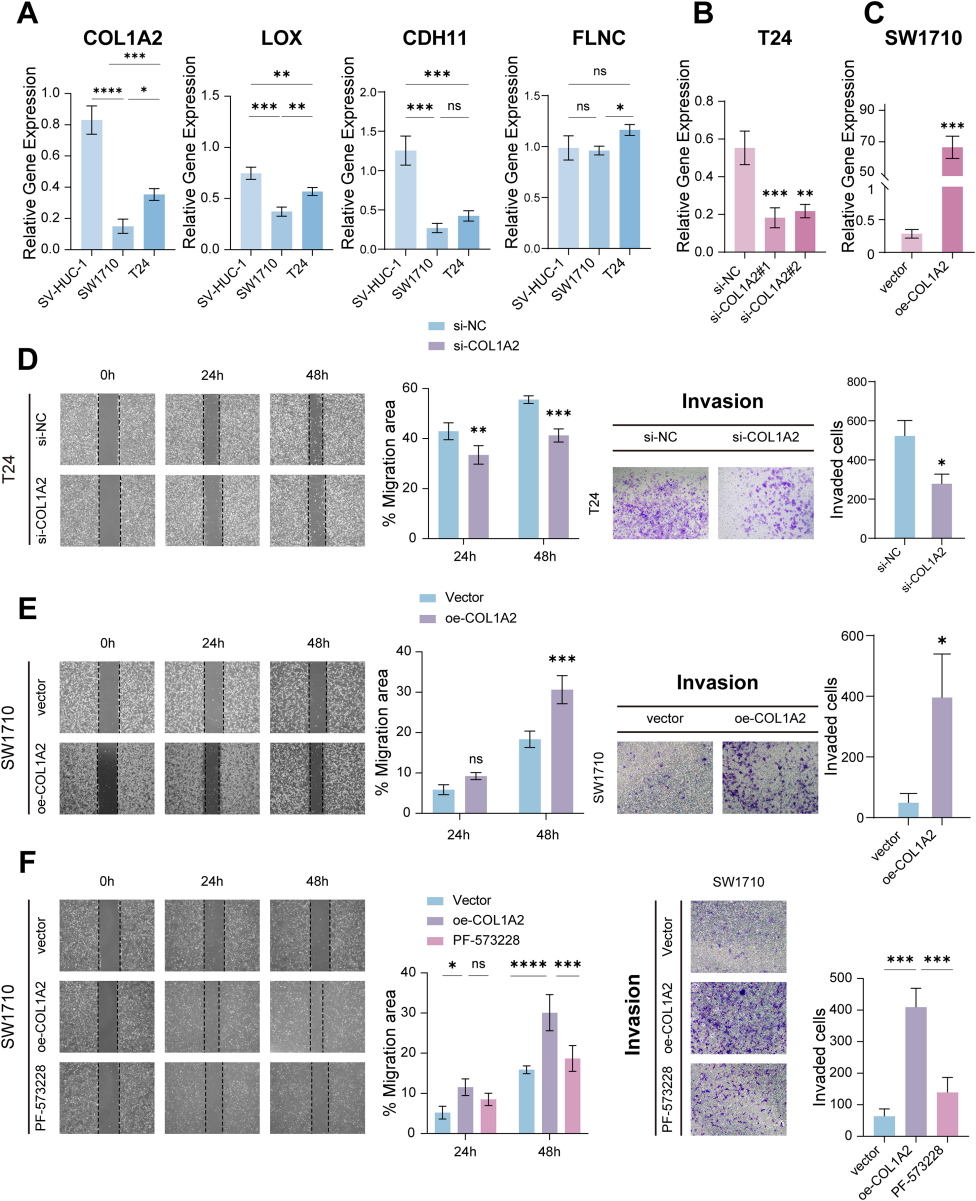
COL1A2 activates downstream FAK signaling to promote bladder cancer invasion. **(A)** RT-qPCR analysis of COL1A2, LOX, FLNC, and CDH11 expression levels across bladder cancer cell lines with varying invasiveness. **(B)** RT-qPCR showing COL1A2 expression in bladder cancer cells transfected with si-COL1A2. **(C)** RT-qPCR analysis of COL1A2 expression in bladder cancer cells transfected with vector control or COL1A2 overexpression plasmid. **(D)** Representative Transwell invasion and wound healing assays following COL1A2 knockdown in bladder cancer cells. **(E)** Representative Transwell invsion and wound healing assays of SW1710 cells overexpressing COL1A2. **(F)** Representative Transwell invasion and wound healing assays of COL1A2-overexpressing SW1710 cells treated with the selective ATP-competitive FAK inhibitor PF-573228. The asterisks denote statistical significance: *p < 0.05, **p < 0.01, ***p < 0.001, ****p < 0.0001, and ns indicating not statistically significant.

Leveraging the established role of COL1A2 in orchestrating extracellular matrix (ECM) remodeling as a pivotal driver of bladder cancer progression, we first characterized its transcriptional profile across a panel of bladder cancer cell lines modeling distinct pathological stages (normal urothelium: SV-HUC-1; low-invasive/NMIBC-like: SW1710; highly invasive/MIBC-like: T24). Quantitative RT-PCR revealed stage-specific dysregulation: COL1A2 was significantly downregulated in SW1710 cells relative to normal urothelium, but markedly re-expressed in invasive T24 cells, indicating its reactivation correlates with aggressive phenotypes and suggesting a role in the NMIBC-to-MIBC transition (**Figure 8A**).

To functionally establish COL1A2 as a driver of invasiveness, we performed gain- and loss-of-function studies in representative models (**Figures 8B, C**). COL1A2 knockdown in T24 cells significantly attenuated migratory and invasive capacities in wound healing and Transwell assays (**Figure 8D**). Conversely, COL1A2 overexpression in SW1710 cells induced a pro-metastatic phenotype (**Figure 8E**). The experiments confirmed the causal role of COL1A2 in promoting bladder cancer aggressiveness.

Given COL1A2’s pivotal role in ECM remodeling and FAK’s essential function in integrin-mediated signal transduction, we evaluated the impact of FAK inhibition on COL1A2-driven invasiveness in SW1710 bladder cancer cells. We established COL1A2-overexpressing SW1710 cells and assessed their invasive capacity and migratory ability using Transwell invasion and wound healing assays, respectively. Compared to control cells, COL1A2 overexpression significantly enhanced both invasion and migration. However, treatment with the selective ATP-competitive FAK inhibitor PF-573228 markedly suppressed these effects, resulting in a substantial decrease in wound closure and Transwell invasion relative to the COL1A2-overexpressing group (**Figure 8F**). These findings demonstrate that FAK activity is critical for mediating the pro-invasive phenotype induced by COL1A2 overexpression in SW1710 cell.

Collectively, our data delineate a COL1A2-driven signaling axis wherein ECM remodeling activates integrin-FAK signaling cascades, thereby facilitating the transition from non–muscle-invasive to muscle-invasive bladder cancer. This COL1A2/ECM/FAK axis constitutes a critical molecular framework underpinning bladder cancer progression and represents a promising therapeutic target for mitigating invasiveness and metastasis.

## 4 Discussion

The dynamic remodeling of ECM constitutes a critical determinant of tumor progression, serving as a mandatory prerequisite for both primary lesion expansion and metastatic niche formation (32, 33). Emerging molecular genetic evidence underscores distinct pathogenetic pathways governing non-muscle-invasive bladder cancer NMIBC and MIBC, with ECM dysregulation emerging as a hallmark of disease aggressiveness (34). Notably, accumulating studies have identified specific ECM-related genes that orchestrate matrix composition and biomechanical properties, which strongly correlate with advanced tumor stage and unfavorable prognosis in bladder cancer (8). Despite these advances, current prognostic models and imaging modalities remain insufficient for reliable risk stratification or accurate prediction of Urothelial bladder cancer (UBC) recurrence and survival outcomes, particularly in distinguishing indolent NMIBC from lethal MIBC phenotypes (35, 36). This knowledge gap underscores an urgent need to dissect the ECM-driven molecular networks underlying the NMIBC-to-MIBC transition, with the dual objectives of identifying stage-specific prognostic biomarkers and developing ECM-targeted therapeutic strategies to intercept metastatic progression.

Integrated transcriptomic profiling of TCGA and GEO cohorts identified DEGs associated with bladder cancer progression (37–40). Rigorous bioinformatic screening—incorporating Cox regression and survival analyses—revealed CDH11, COL1A2, FLNC, and LOX as independent prognostic determinants for the NMIBC-to-MIBC transition. GSEA of pan-bladder cancer samples ranked ECM-related pathways as the most significantly enriched processes, with ECM-receptor interaction and collagen fibril organization being top dysregulated mechanisms. Strikingly, ssGSEA demonstrated COL1A2 as the paramount ECM pathway activator, exhibiting stronger correlations with invasive phenotypes than other candidates. This multi-cohort approach not only prioritizes COL1A2 as a mechanistically pivotal target but also establishes a paradigm for translating bulk omics data into stage-specific therapeutic strategies.

Our ssGSEA analysis revealed a robust association between COL1A2 and the ECM pathway, highlighting its pivotal role in ECM remodeling. Functional validation demonstrated that COL1A2 knockdown significantly suppressed bladder cancer cell invasion and migration, while COL1A2 overexpression conversely enhanced invasive phenotypes. These findings align with established mechanisms whereby MMPs (e.g., MMP-2/MMP-9) promote tumor progression via ECM degradation (e.g., type IV collagen) (14, 41). By integrating bioinformatics predictions with experimental validation, we not only delineated COL1A2’s biological function in bladder cancer but also identified it as a potential driver of ECM dysregulation, providing a mechanistic foundation for further exploration.

To elucidate the molecular axis by which COL1A2 regulates ECM remodeling, we performed DGEs analysis on transcriptomic data from patients with high versus low COL1A2 expression. Subsequent KEGG pathway enrichment and GO functional analyses consistently identified focal adhesion as the most significantly enriched pathway. Experimental validation demonstrated that COL1A2 overexpression markedly enhanced FAK activity, while pharmacological inhibition of FAK with PF573228 partly abrogated COL1A2-mediated ECM degradation. These findings establish a mechanistically coherent pathway whereby COL1A2 governs ECM remodeling through FAK signaling activation, aligning with the well-documented role of focal adhesion in tumor invasion (42–44).

Our integrative single-cell transcriptomic analysis reveals a previously unappreciated spatial orchestration of the COL1A2–ECM–FAK axis within the bladder tumor microenvironment. Crucially, the compartmentalized expression of COL1A2 in matrix cancer-associated fibroblasts and PTK2/FAK activation in epithelial cells undergoing epithelial–mesenchymal transition suggests a paracrine signaling paradigm that transcends traditional cell-autonomous frameworks. This spatial segregation resolves the paradox posed by bulk transcriptomics—how COL1A2, often regarded solely as a structural ECM component, mechanistically drives FAK-mediated EMT (45). Ligand-receptor topology inferred by CellChat further identifies syndecan-mediated signaling, specifically the interactions between COL1A1/COL1A2 and receptors SDC4/SDC1, as the principal communication axis from matrix CAFs to Epithelial_EMT cells, positioning SDC4 as a privileged receptor for COL1A2-enriched ECM cues. Notably, SDC1, another syndecan family member, is implicated in ECM-mediated immune exclusion through its heparan sulfate chains, suggesting a bifurcated functional specialization: SDC4 coordinates FAK-dependent EMT via collagen engagement, whereas SDC1 reinforces the ECM barrier to impede T-cell infiltration (46). This spatial and functional segregation aligns with our observations of distinct CAF–epithelial crosstalk modes. Consistent with emerging evidence, syndecan-4 emerges as a critical mediator of ECM-integrin interplay, facilitating focal adhesion kinase activation and cytoskeletal remodeling that promote tumor invasiveness (47). The co-option of SDC4 for mechanotransduction and SDC1 for immune evasion underscores their contextual specialization within the tumor microenvironment. Importantly, the unique role of SDC4—a heparan sulfate proteoglycan—as a gatekeeper of CAF-driven malignancy reveals its potential cooperation with integrins such as α2β1 (47, 48), amplifying pro-invasive mechanotransduction and offering a focused therapeutic target to disrupt stromal-epithelial signaling that underpins bladder cancer progression.

This study reveals that COL1A2 drives the transition from non-muscle-invasive to muscle-invasive bladder cancer via a spatially resolved ECM-FAK signaling axis, delineated through single-cell transcriptomics. Distinct from previous bioinformatic predictions identifying progression-associated genes, we establish COL1A2 as an upstream ECM orchestrator that facilitates FAK-dependent invasion by matrix CAF-derived ECM scaffolds engaging SDC4/SDC1 receptors on Epithelial_EMT cell. Notably, COL1A2 upregulation during malignant transformation correlates with poorer prognosis, underscoring its potential both as an early detection biomarker and a therapeutic target. This spatially defined signaling circuit moves beyond correlative molecular insights to mechanistically elucidate how stromal–epithelial crosstalk underpins the transition between non-muscle-invasive and muscle-invasive bladder cancer.

While this study delineates the COL1A2–ECM–FAK regulatory axis, several key limitations remain. First, the direct molecular link connecting COL1A2-enriched ECM scaffolds to FAK activation is not fully resolved; it remains unclear whether signaling is initiated via direct FAK recruitment through integrin clustering or mediated indirectly through transcriptional regulation. Addressing this will require mechanistic studies employing FRET-based tension sensors and promoter-reporter assays. Second, the observed biphasic pattern of COL1A2 expression—repressed in NMIBC and reactivated in MIBC—likely reflects stage-specific modulation by TGF-β signaling (49–51), but the precise spatiotemporal regulators driving this switch await validation through longitudinal single-cell analyses of matched patient samples. Lastly, potential crosstalk with EMT or Wnt/β-catenin pathways could enable compensatory invasion mechanisms; dissecting these will benefit from conditional COL1A2 knockout in matrix CAFs combined with phosphoproteomic profiling to identify escape routes.

Collectively, our findings define a spatially orchestrated stromal–epithelial signaling axis within the tumour microenvironment that drives bladder cancer invasion. Central to this axis, matrix cancer-associated fibroblasts secrete COL1A2 to mediate pathogenic ECM remodeling. This remodeling, in turn, activates syndecan-4/syndecan-1 (SDC4/SDC1)-dependent FAK signaling within epithelial cells undergoing epithelial–mesenchymal transition, thereby accelerating the transition from NMIBC to MIBC. Beyond elucidating a key mechanistic framework, this pathway nominates COL1A2 and its downstream effectors as multifunctional biomarkers capable of refining patient risk stratification, enabling non-invasive disease monitoring, and providing early recurrence alerts. Critically, given the invasive nature and sampling bias of current cystoscopic surveillance, leveraging COL1A2 detection via liquid biopsy or targeted molecular imaging offers a transformative approach for precision staging. While tumour heterogeneity and therapeutic resistance persist as translational hurdles, the spatially resolved architecture and druggable nodes of this axis deliver an actionable blueprint for developing mechanistically grounded therapies.

## 5 Conclusions

In this study, we integrated multi-omics and single-cell transcriptomic analyses to elucidate the role of tumor microenvironment components in bladder cancer. We identified a COL1A2-driven ECM remodeling and focal adhesion signaling axis that is closely associated with the invasive phenotype in MIBC. This COL1A2–ECM–FAK cascade mediates crosstalk between matrix cancer-associated fibroblasts and epithelial cells undergoing EMT, thereby facilitating tumor invasiveness. Our findings highlight the central function of COL1A2 in shaping the extracellular matrix and activating integrin-FAK signaling to promote malignant cell behaviors. Importantly, these results provide novel insights into the mechanistic underpinnings of bladder cancer invasiveness and position COL1A2 as a promising biomarker and therapeutic target. Targeting the COL1A2-mediated ECM remodeling pathway may offer new opportunities to disrupt tumor-stroma interactions and restrain bladder cancer aggressiveness.

## Data Availability

The original contributions presented in the study are included in the article/**Supplementary Material**. Further inquiries can be directed to the corresponding authors.
