# Supplementary figures and images for: Integrative bulk and single-cell transcriptomic analysis reveals COL1A2-driven ECM remodeling and focal adhesion signaling associated with the transition from non-muscle-invasive to muscle-invasive bladder cancer (part 1 of 2)
Source: Front Oncol. 2026 Jan 5;15:1716324. doi: 10.3389/fonc.2025.1716324 (PMC12812713; doi:10.3389/fonc.2025.1716324)

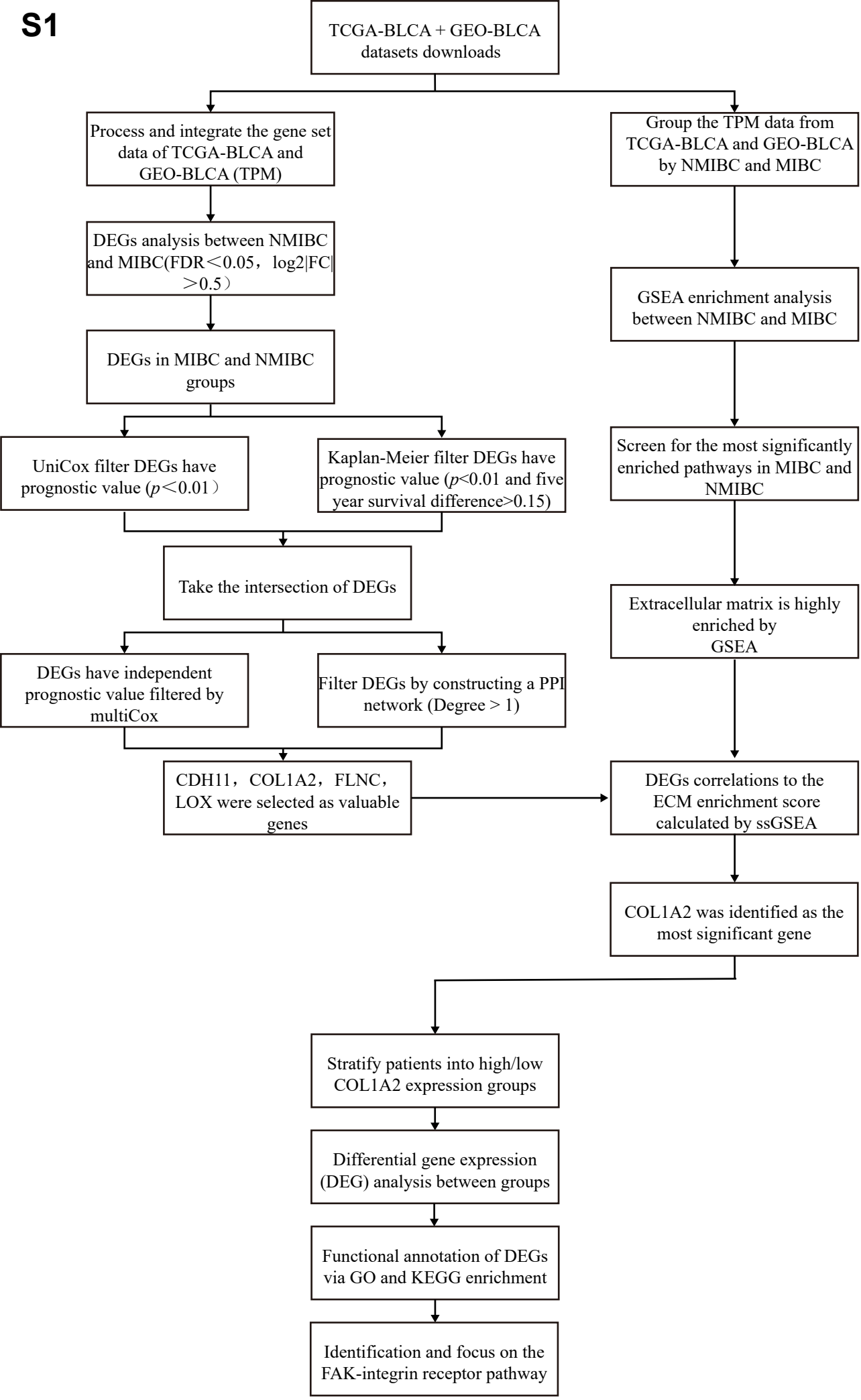

Supplement: Supplementary file 1 [file DataSheet1.pdf]

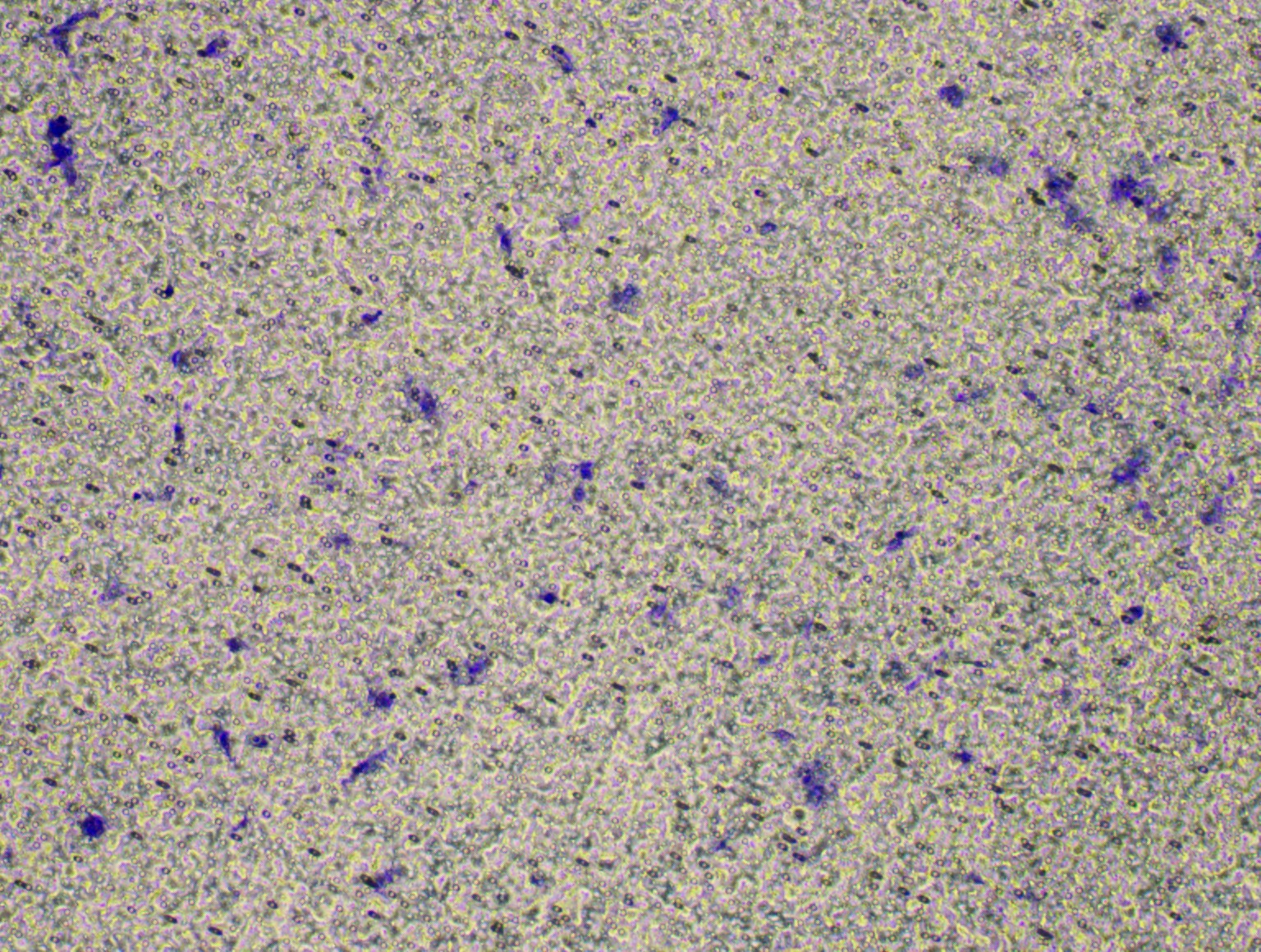

Supplement: Supplementary file 3 [file DataSheet3.zip › transwell-oe-COL1A2/1-1.jpg]

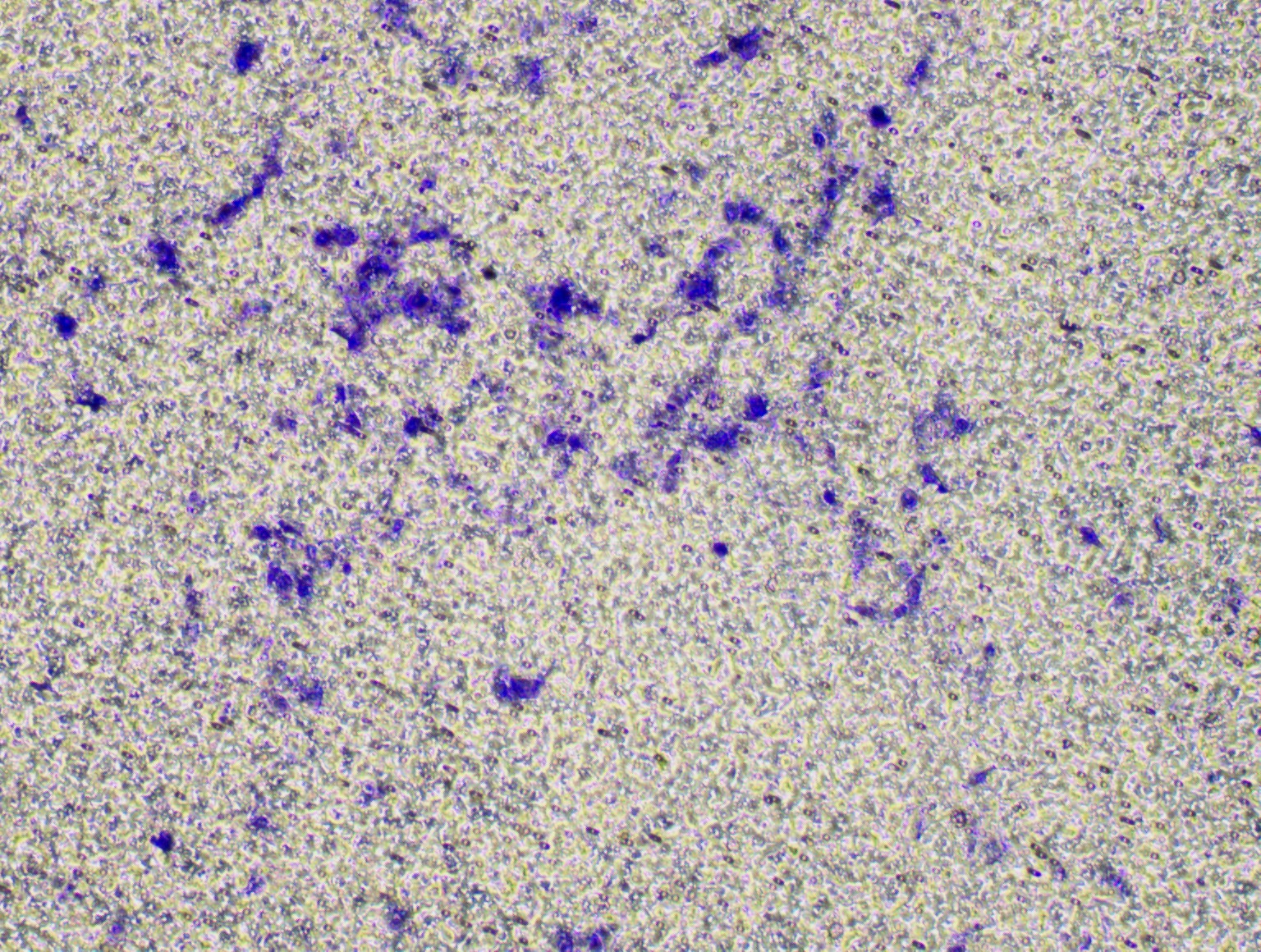

Supplement: Supplementary file 3 [file DataSheet3.zip › transwell-oe-COL1A2/1-2.jpg]

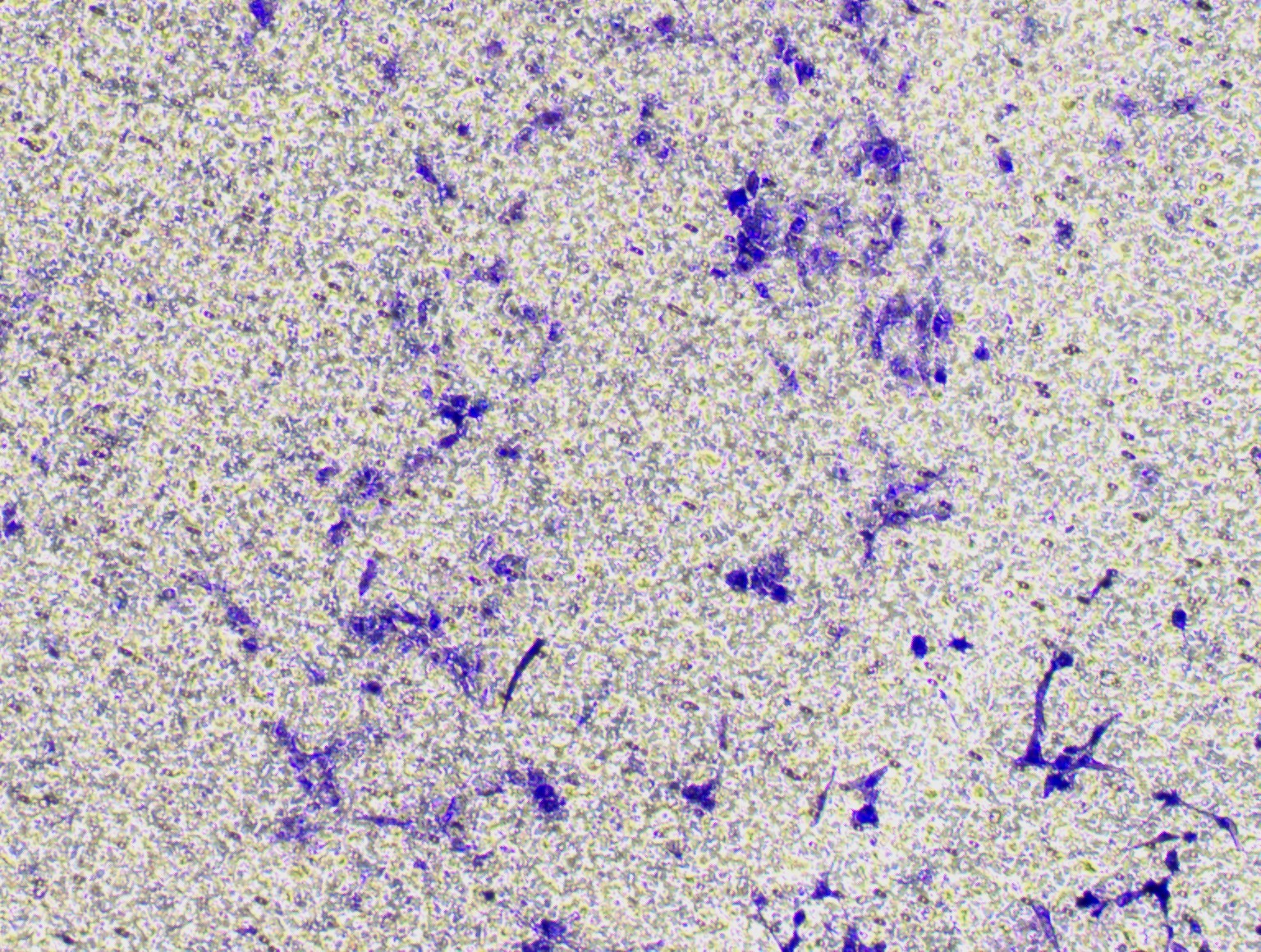

Supplement: Supplementary file 3 [file DataSheet3.zip › transwell-oe-COL1A2/1-3.jpg]

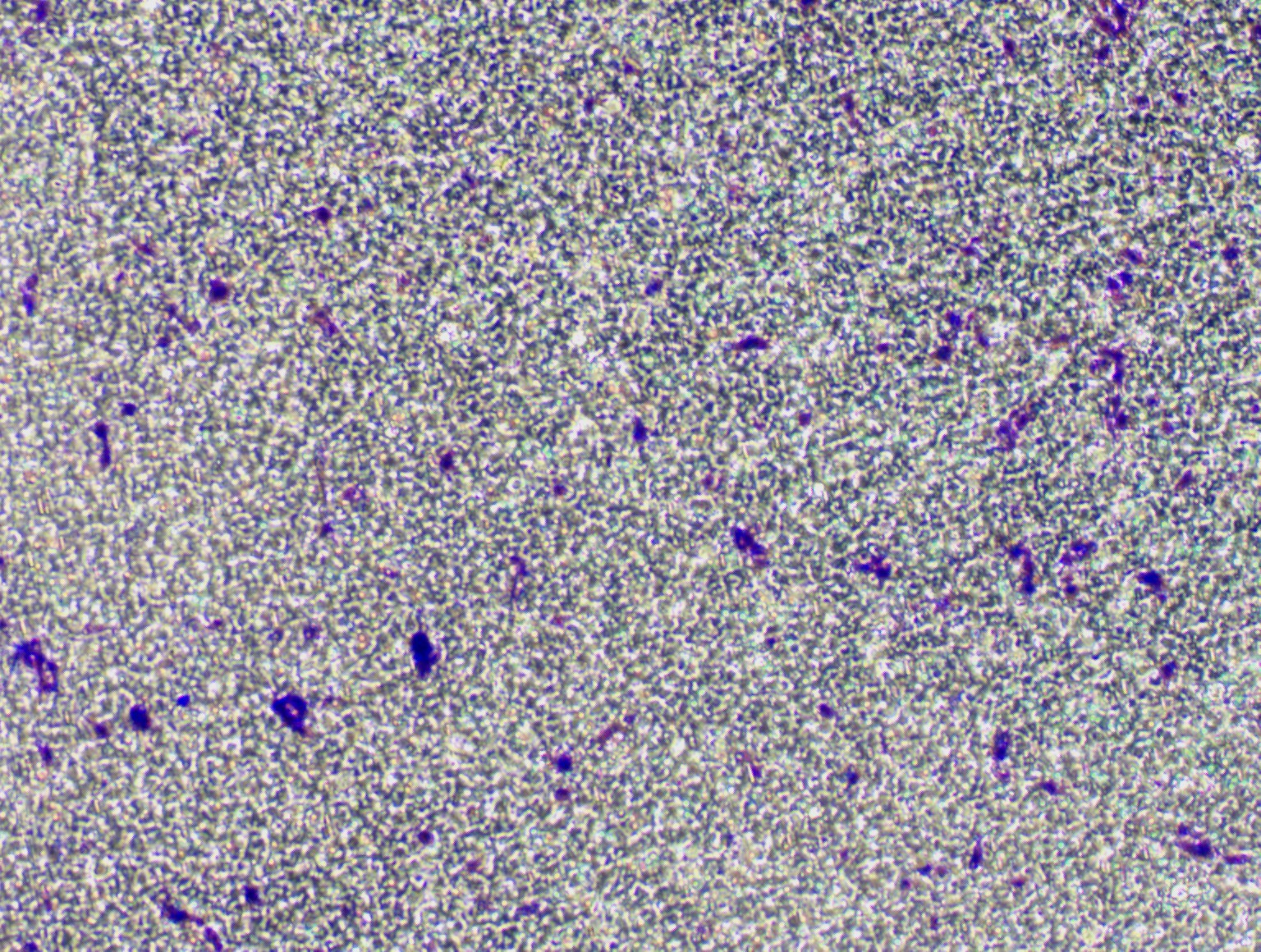

Supplement: Supplementary file 3 [file DataSheet3.zip › transwell-oe-COL1A2/1-4.jpg]

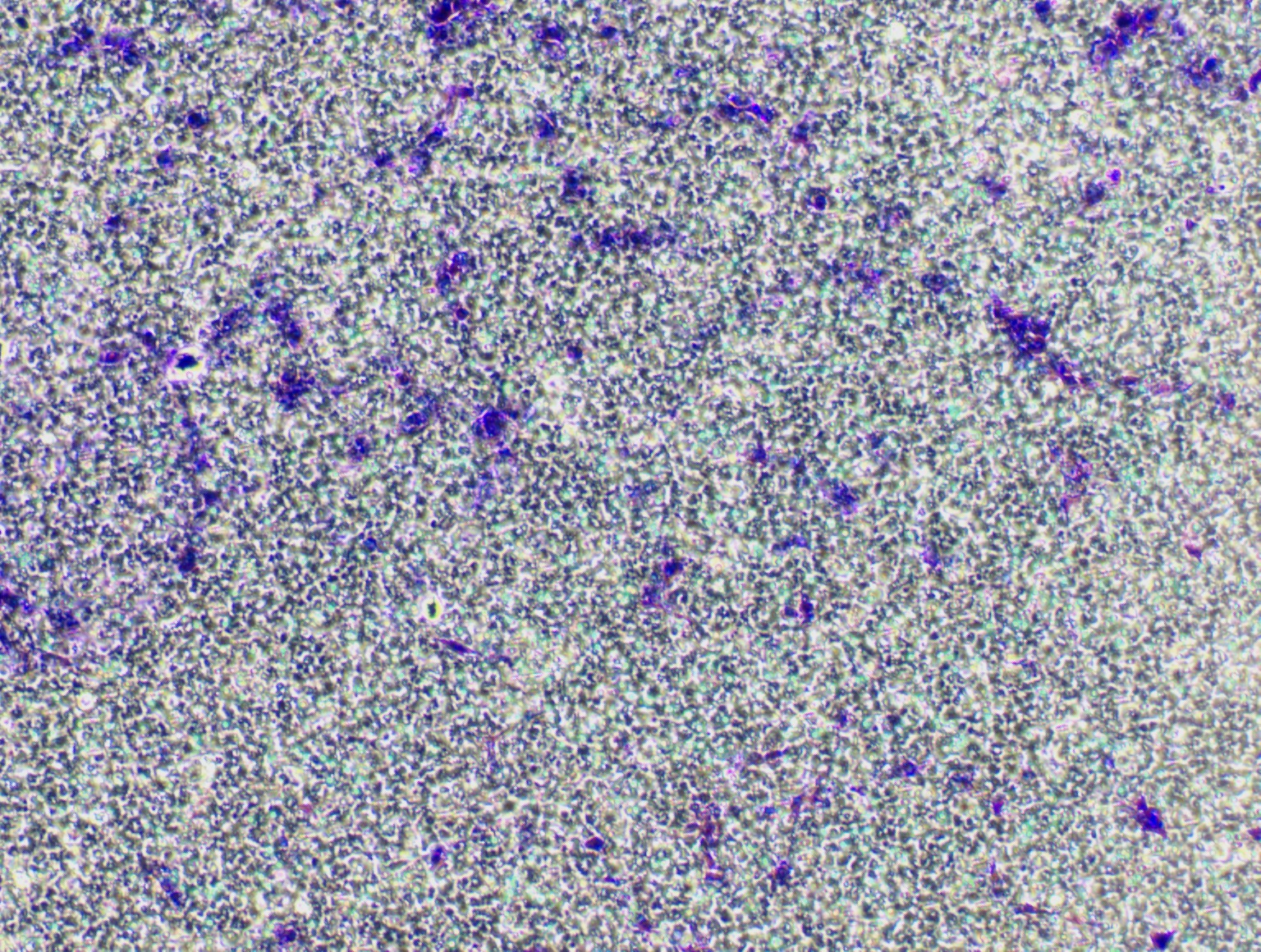

Supplement: Supplementary file 3 [file DataSheet3.zip › transwell-oe-COL1A2/2-1.jpg]

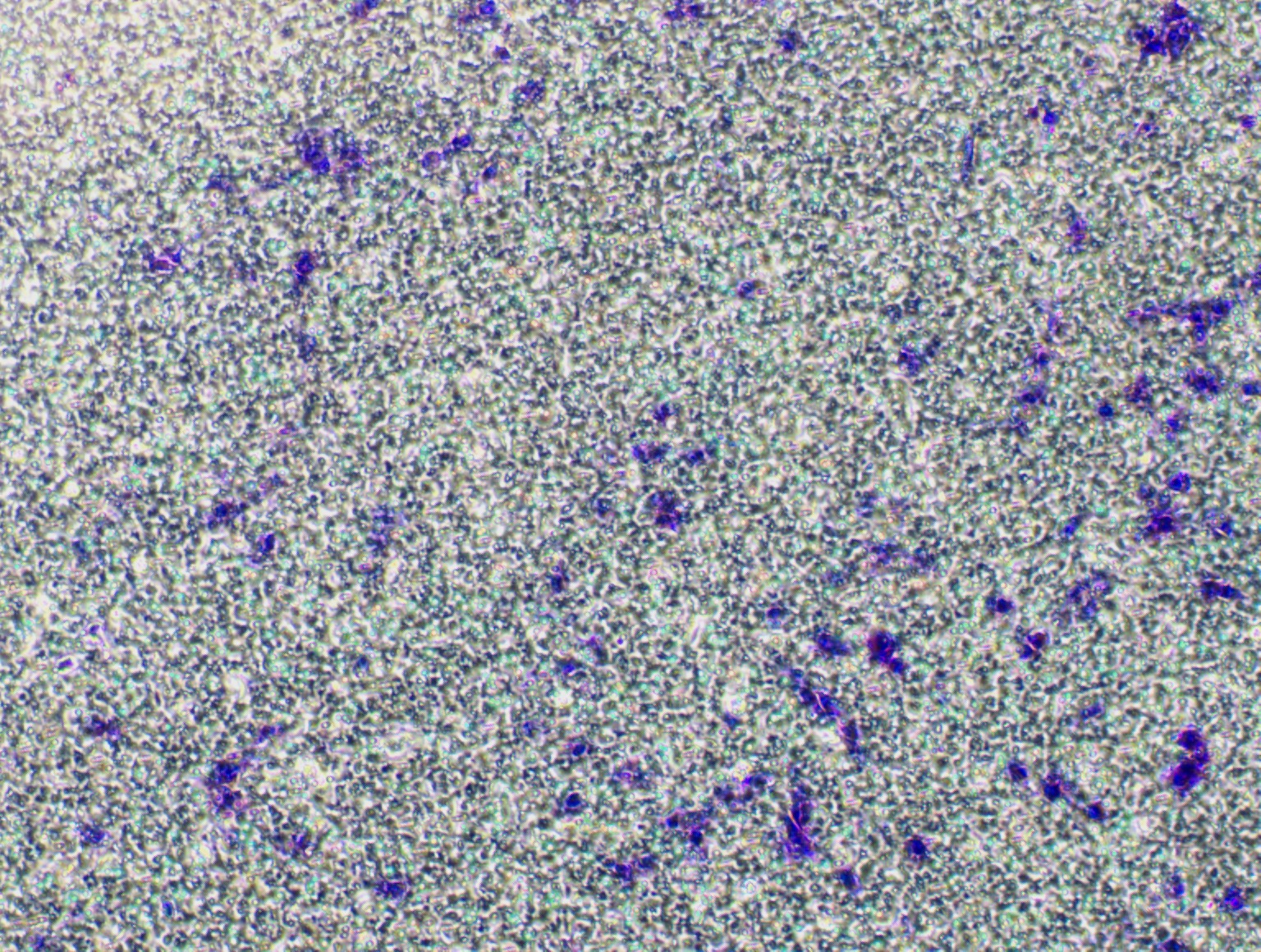

Supplement: Supplementary file 3 [file DataSheet3.zip › transwell-oe-COL1A2/2-2.jpg]

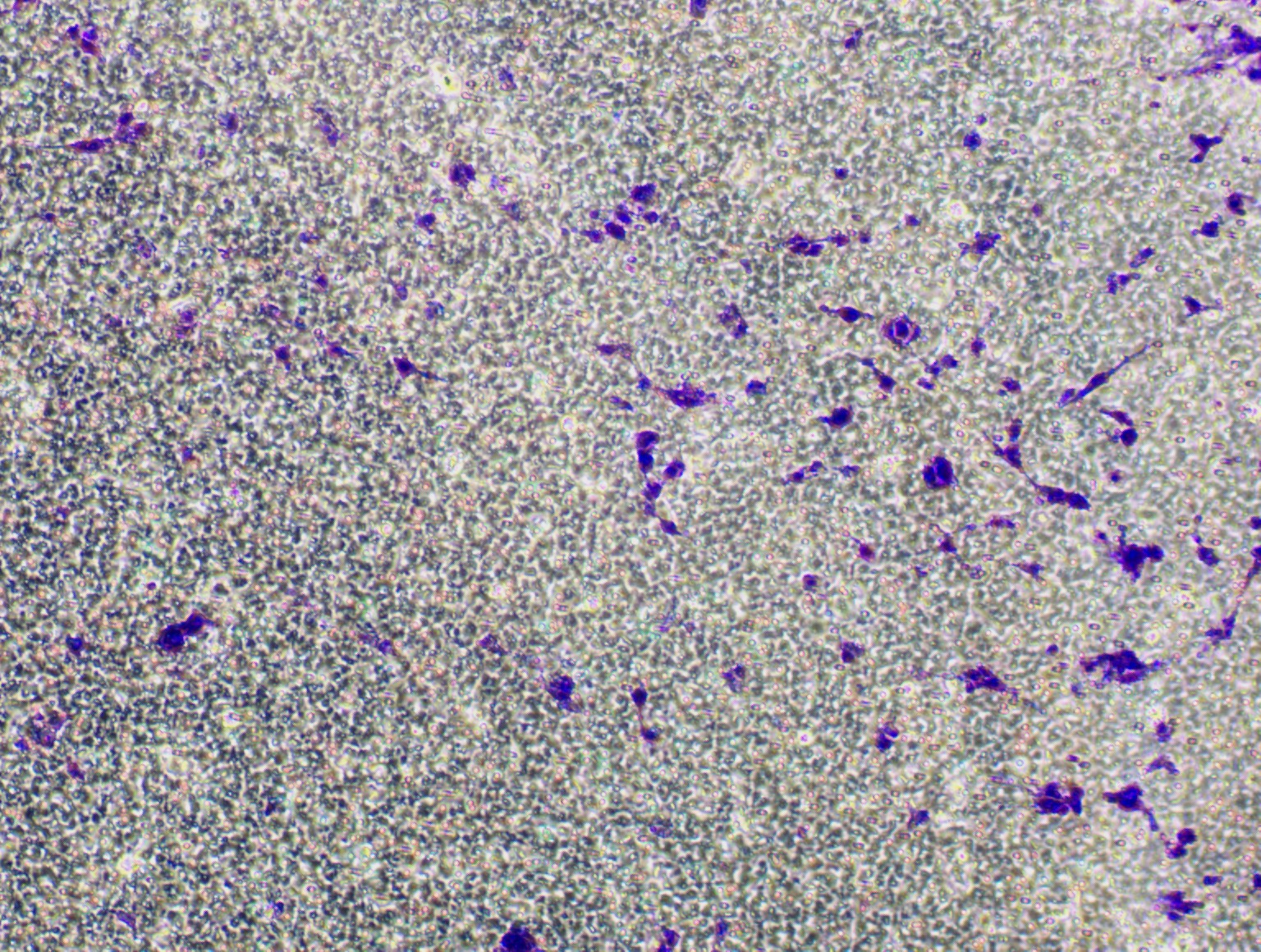

Supplement: Supplementary file 3 [file DataSheet3.zip › transwell-oe-COL1A2/2-3.jpg]

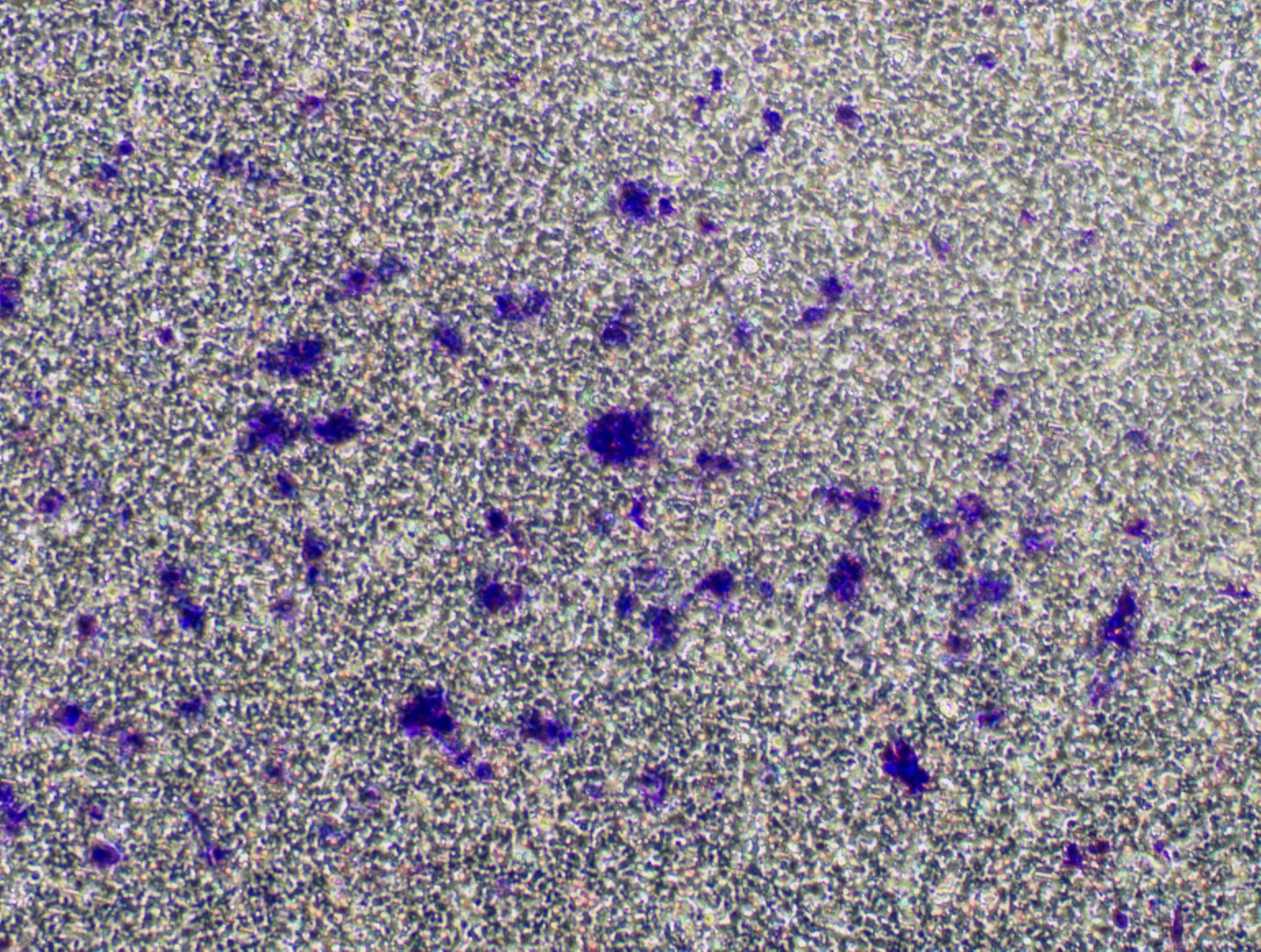

Supplement: Supplementary file 3 [file DataSheet3.zip › transwell-oe-COL1A2/2-4.jpg]

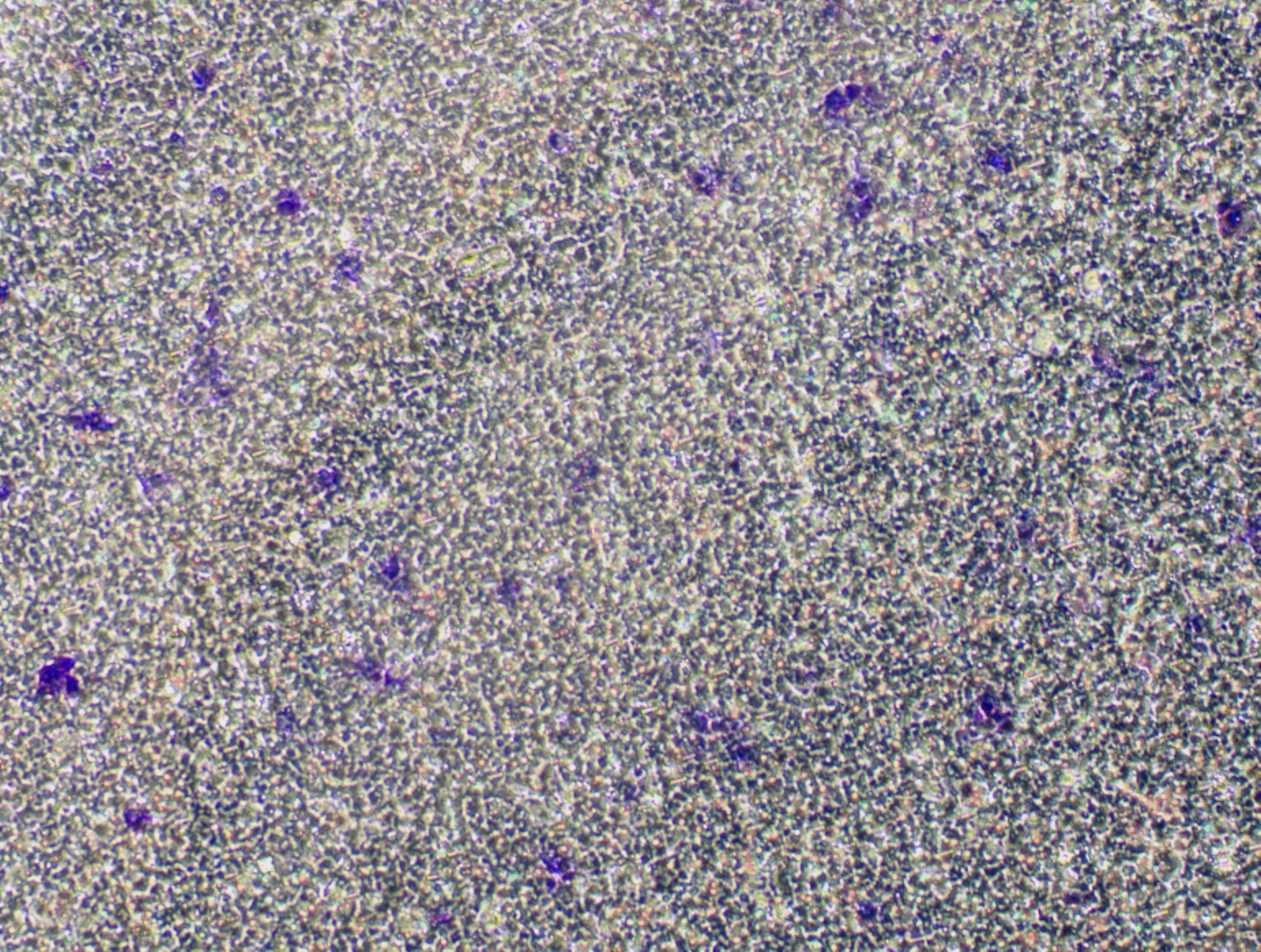

Supplement: Supplementary file 3 [file DataSheet3.zip › transwell-oe-COL1A2/3-1.jpg]

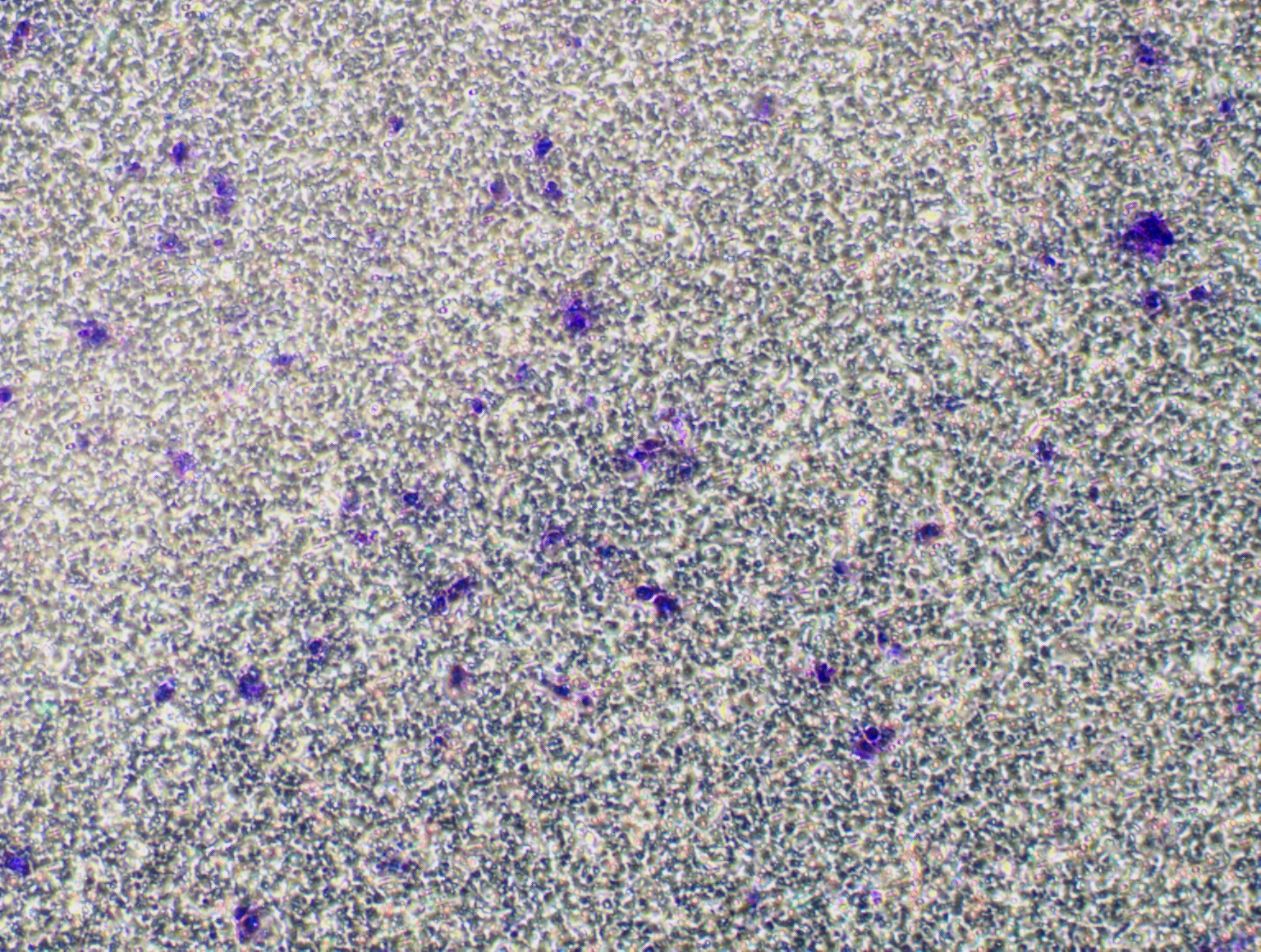

Supplement: Supplementary file 3 [file DataSheet3.zip › transwell-oe-COL1A2/3-2.jpg]

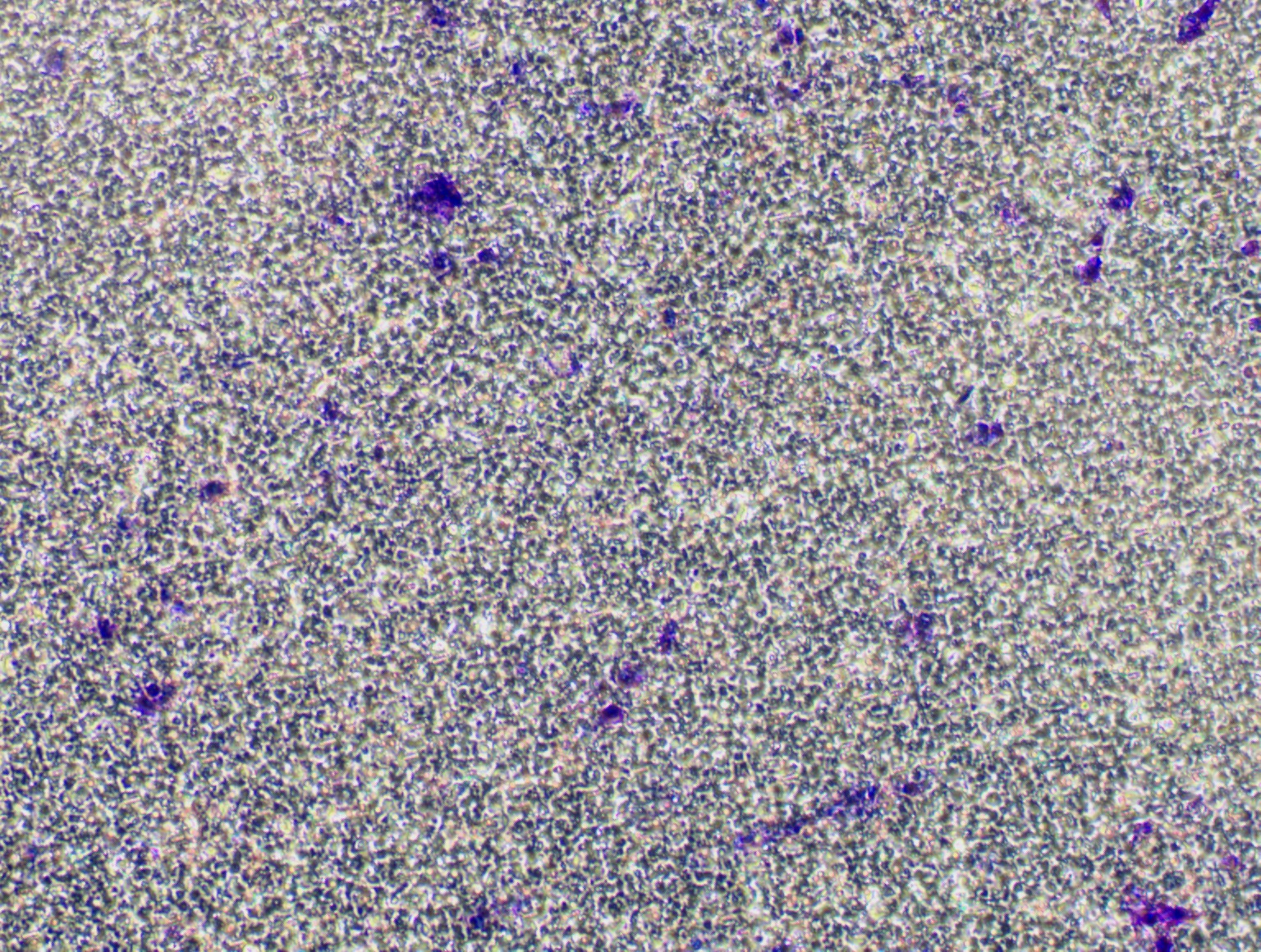

Supplement: Supplementary file 3 [file DataSheet3.zip › transwell-oe-COL1A2/3-3.jpg]

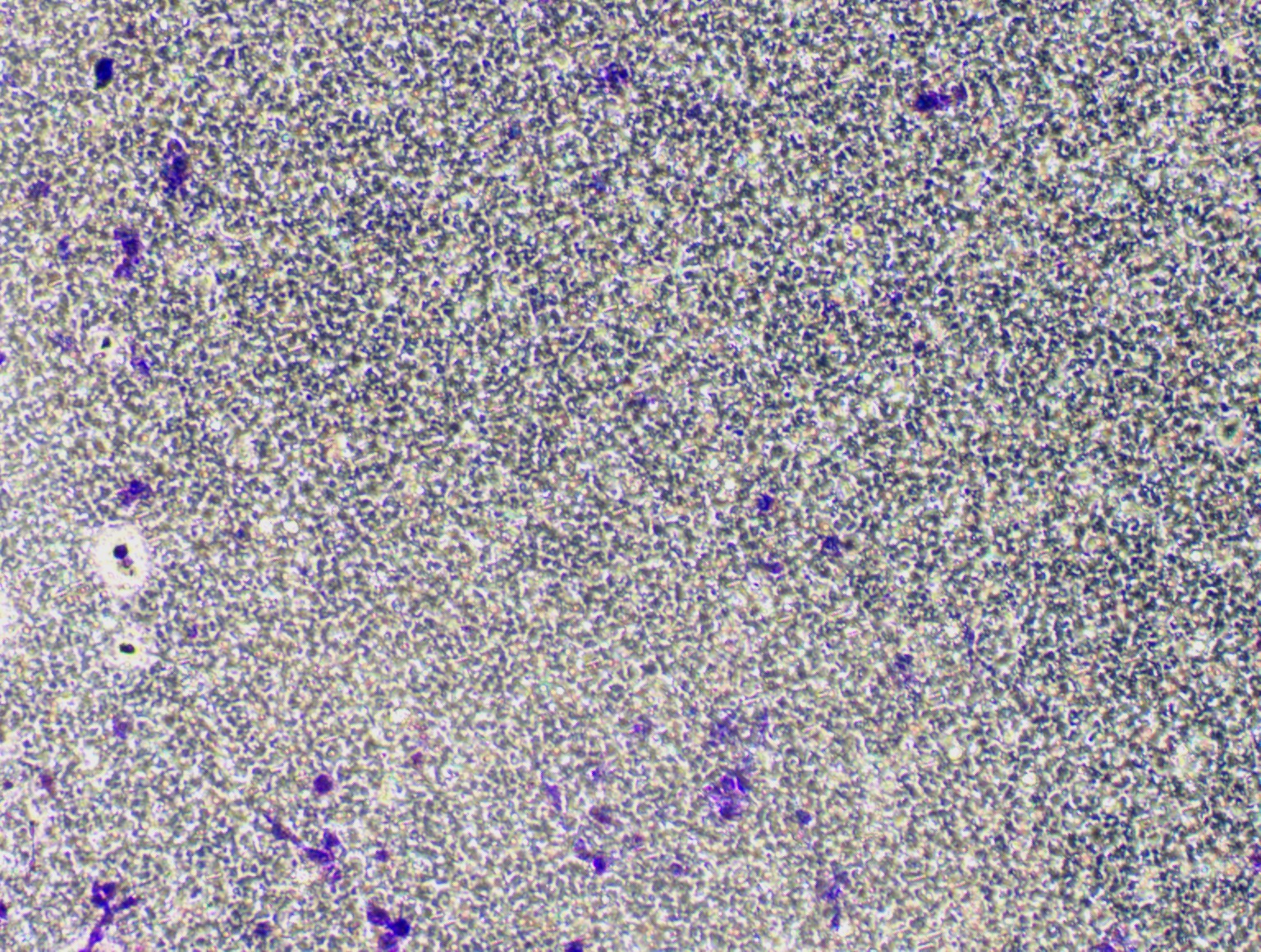

Supplement: Supplementary file 3 [file DataSheet3.zip › transwell-oe-COL1A2/3-4.jpg]

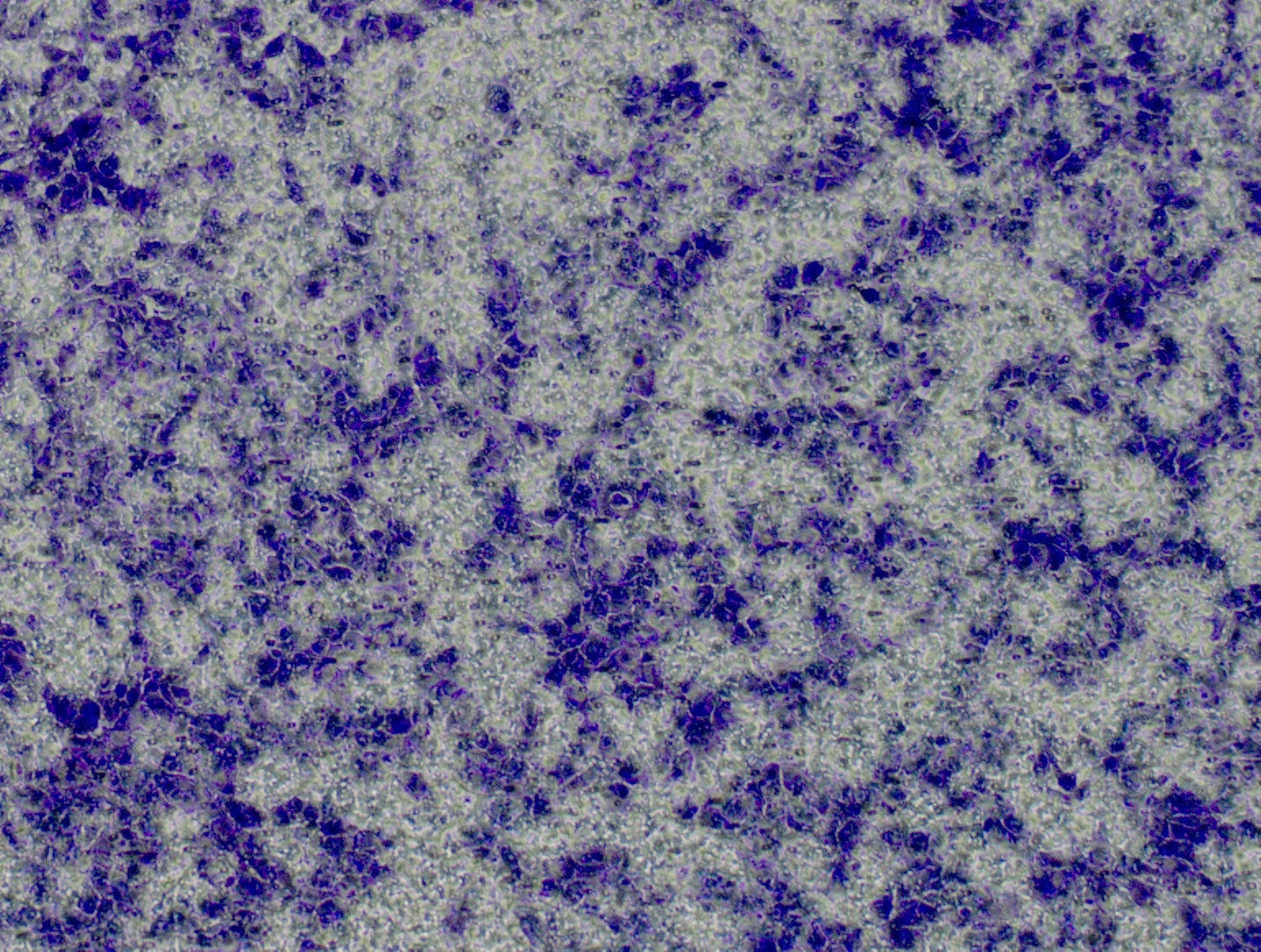

Supplement: Supplementary file 3 [file DataSheet3.zip › transwell-oe-COL1A2/4-1.jpg]

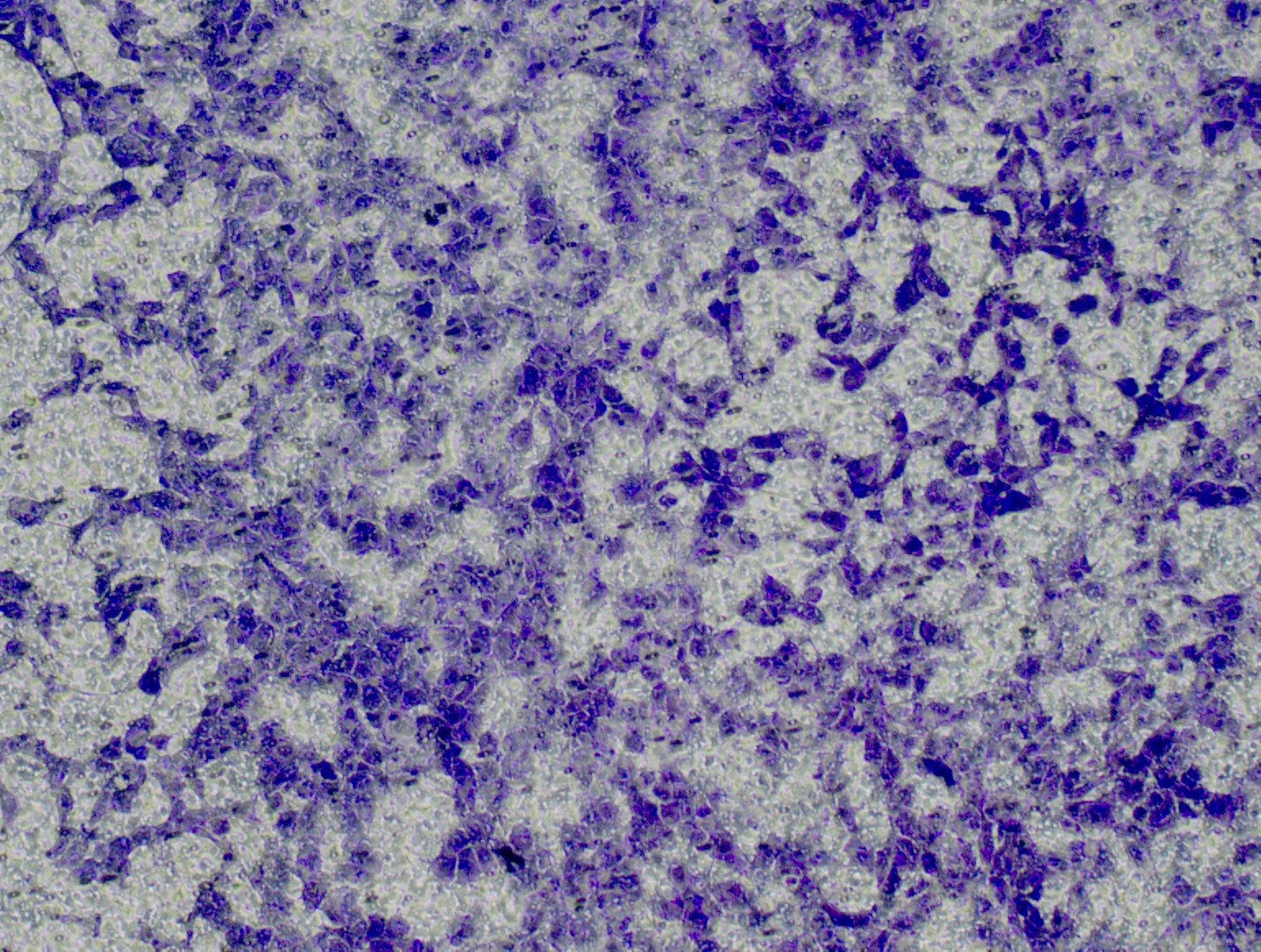

Supplement: Supplementary file 3 [file DataSheet3.zip › transwell-oe-COL1A2/4-2.jpg]

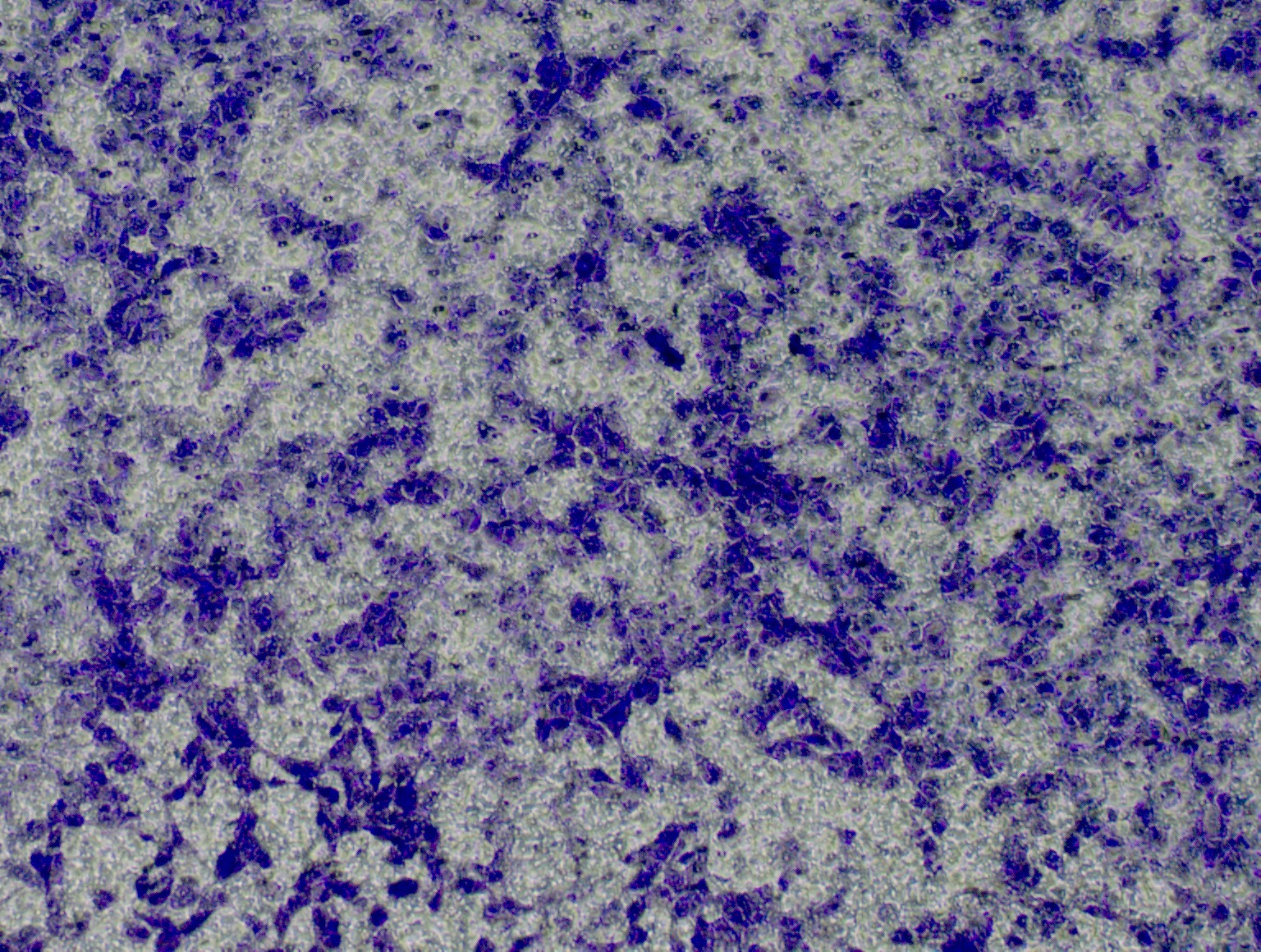

Supplement: Supplementary file 3 [file DataSheet3.zip › transwell-oe-COL1A2/4-3.jpg]

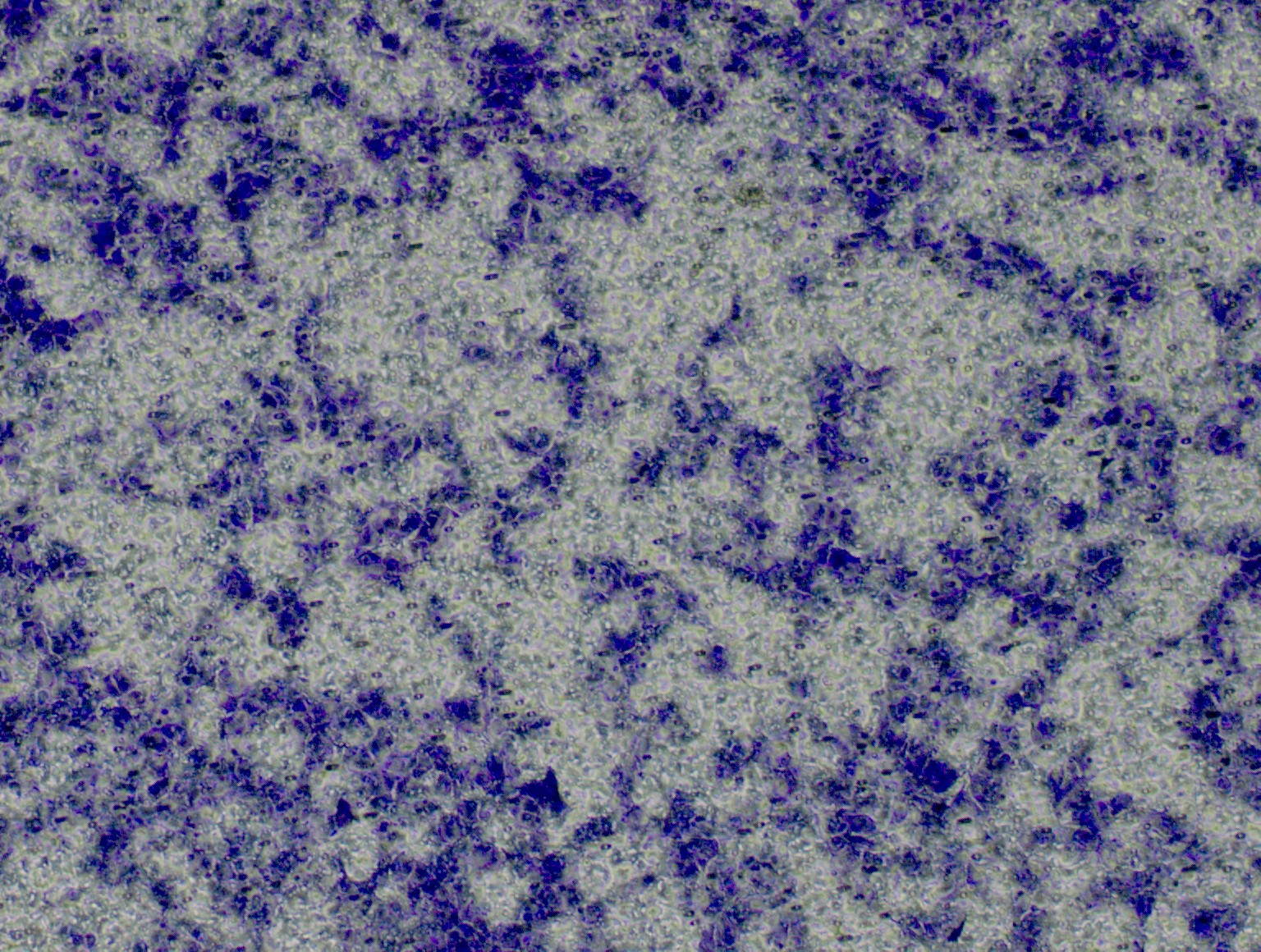

Supplement: Supplementary file 3 [file DataSheet3.zip › transwell-oe-COL1A2/4-4.jpg]

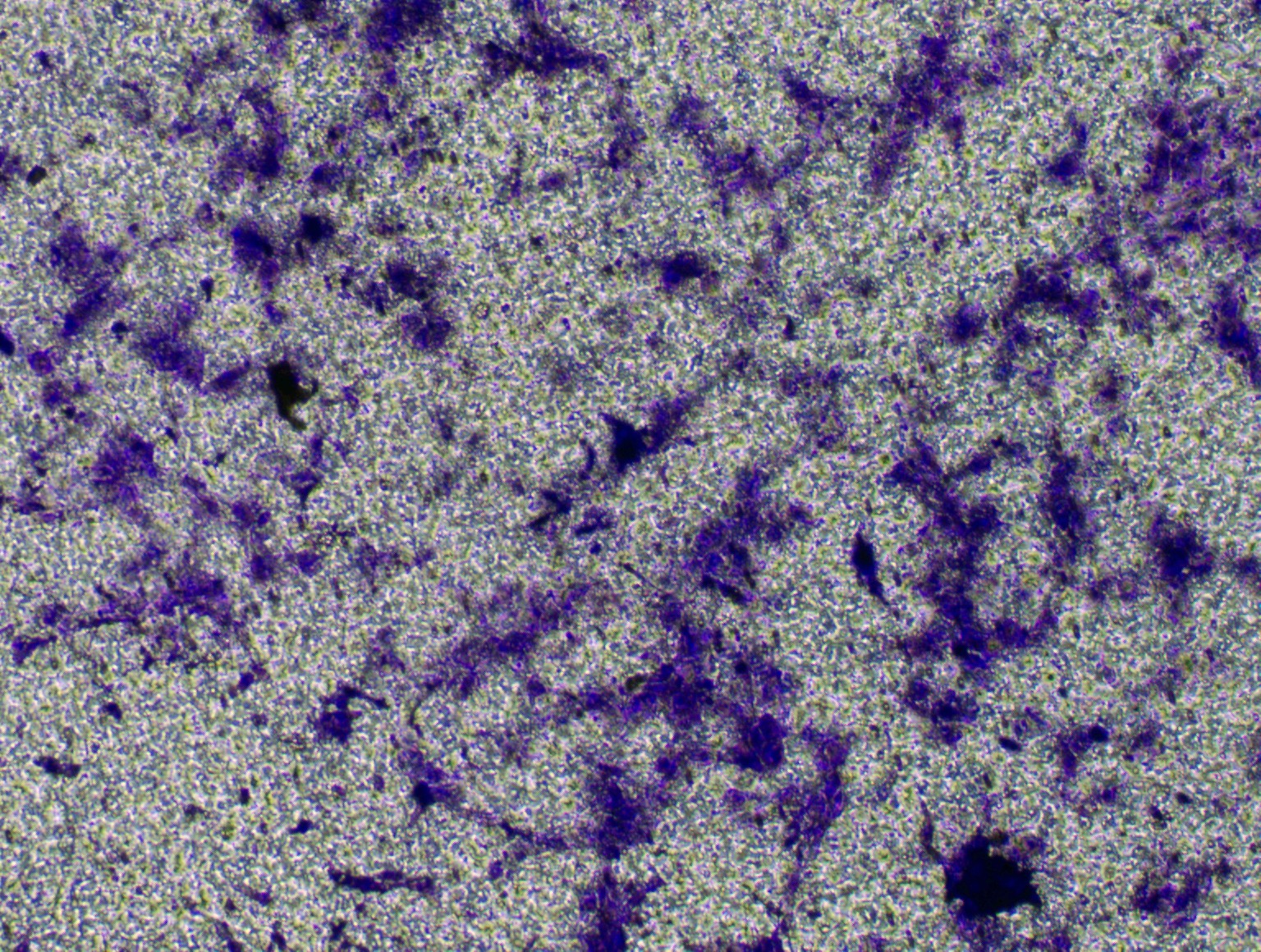

Supplement: Supplementary file 3 [file DataSheet3.zip › transwell-oe-COL1A2/5-1.jpg]

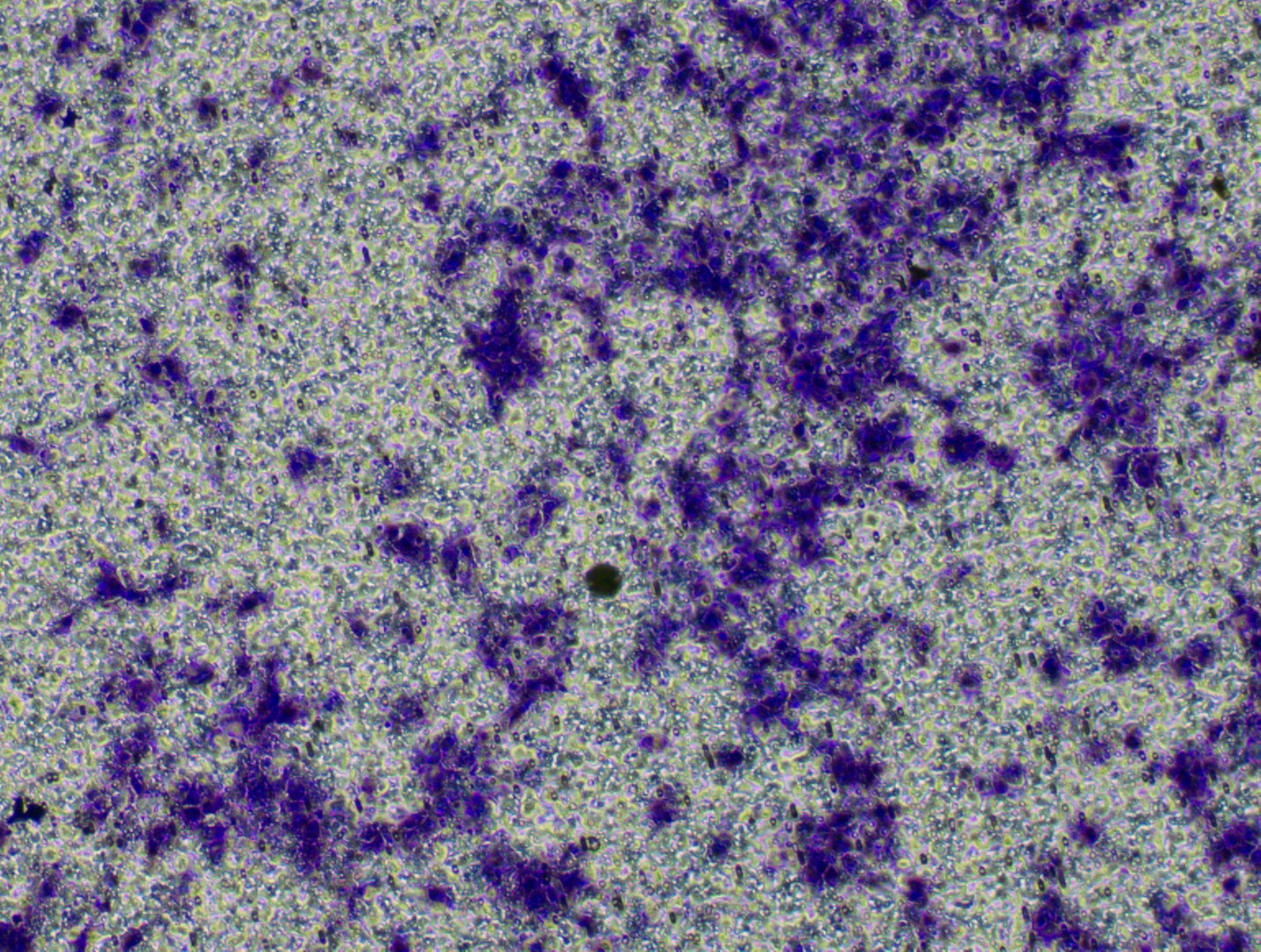

Supplement: Supplementary file 3 [file DataSheet3.zip › transwell-oe-COL1A2/5-2.jpg]

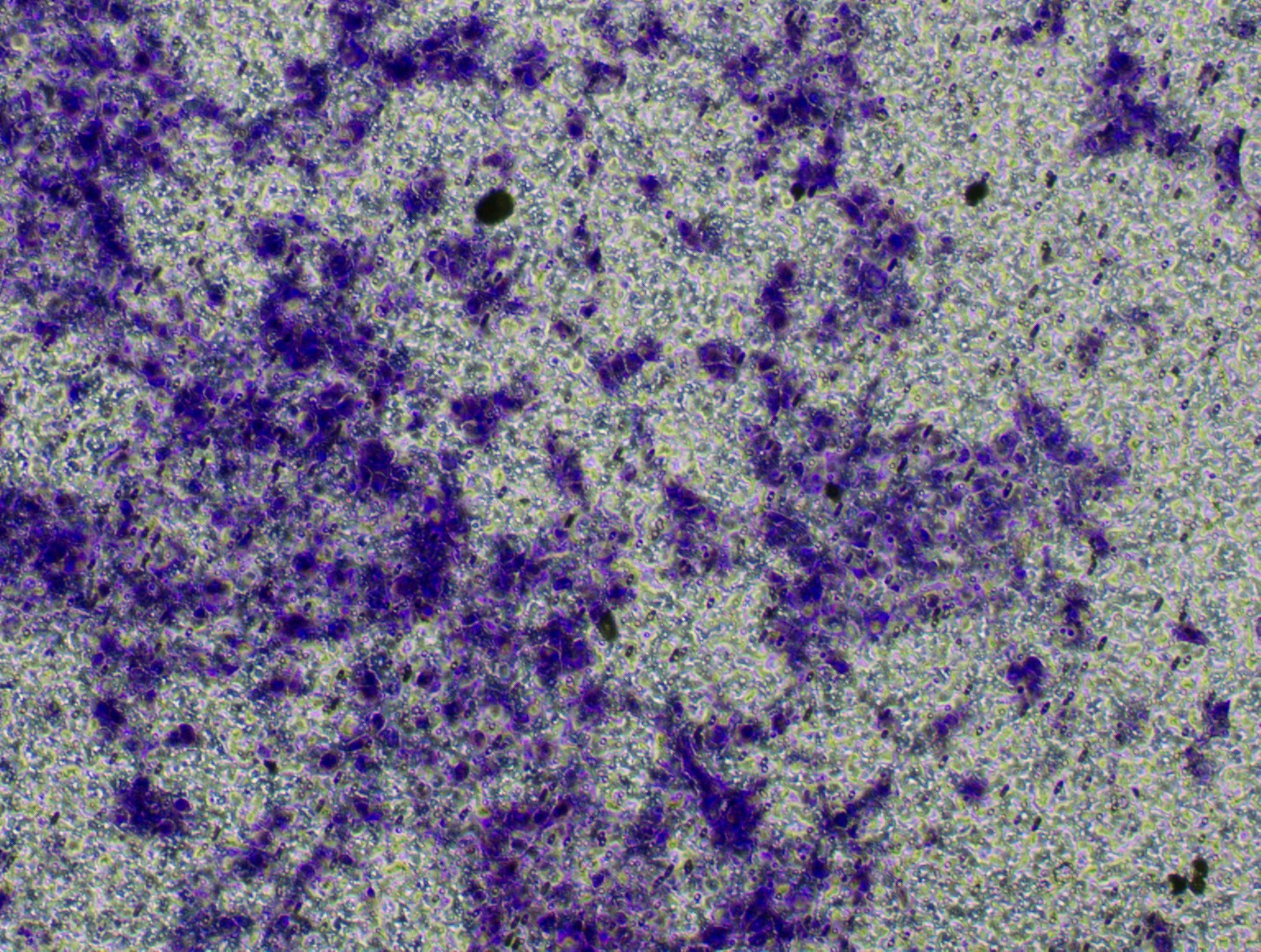

Supplement: Supplementary file 3 [file DataSheet3.zip › transwell-oe-COL1A2/5-3.jpg]

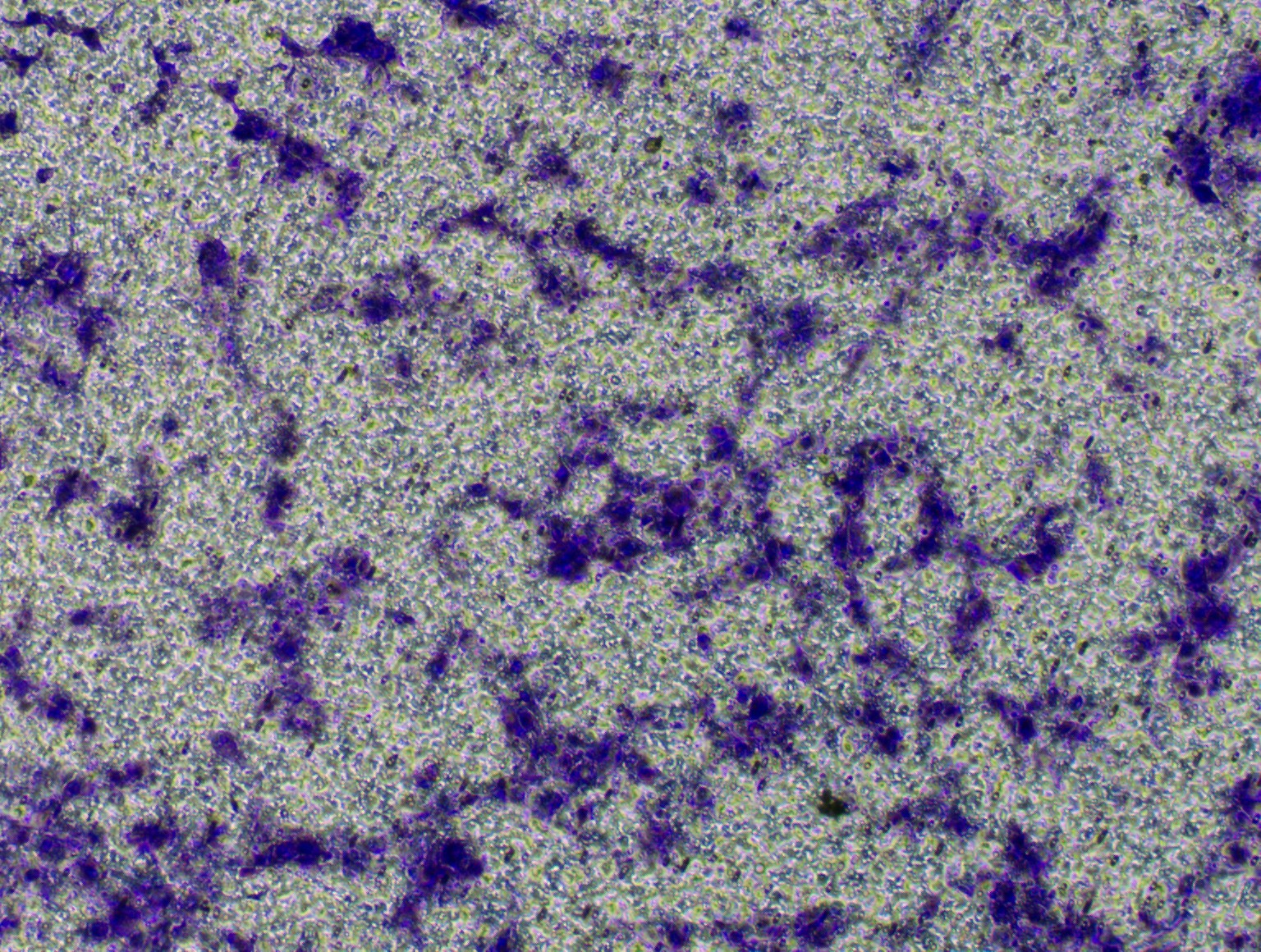

Supplement: Supplementary file 3 [file DataSheet3.zip › transwell-oe-COL1A2/5-4.jpg]

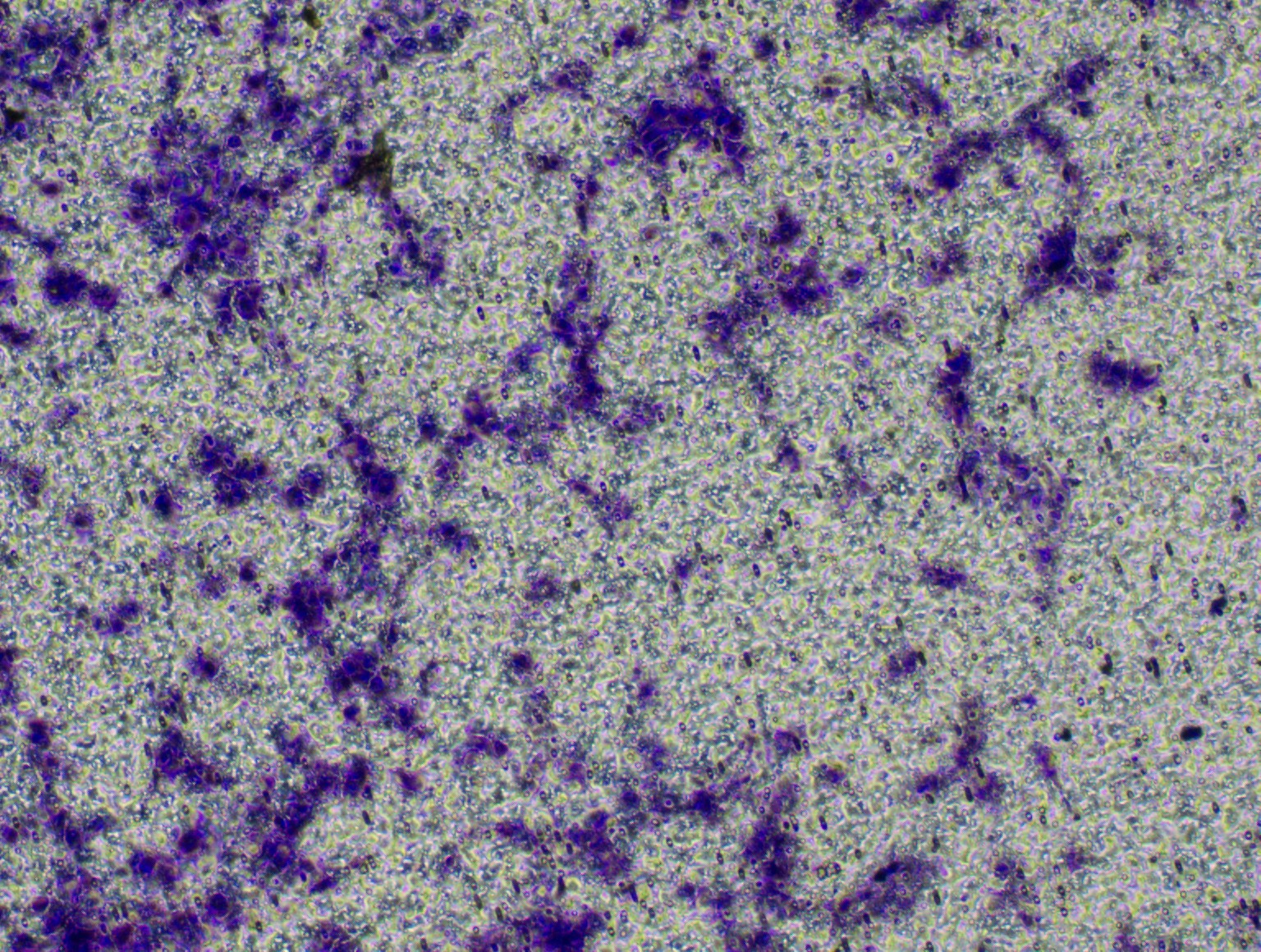

Supplement: Supplementary file 3 [file DataSheet3.zip › transwell-oe-COL1A2/6-1.jpg]

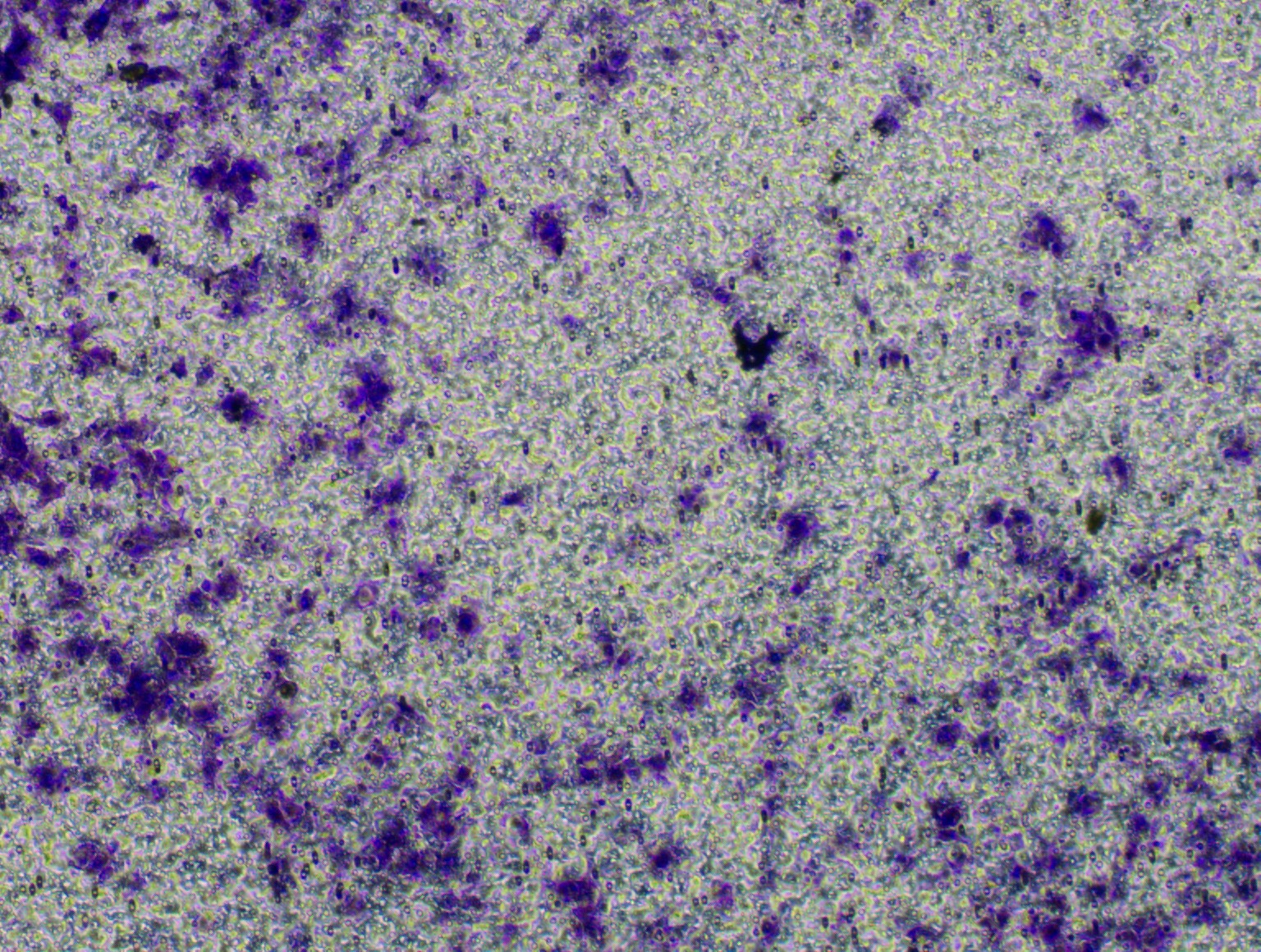

Supplement: Supplementary file 3 [file DataSheet3.zip › transwell-oe-COL1A2/6-2.jpg]

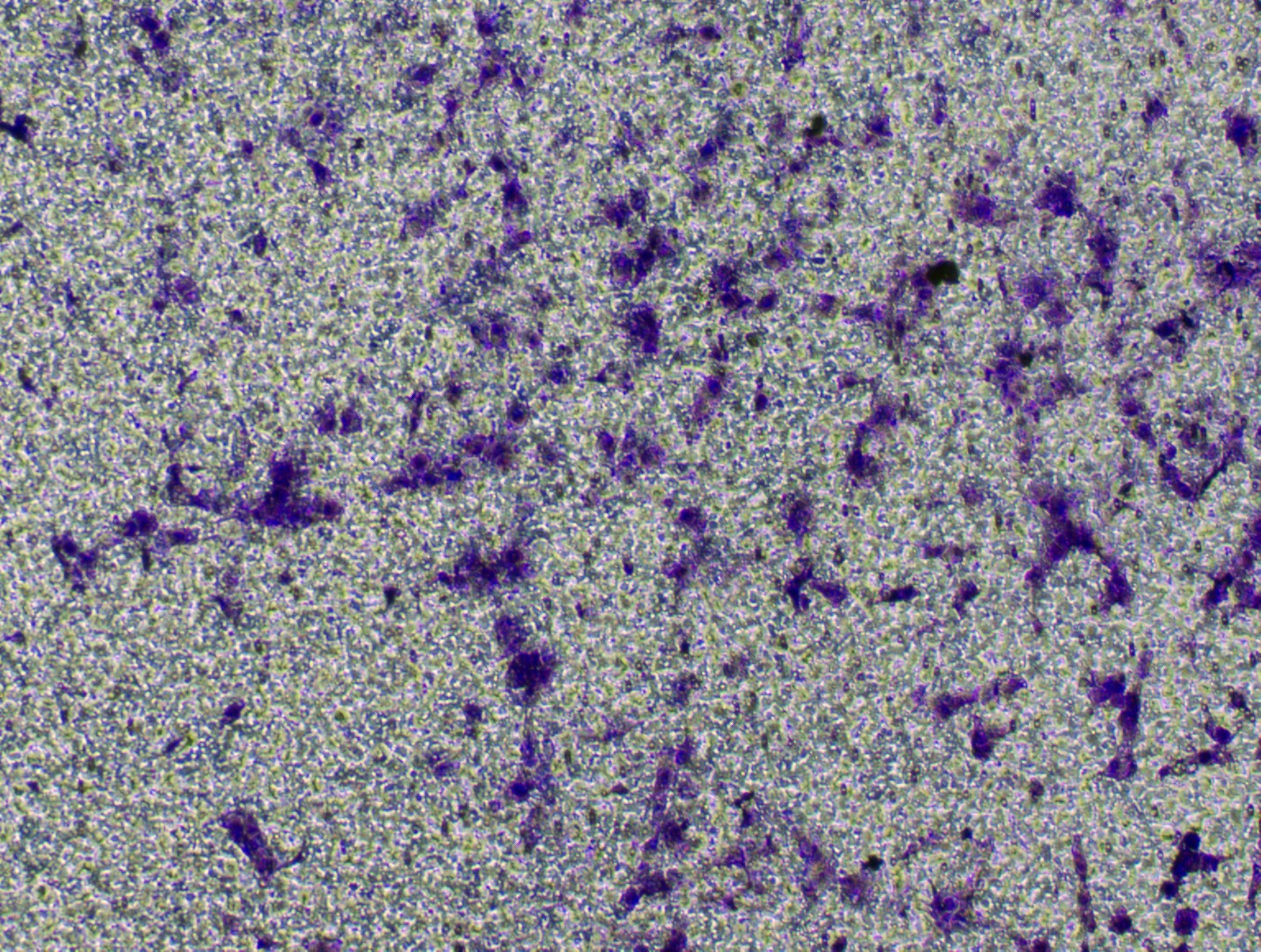

Supplement: Supplementary file 3 [file DataSheet3.zip › transwell-oe-COL1A2/6-3.jpg]

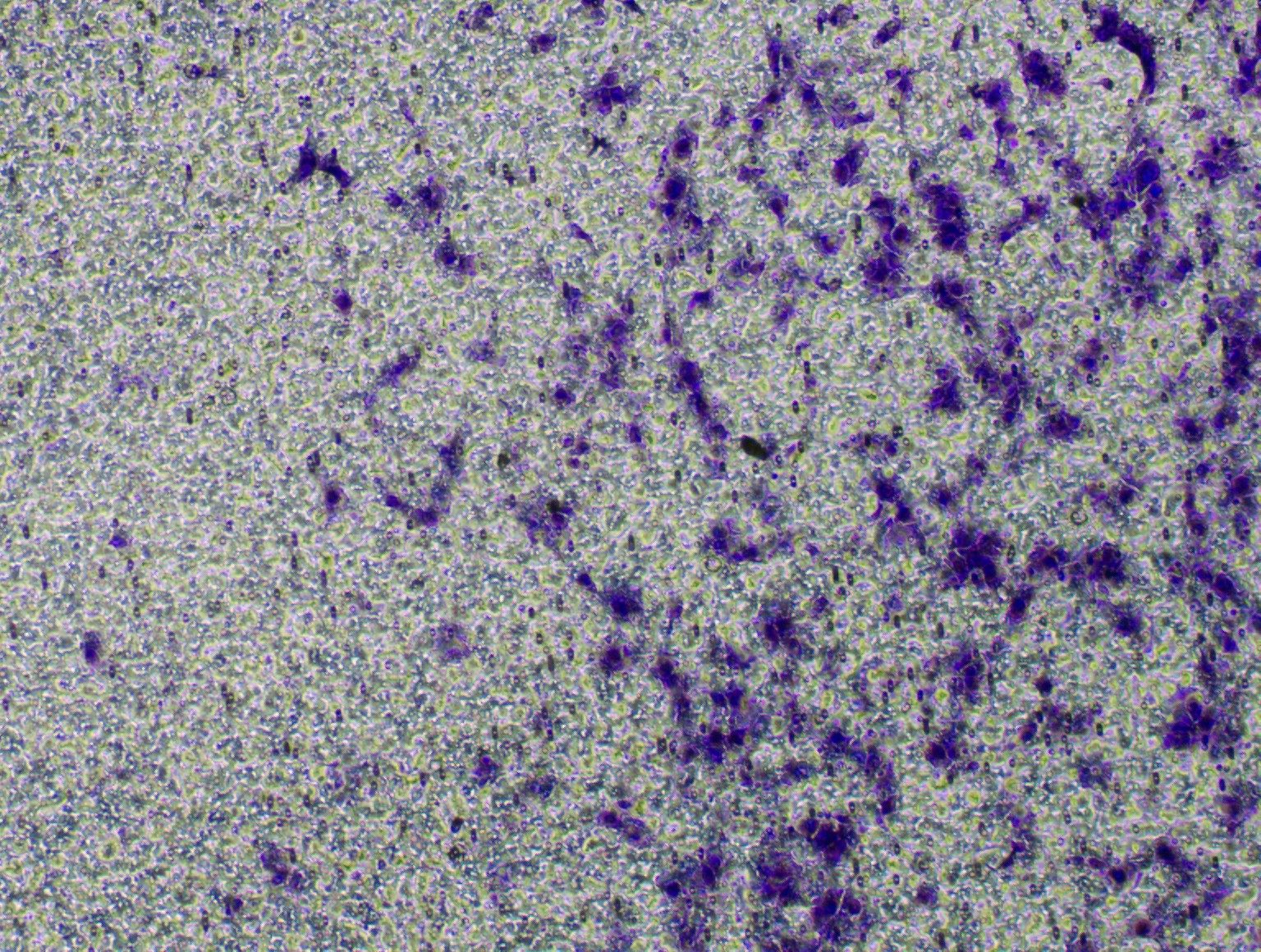

Supplement: Supplementary file 3 [file DataSheet3.zip › transwell-oe-COL1A2/6-4.jpg]

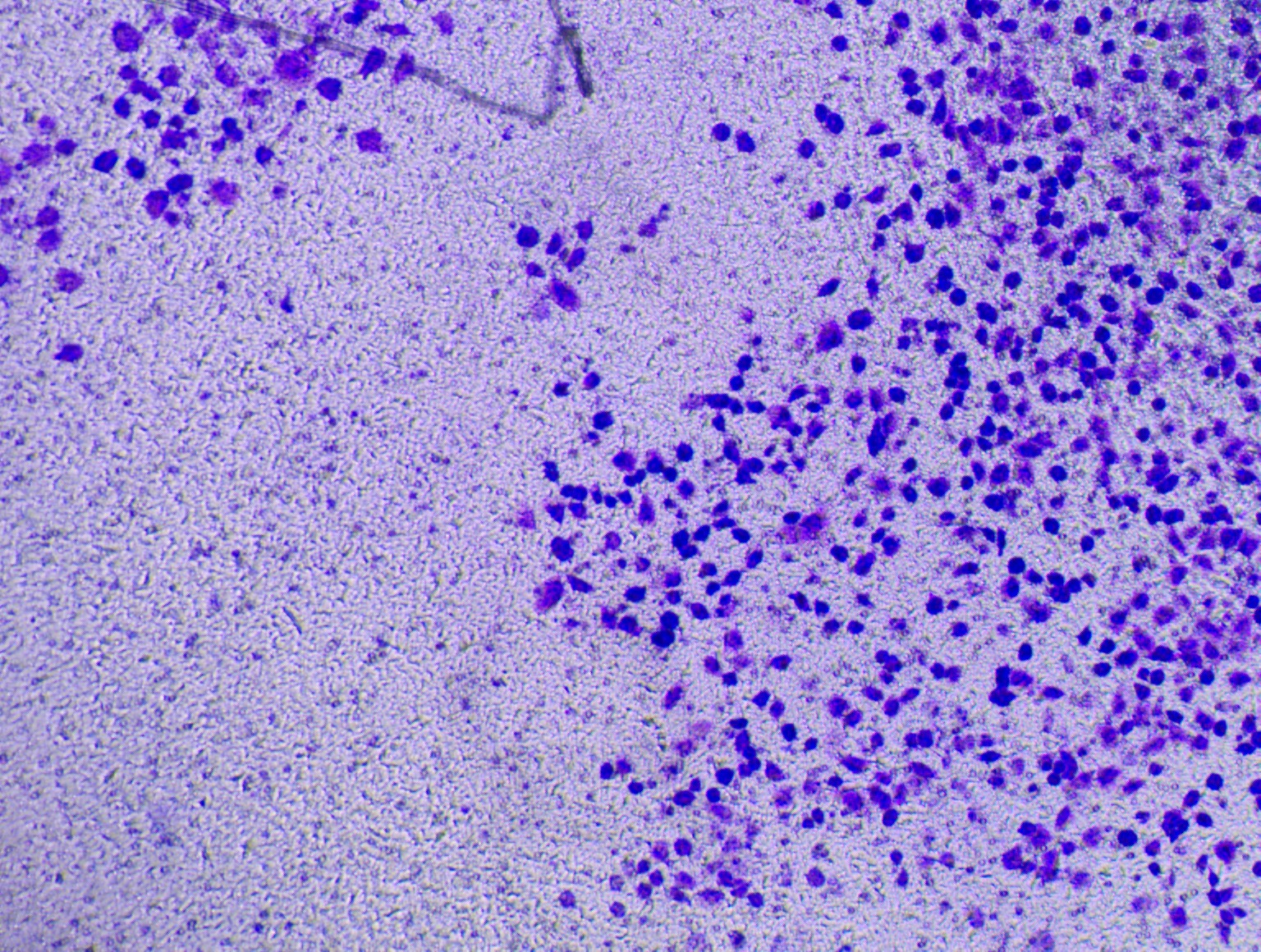

Supplement: Supplementary file 4 [file DataSheet4.zip › transwell-si-COL1A2/1-1.jpg]

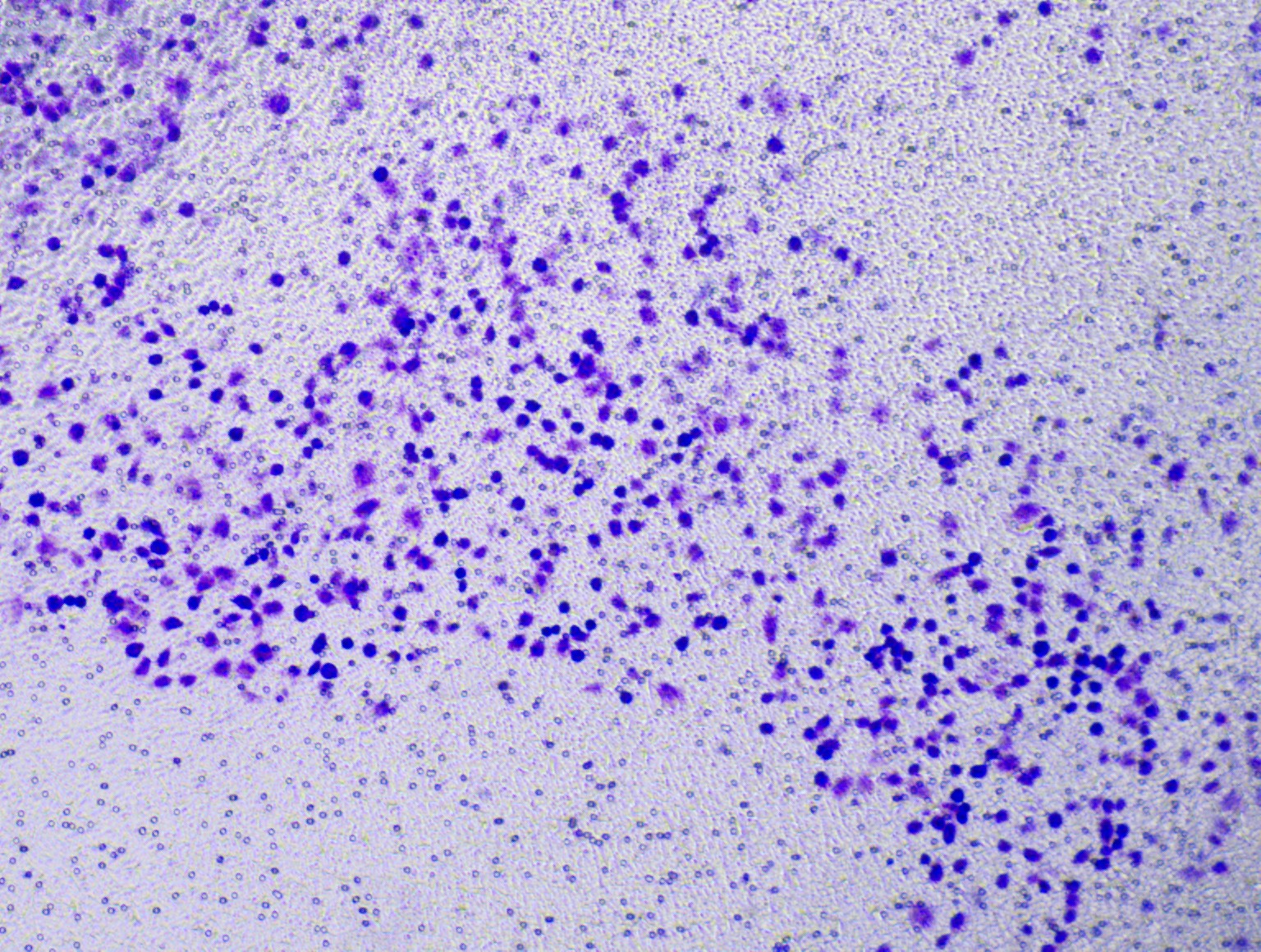

Supplement: Supplementary file 4 [file DataSheet4.zip › transwell-si-COL1A2/1-2.jpg]

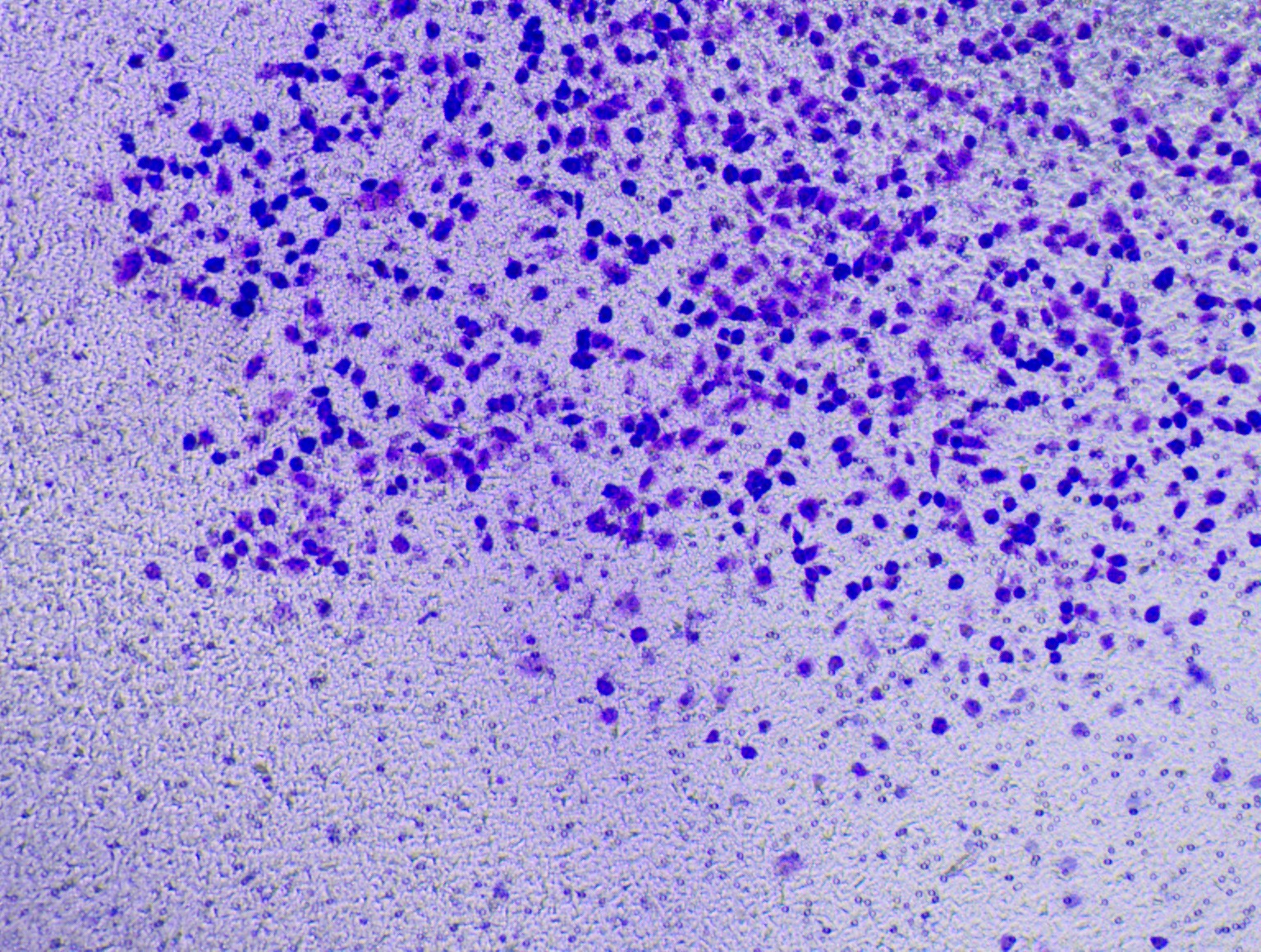

Supplement: Supplementary file 4 [file DataSheet4.zip › transwell-si-COL1A2/1-3.jpg]

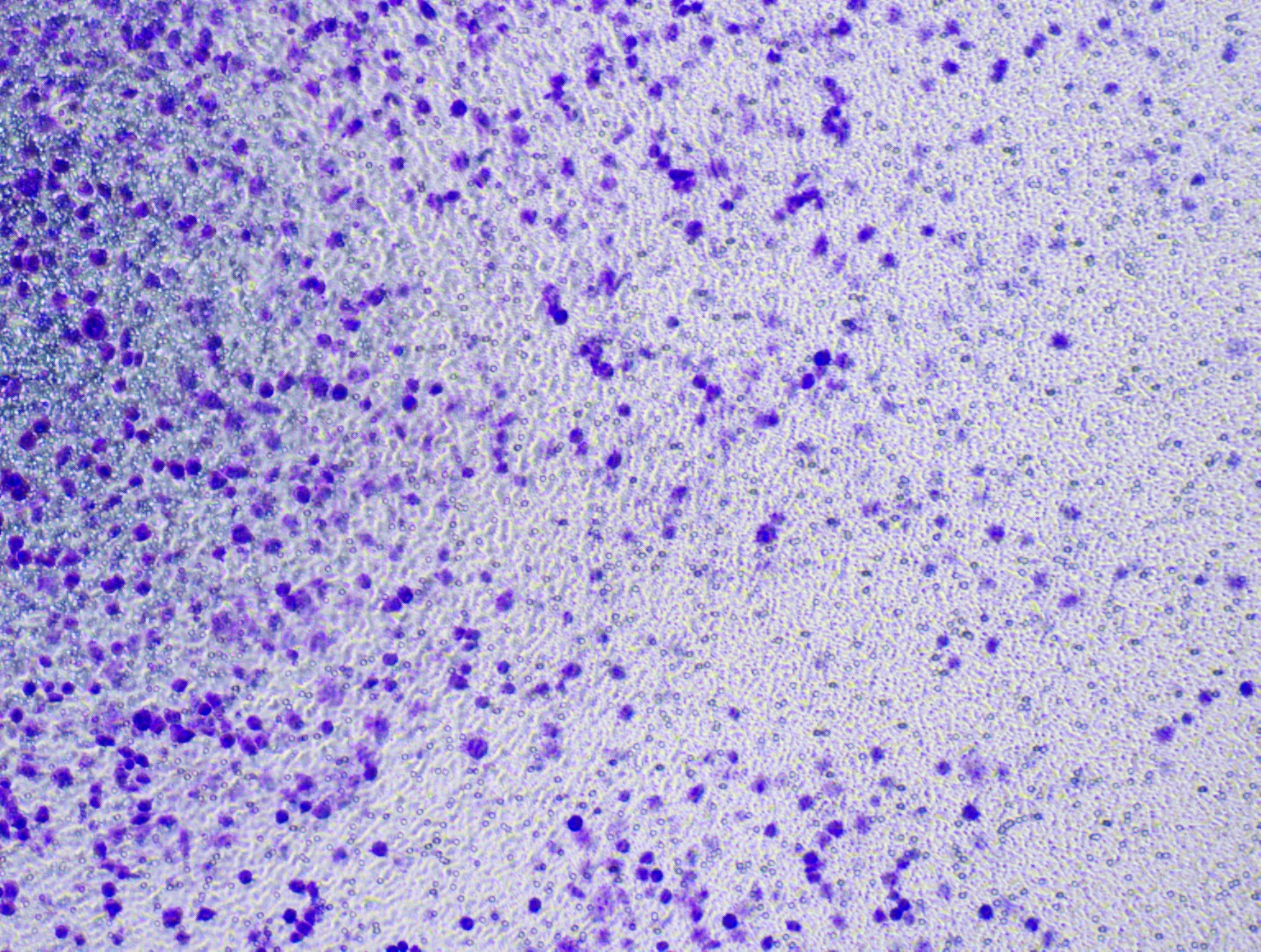

Supplement: Supplementary file 4 [file DataSheet4.zip › transwell-si-COL1A2/1-4.jpg]

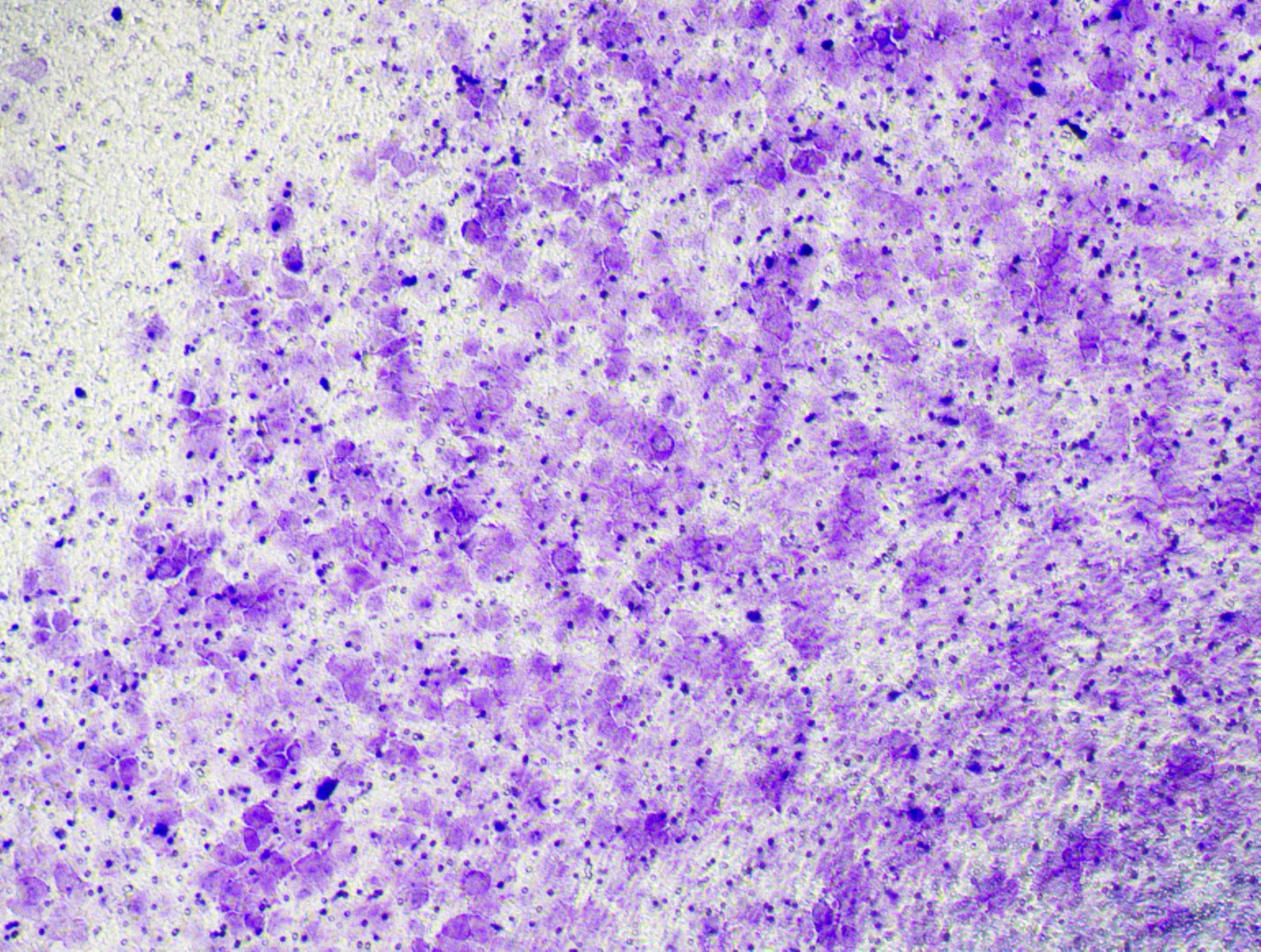

Supplement: Supplementary file 4 [file DataSheet4.zip › transwell-si-COL1A2/2-1.jpg]

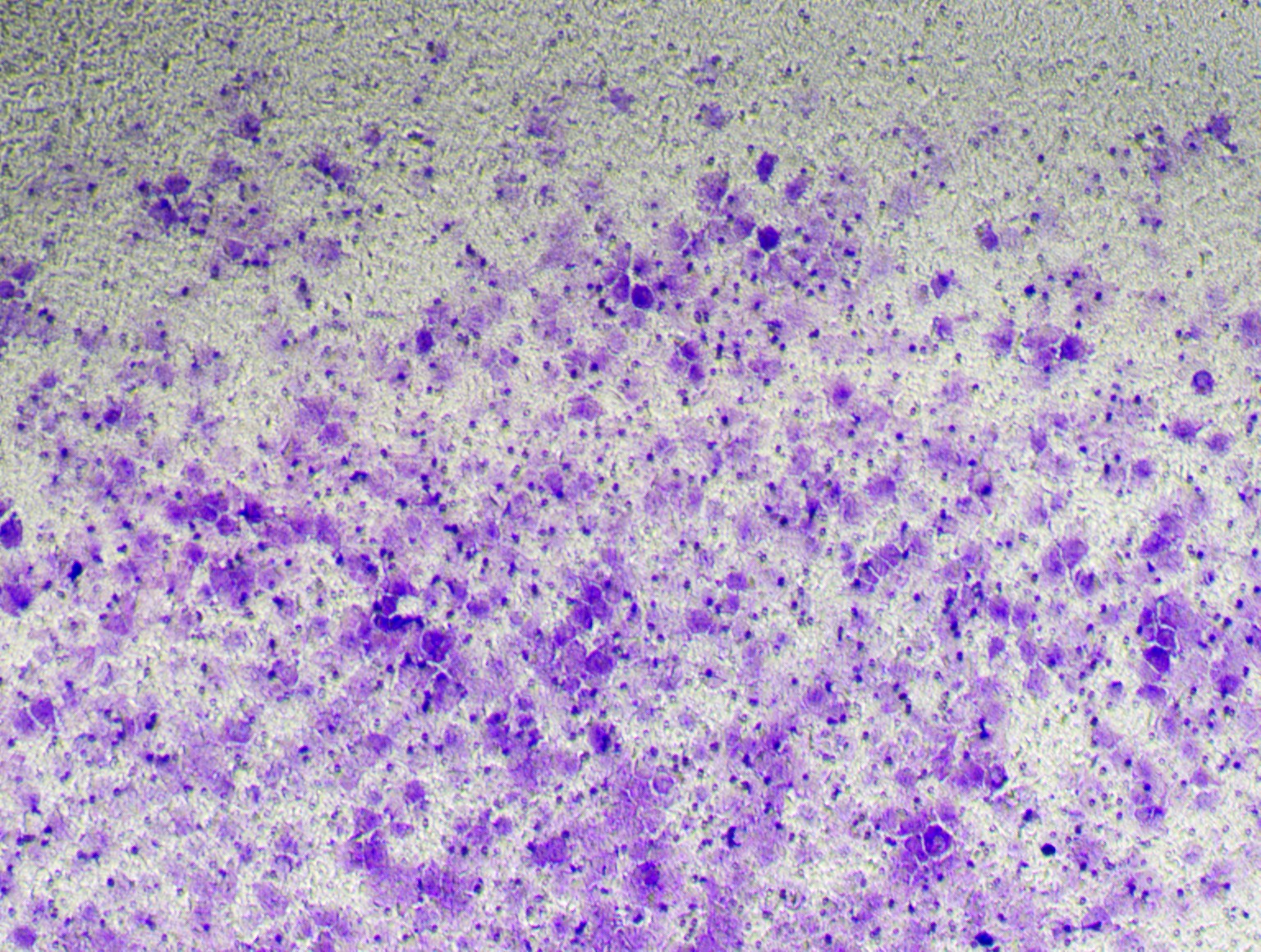

Supplement: Supplementary file 4 [file DataSheet4.zip › transwell-si-COL1A2/2-2.jpg]

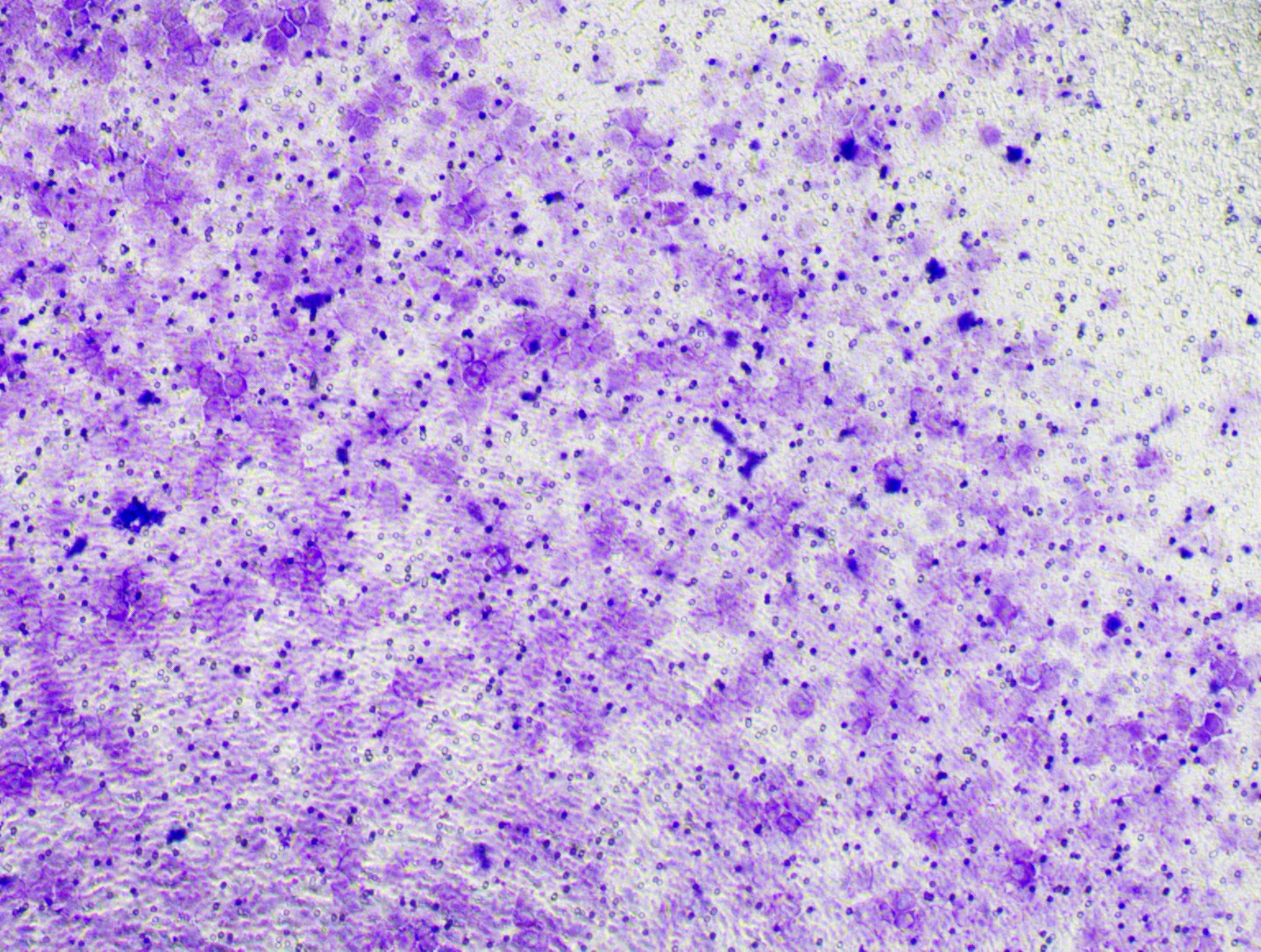

Supplement: Supplementary file 4 [file DataSheet4.zip › transwell-si-COL1A2/2-3.jpg]

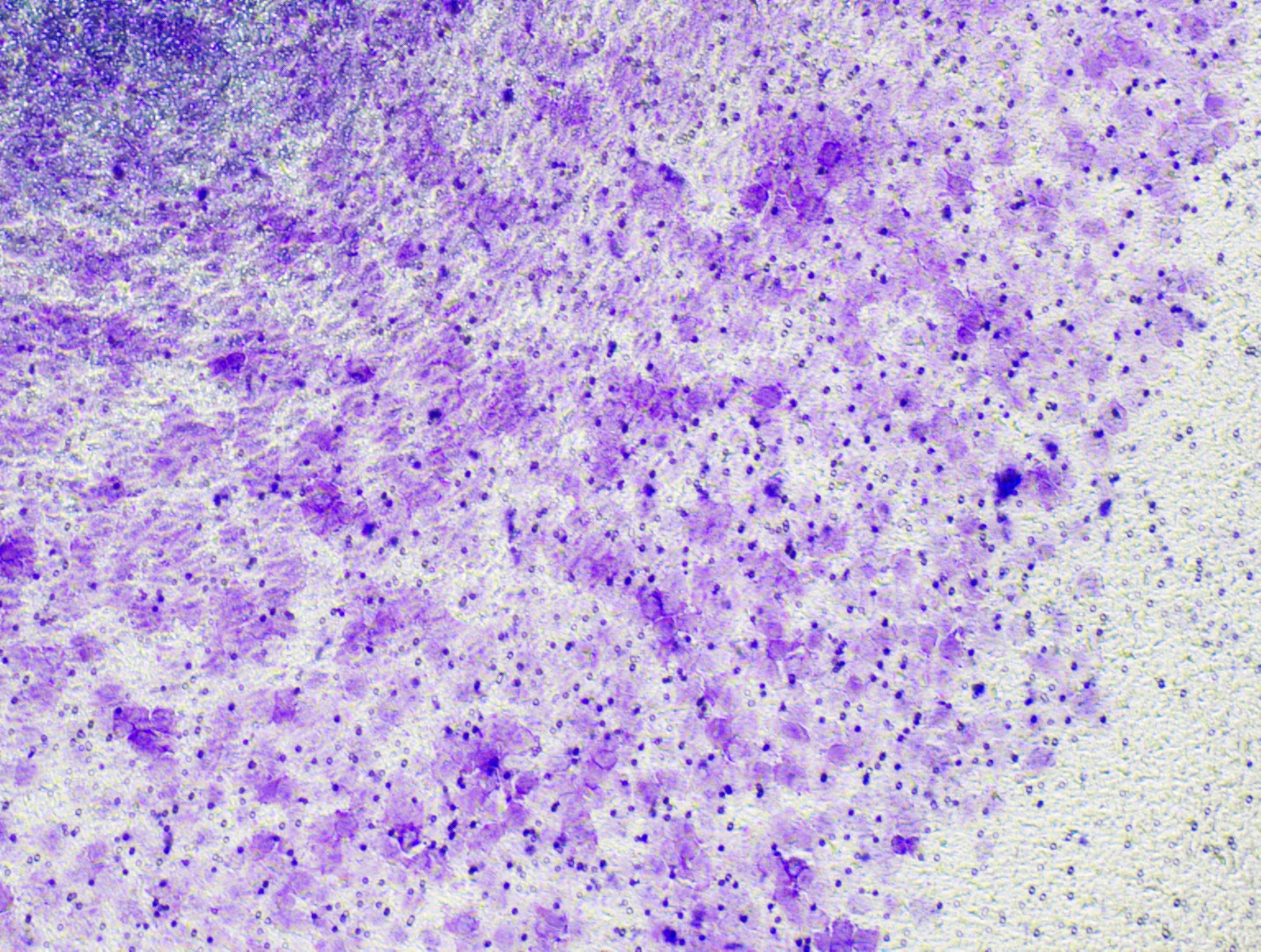

Supplement: Supplementary file 4 [file DataSheet4.zip › transwell-si-COL1A2/2-4.jpg]

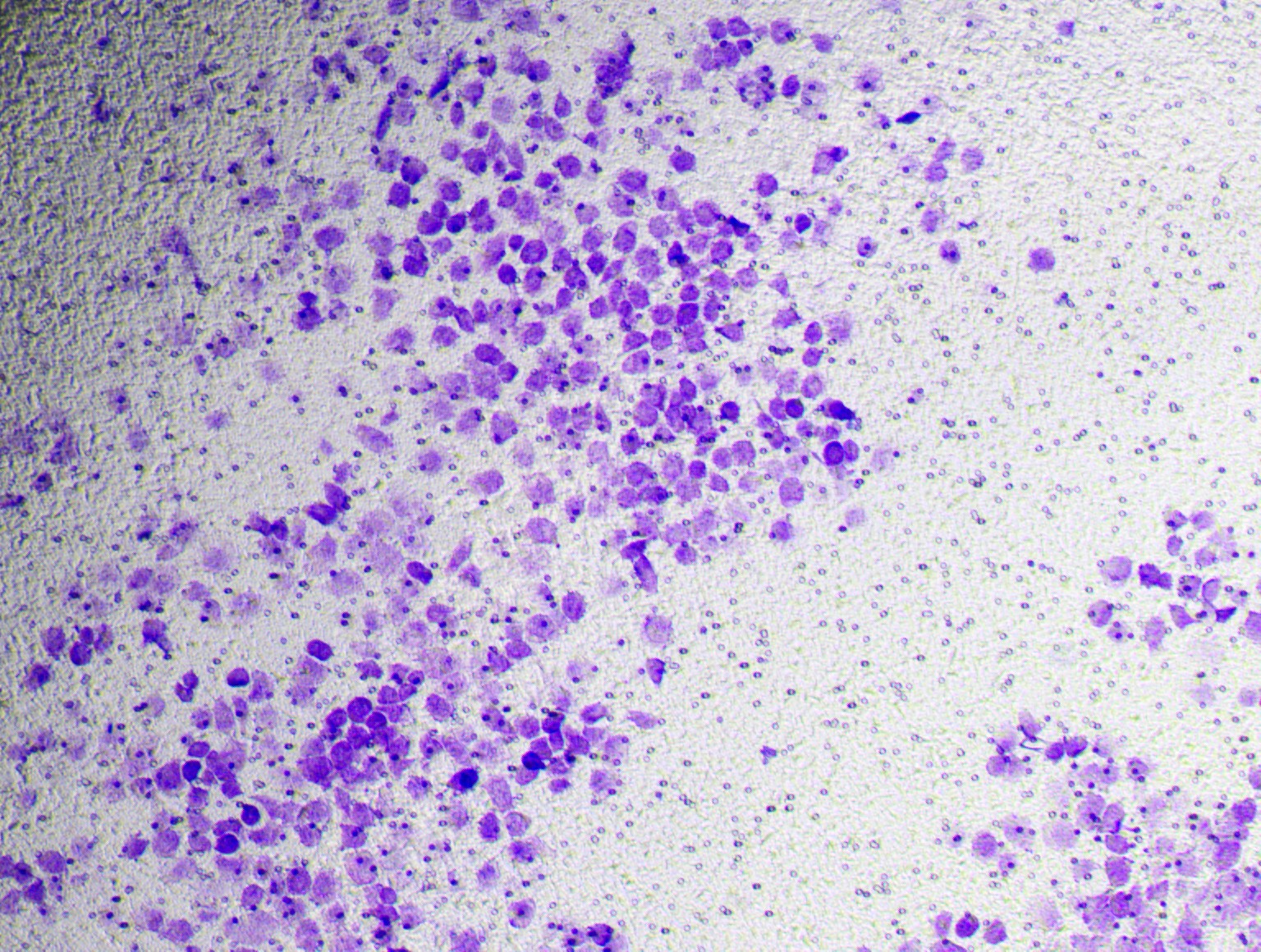

Supplement: Supplementary file 4 [file DataSheet4.zip › transwell-si-COL1A2/3-1.jpg]

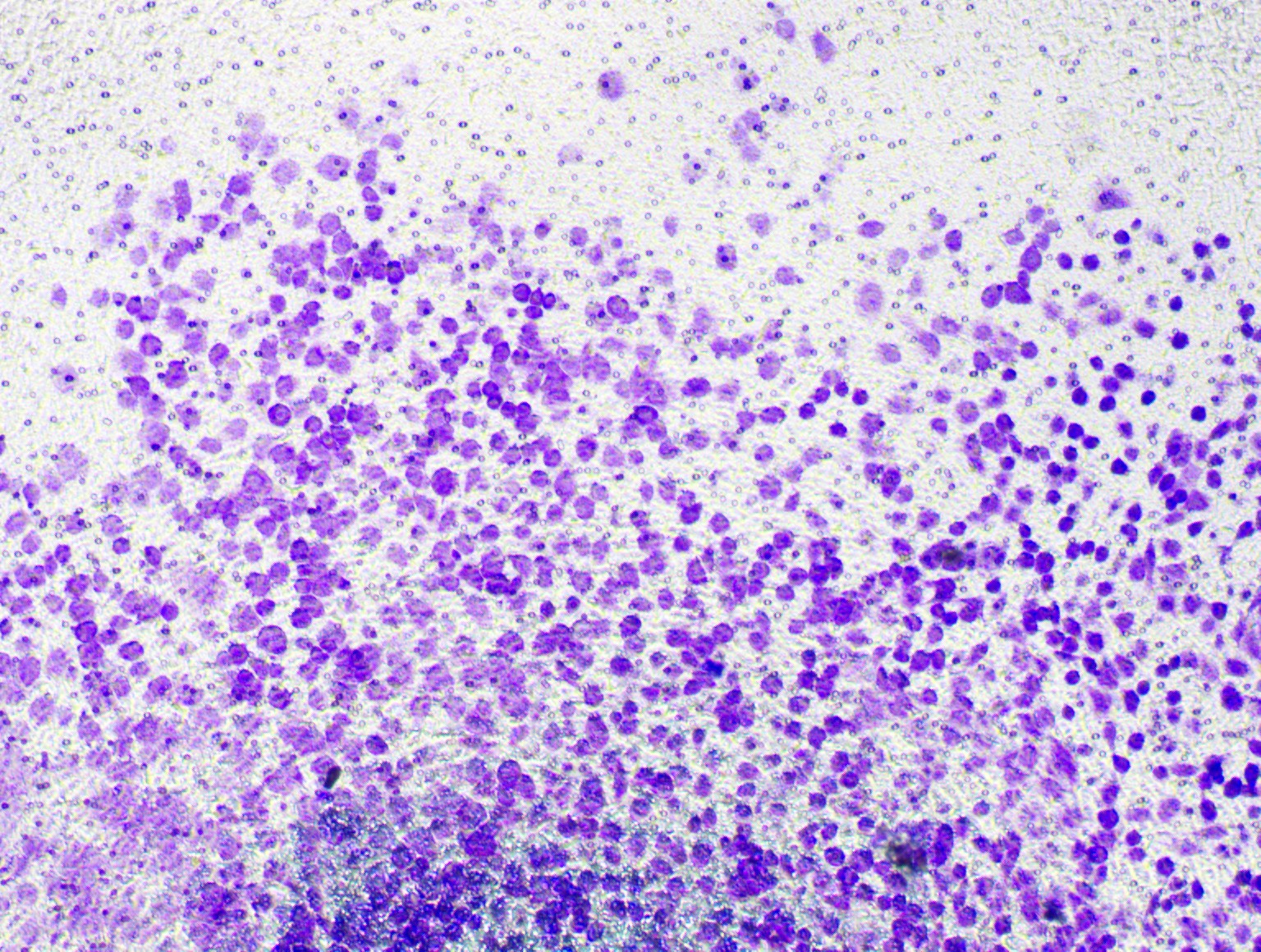

Supplement: Supplementary file 4 [file DataSheet4.zip › transwell-si-COL1A2/3-2.jpg]

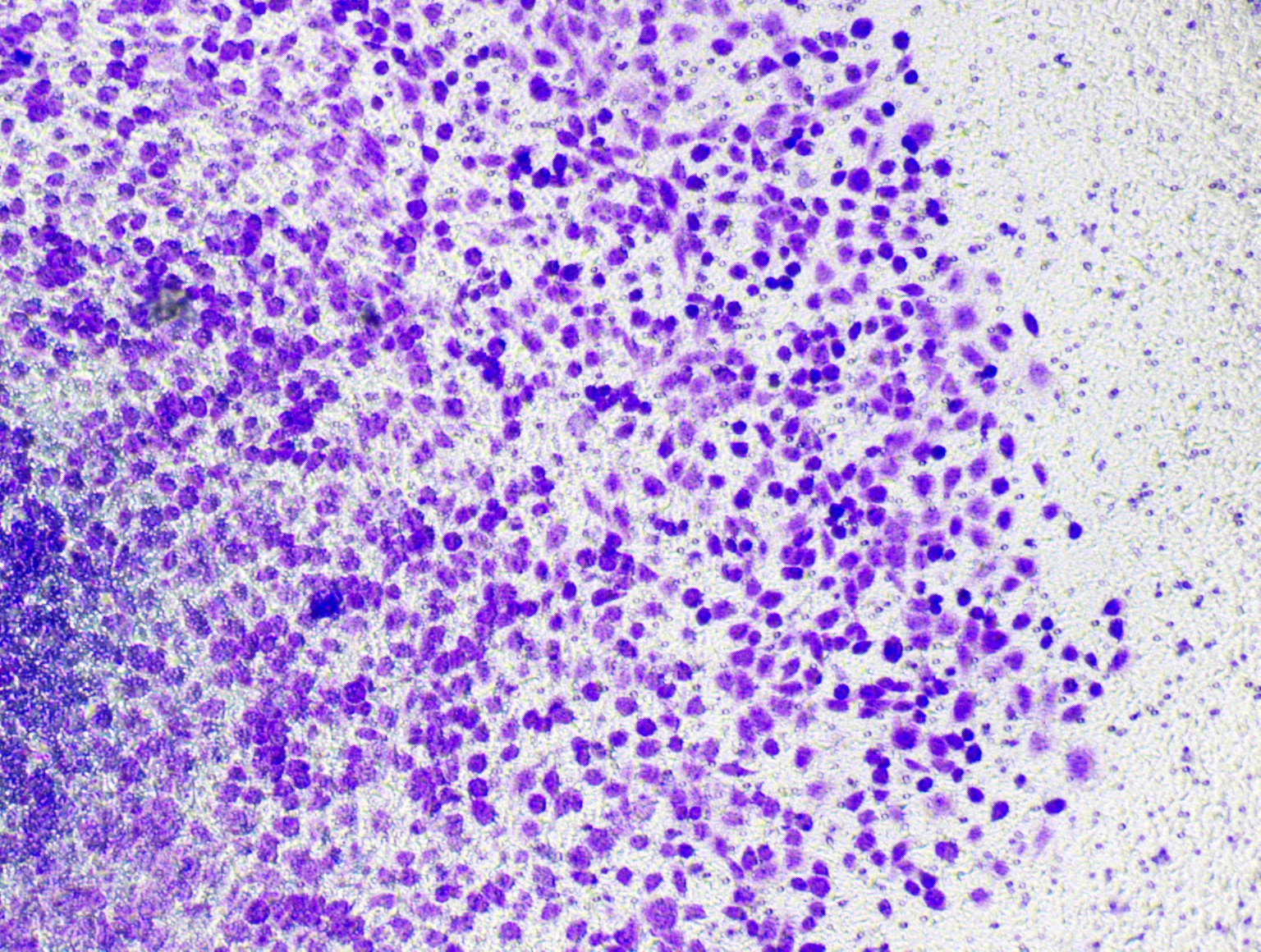

Supplement: Supplementary file 4 [file DataSheet4.zip › transwell-si-COL1A2/3-3.jpg]

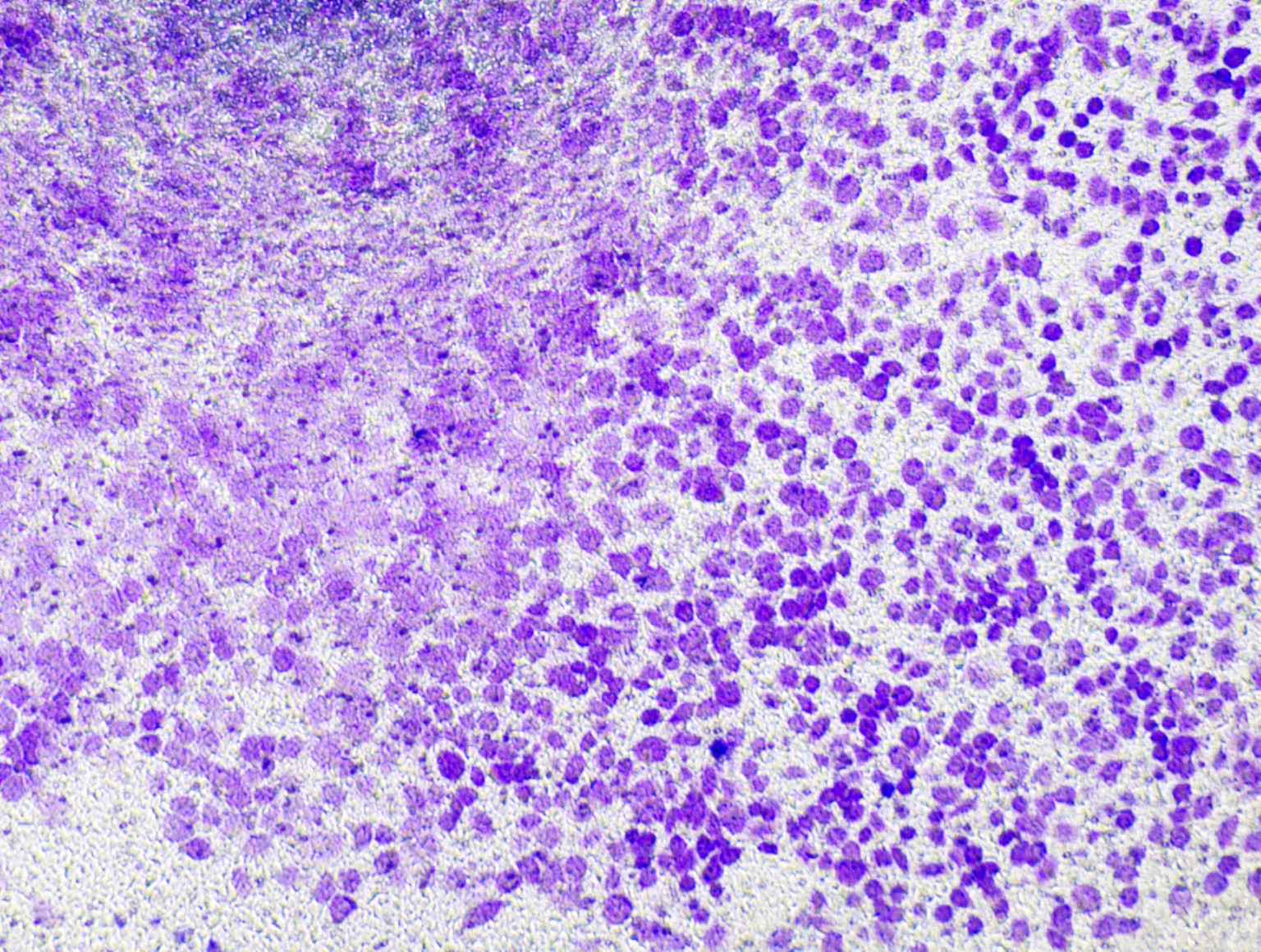

Supplement: Supplementary file 4 [file DataSheet4.zip › transwell-si-COL1A2/3-4.jpg]

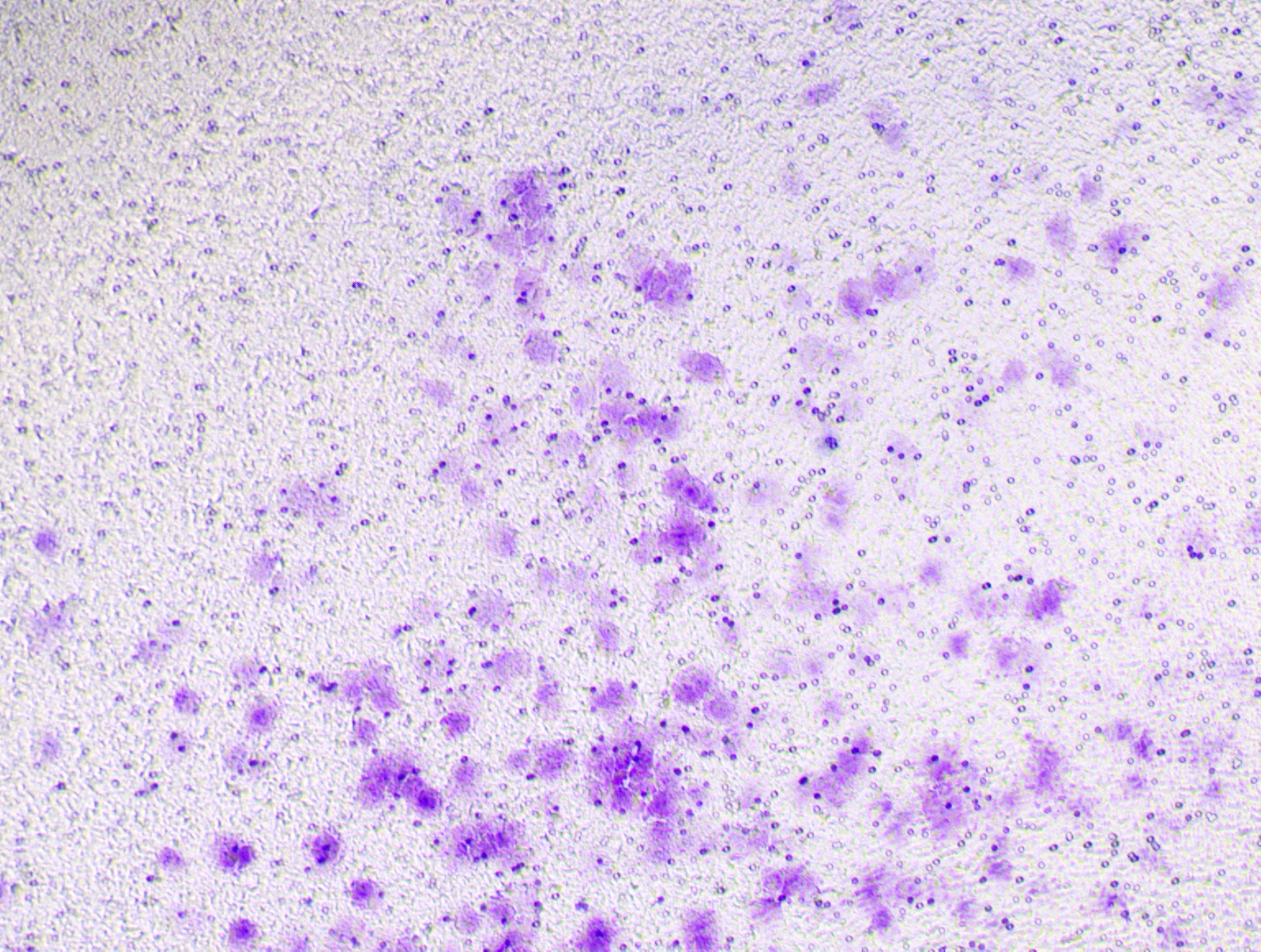

Supplement: Supplementary file 4 [file DataSheet4.zip › transwell-si-COL1A2/4-1.jpg]

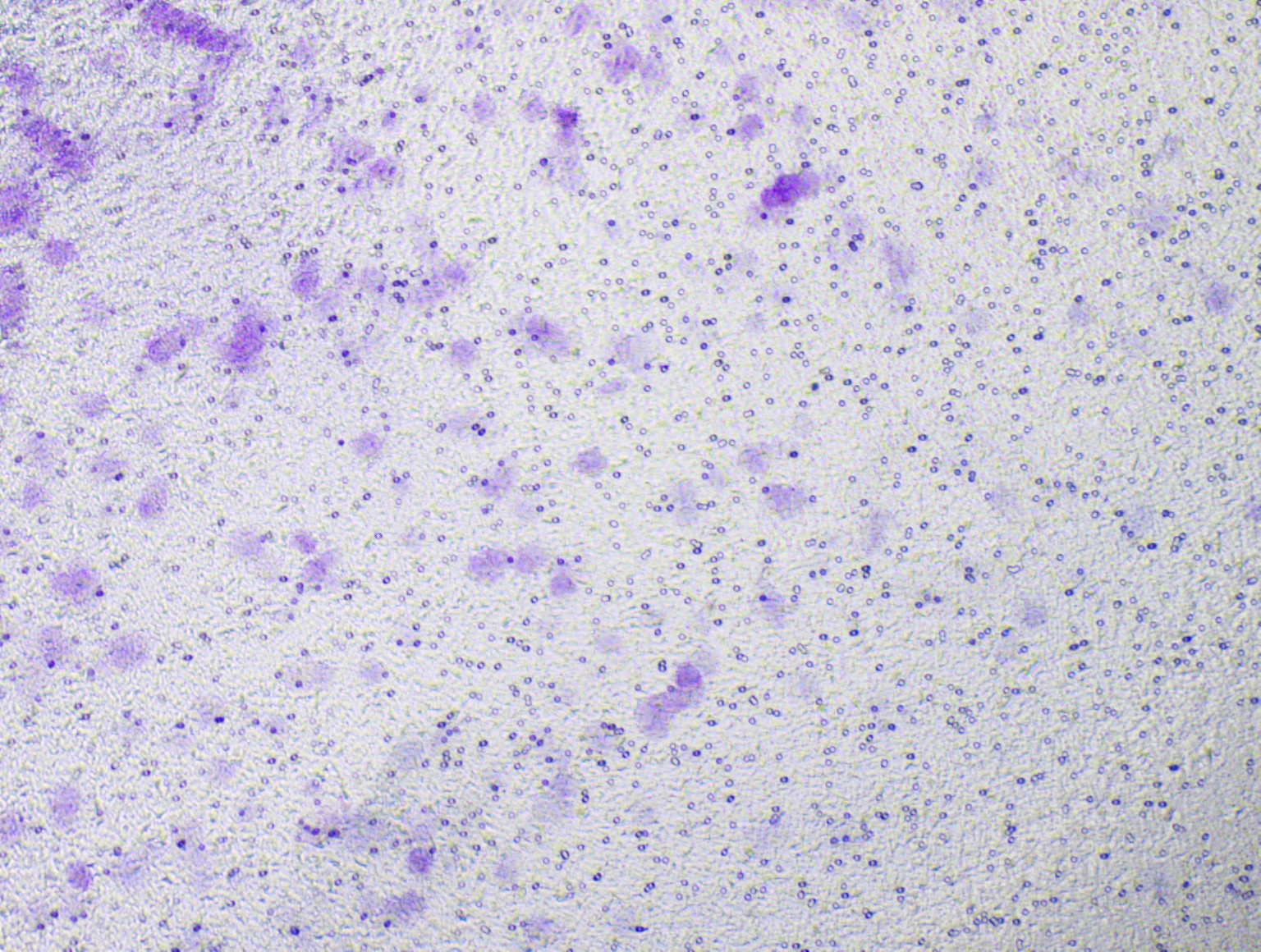

Supplement: Supplementary file 4 [file DataSheet4.zip › transwell-si-COL1A2/4-2.jpg]

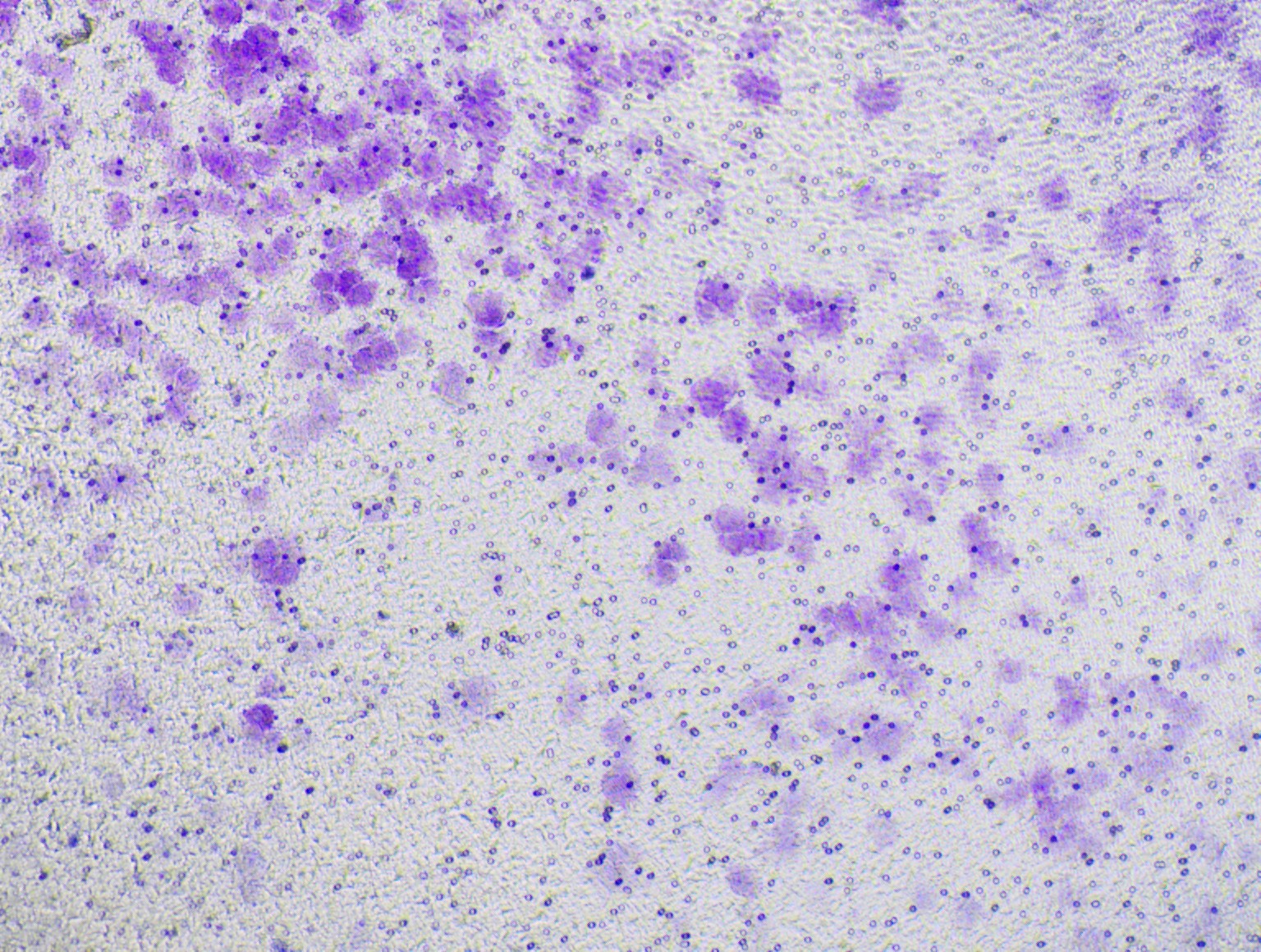

Supplement: Supplementary file 4 [file DataSheet4.zip › transwell-si-COL1A2/4-3.jpg]

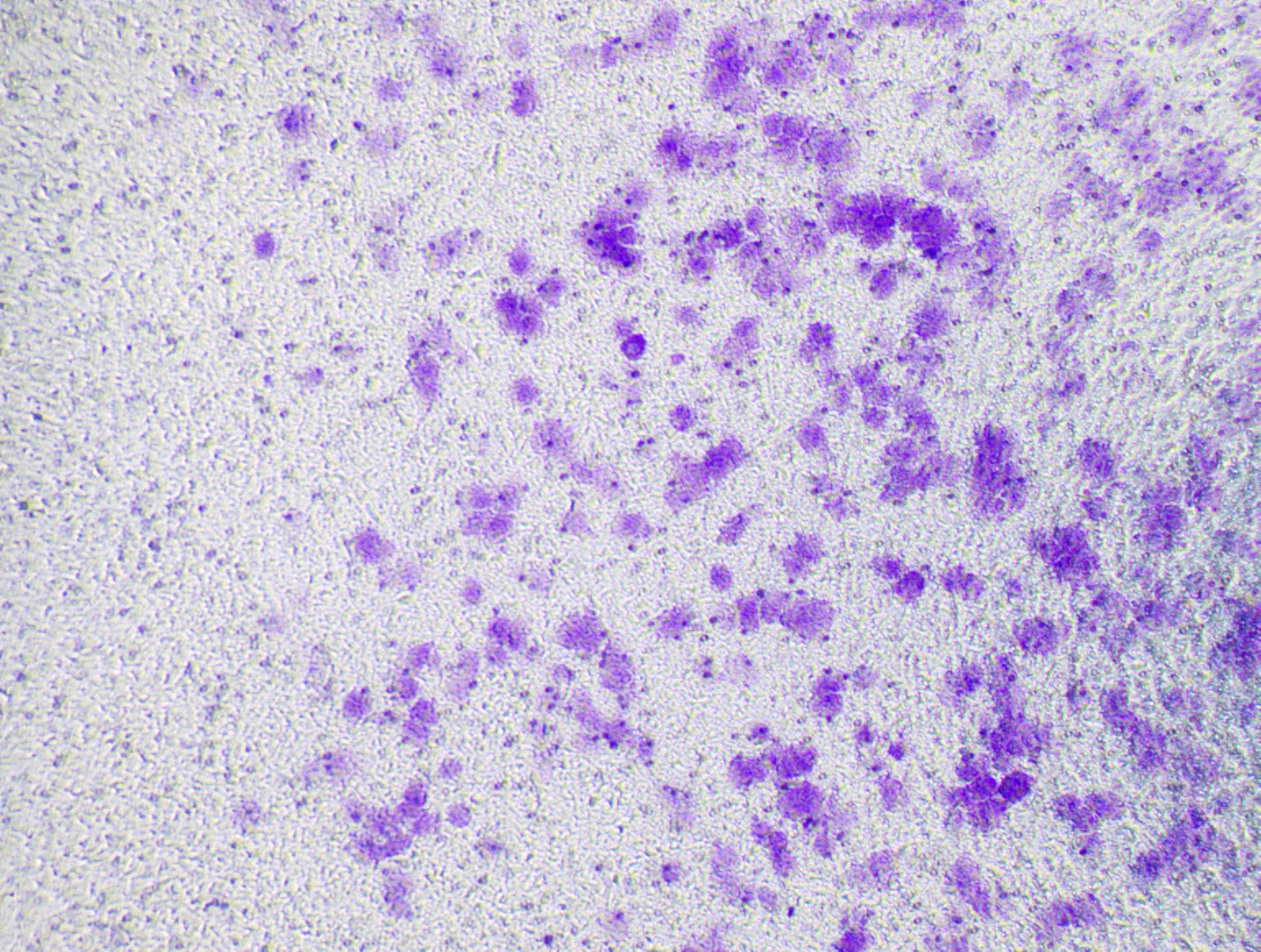

Supplement: Supplementary file 4 [file DataSheet4.zip › transwell-si-COL1A2/4-4.jpg]

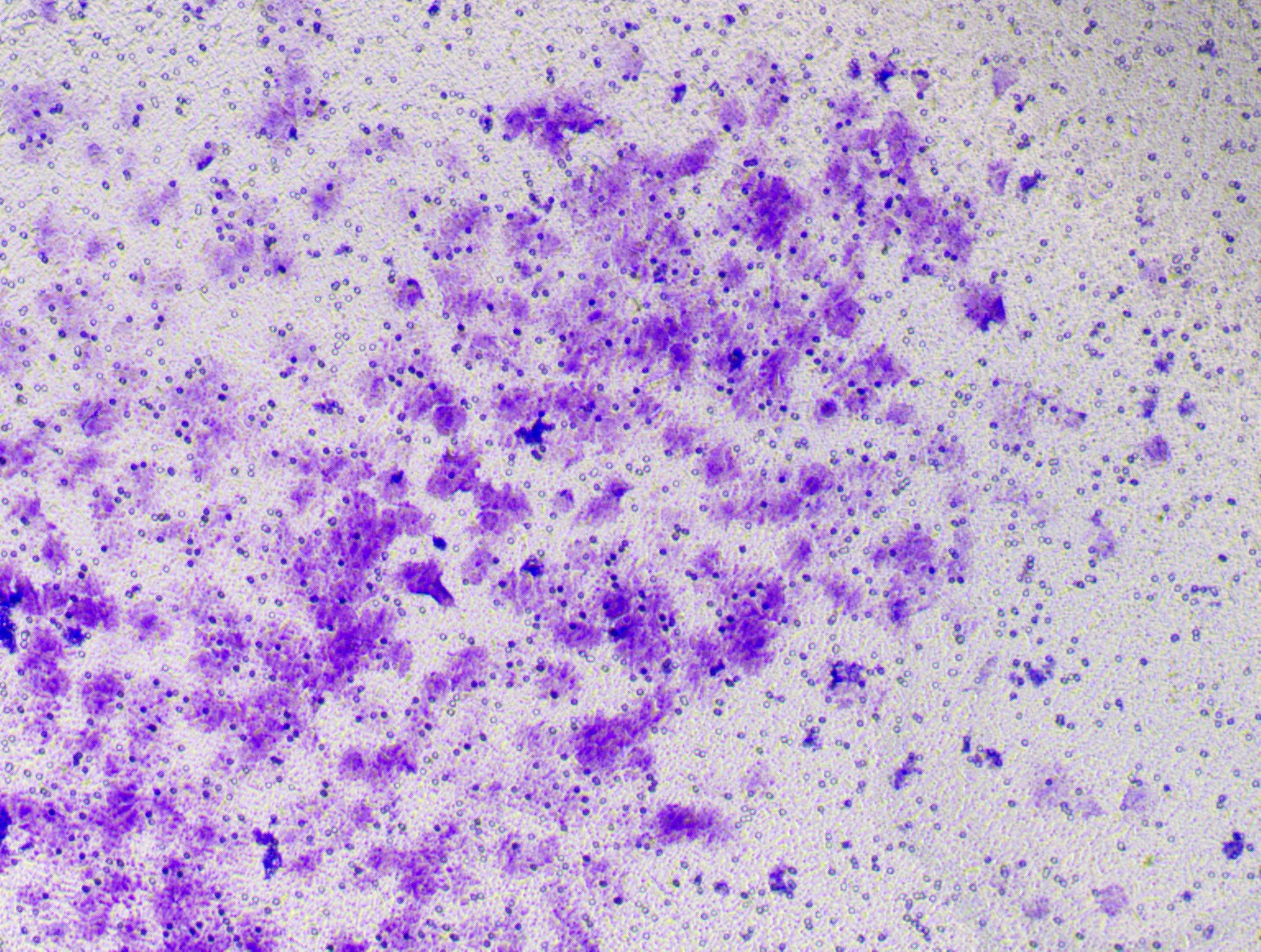

Supplement: Supplementary file 4 [file DataSheet4.zip › transwell-si-COL1A2/5-1.jpg]

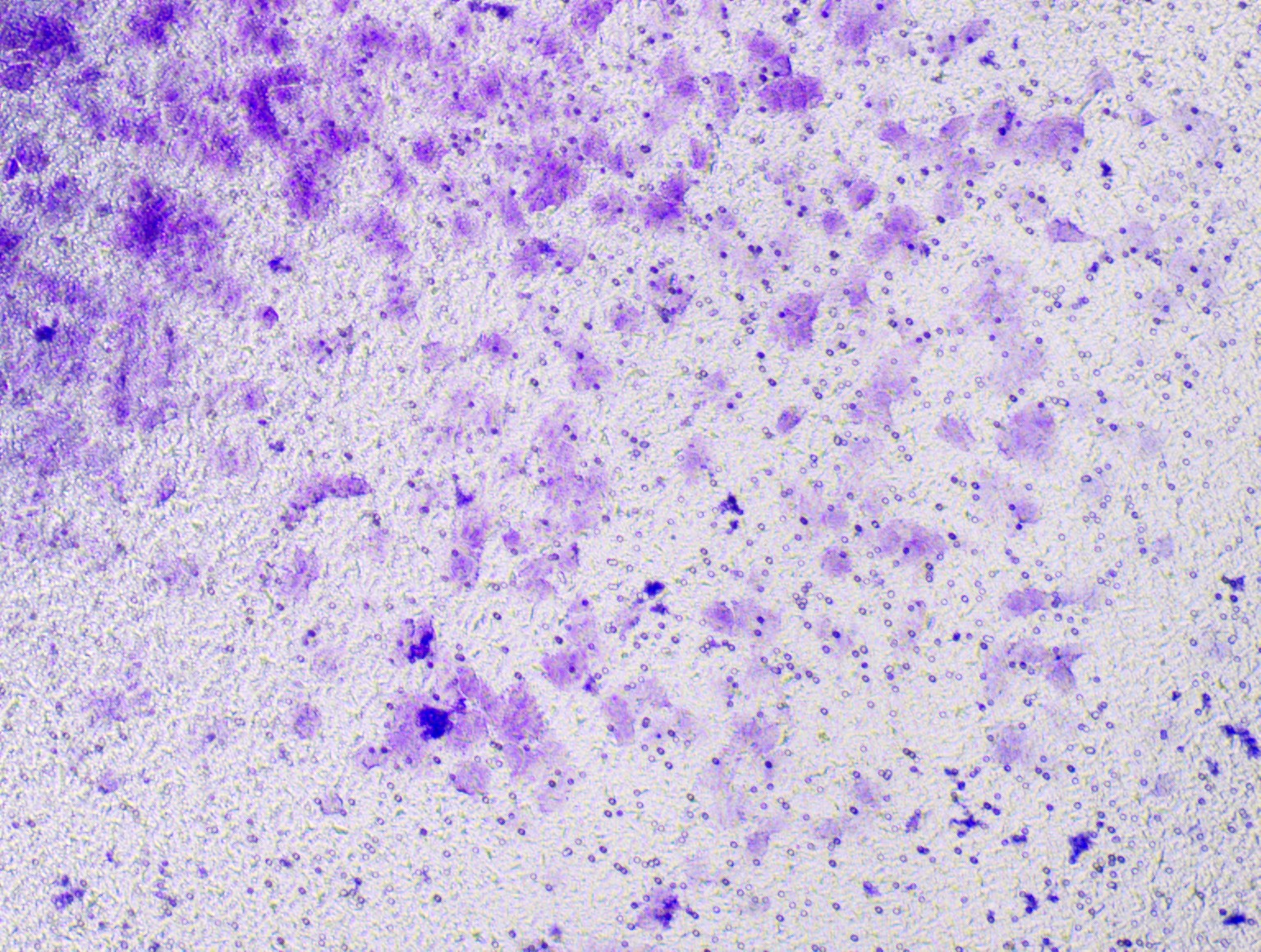

Supplement: Supplementary file 4 [file DataSheet4.zip › transwell-si-COL1A2/5-2.jpg]

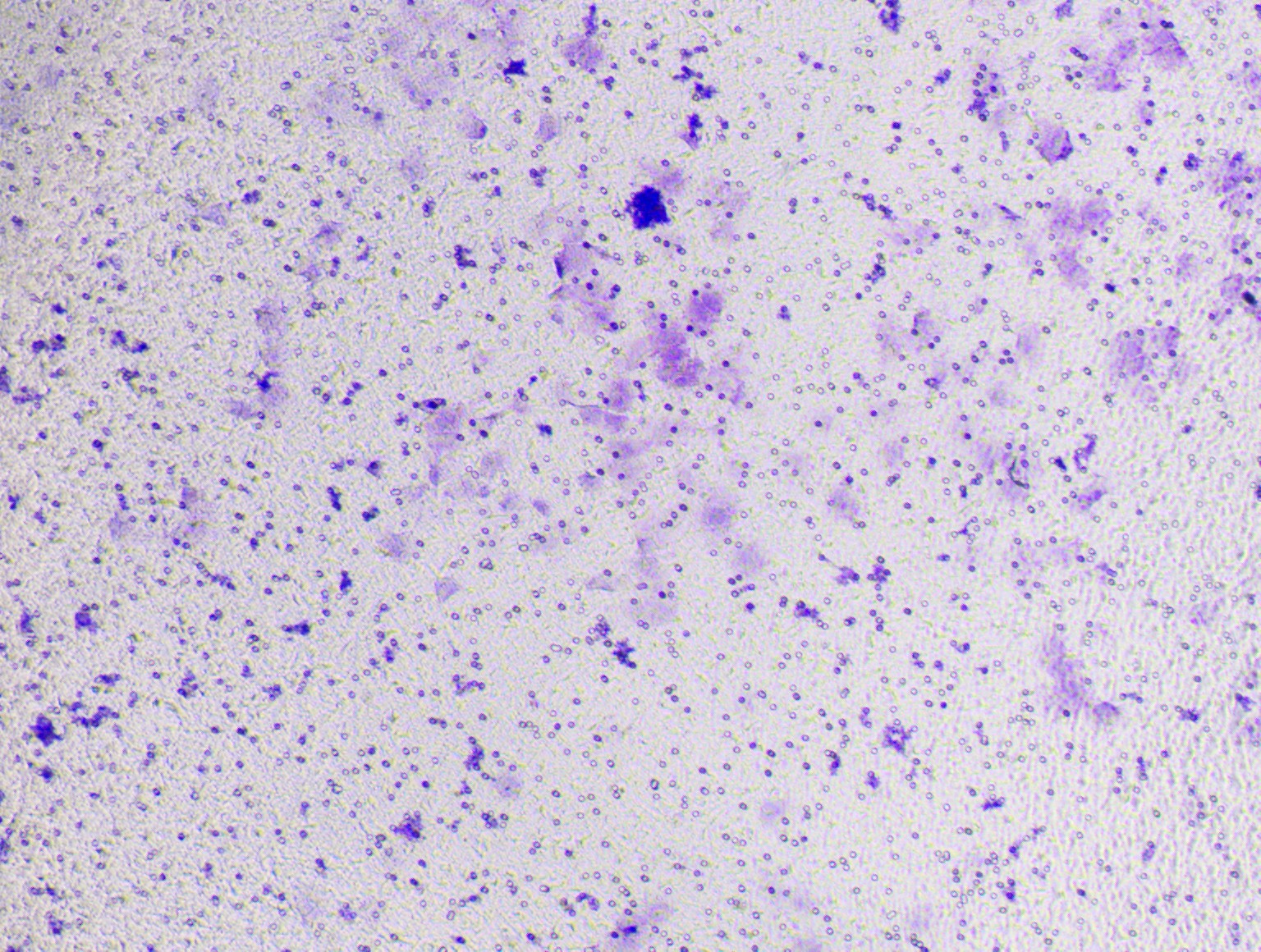

Supplement: Supplementary file 4 [file DataSheet4.zip › transwell-si-COL1A2/5-3.jpg]

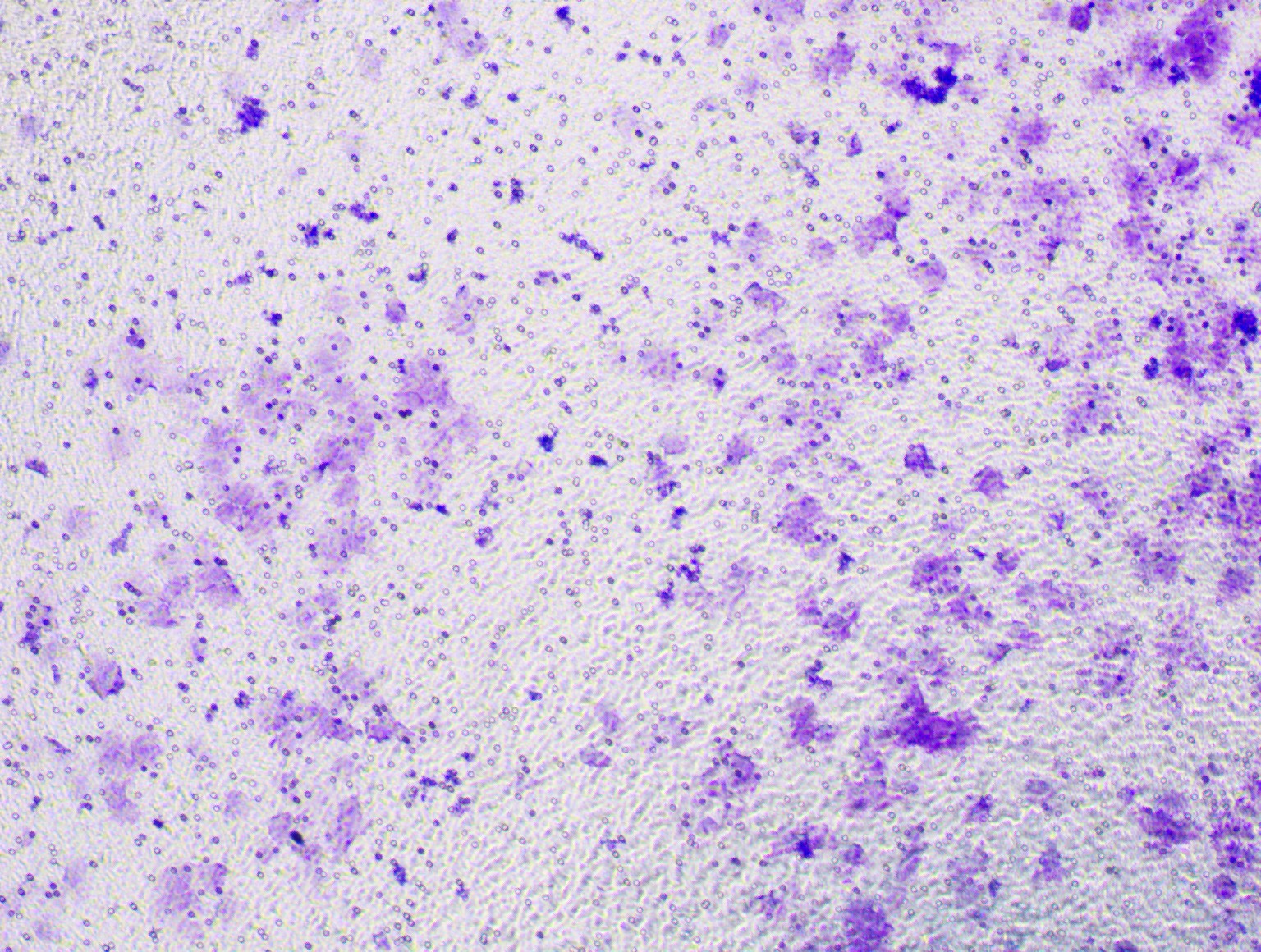

Supplement: Supplementary file 4 [file DataSheet4.zip › transwell-si-COL1A2/5-4.jpg]

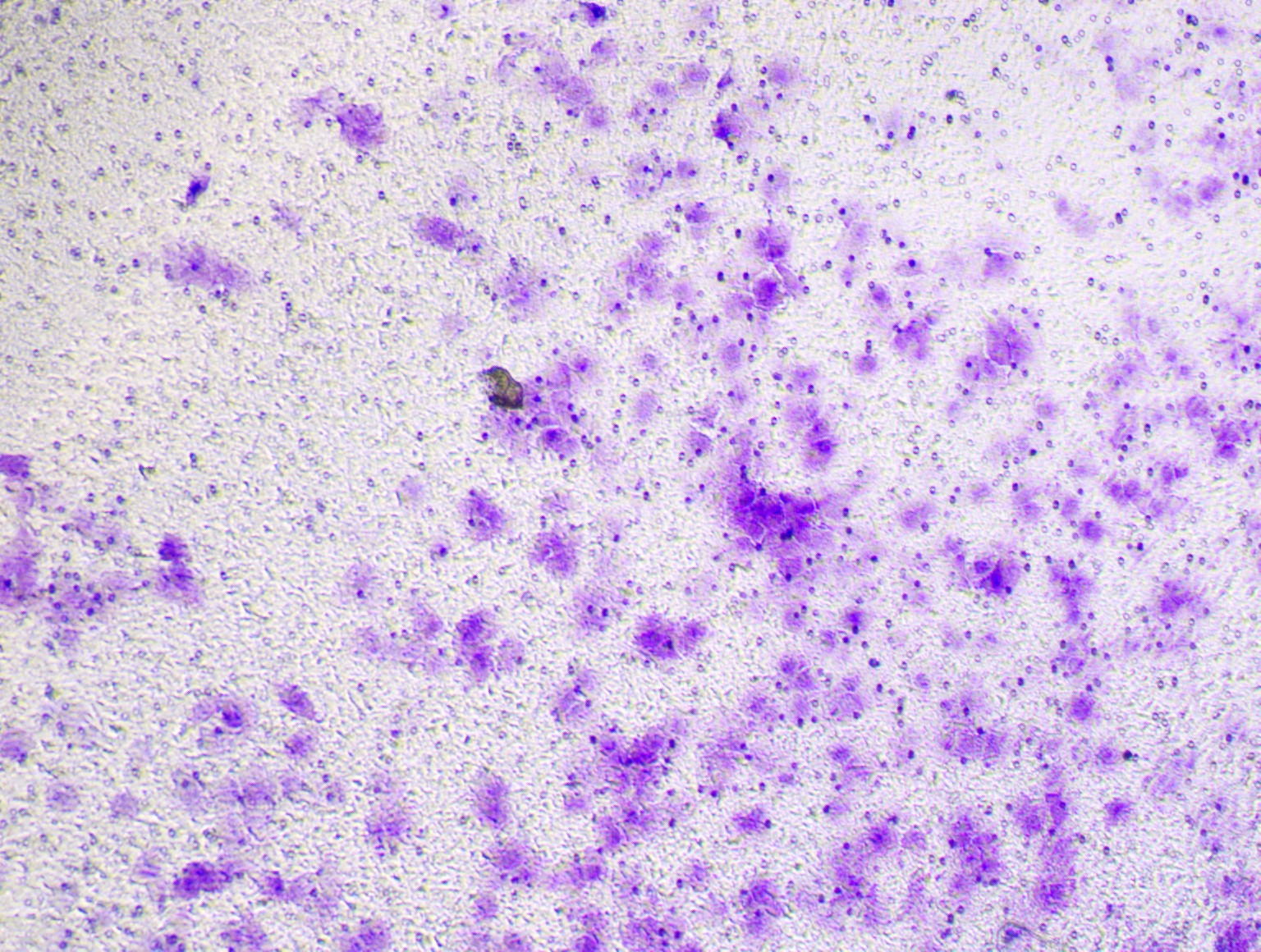

Supplement: Supplementary file 4 [file DataSheet4.zip › transwell-si-COL1A2/6-1.jpg]

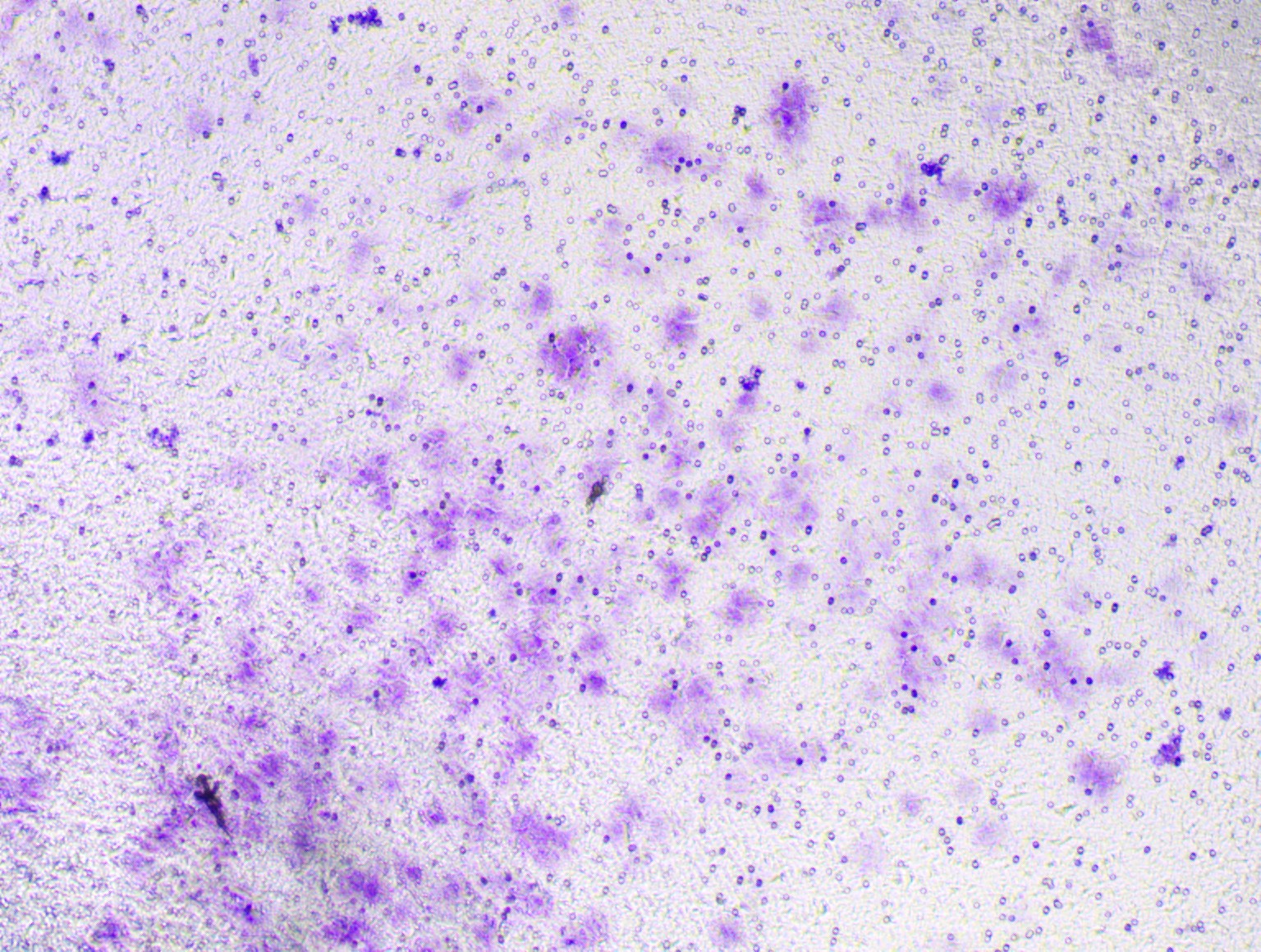

Supplement: Supplementary file 4 [file DataSheet4.zip › transwell-si-COL1A2/6-2.jpg]

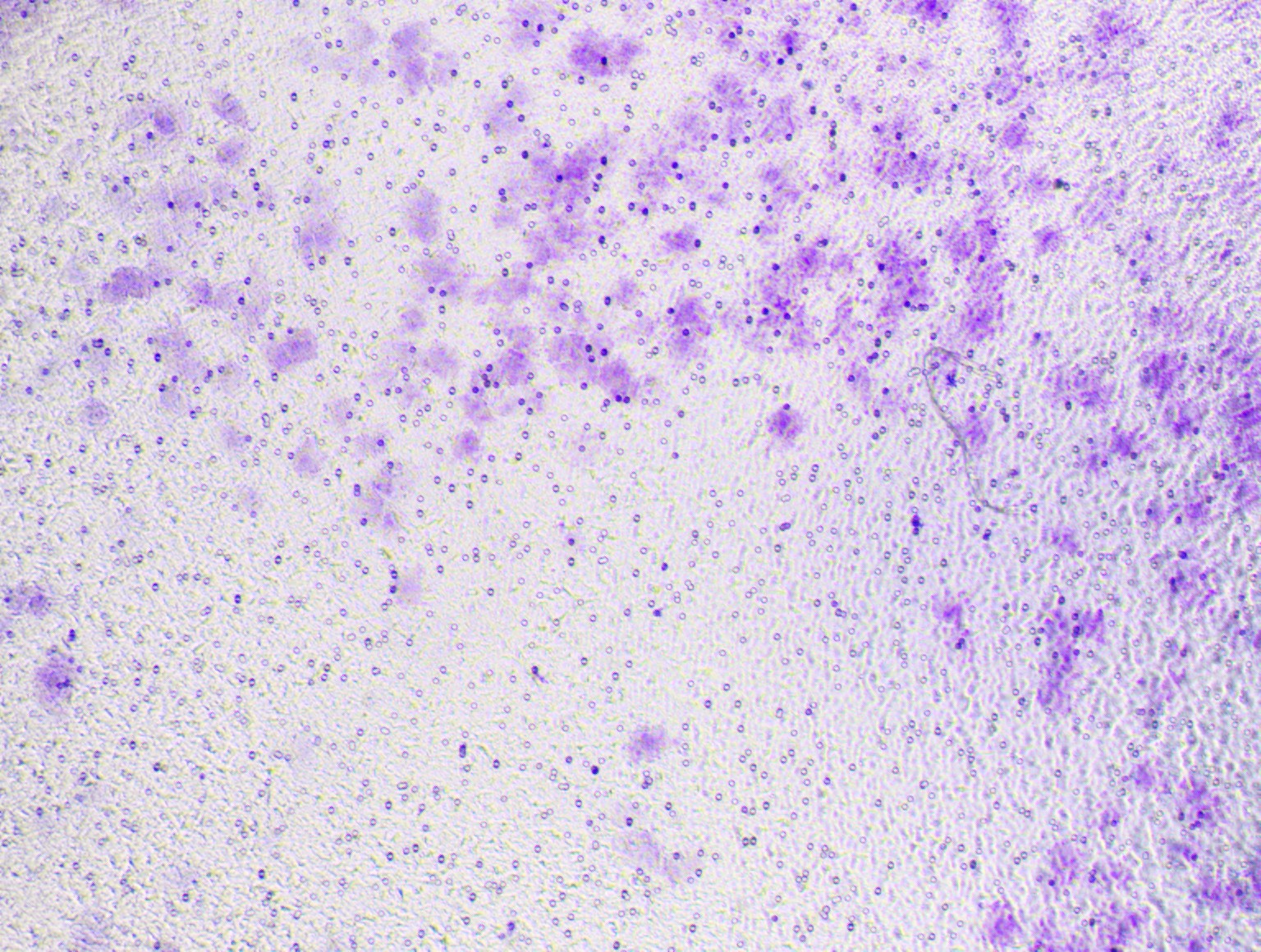

Supplement: Supplementary file 4 [file DataSheet4.zip › transwell-si-COL1A2/6-3.jpg]

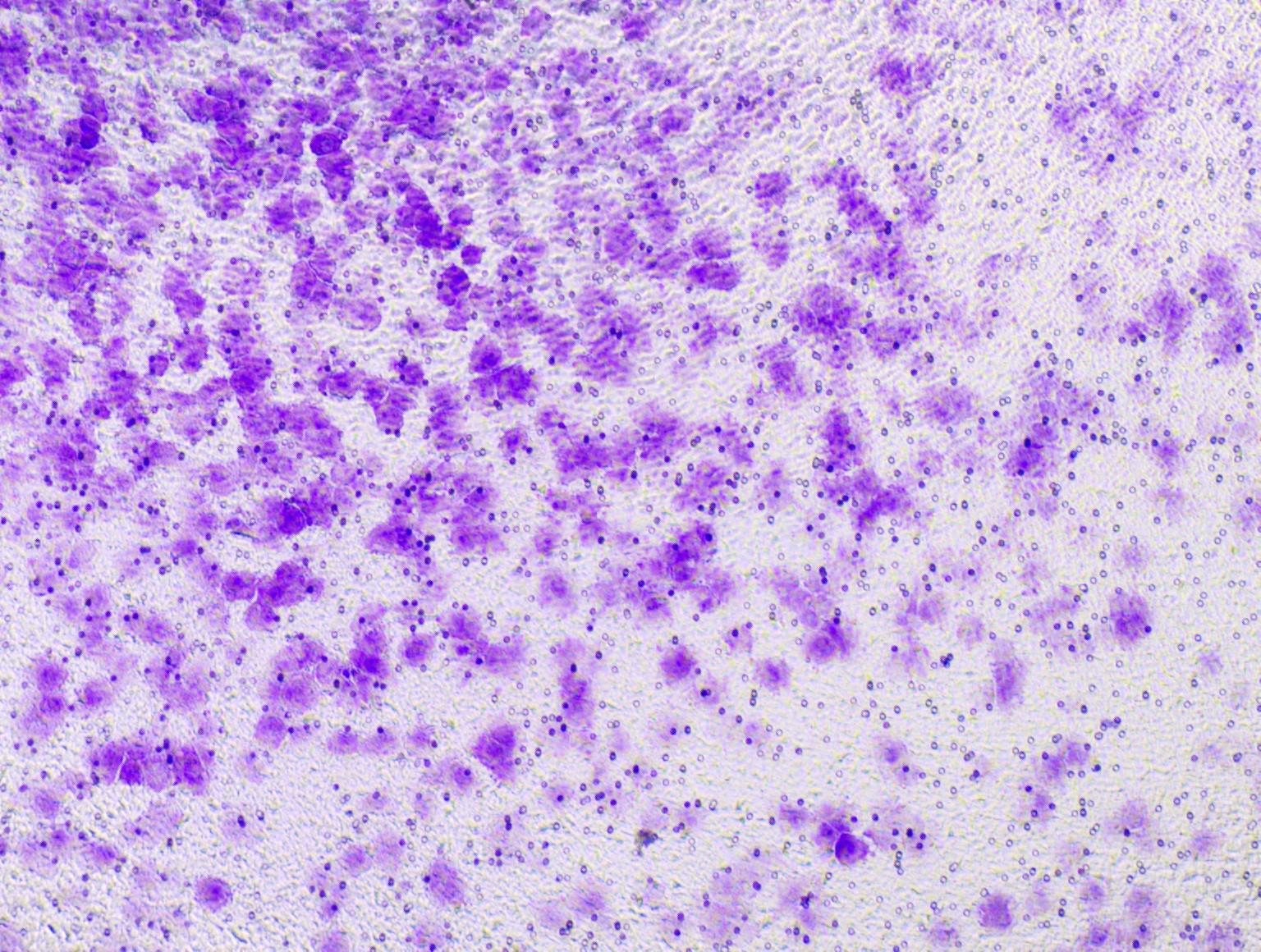

Supplement: Supplementary file 4 [file DataSheet4.zip › transwell-si-COL1A2/6-4.jpg]

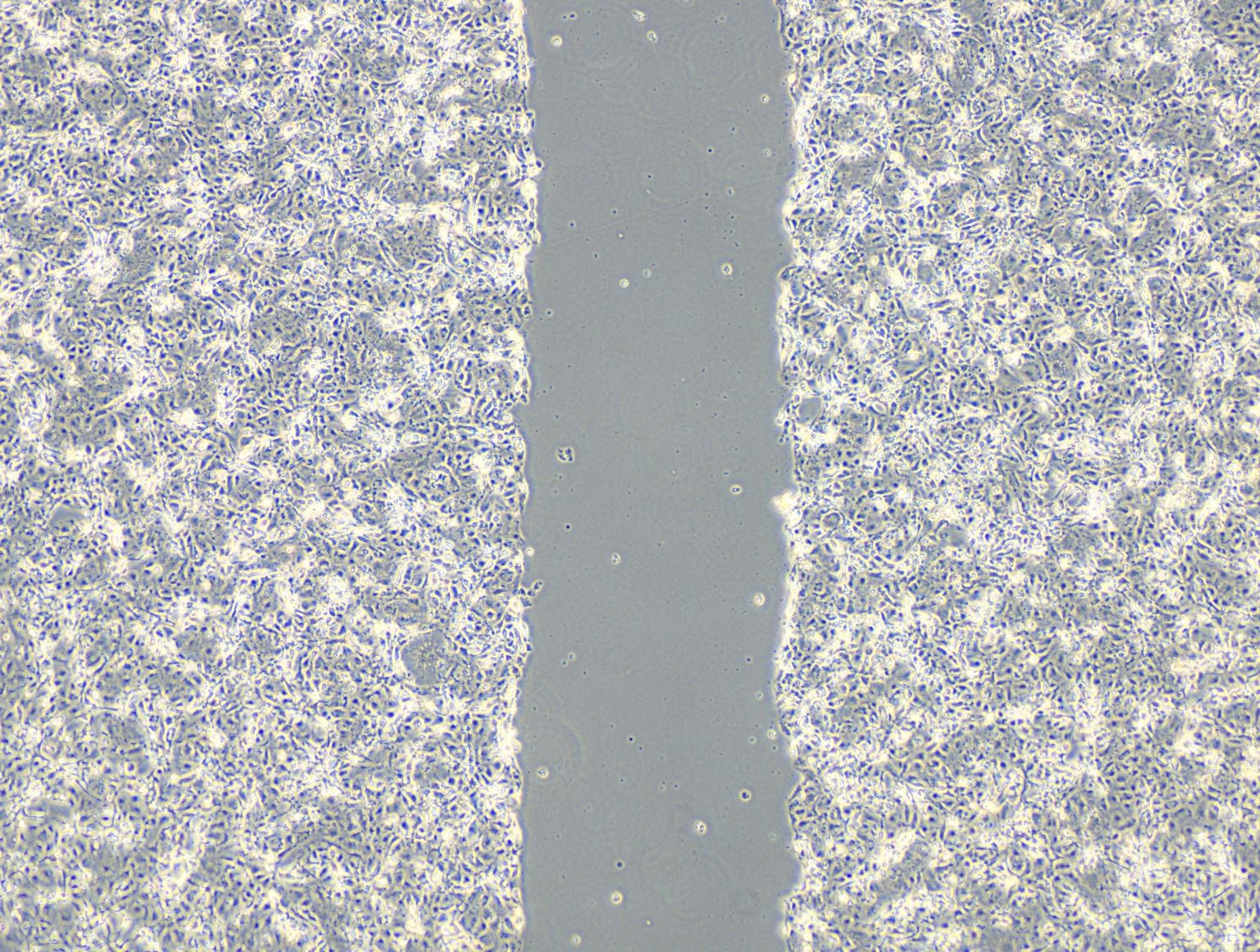

Supplement: Supplementary file 6 [file DataSheet6.zip › wound healing assay-oe-COL1A2/1-NC-0h.jpg]

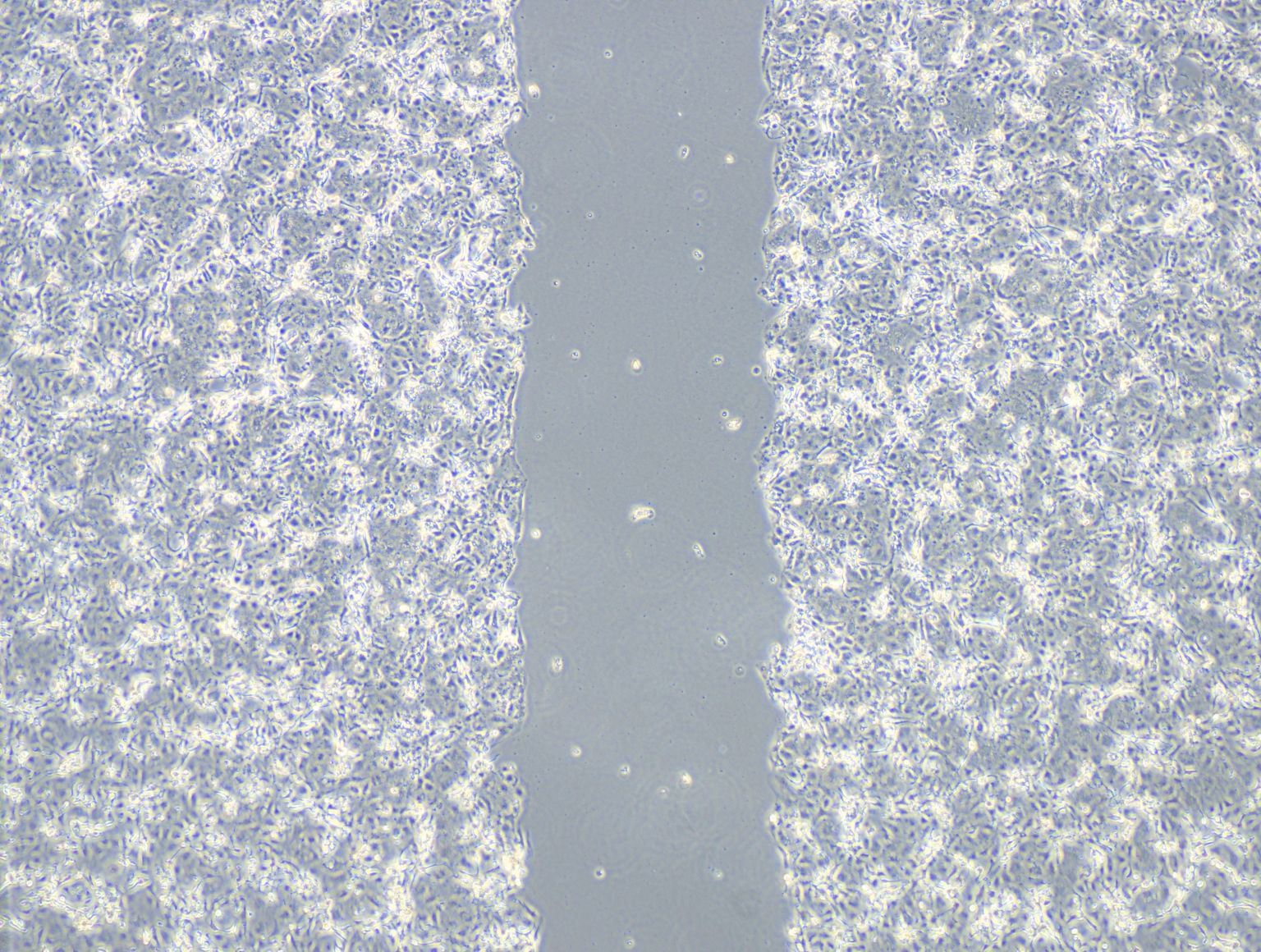

Supplement: Supplementary file 6 [file DataSheet6.zip › wound healing assay-oe-COL1A2/1-NC-24h.jpg]

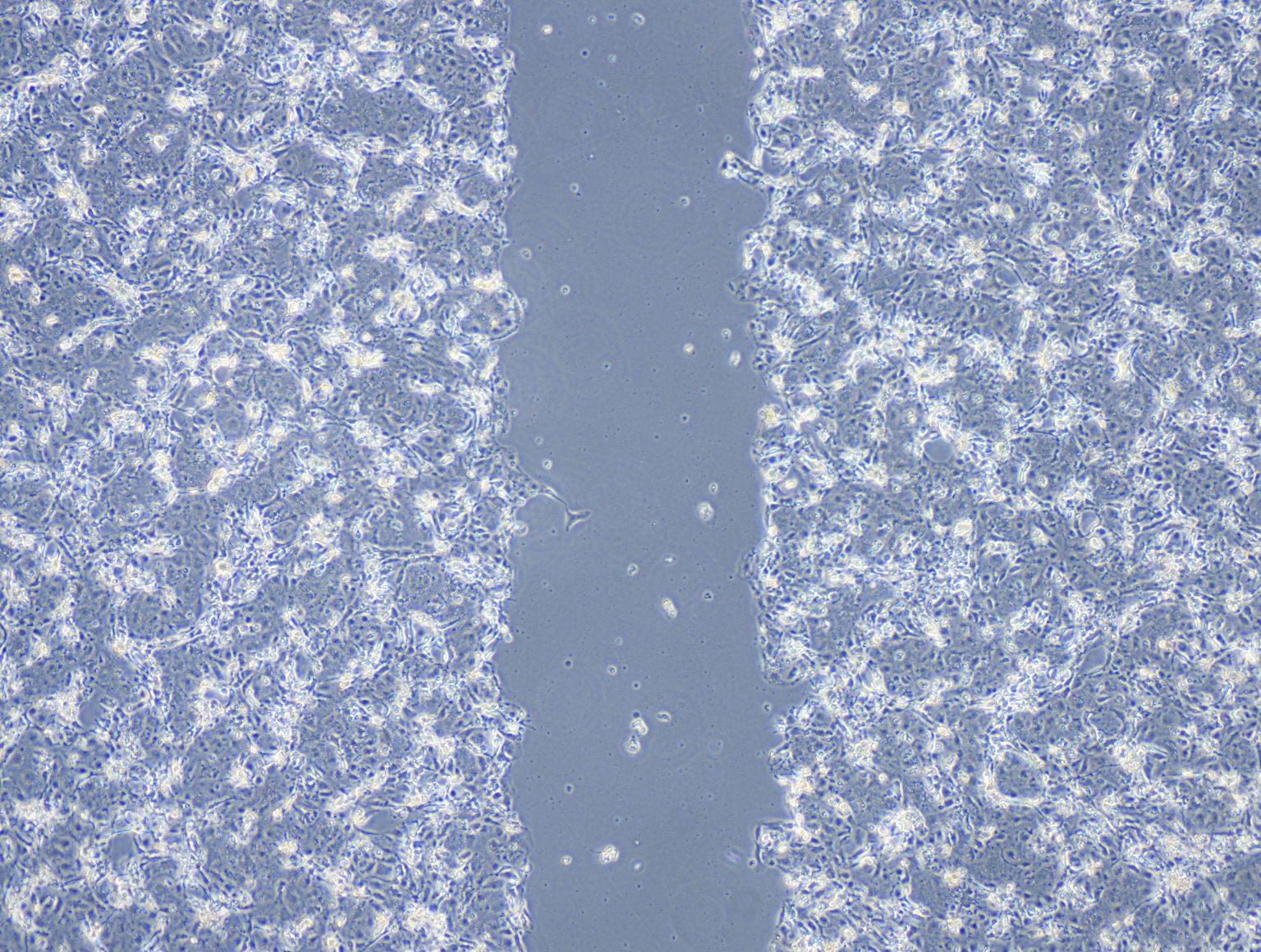

Supplement: Supplementary file 6 [file DataSheet6.zip › wound healing assay-oe-COL1A2/1-NC-48h.jpg]

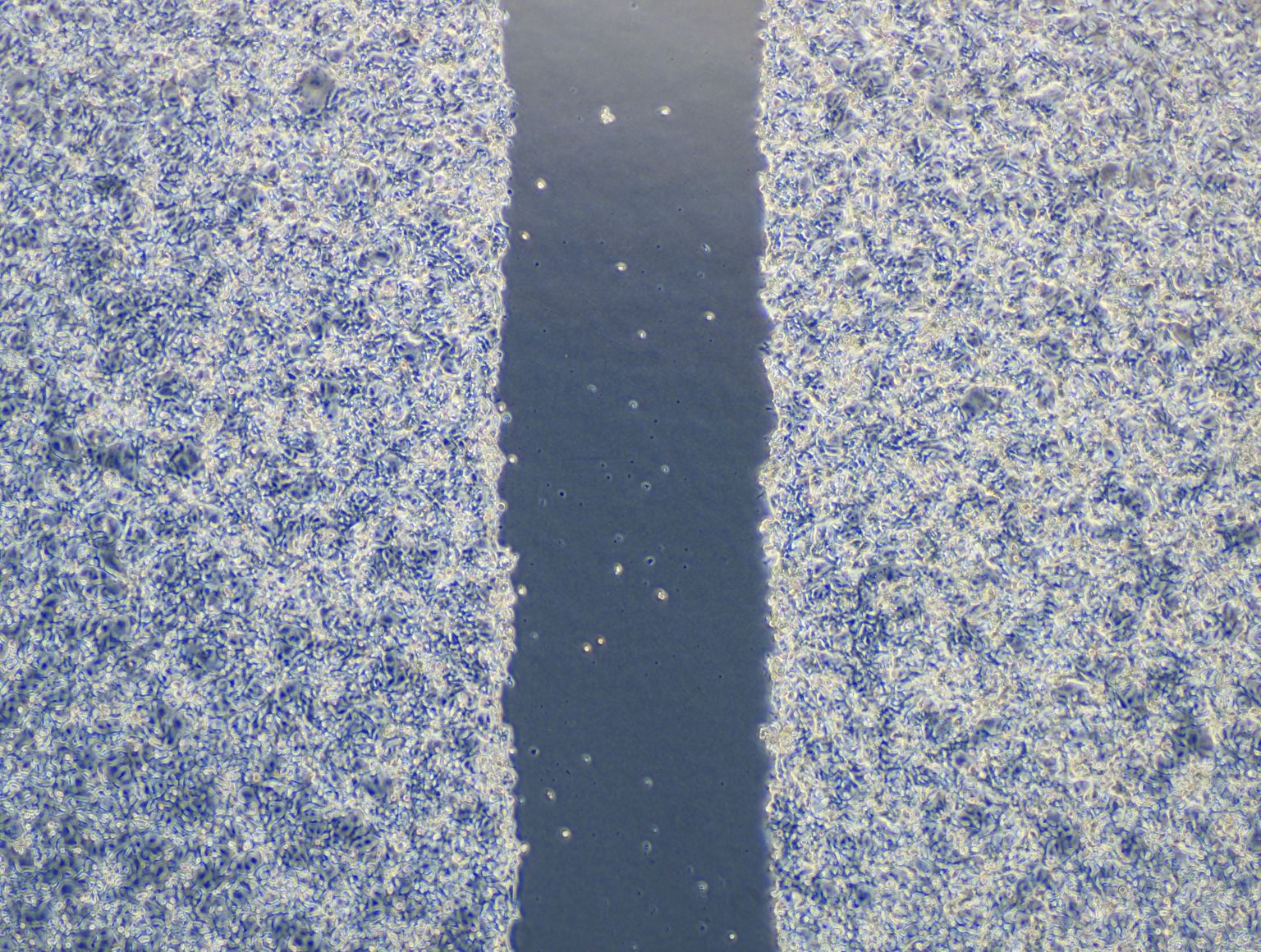

Supplement: Supplementary file 6 [file DataSheet6.zip › wound healing assay-oe-COL1A2/1-oe-0h.jpg]

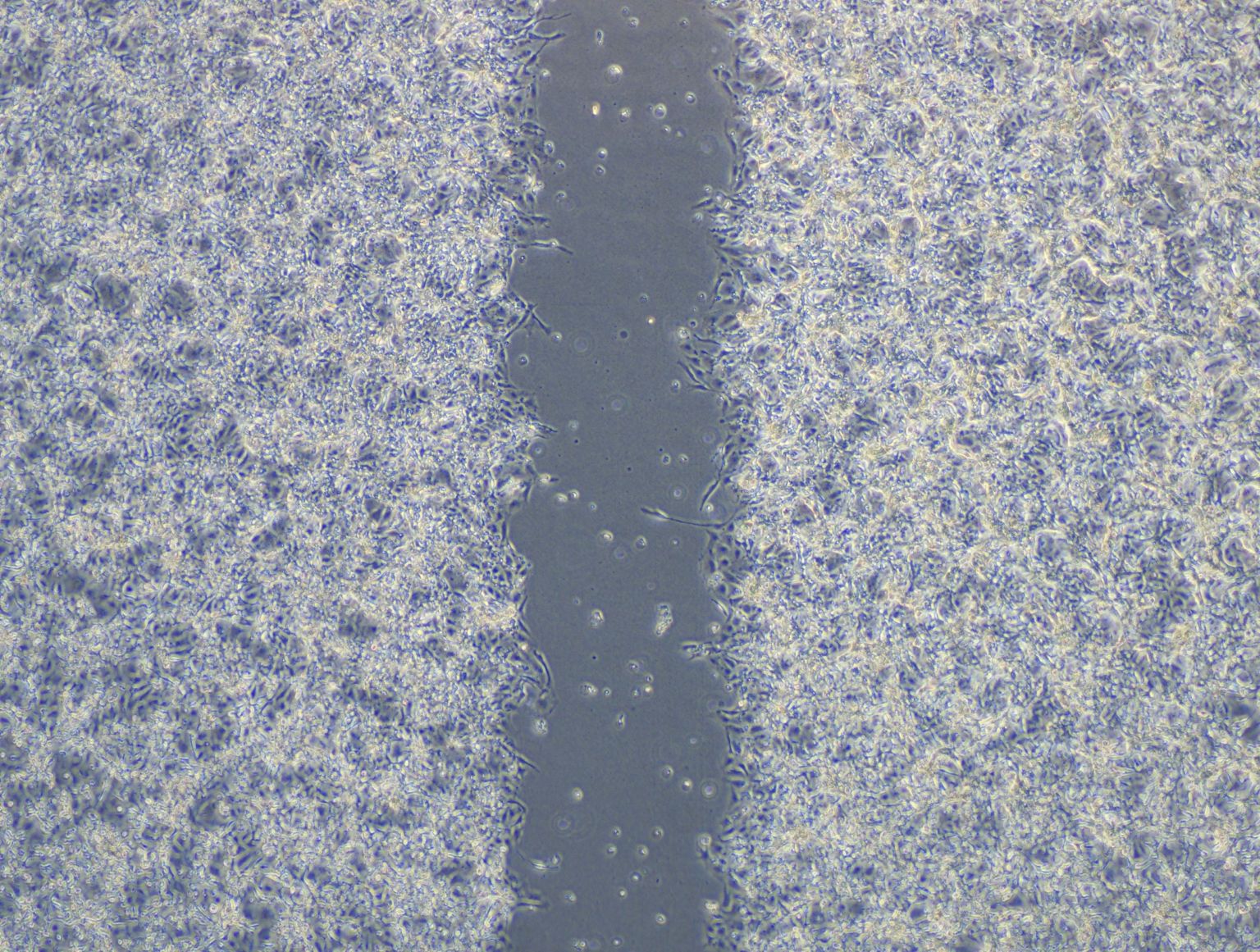

Supplement: Supplementary file 6 [file DataSheet6.zip › wound healing assay-oe-COL1A2/1-oe-24h.jpg]

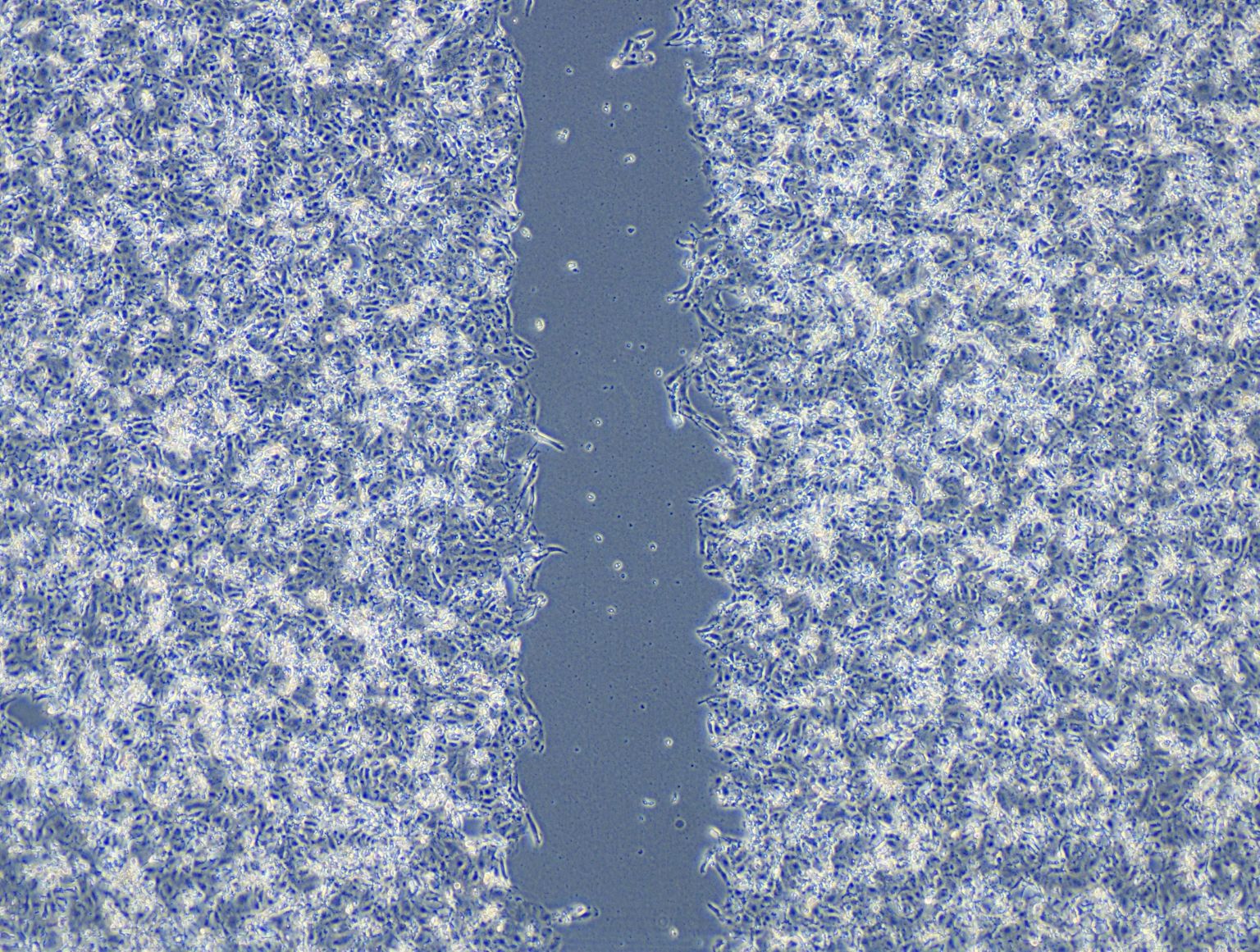

Supplement: Supplementary file 6 [file DataSheet6.zip › wound healing assay-oe-COL1A2/1-oe-48h.jpg]

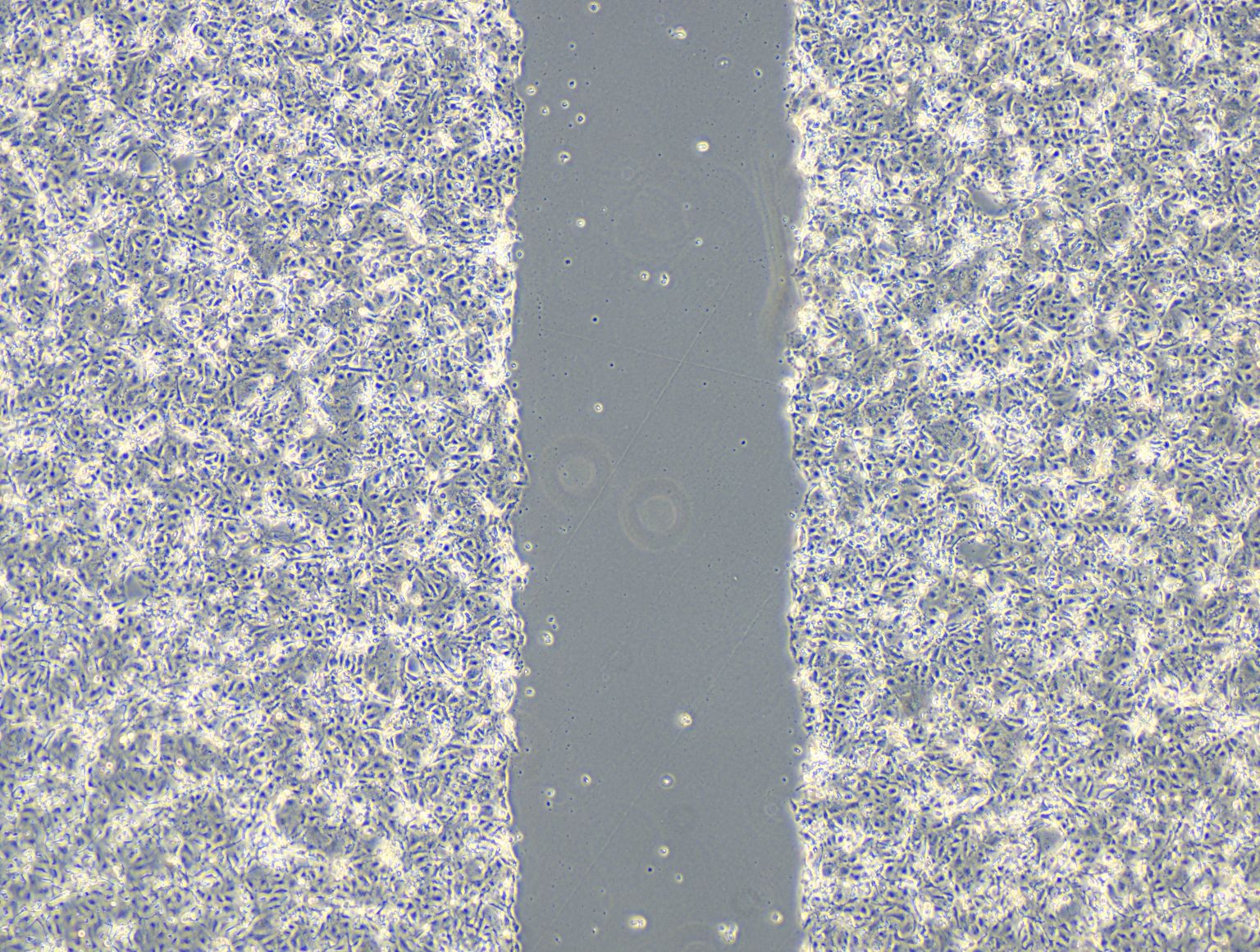

Supplement: Supplementary file 6 [file DataSheet6.zip › wound healing assay-oe-COL1A2/2-NC-0h.jpg]

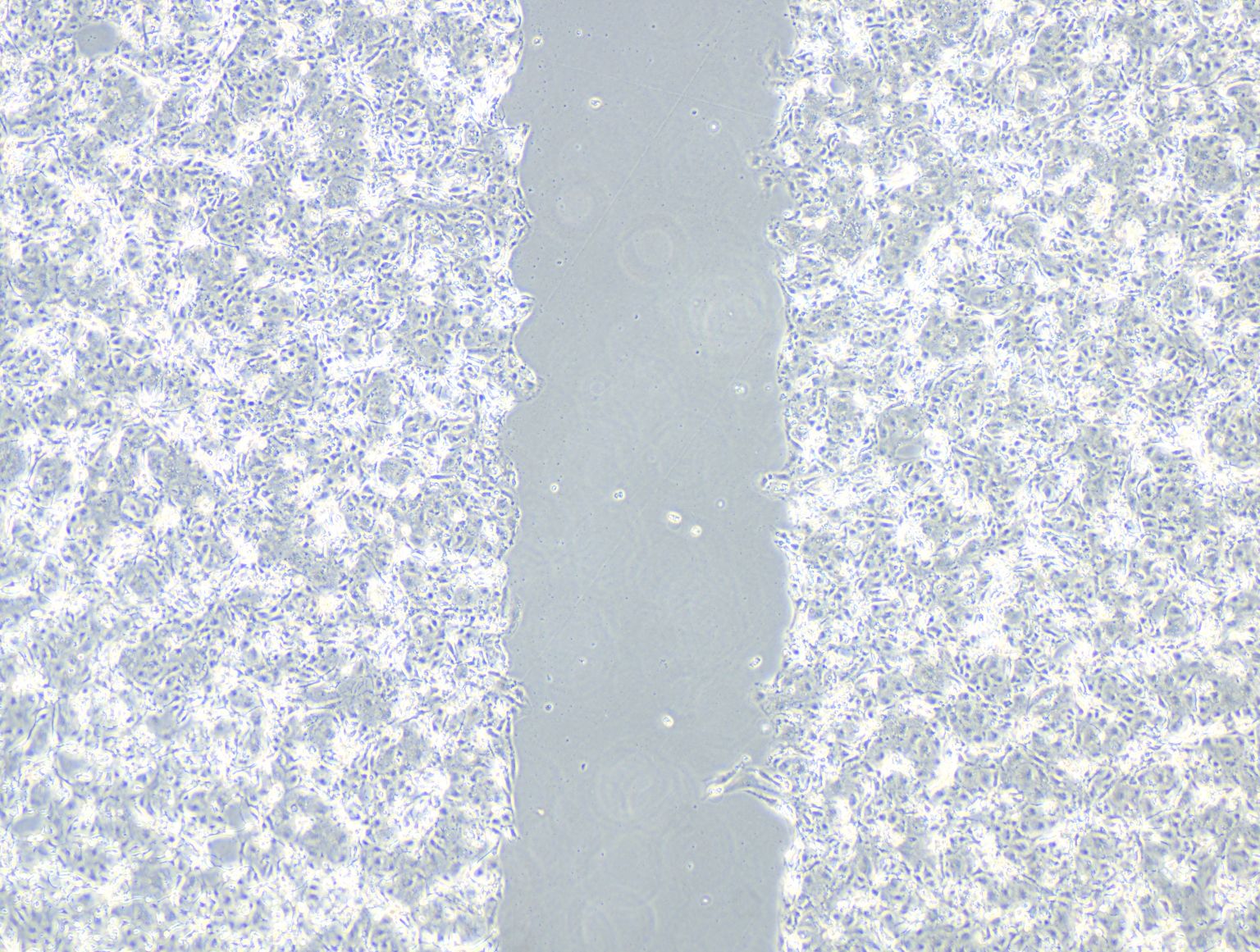

Supplement: Supplementary file 6 [file DataSheet6.zip › wound healing assay-oe-COL1A2/2-NC-24h.jpg]

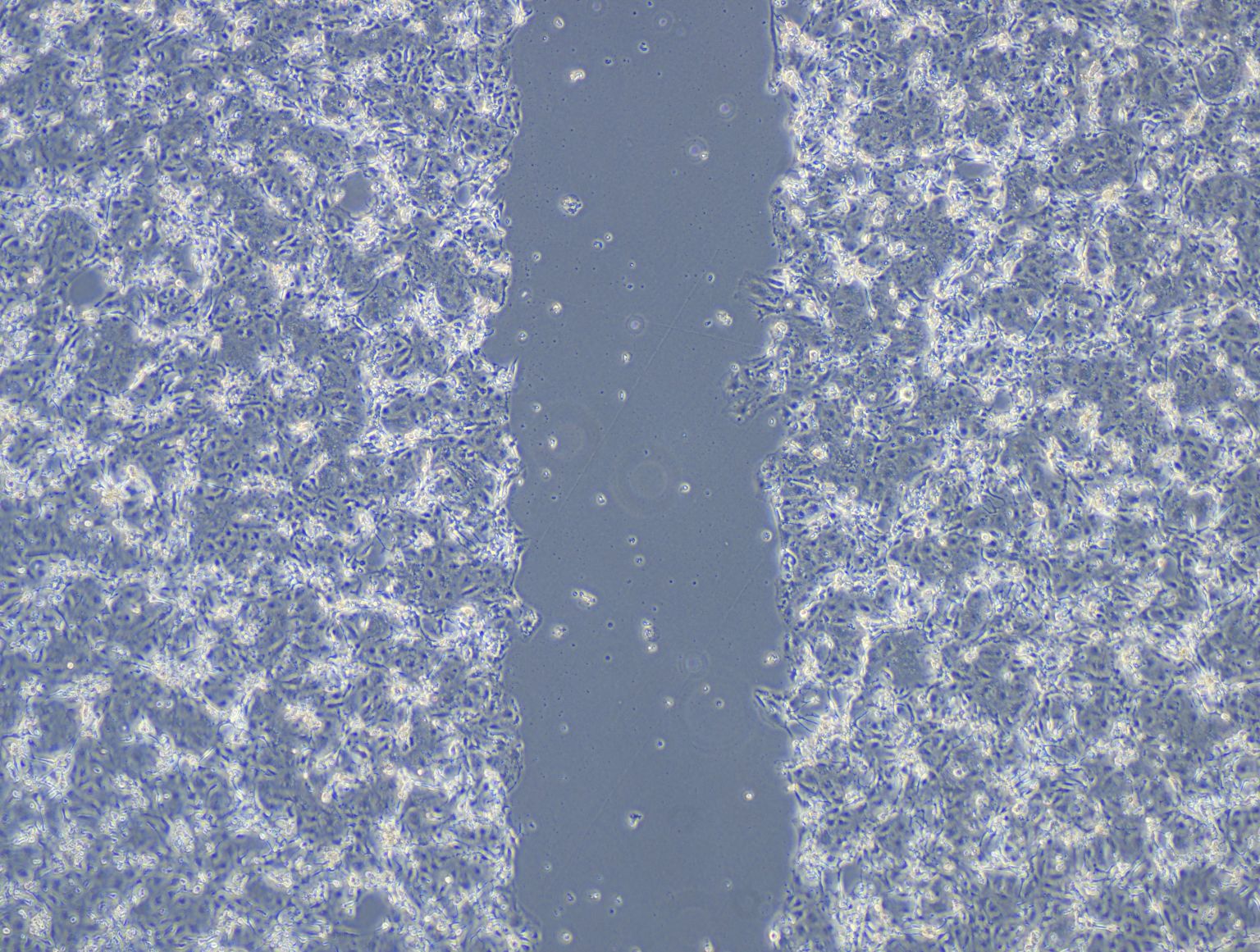

Supplement: Supplementary file 6 [file DataSheet6.zip › wound healing assay-oe-COL1A2/2-NC-48h.jpg]

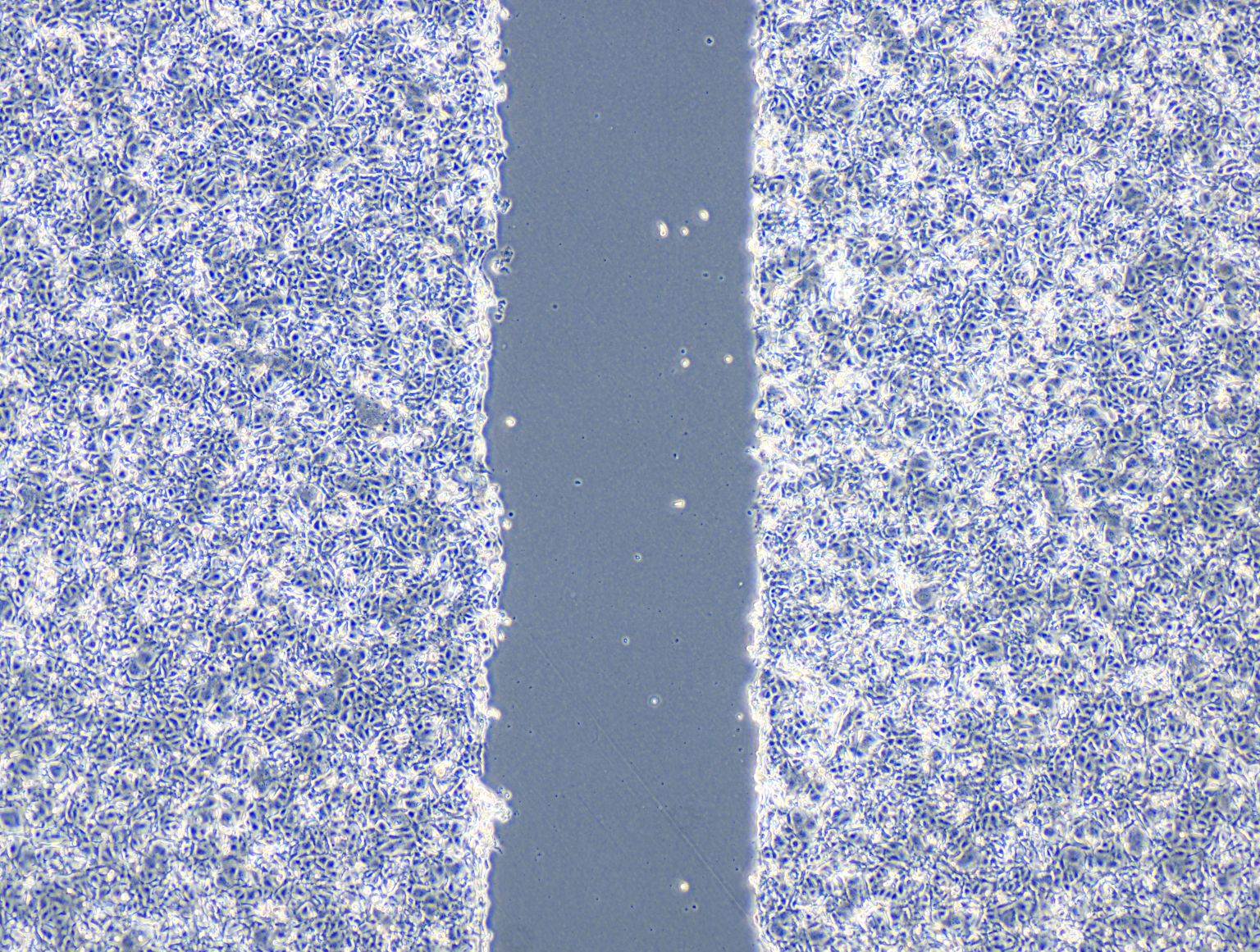

Supplement: Supplementary file 6 [file DataSheet6.zip › wound healing assay-oe-COL1A2/2-oe-0h.jpg]

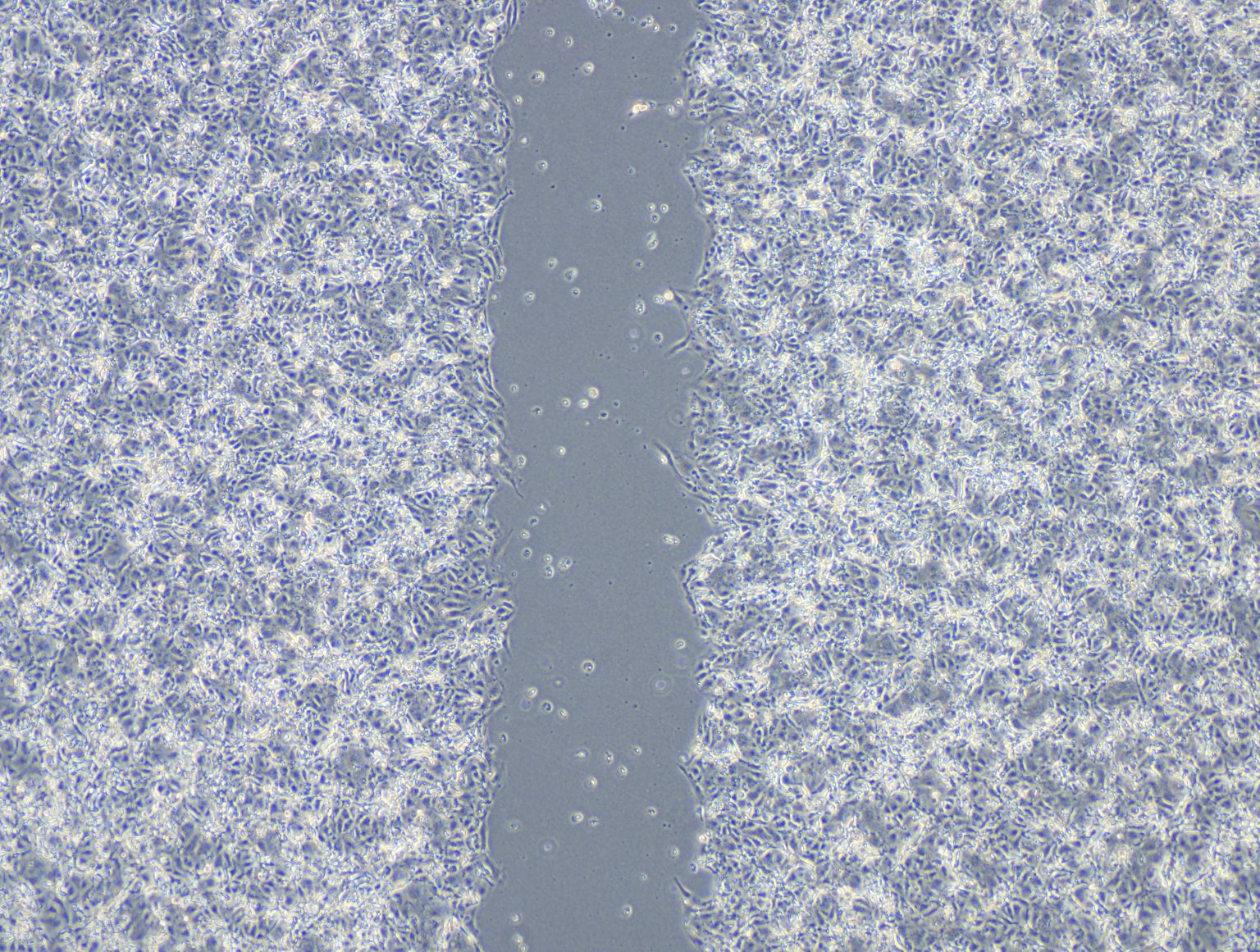

Supplement: Supplementary file 6 [file DataSheet6.zip › wound healing assay-oe-COL1A2/2-oe-24h.jpg]

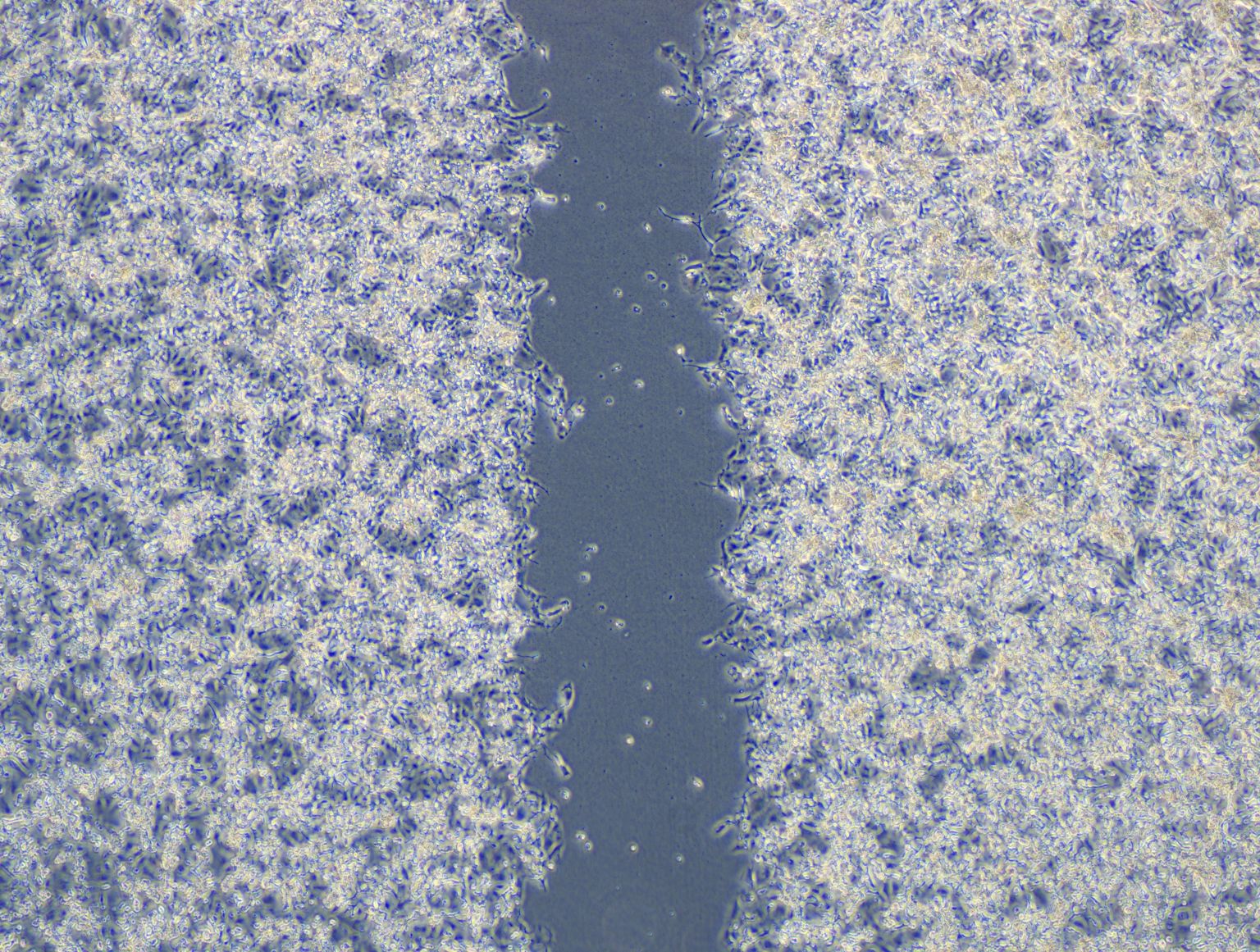

Supplement: Supplementary file 6 [file DataSheet6.zip › wound healing assay-oe-COL1A2/2-oe-48h.jpg]

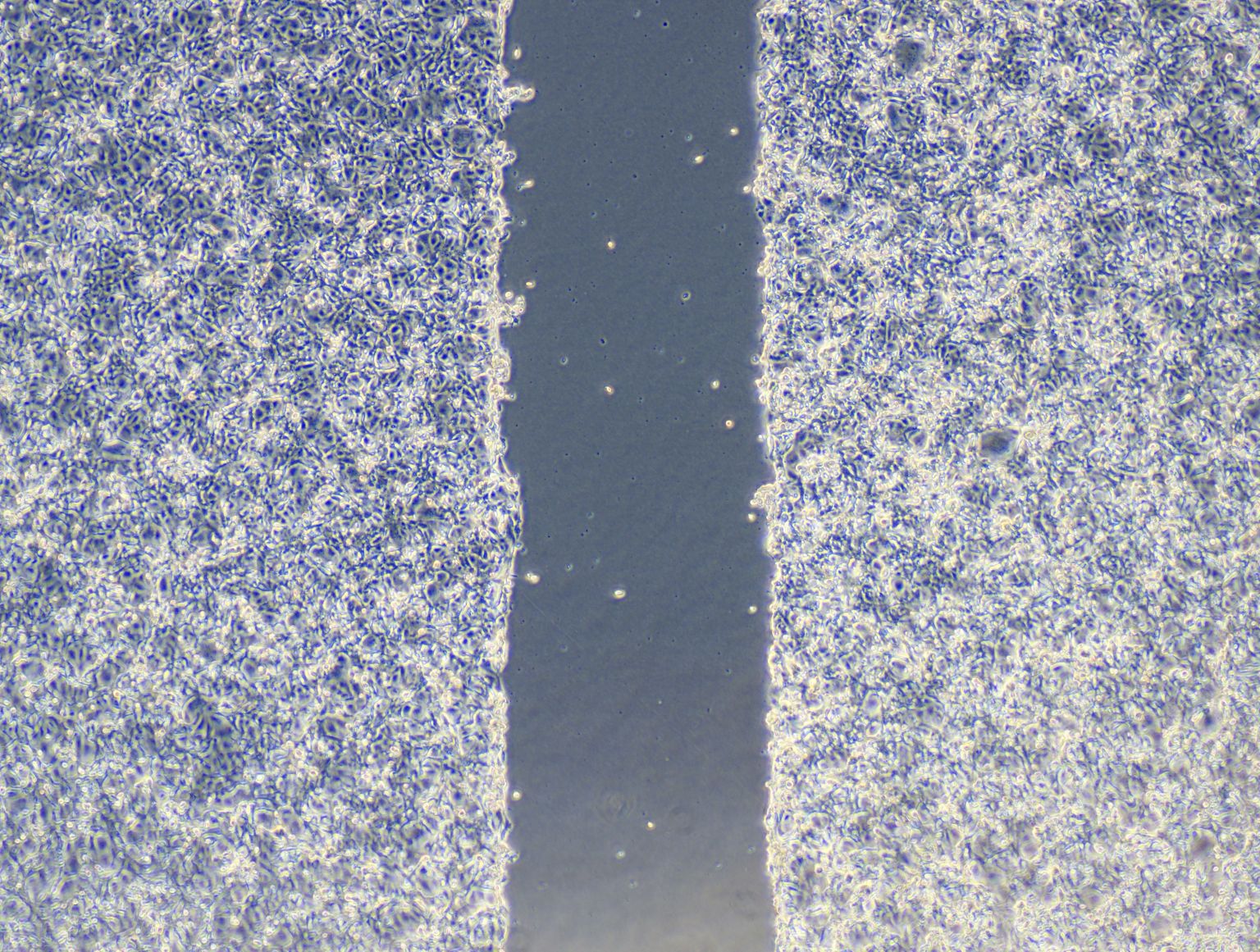

Supplement: Supplementary file 6 [file DataSheet6.zip › wound healing assay-oe-COL1A2/3-NC-0h.jpg]

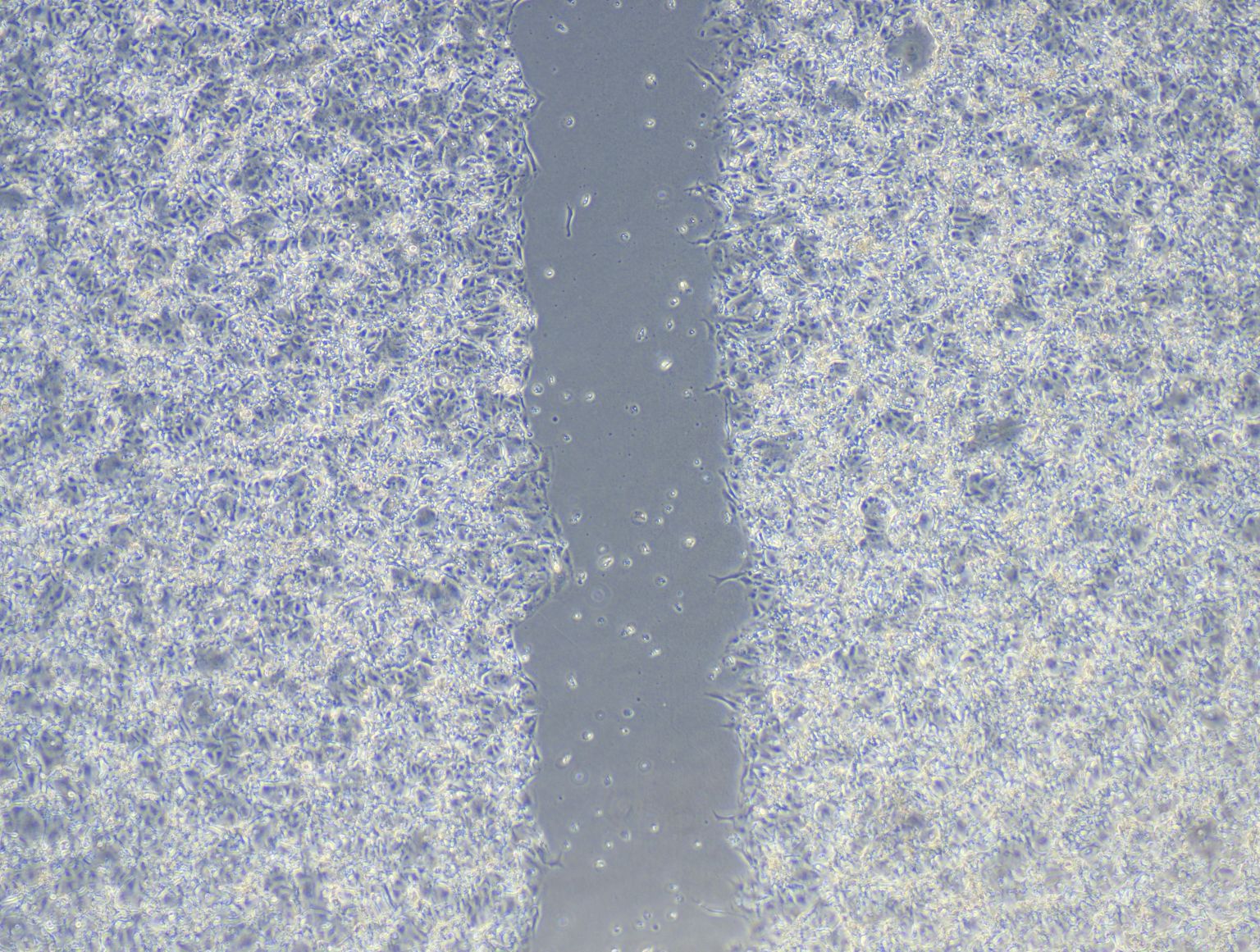

Supplement: Supplementary file 6 [file DataSheet6.zip › wound healing assay-oe-COL1A2/3-NC-24h.jpg]

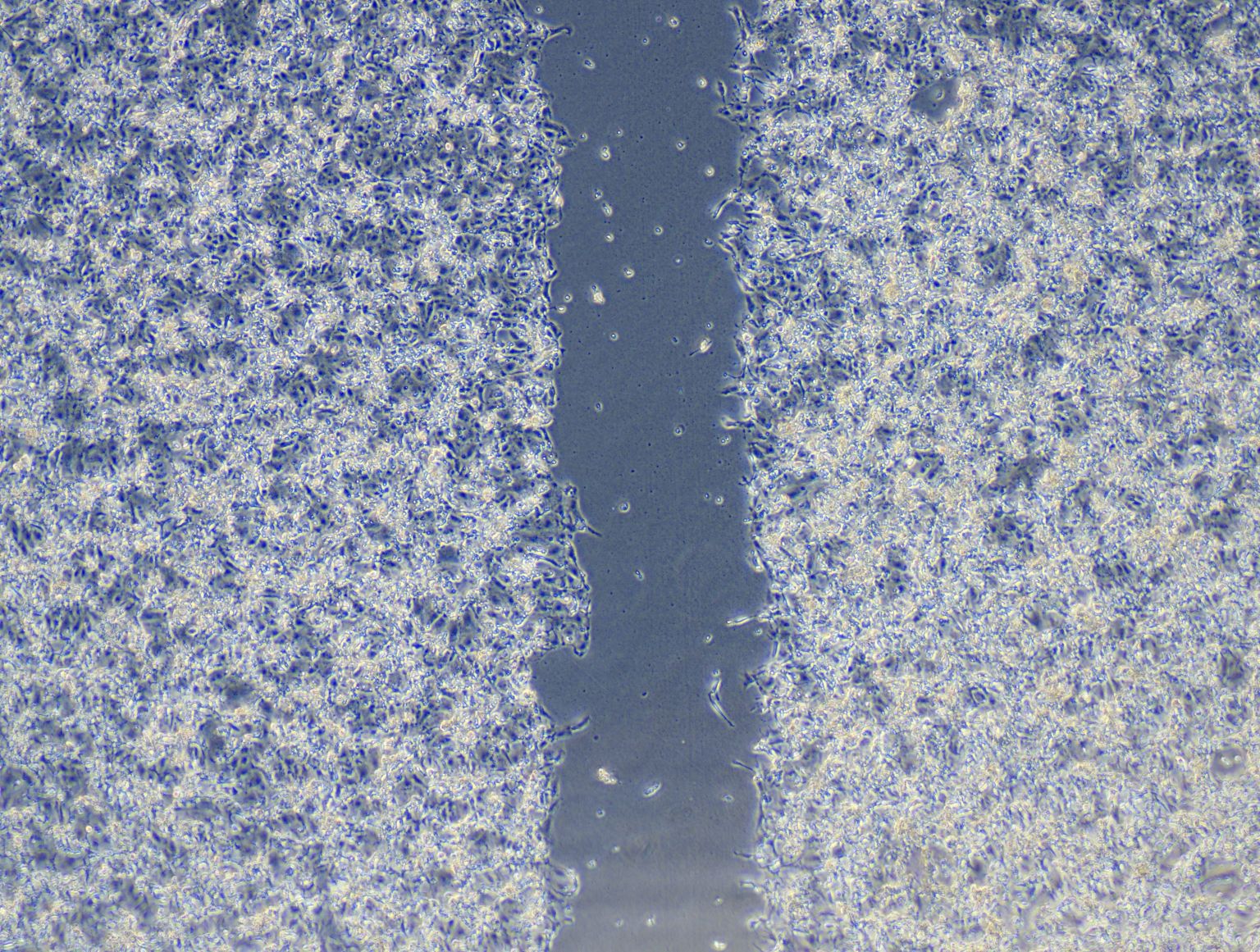

Supplement: Supplementary file 6 [file DataSheet6.zip › wound healing assay-oe-COL1A2/3-NC-48h.jpg]

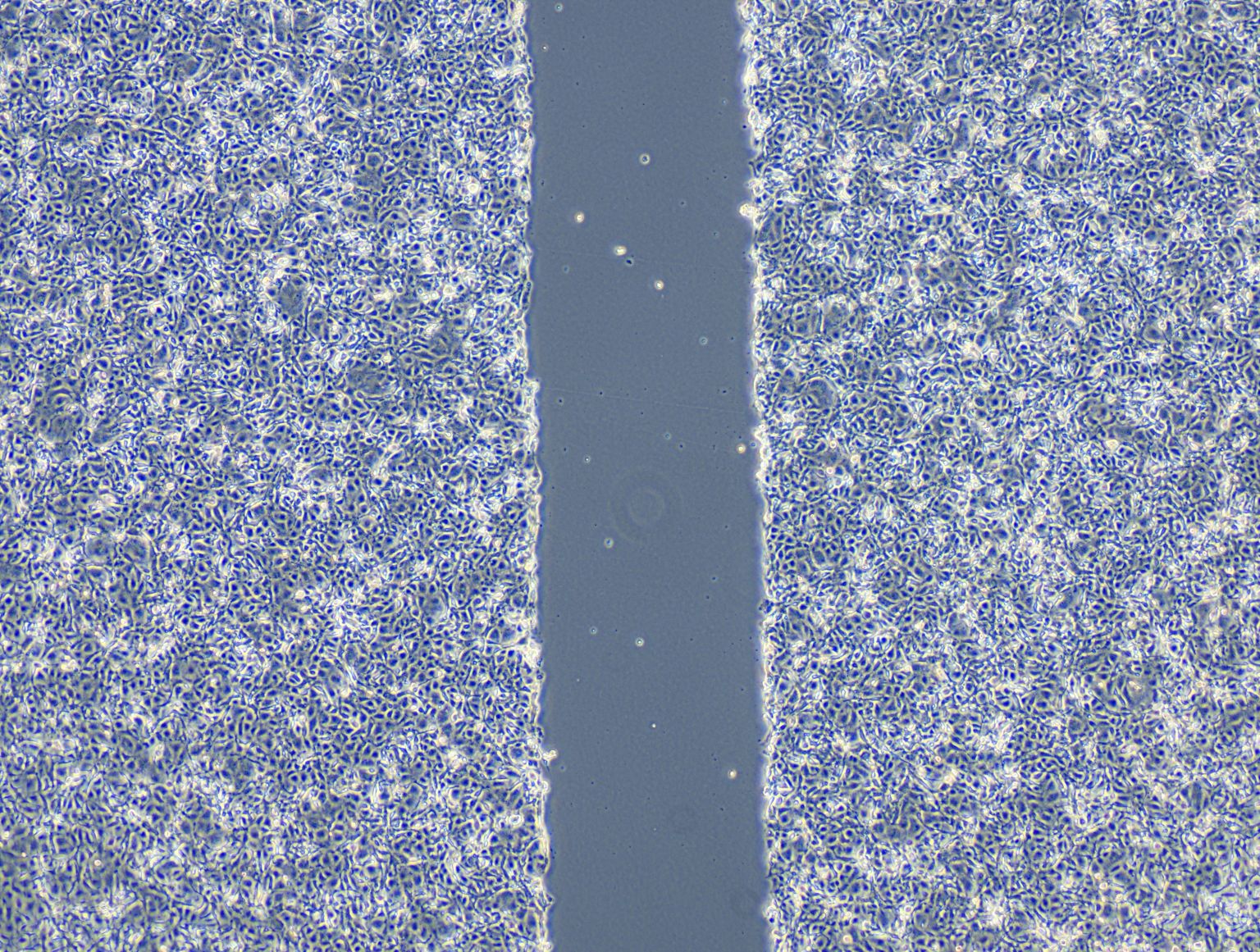

Supplement: Supplementary file 6 [file DataSheet6.zip › wound healing assay-oe-COL1A2/3-oe-0h.jpg]

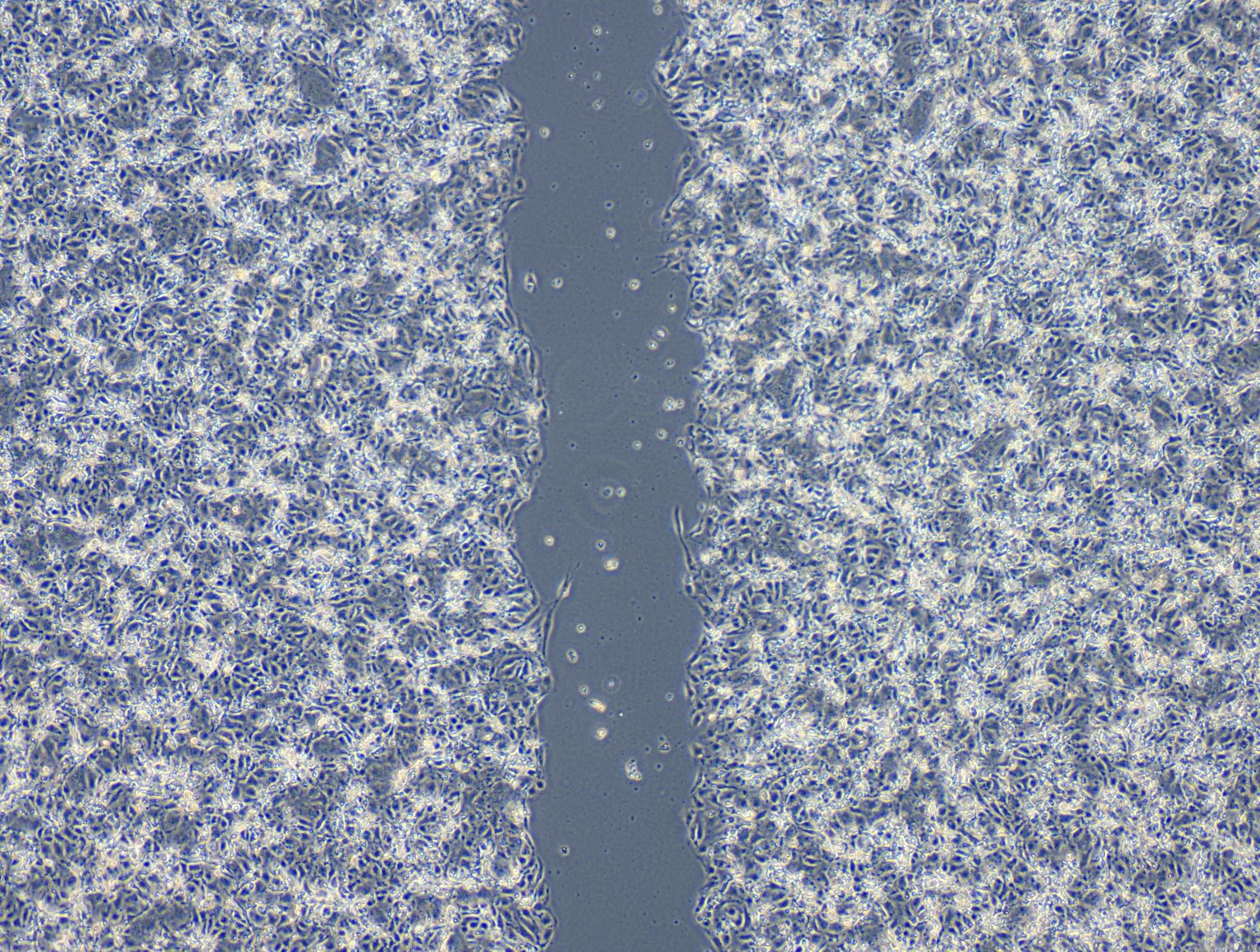

Supplement: Supplementary file 6 [file DataSheet6.zip › wound healing assay-oe-COL1A2/3-oe-24h.jpg]

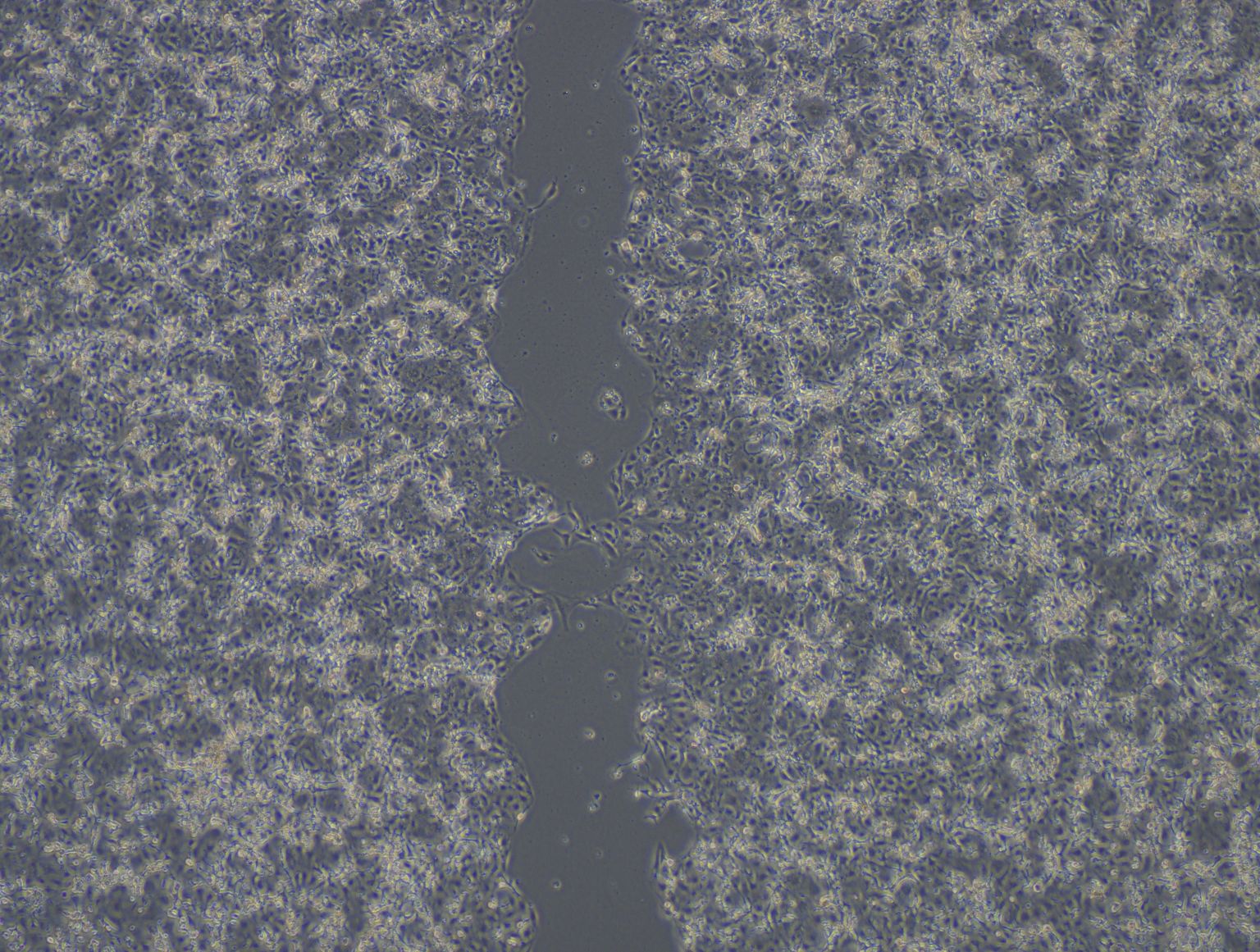

Supplement: Supplementary file 6 [file DataSheet6.zip › wound healing assay-oe-COL1A2/3-oe-48h.jpg]

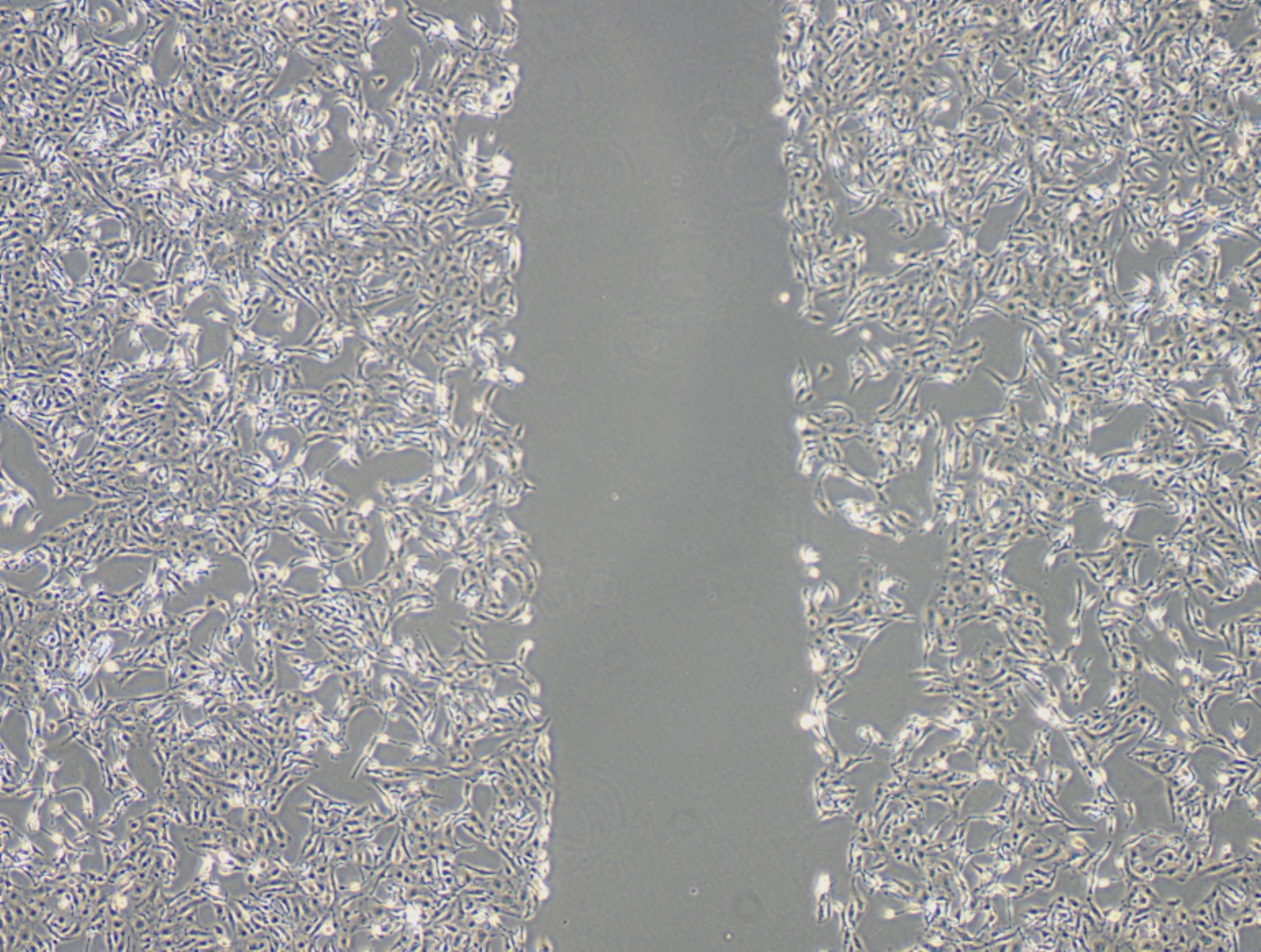

Supplement: Supplementary file 7 [file DataSheet7.zip › wound healing assay-PF-573228/1-NC-0h.jpg]

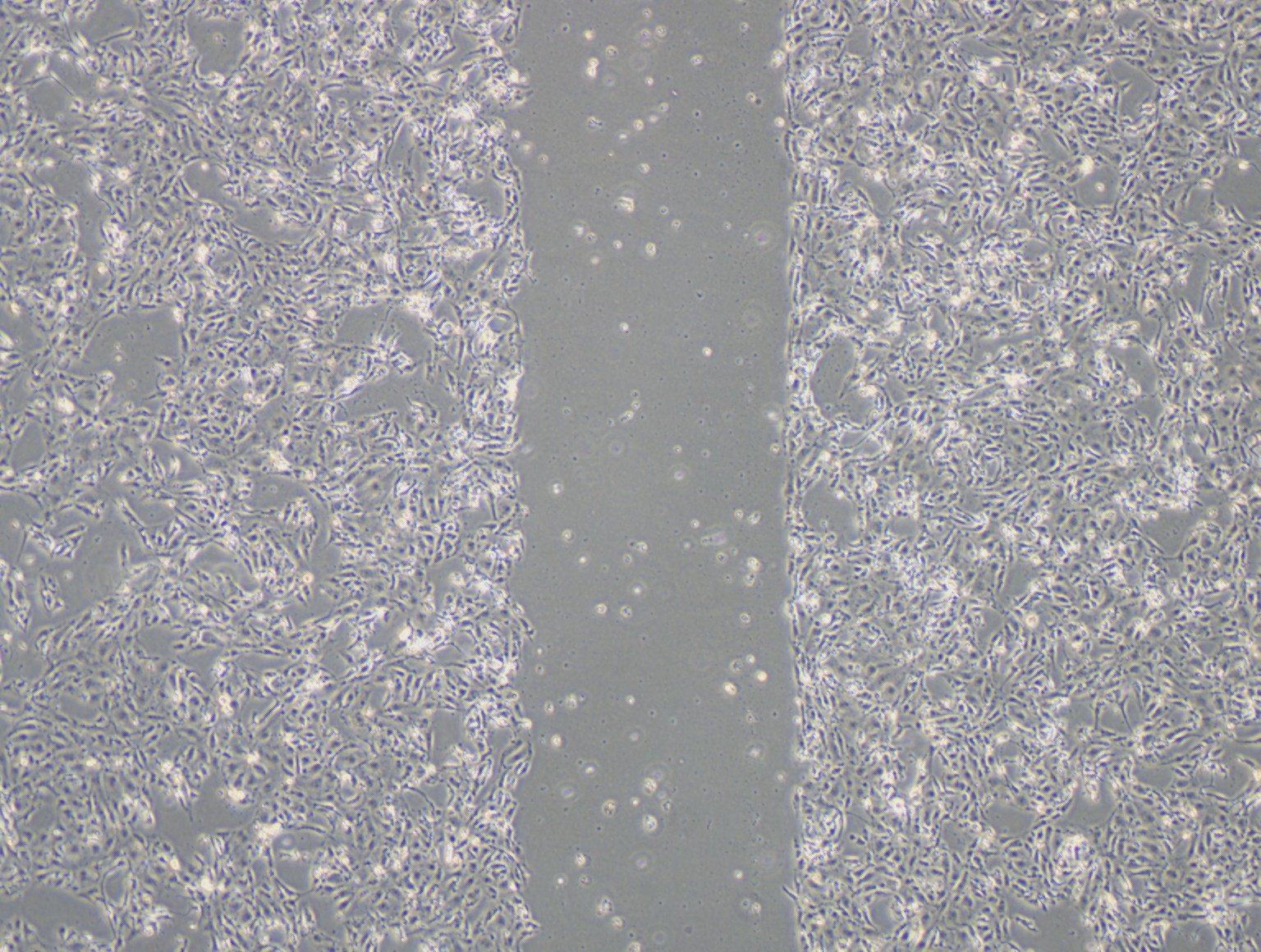

Supplement: Supplementary file 7 [file DataSheet7.zip › wound healing assay-PF-573228/1-NC-24h.jpg]

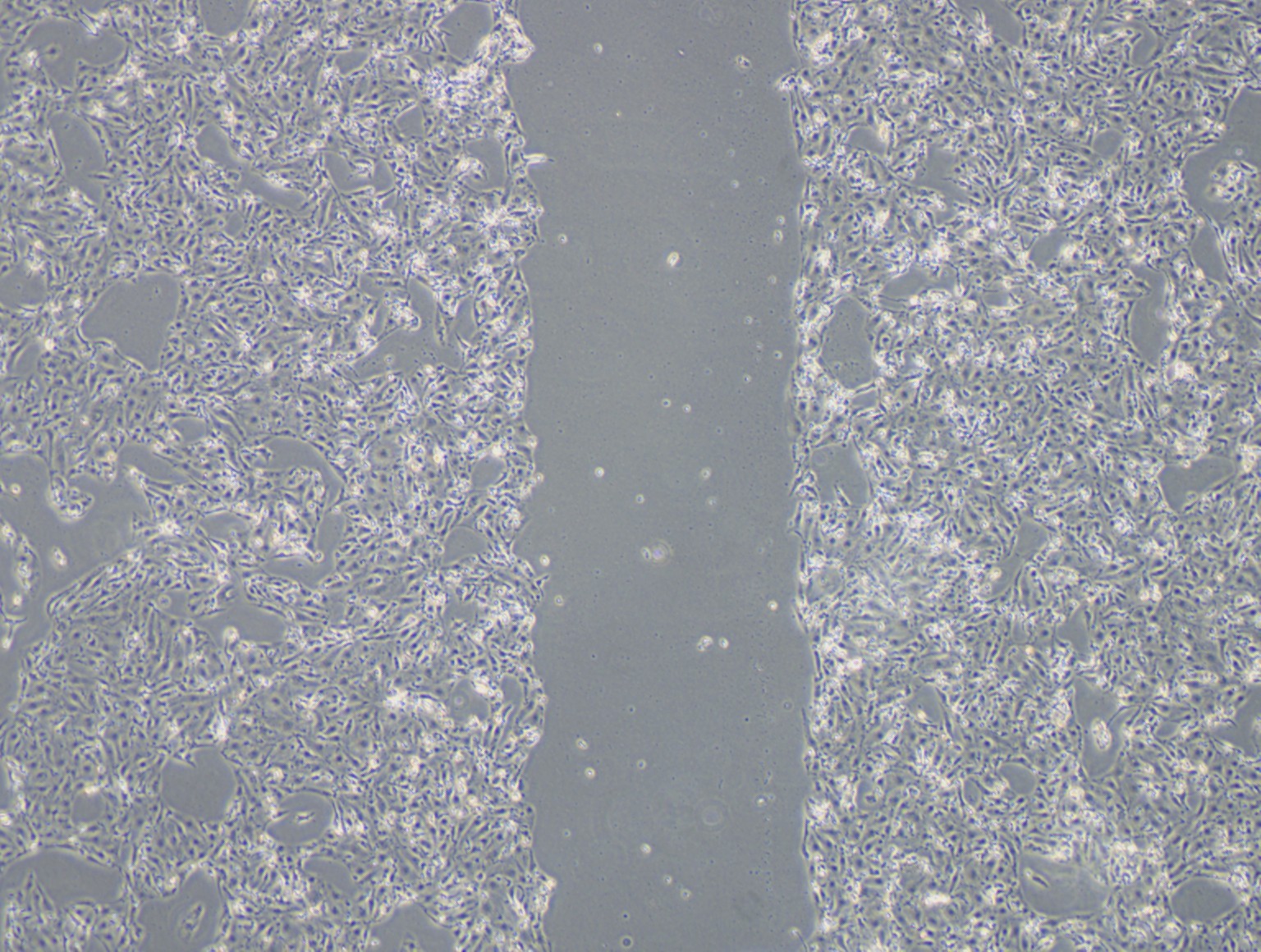

Supplement: Supplementary file 7 [file DataSheet7.zip › wound healing assay-PF-573228/1-NC-48h.jpg]

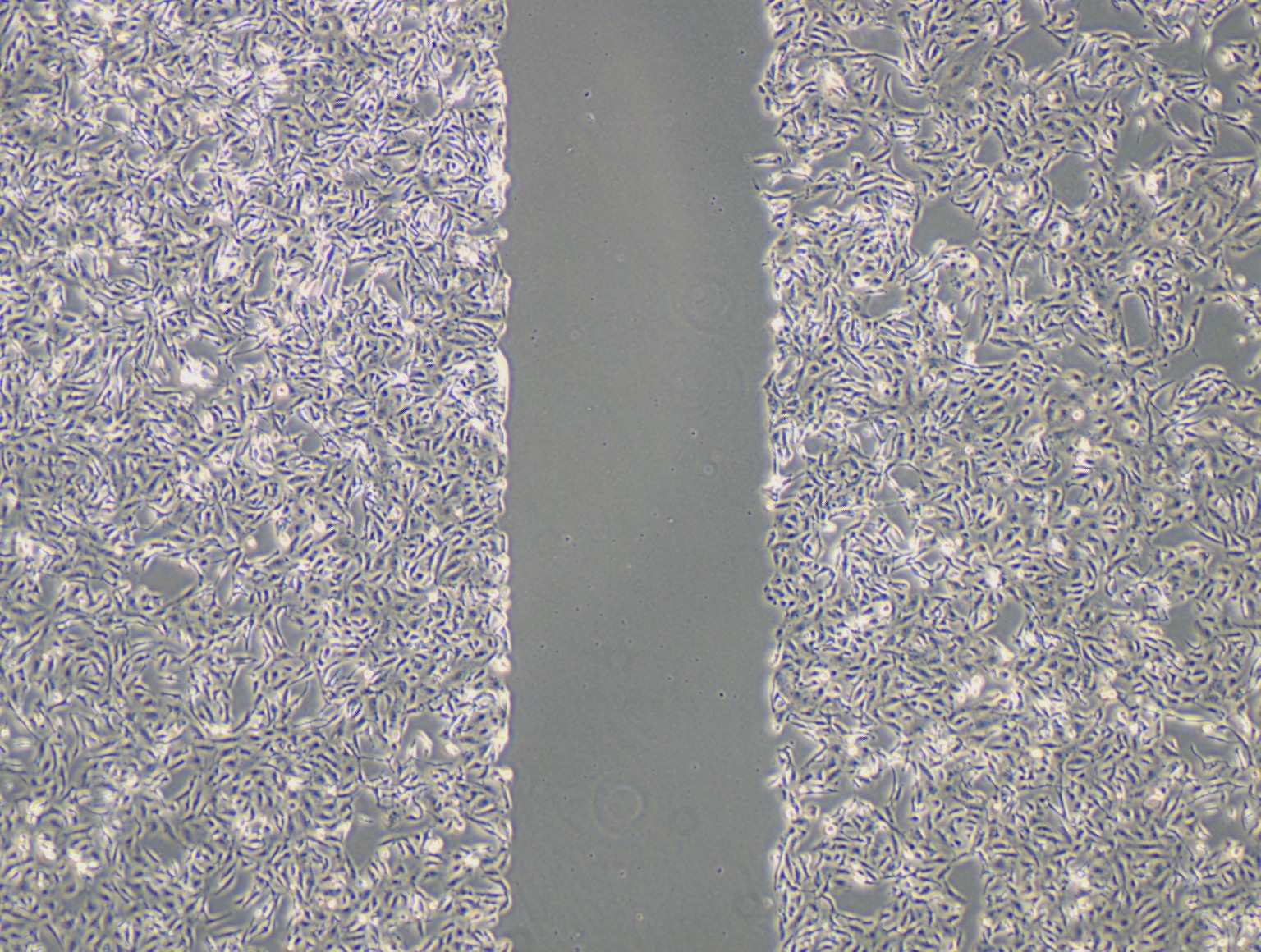

Supplement: Supplementary file 7 [file DataSheet7.zip › wound healing assay-PF-573228/1-oe-0h.jpg]

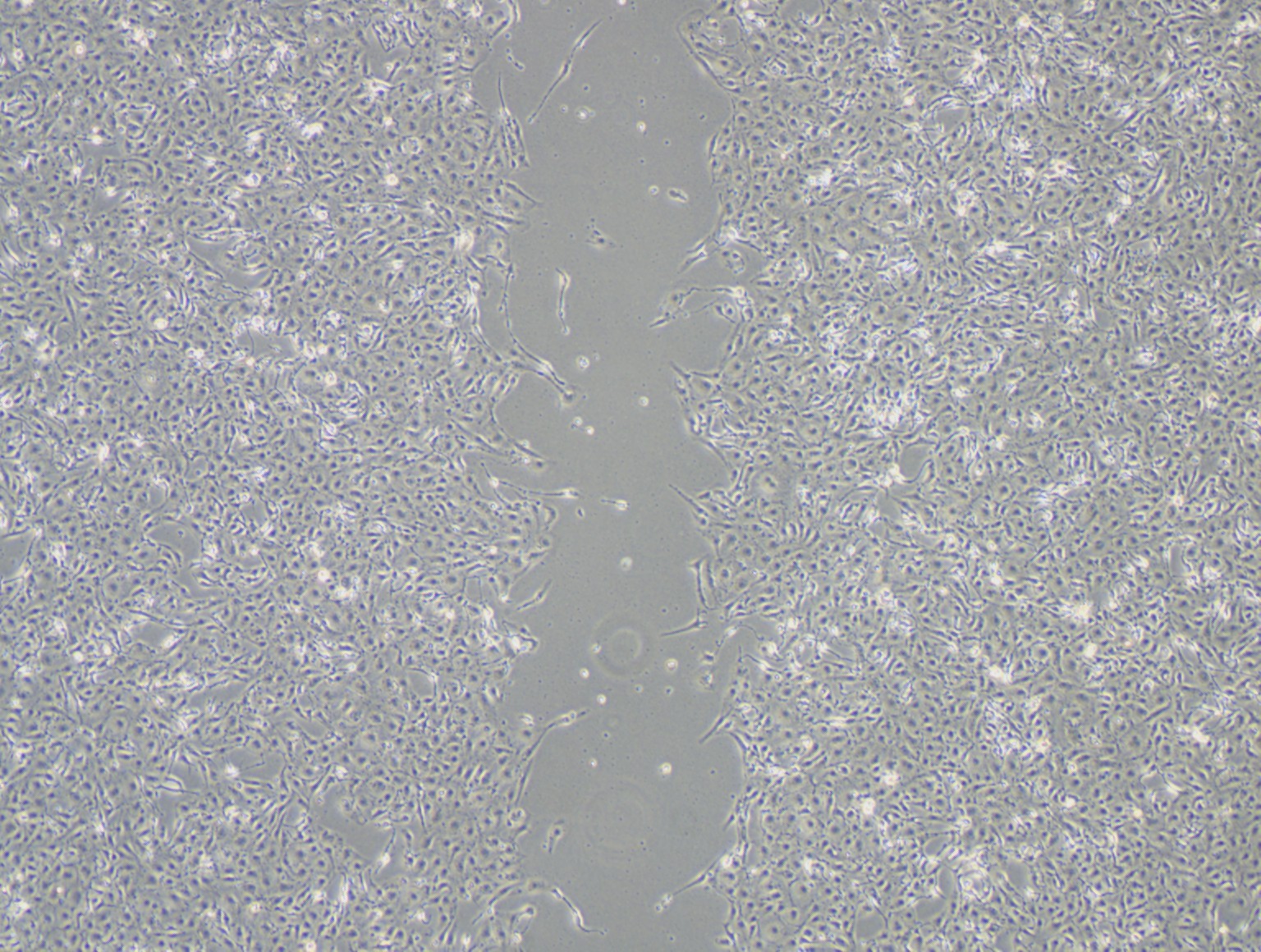

Supplement: Supplementary file 7 [file DataSheet7.zip › wound healing assay-PF-573228/1-oe-24h.jpg]

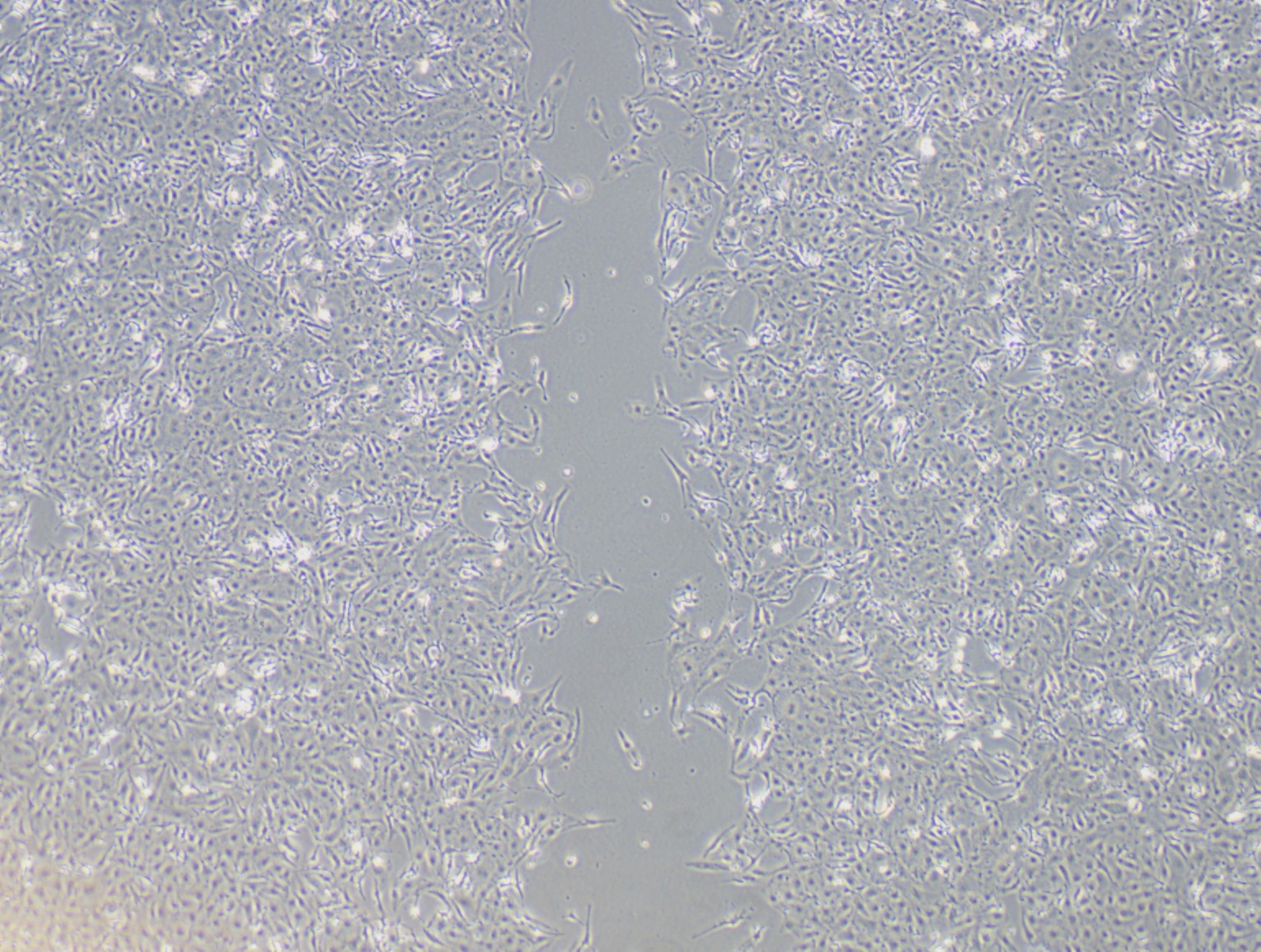

Supplement: Supplementary file 7 [file DataSheet7.zip › wound healing assay-PF-573228/1-oe-48h.jpg]

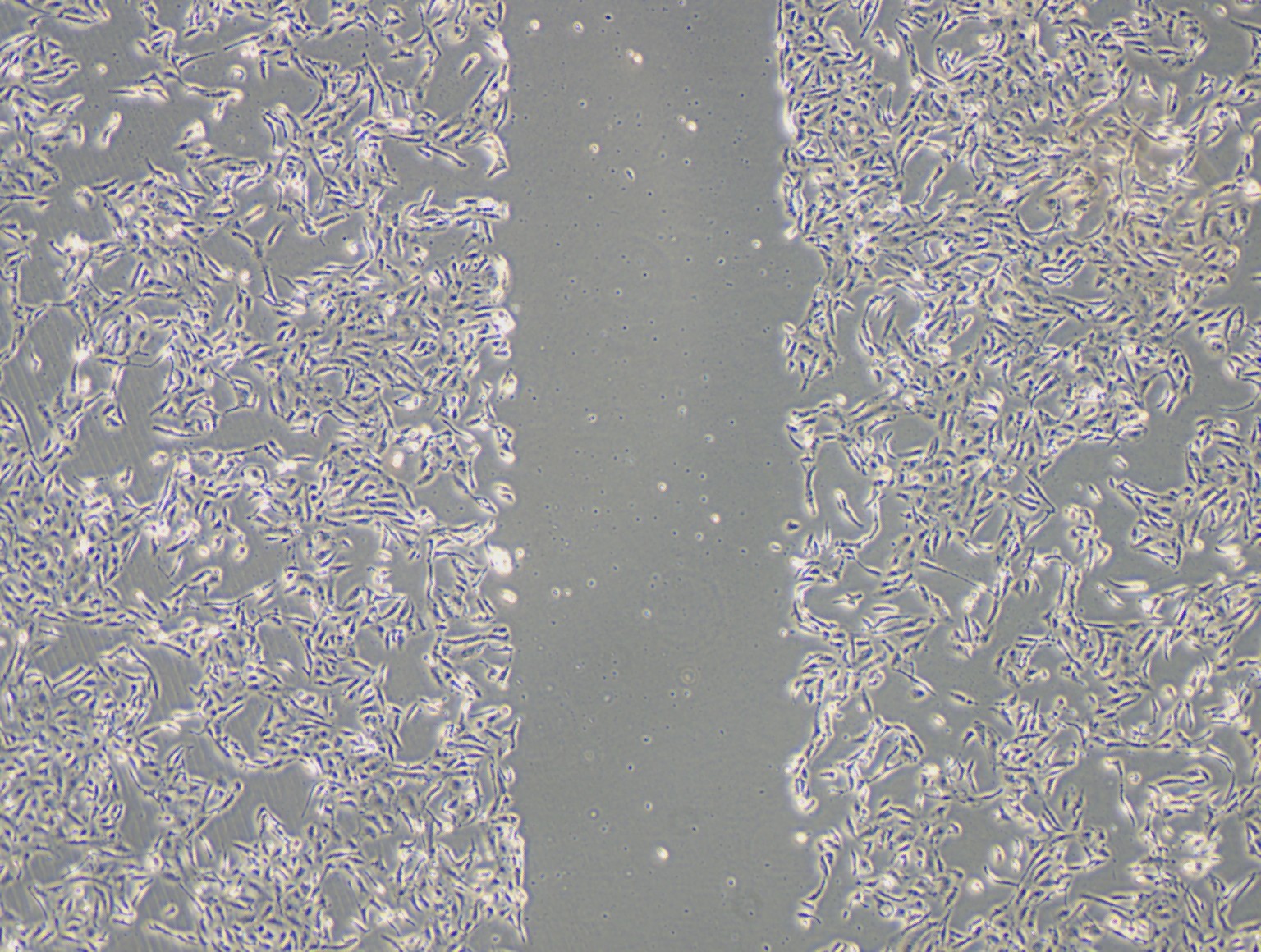

Supplement: Supplementary file 7 [file DataSheet7.zip › wound healing assay-PF-573228/1-PF-573228-0h.jpg]

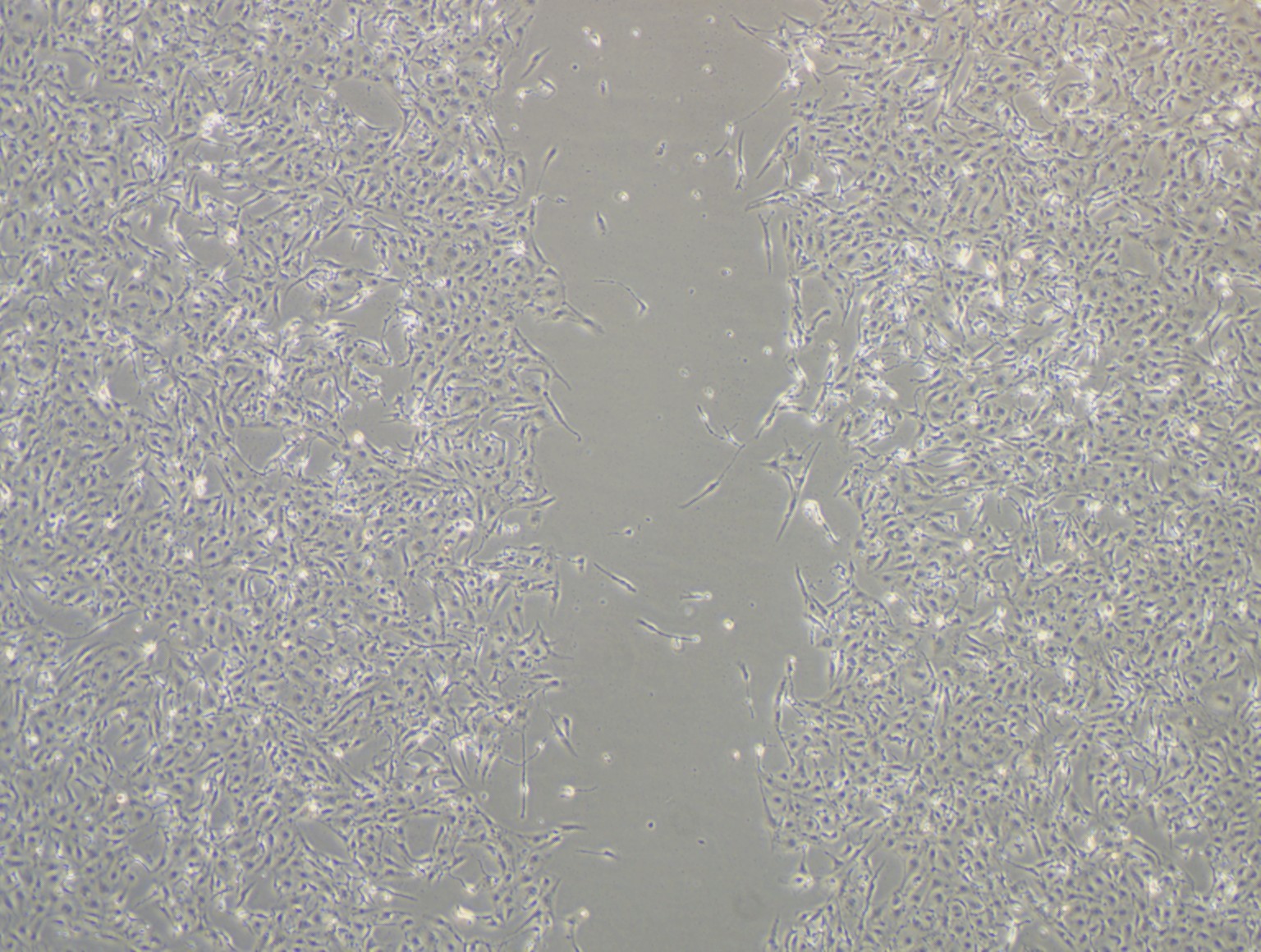

Supplement: Supplementary file 7 [file DataSheet7.zip › wound healing assay-PF-573228/1-PF-573228-24h.jpg]

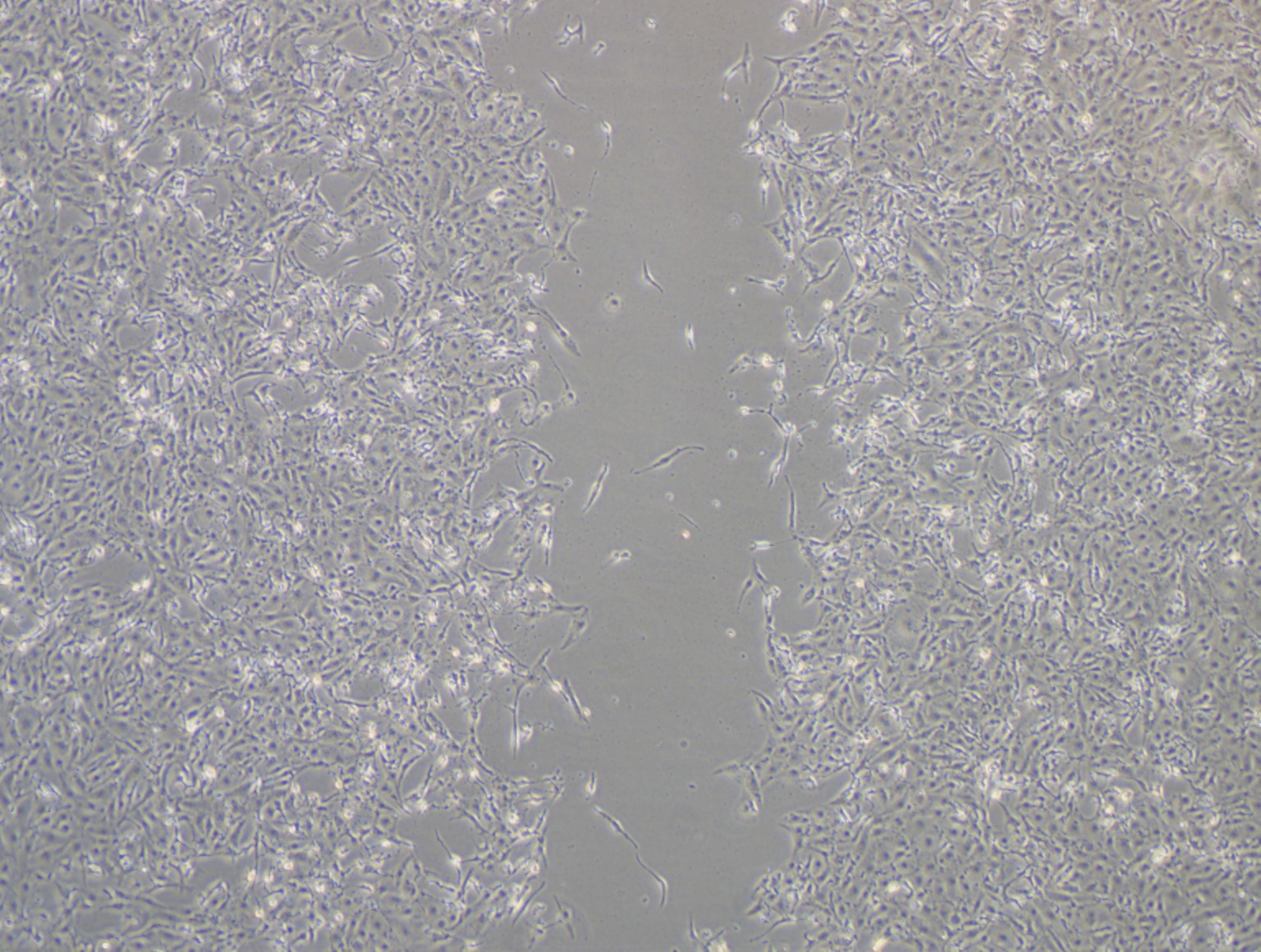

Supplement: Supplementary file 7 [file DataSheet7.zip › wound healing assay-PF-573228/1-PF-573228-48h.jpg]

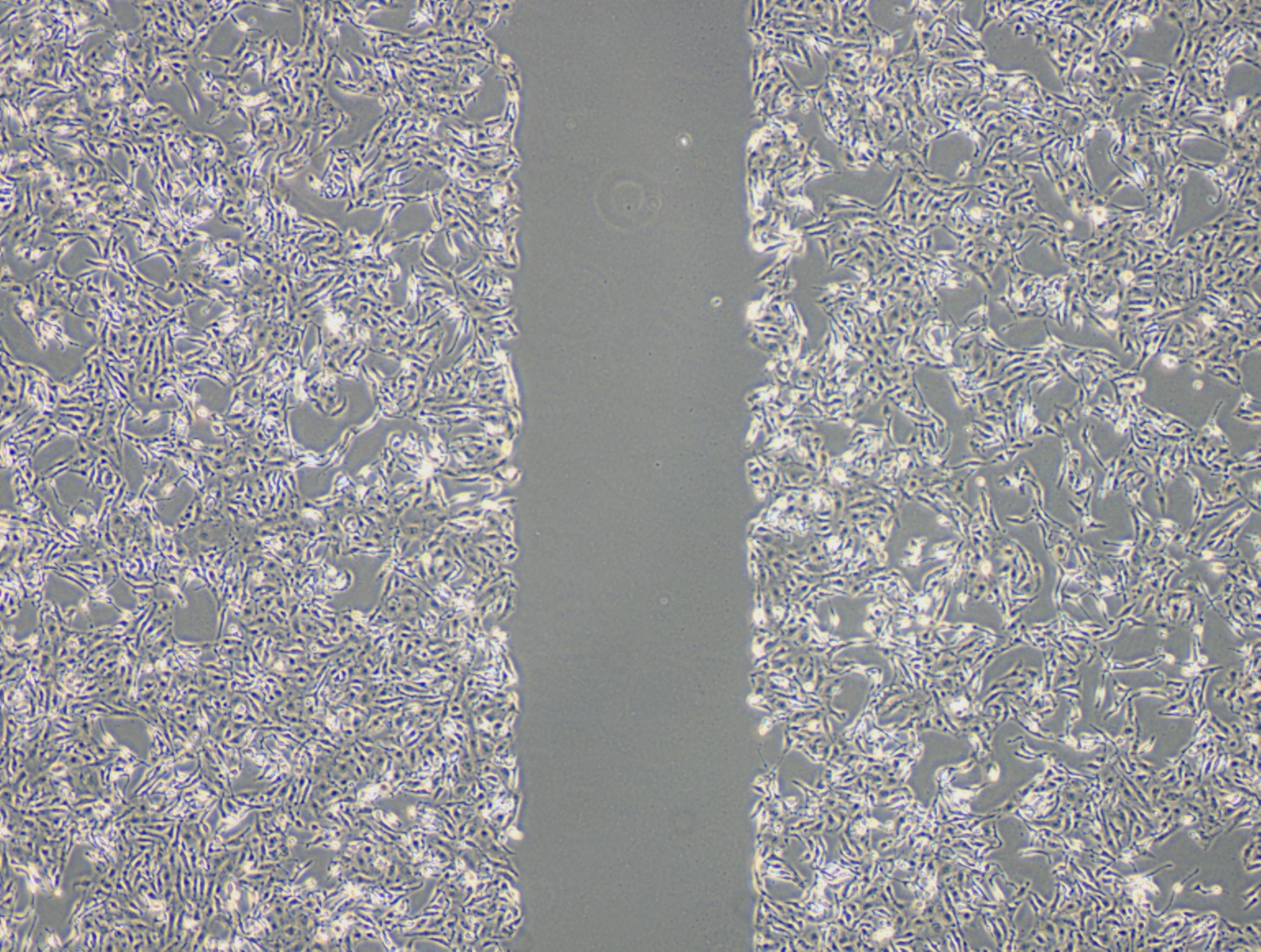

Supplement: Supplementary file 7 [file DataSheet7.zip › wound healing assay-PF-573228/2-NC-0h.jpg]

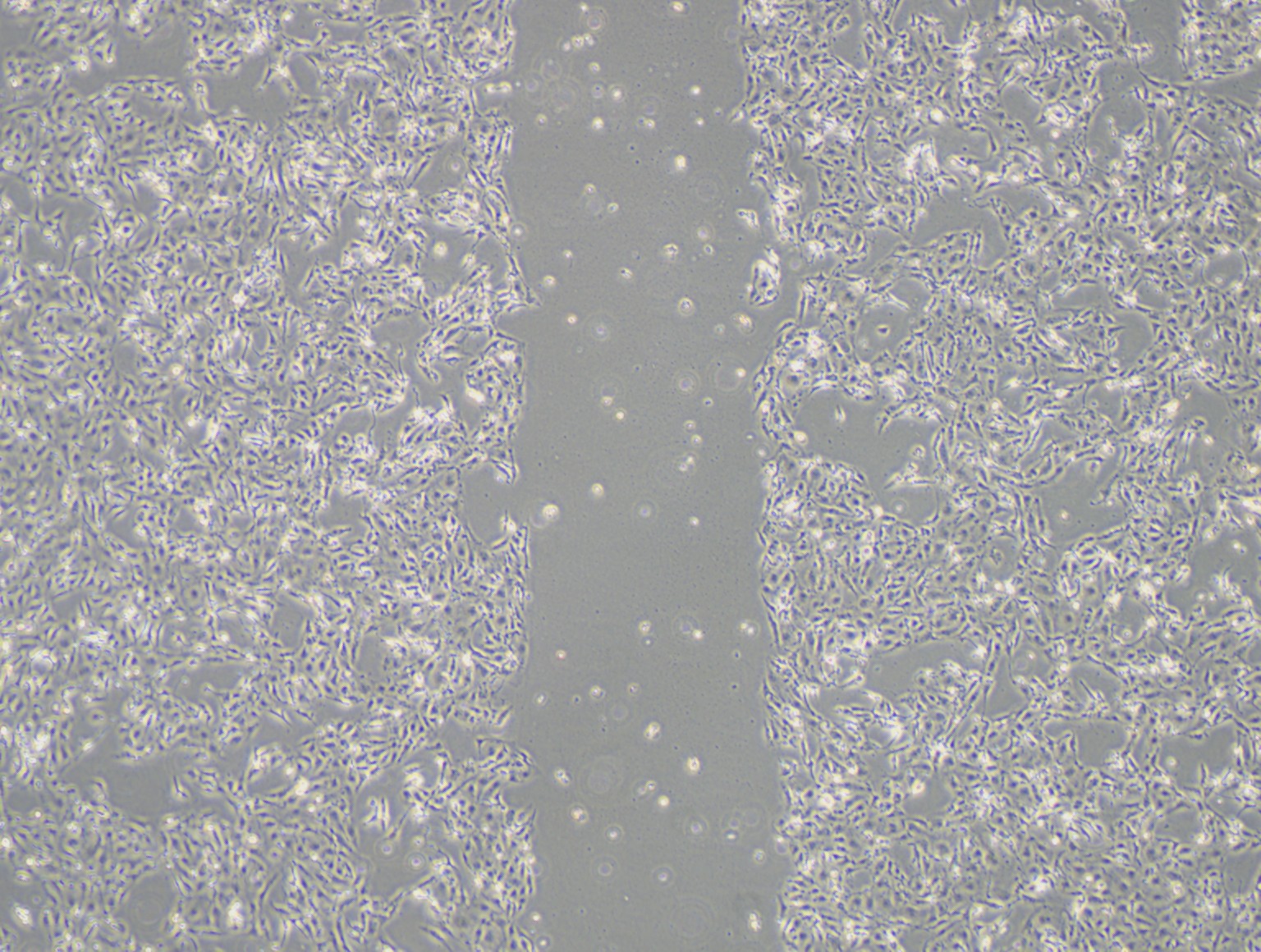

Supplement: Supplementary file 7 [file DataSheet7.zip › wound healing assay-PF-573228/2-NC-24h.jpg]

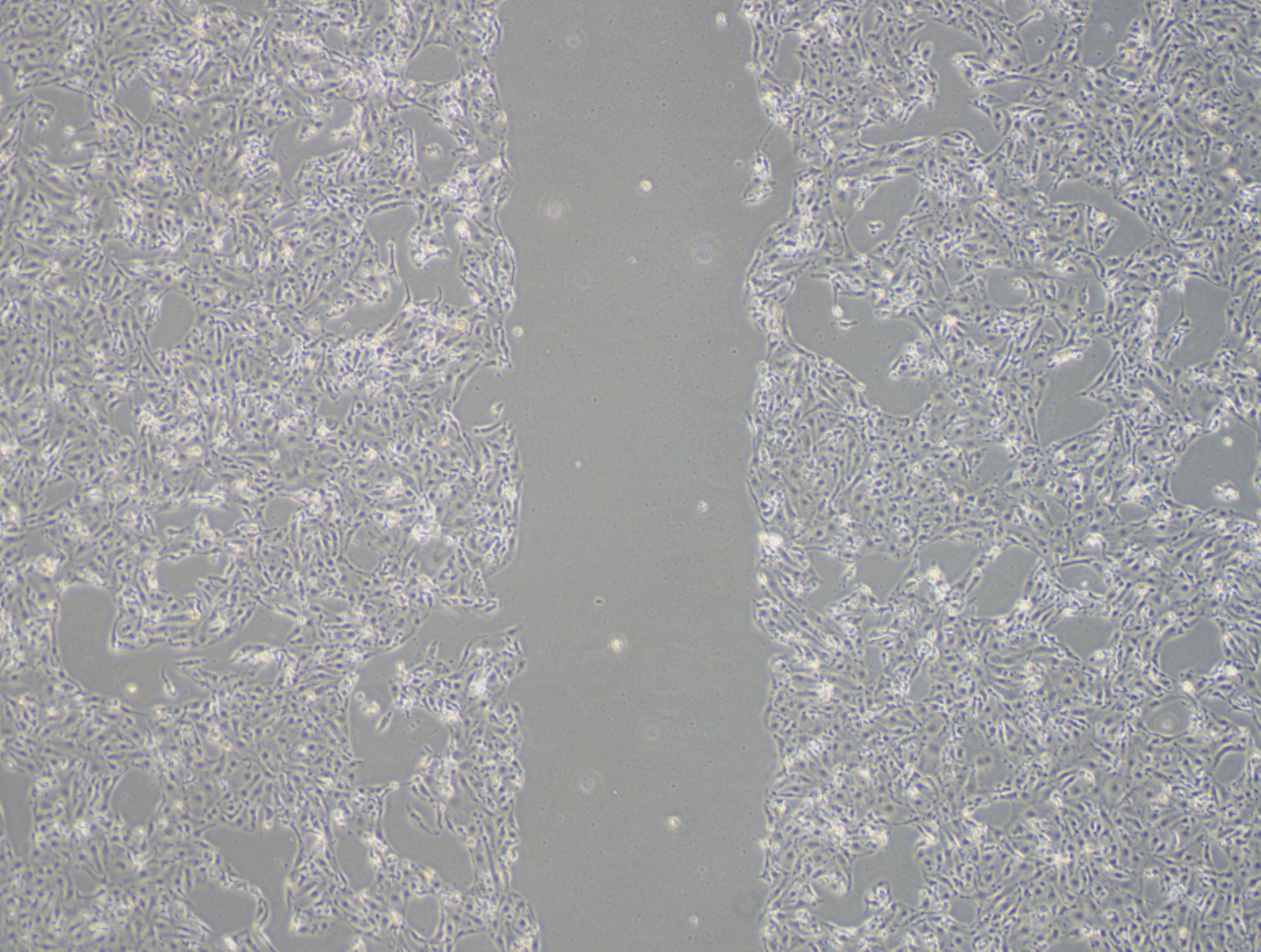

Supplement: Supplementary file 7 [file DataSheet7.zip › wound healing assay-PF-573228/2-NC-48h.jpg]

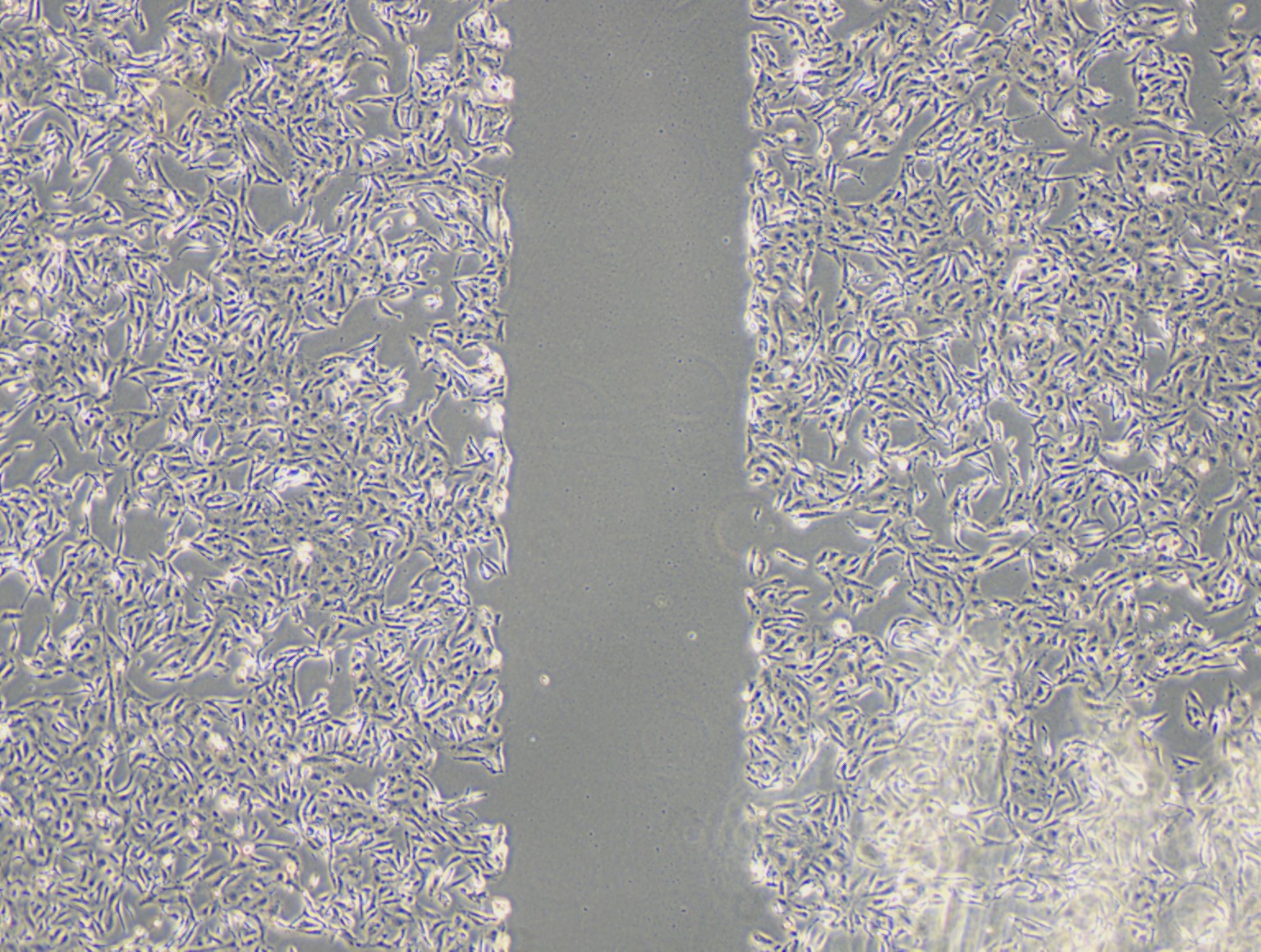

Supplement: Supplementary file 7 [file DataSheet7.zip › wound healing assay-PF-573228/2-oe-0h.jpg]

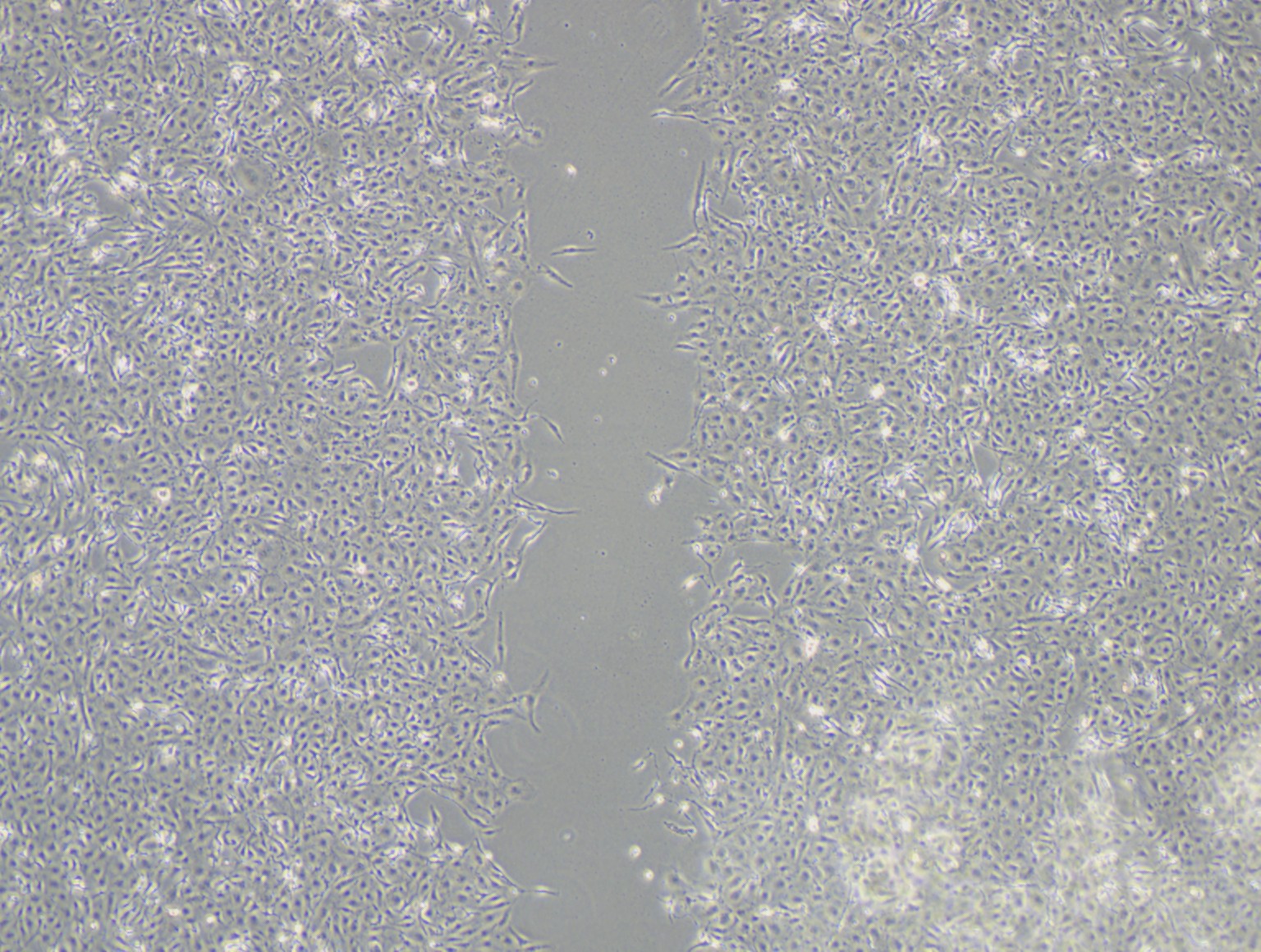

Supplement: Supplementary file 7 [file DataSheet7.zip › wound healing assay-PF-573228/2-oe-24h.jpg]

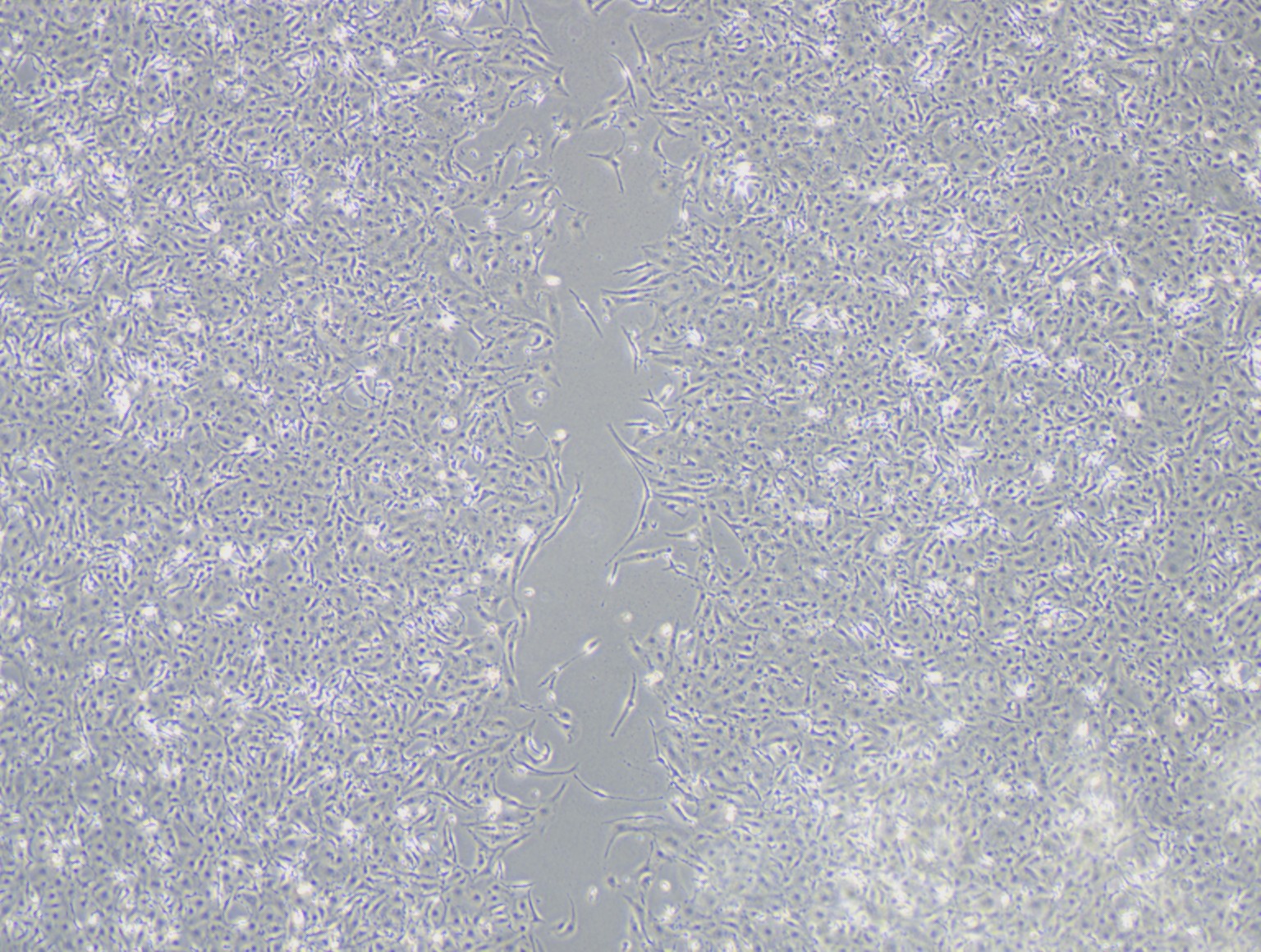

Supplement: Supplementary file 7 [file DataSheet7.zip › wound healing assay-PF-573228/2-oe-48h.jpg]

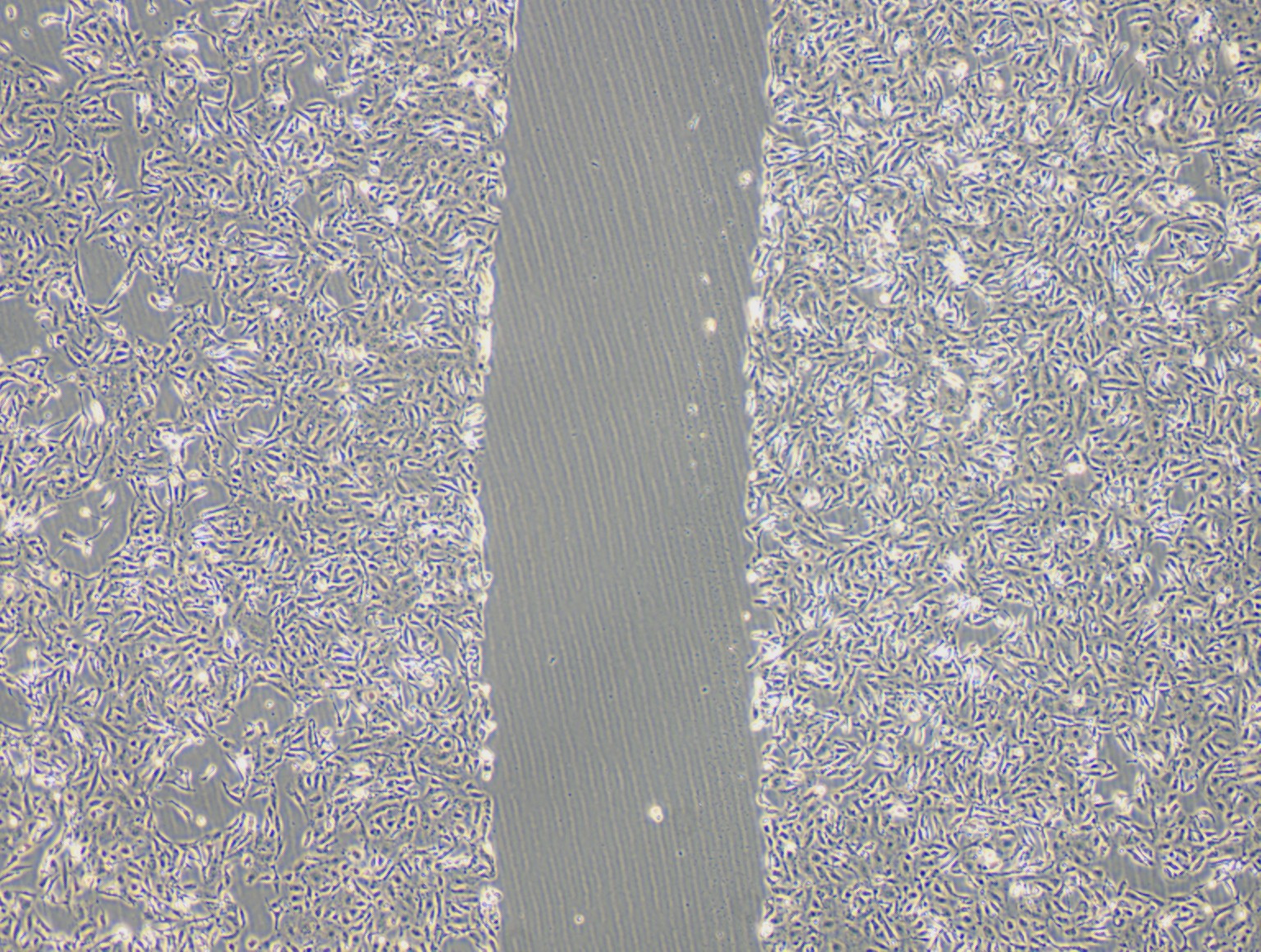

Supplement: Supplementary file 7 [file DataSheet7.zip › wound healing assay-PF-573228/2-PF-573228-0h.jpg]

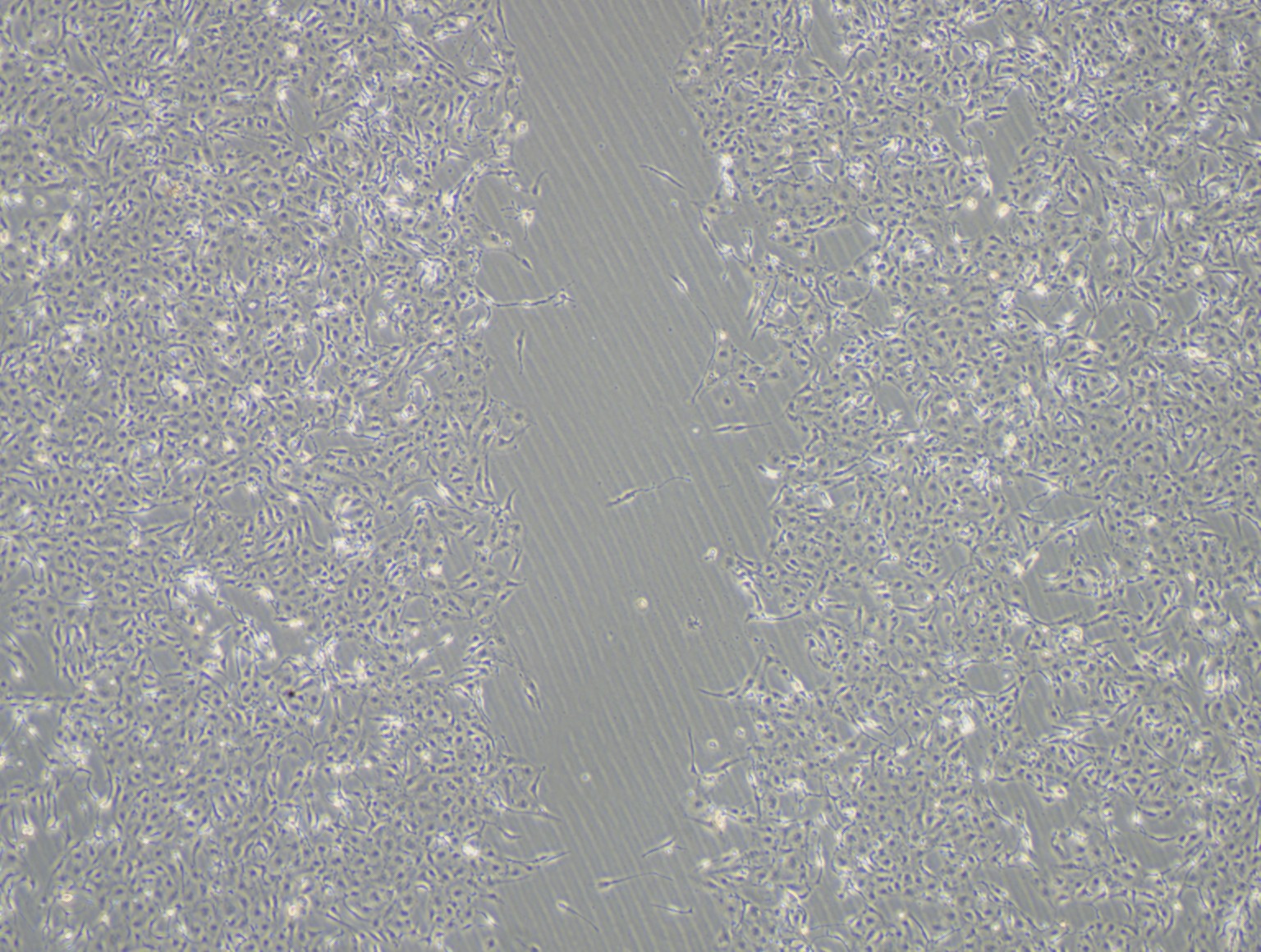

Supplement: Supplementary file 7 [file DataSheet7.zip › wound healing assay-PF-573228/2-PF-573228-24h.jpg]

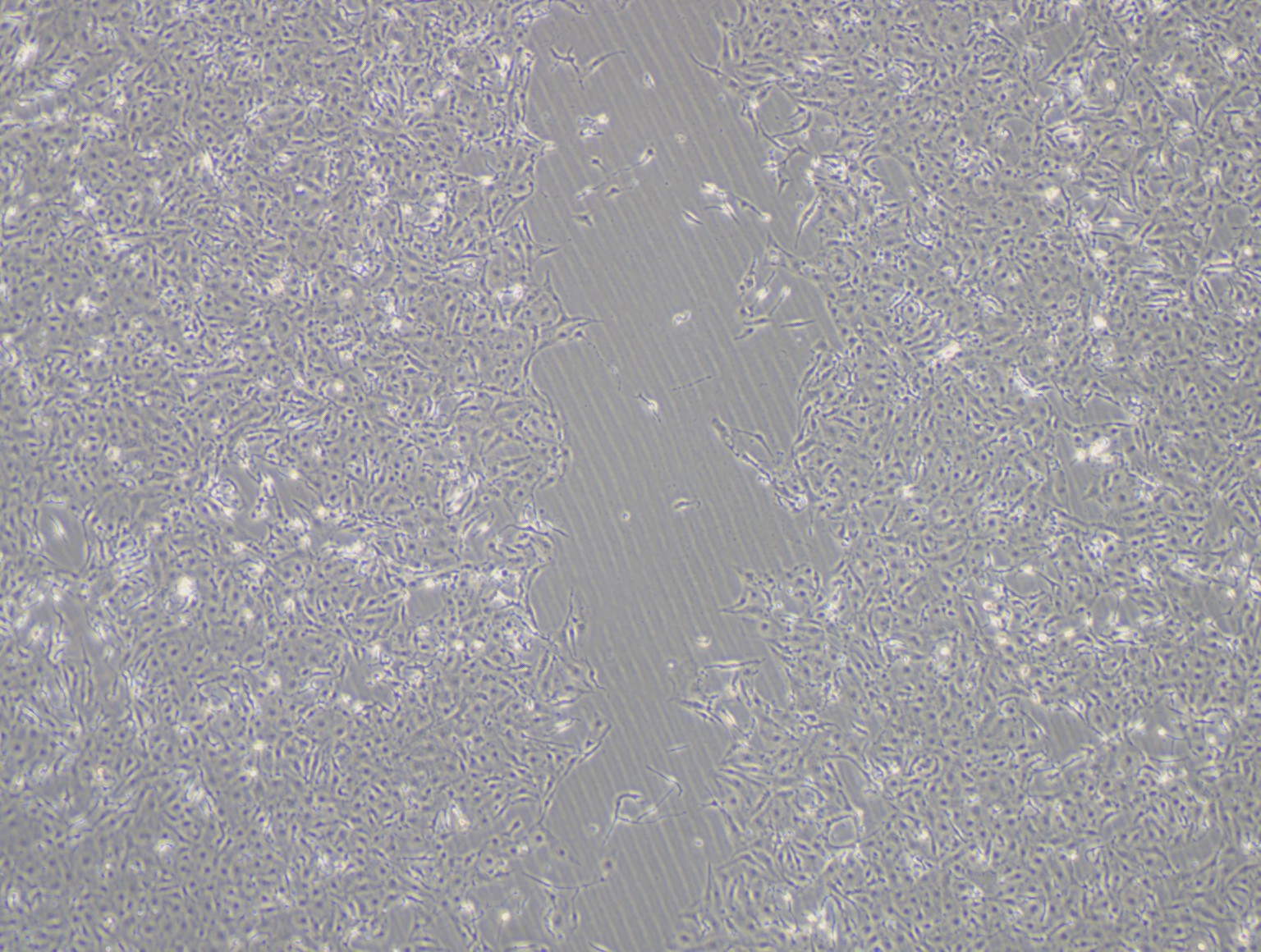

Supplement: Supplementary file 7 [file DataSheet7.zip › wound healing assay-PF-573228/2-PF-573228-48h.jpg]

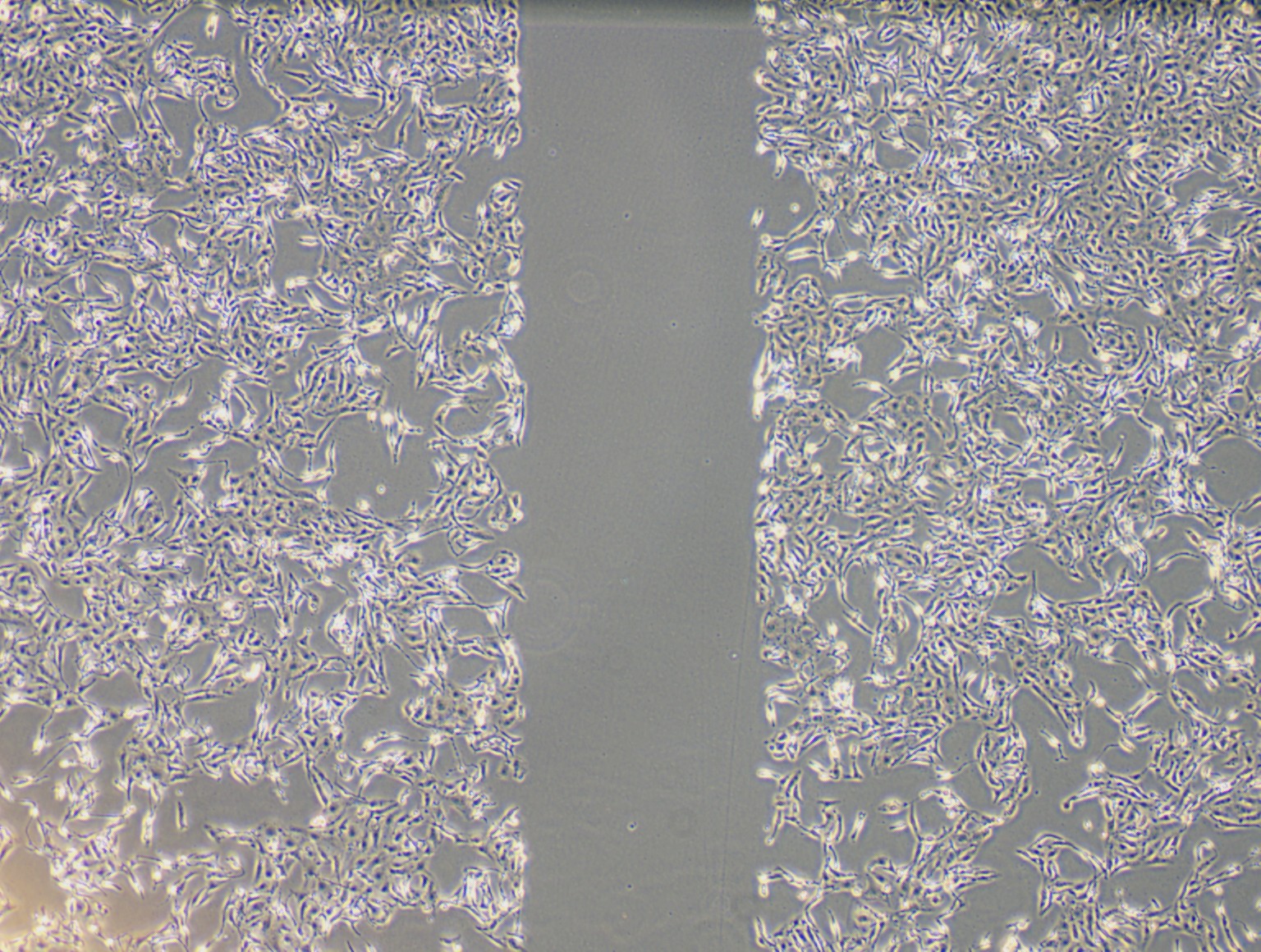

Supplement: Supplementary file 7 [file DataSheet7.zip › wound healing assay-PF-573228/3-NC-0h.jpg]

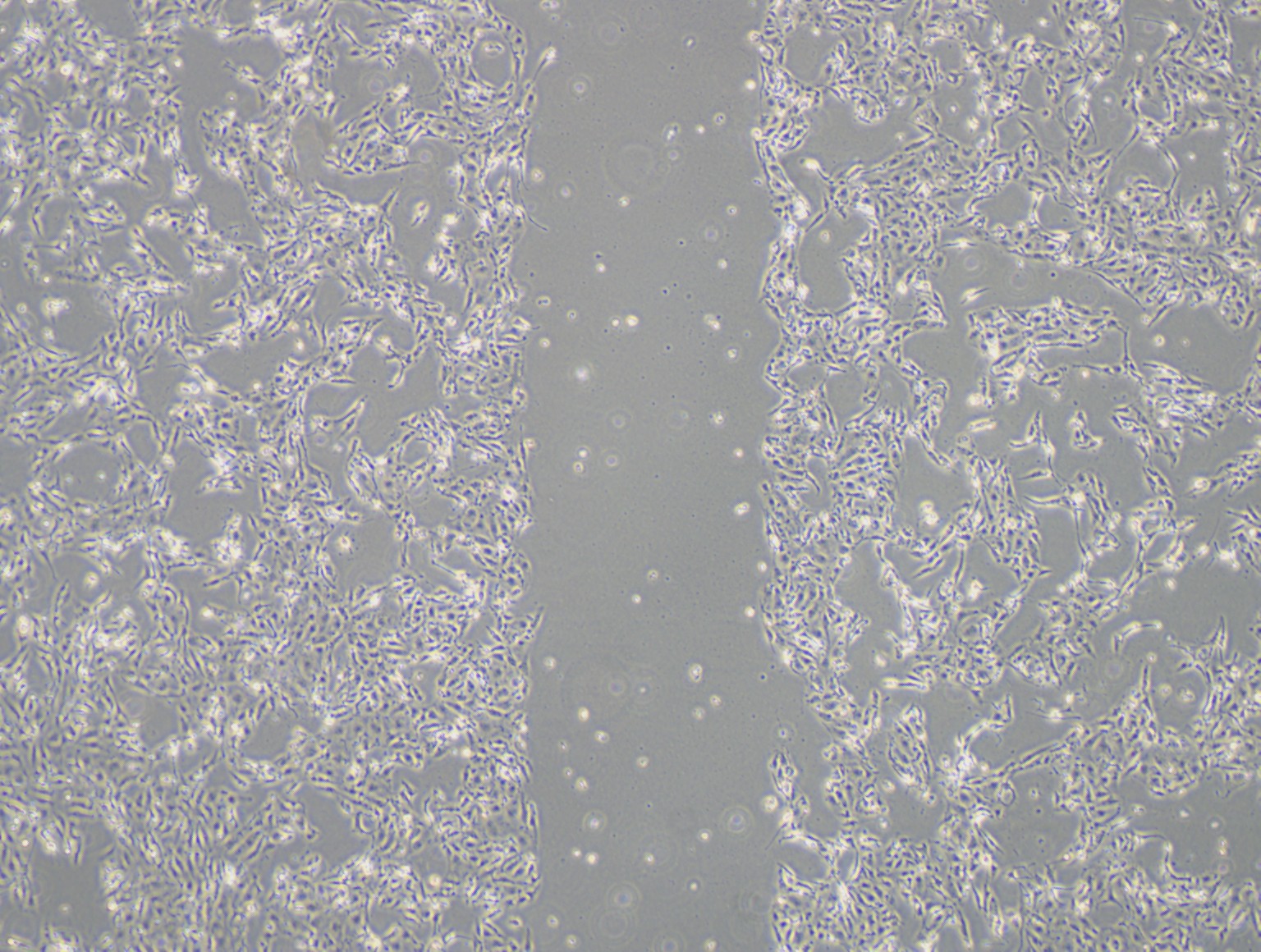

Supplement: Supplementary file 7 [file DataSheet7.zip › wound healing assay-PF-573228/3-NC-24h.jpg]

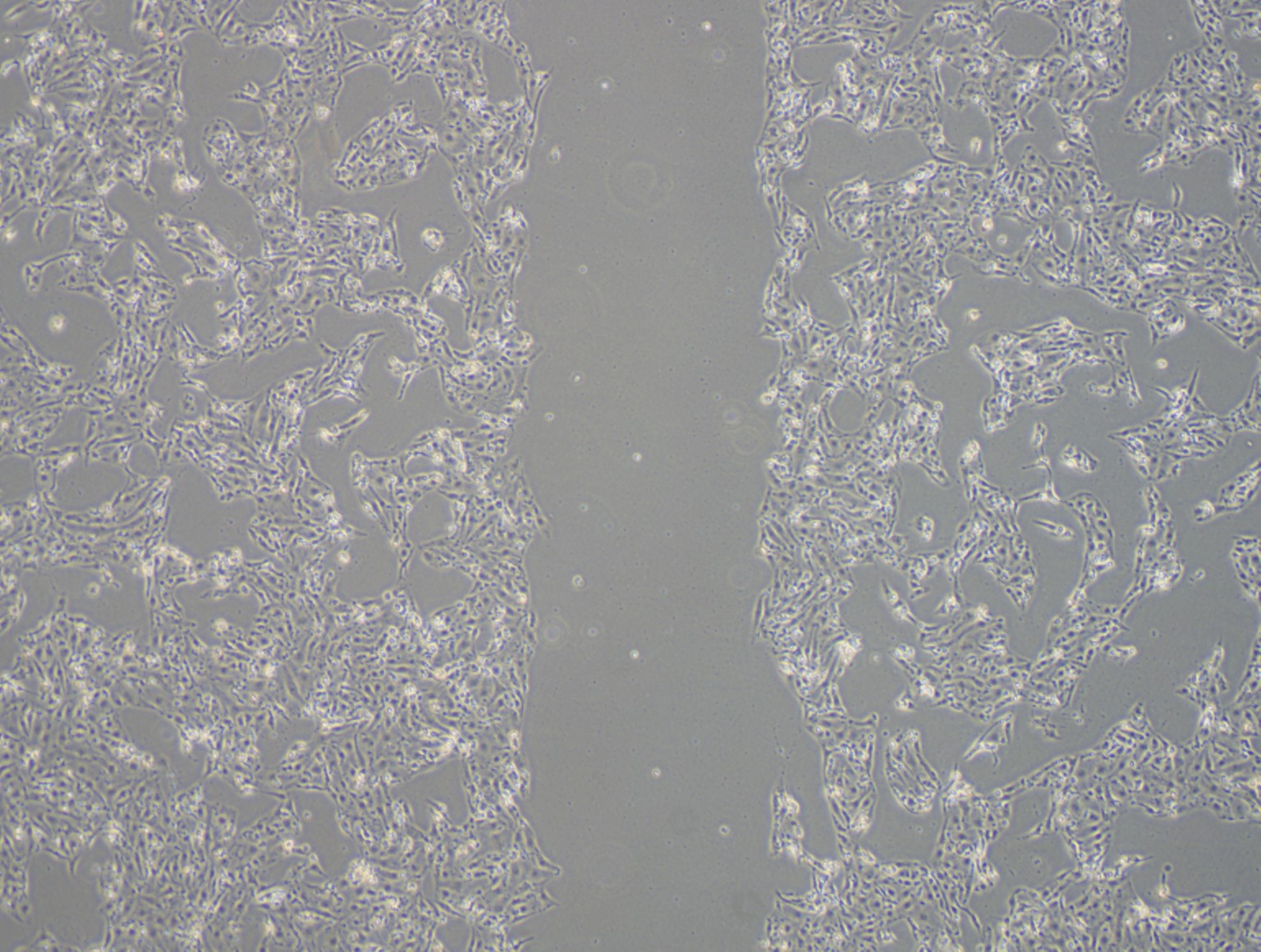

Supplement: Supplementary file 7 [file DataSheet7.zip › wound healing assay-PF-573228/3-NC-48h.jpg]

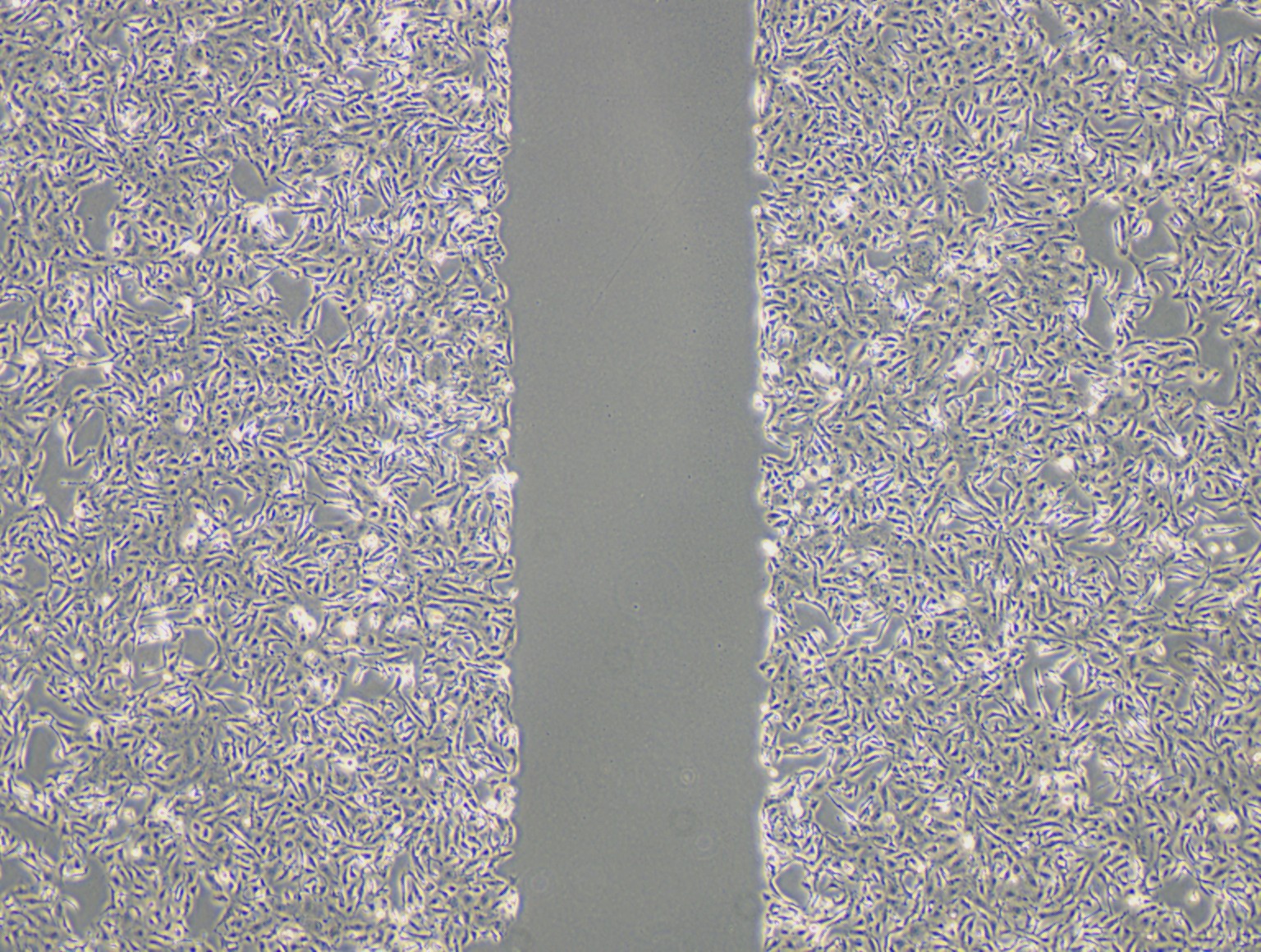

Supplement: Supplementary file 7 [file DataSheet7.zip › wound healing assay-PF-573228/3-oe-0h.jpg]

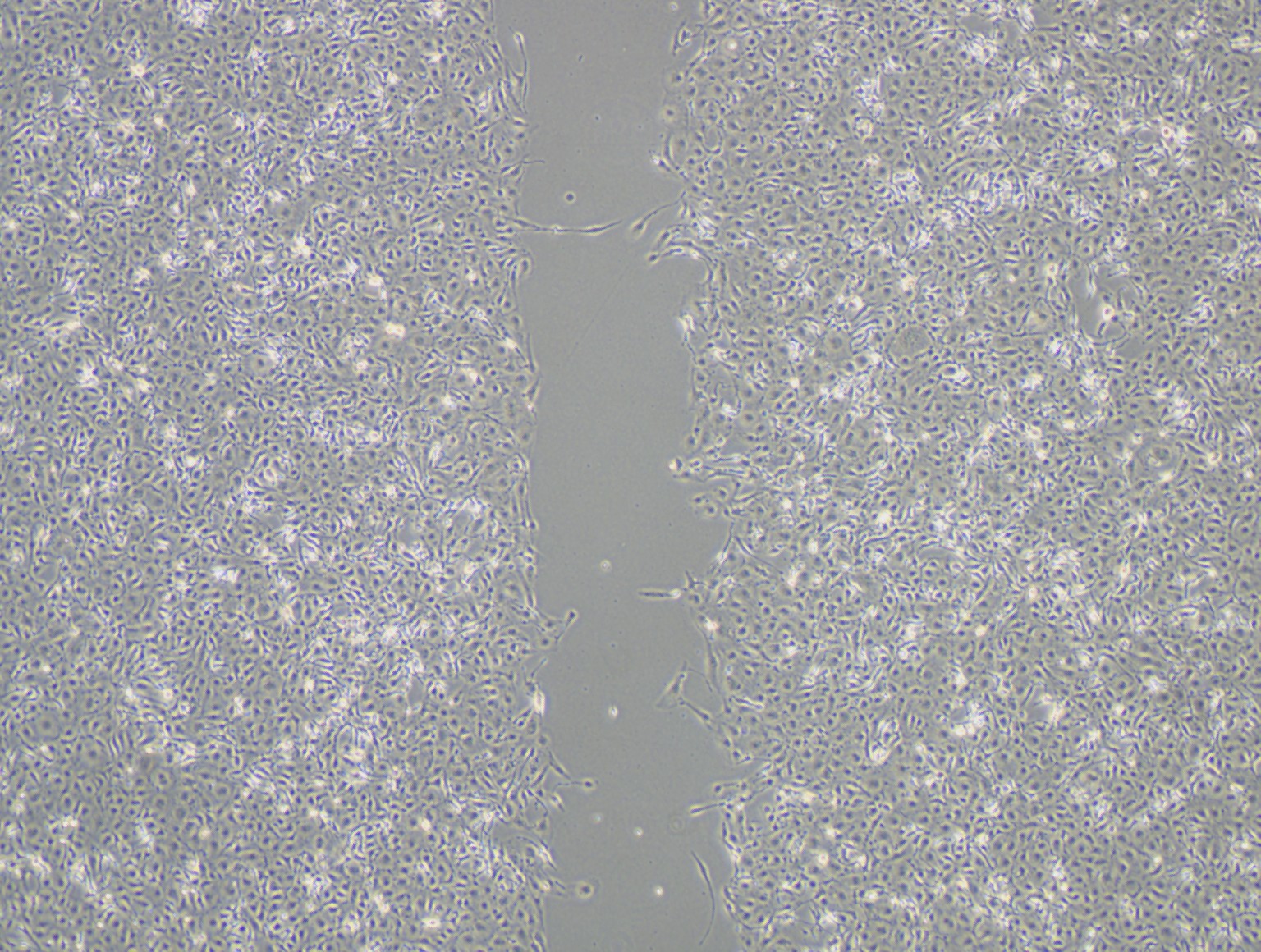

Supplement: Supplementary file 7 [file DataSheet7.zip › wound healing assay-PF-573228/3-oe-24h.jpg]

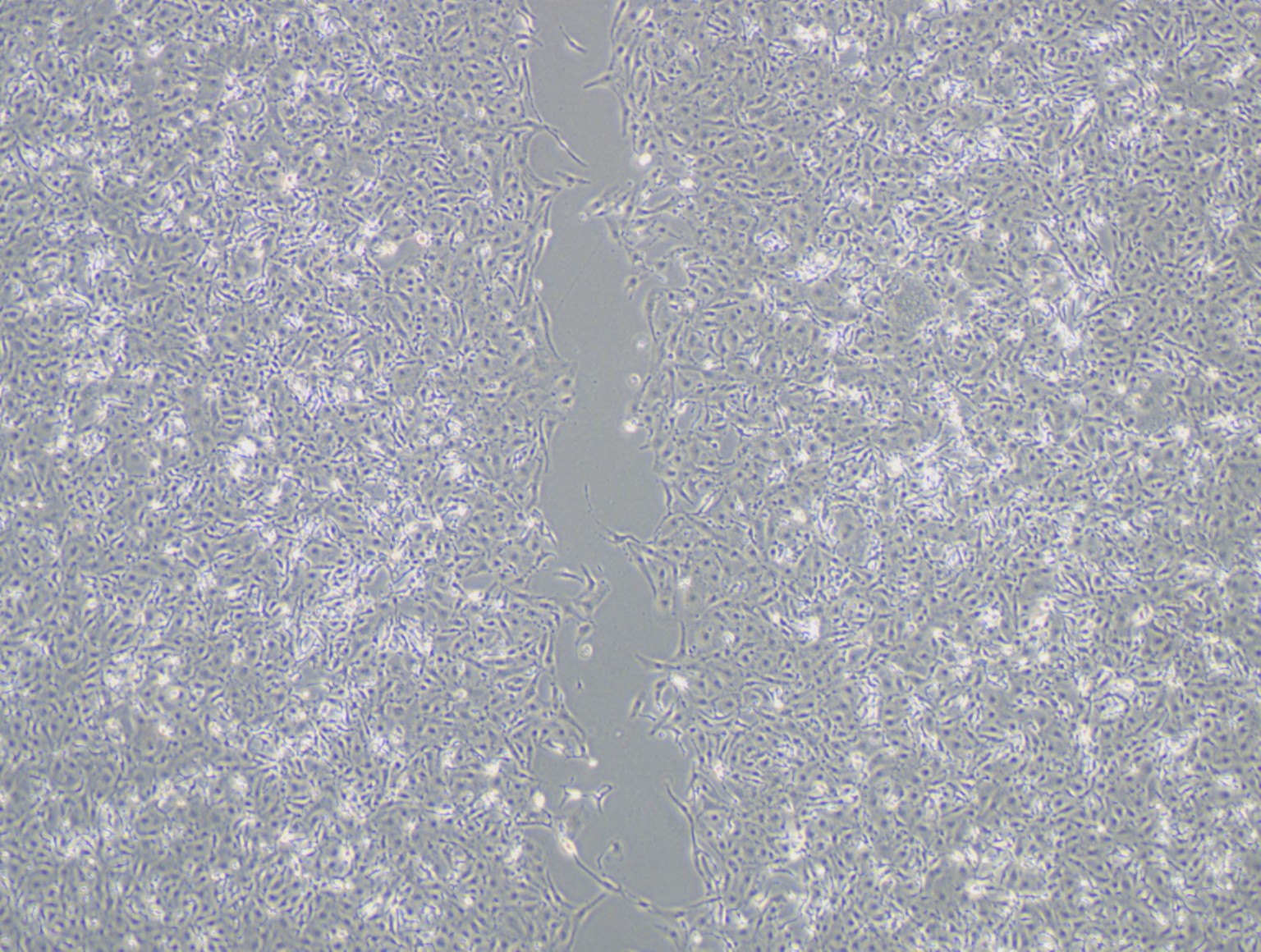

Supplement: Supplementary file 7 [file DataSheet7.zip › wound healing assay-PF-573228/3-oe-48h.jpg]

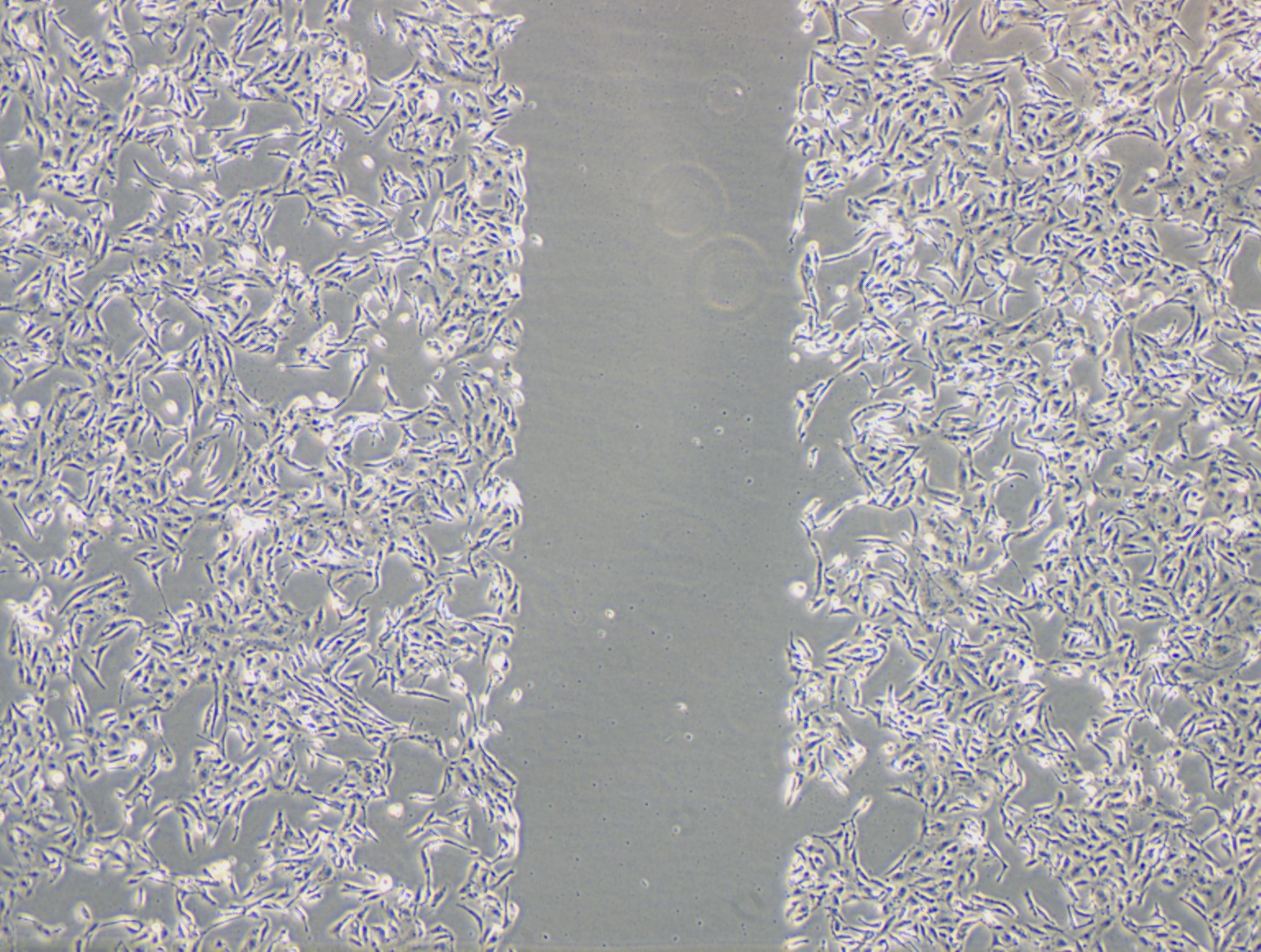

Supplement: Supplementary file 7 [file DataSheet7.zip › wound healing assay-PF-573228/3-PF-573228-0h.jpg]

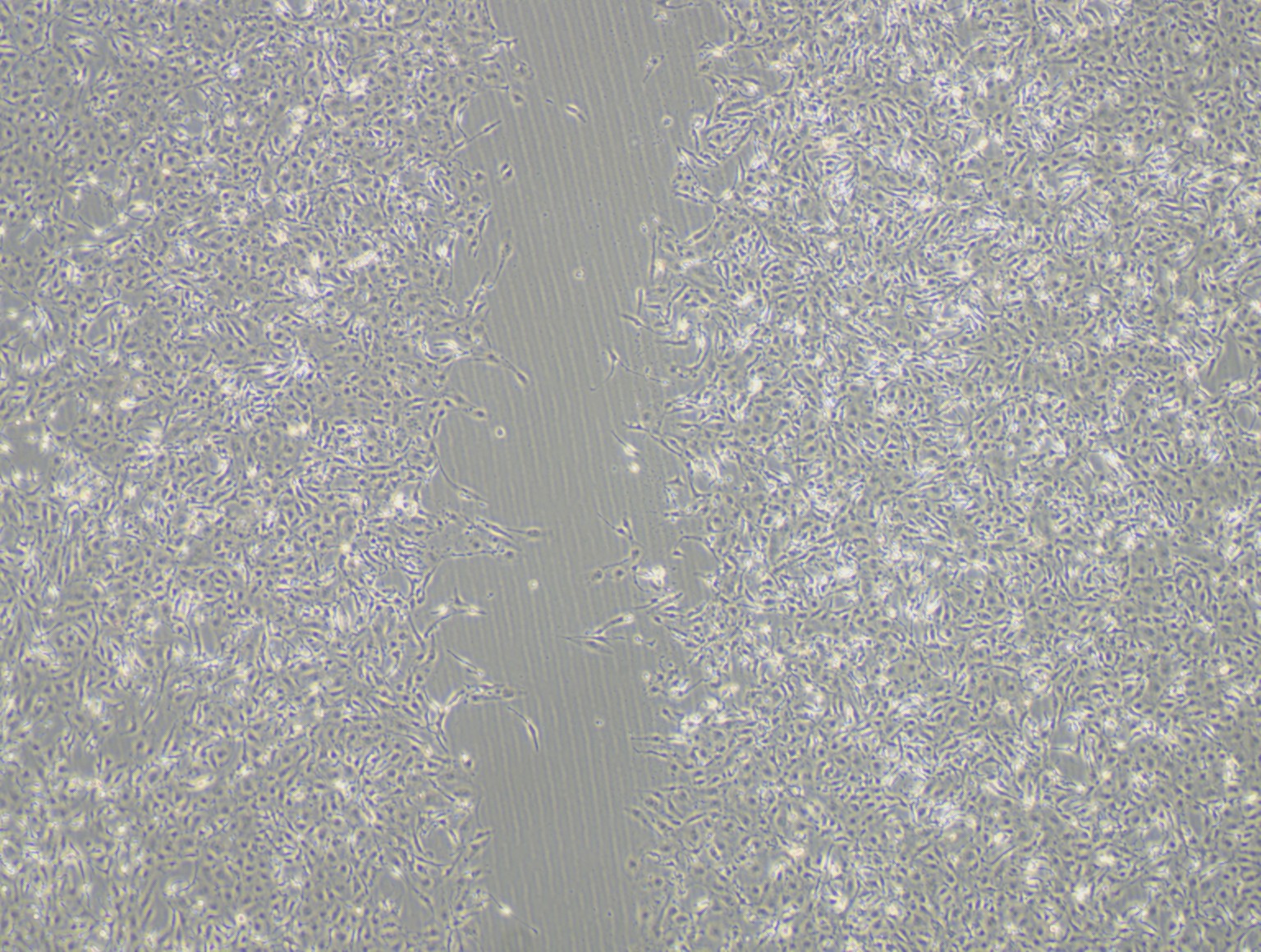

Supplement: Supplementary file 7 [file DataSheet7.zip › wound healing assay-PF-573228/3-PF-573228-24h.jpg]

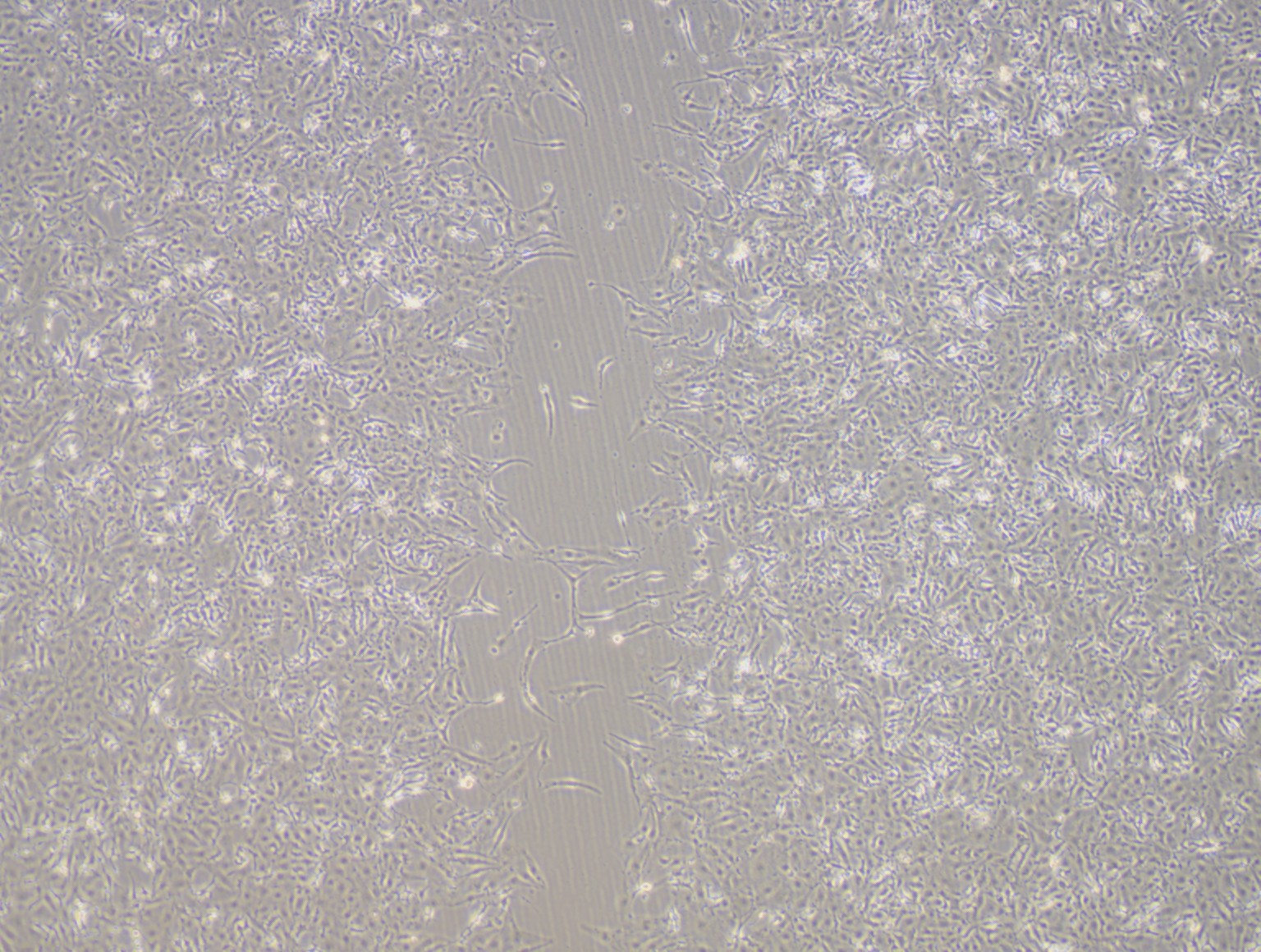

Supplement: Supplementary file 7 [file DataSheet7.zip › wound healing assay-PF-573228/3-PF-573228-48h.jpg]

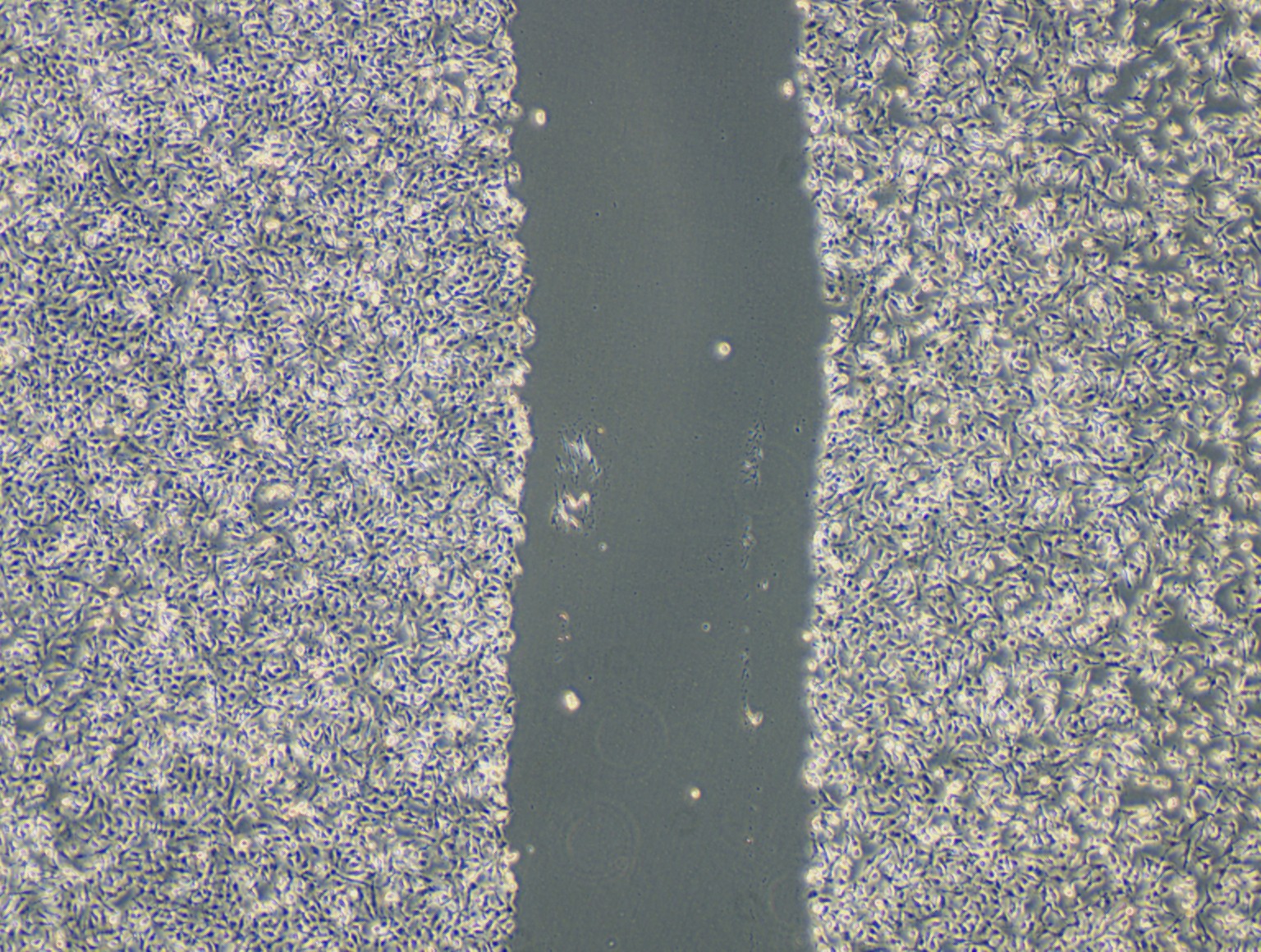

Supplement: Supplementary file 8 [file DataSheet8.zip › wound healing assay-si-COL1A2/1-NC-0h.jpg]

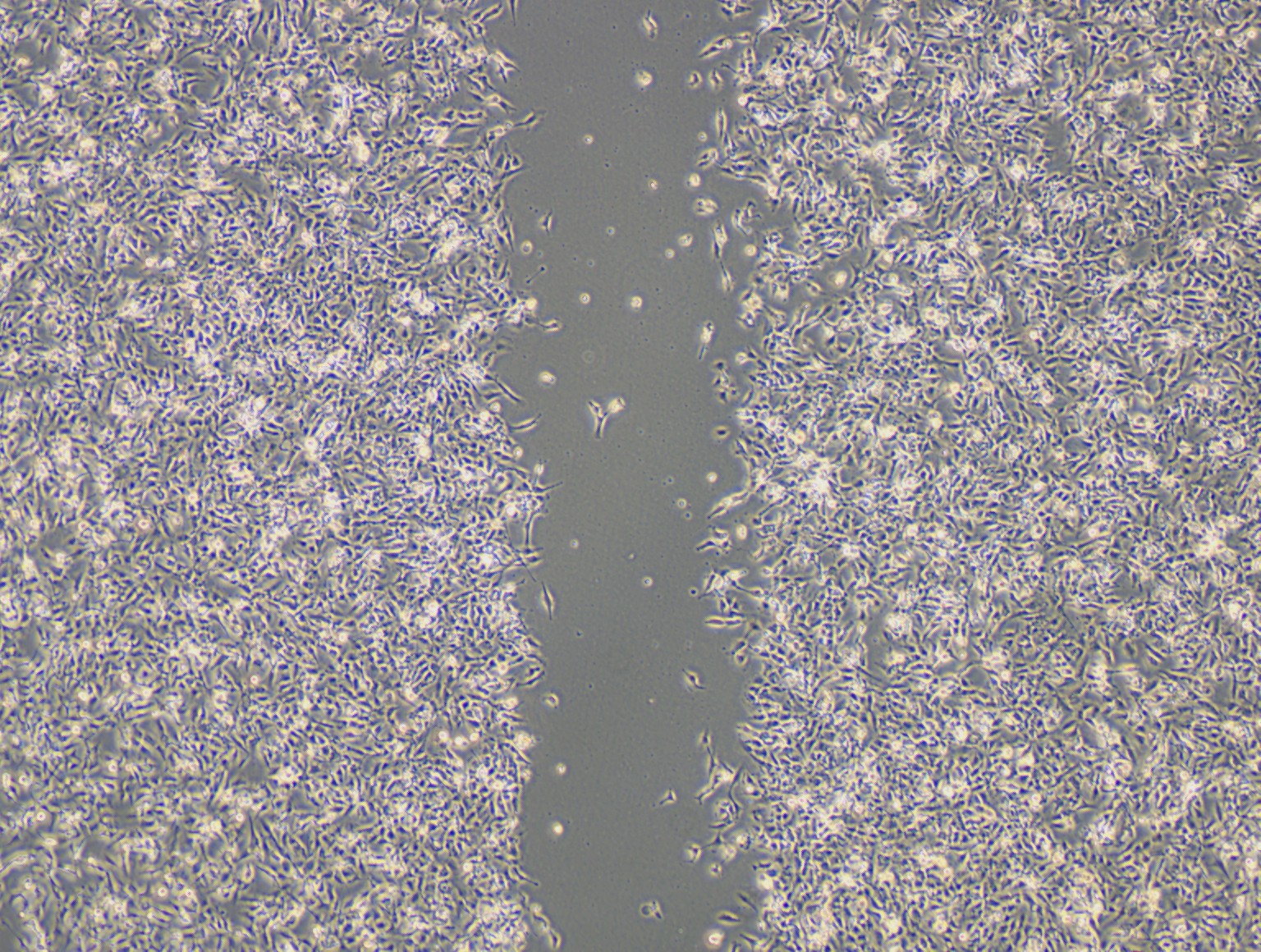

Supplement: Supplementary file 8 [file DataSheet8.zip › wound healing assay-si-COL1A2/1-NC-24h.jpg]

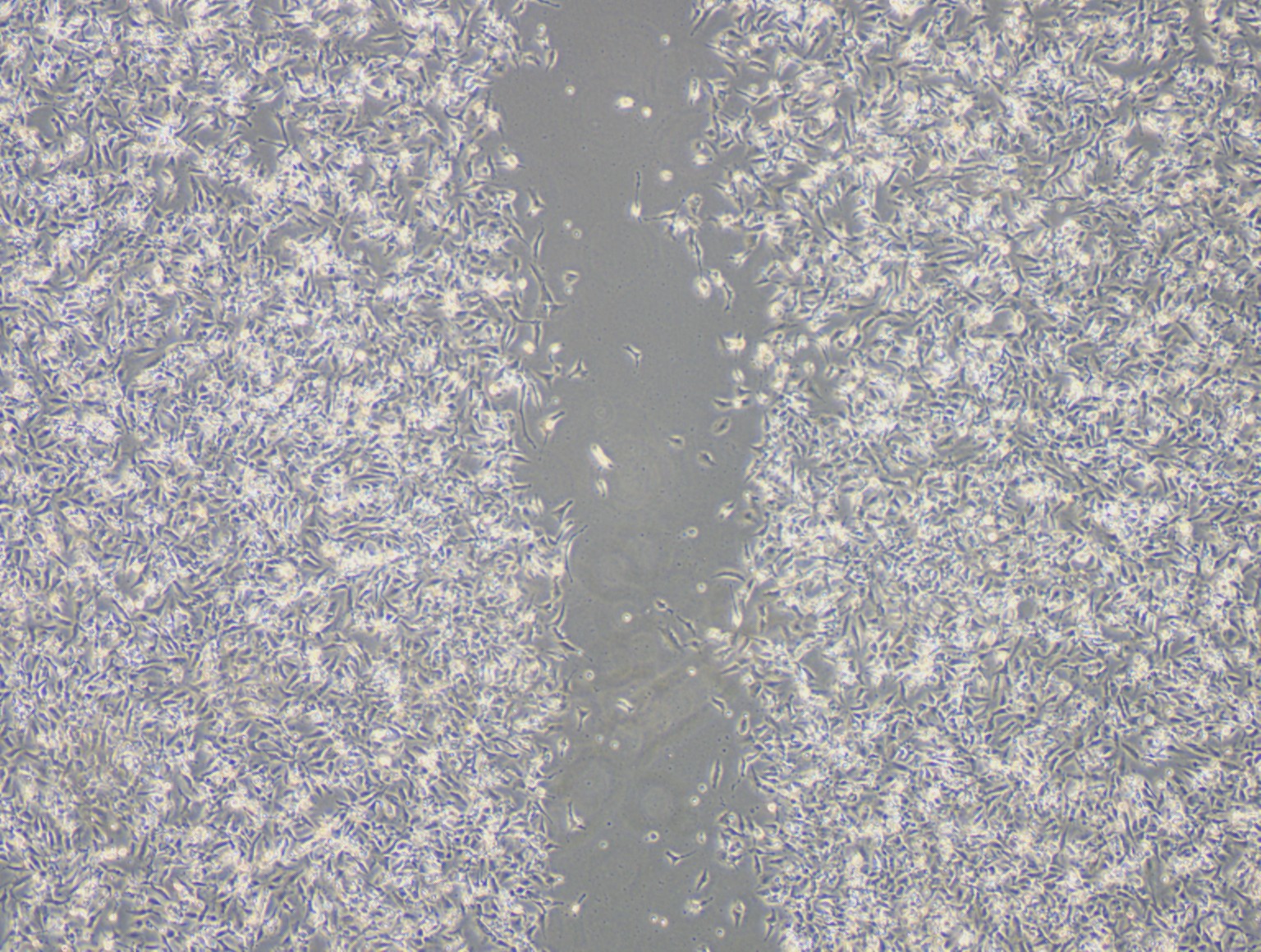

Supplement: Supplementary file 8 [file DataSheet8.zip › wound healing assay-si-COL1A2/1-NC-48h.jpg]

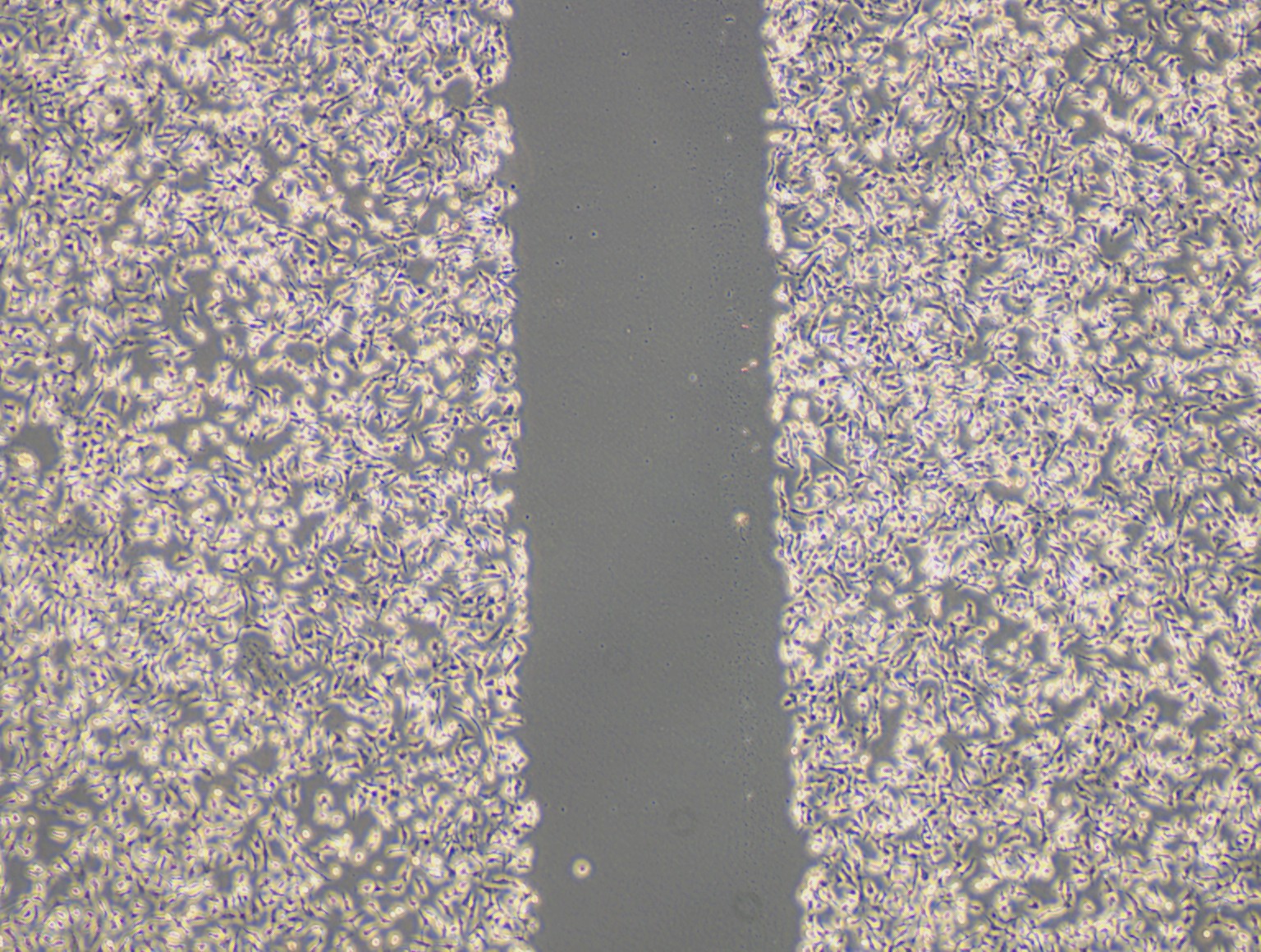

Supplement: Supplementary file 8 [file DataSheet8.zip › wound healing assay-si-COL1A2/1-si-0h.jpg]

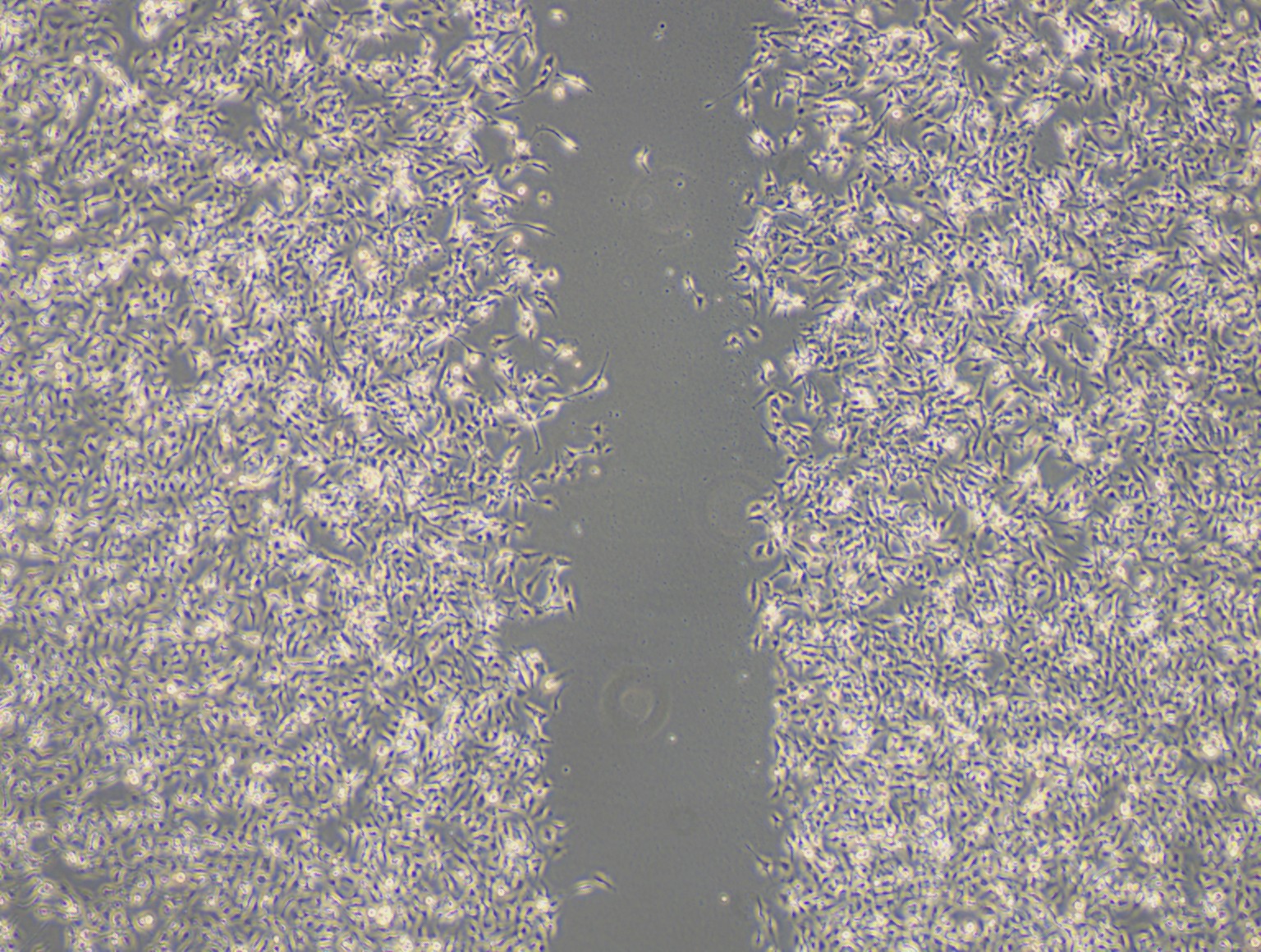

Supplement: Supplementary file 8 [file DataSheet8.zip › wound healing assay-si-COL1A2/1-si-24h.jpg]

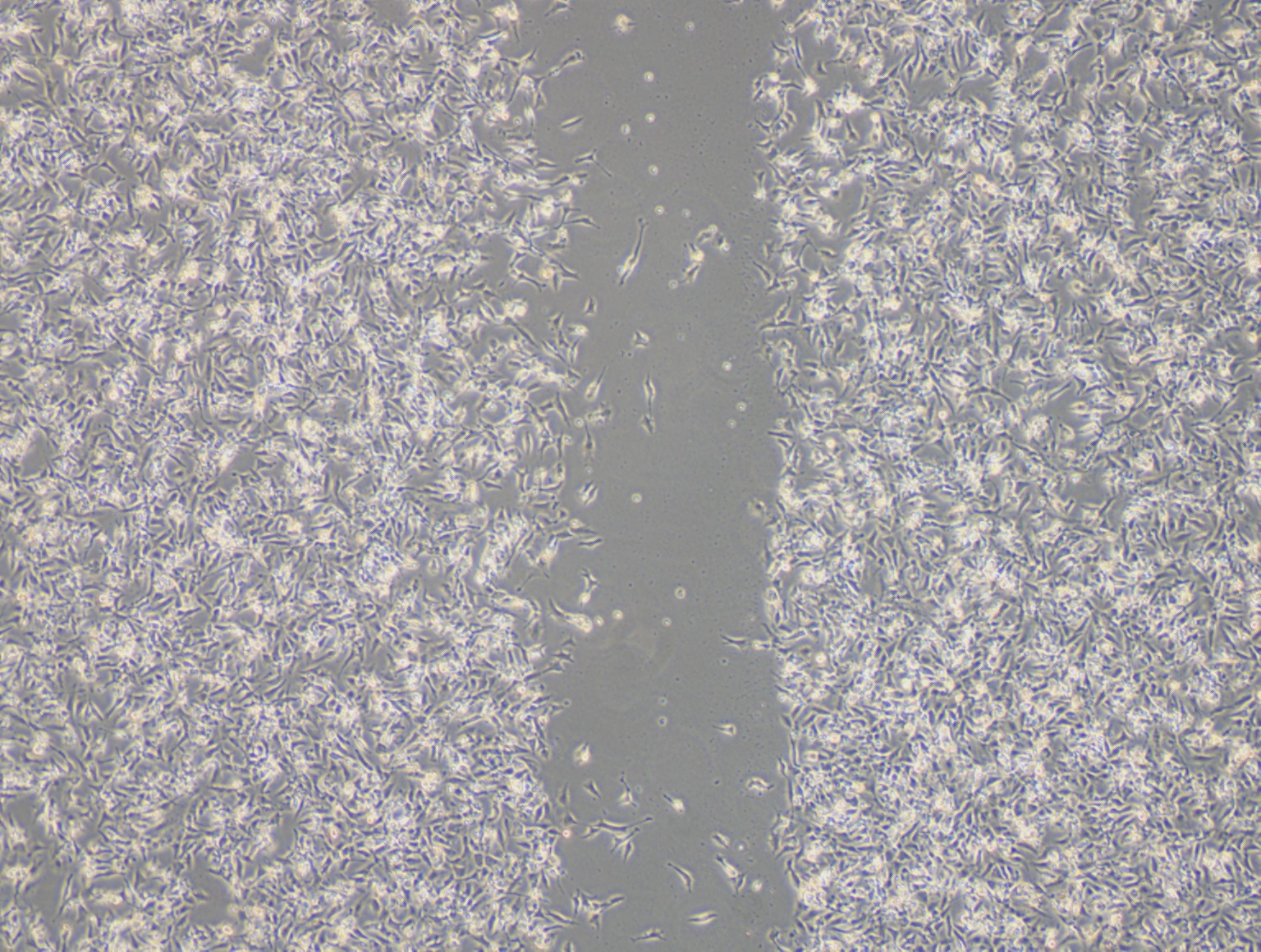

Supplement: Supplementary file 8 [file DataSheet8.zip › wound healing assay-si-COL1A2/1-si-48h.jpg]
