# Supplementary figures and images for: Integrative bulk and single-cell transcriptomic analysis reveals COL1A2-driven ECM remodeling and focal adhesion signaling associated with the transition from non-muscle-invasive to muscle-invasive bladder cancer (part 2 of 2)
Source: Front Oncol. 2026 Jan 5;15:1716324. doi: 10.3389/fonc.2025.1716324 (PMC12812713; doi:10.3389/fonc.2025.1716324)

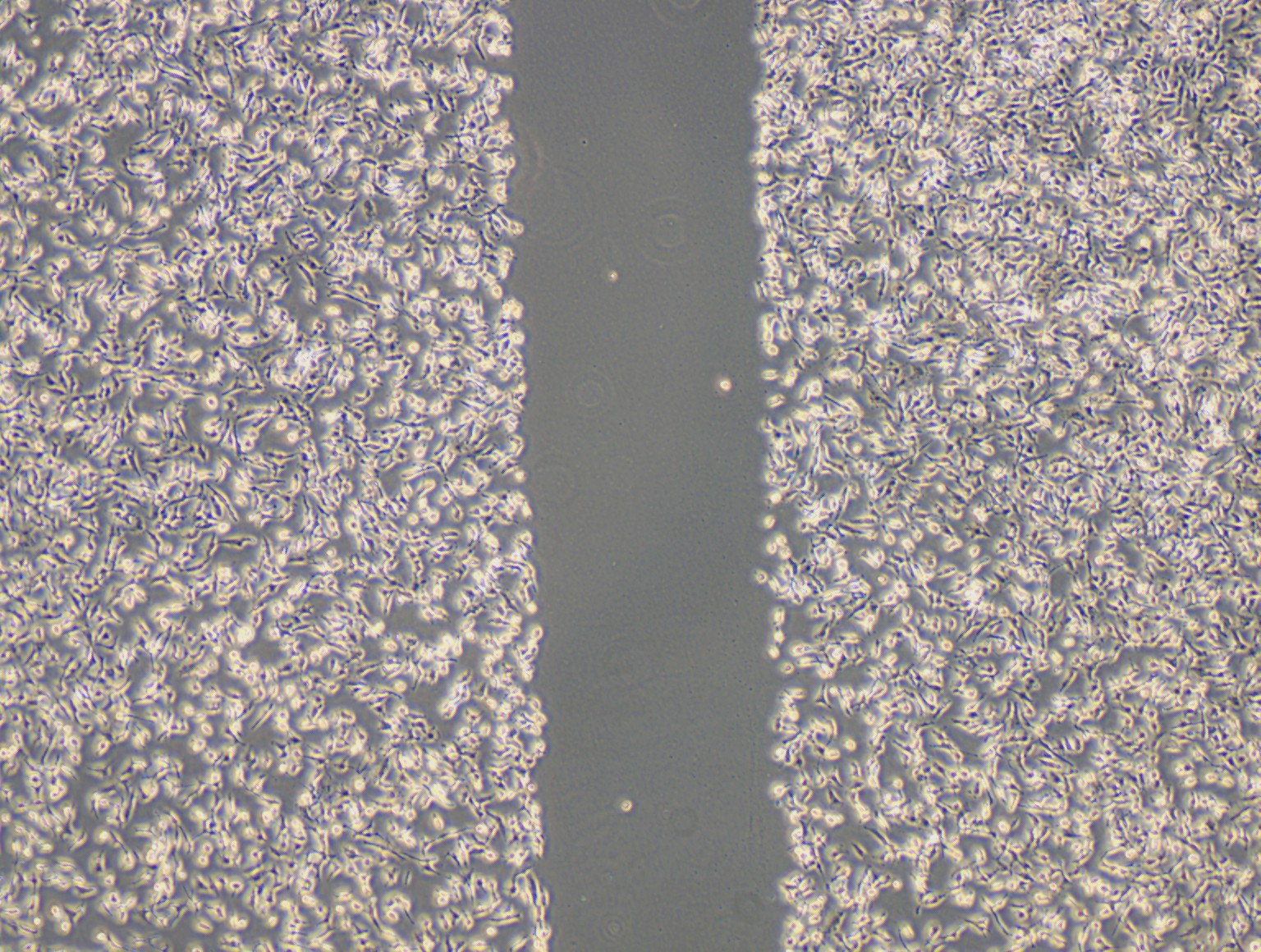

Supplement: Supplementary file 8 [file DataSheet8.zip › wound healing assay-si-COL1A2/2-NC-0h.jpg]

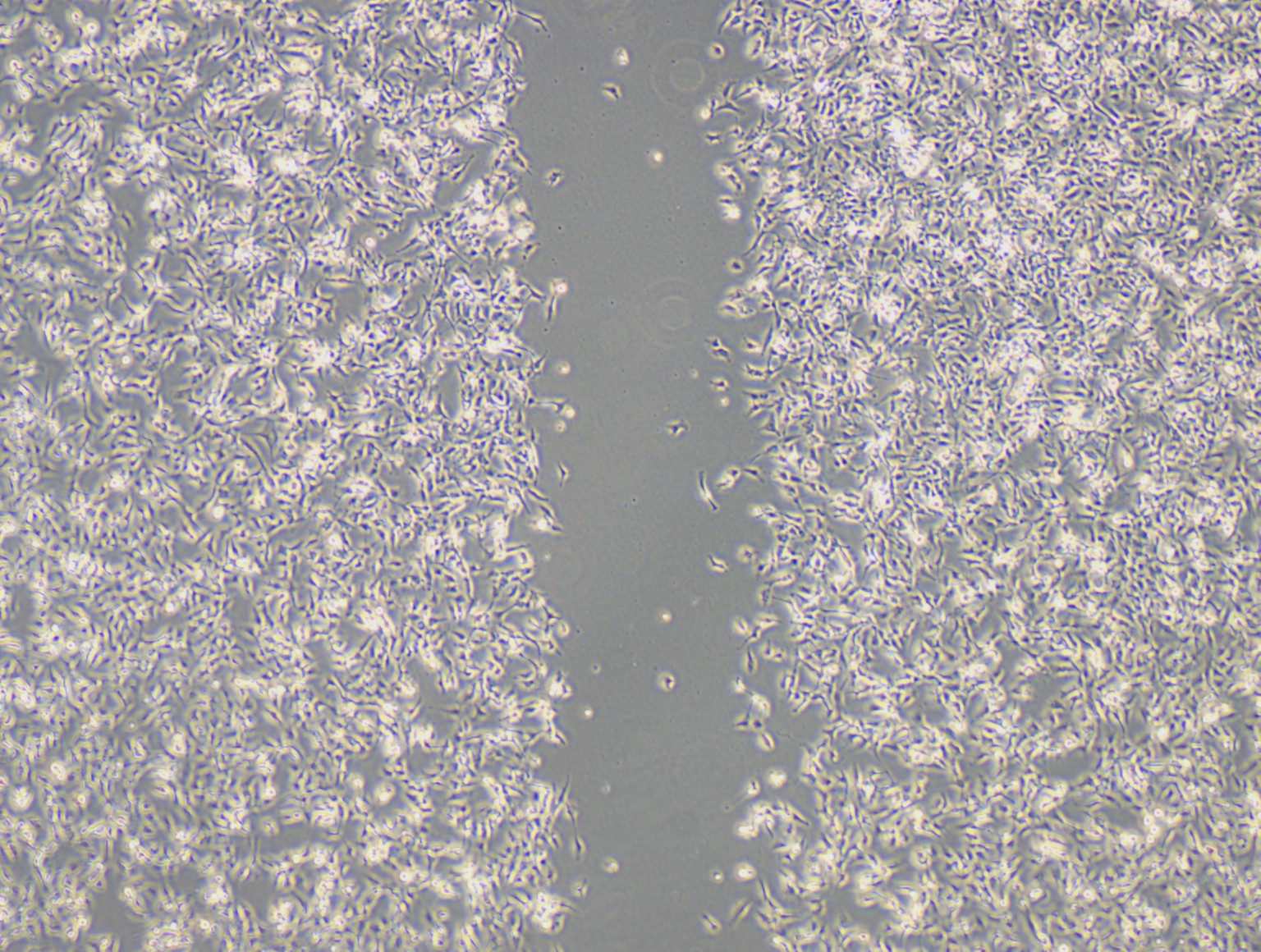

Supplement: Supplementary file 8 [file DataSheet8.zip › wound healing assay-si-COL1A2/2-NC-24h.jpg]

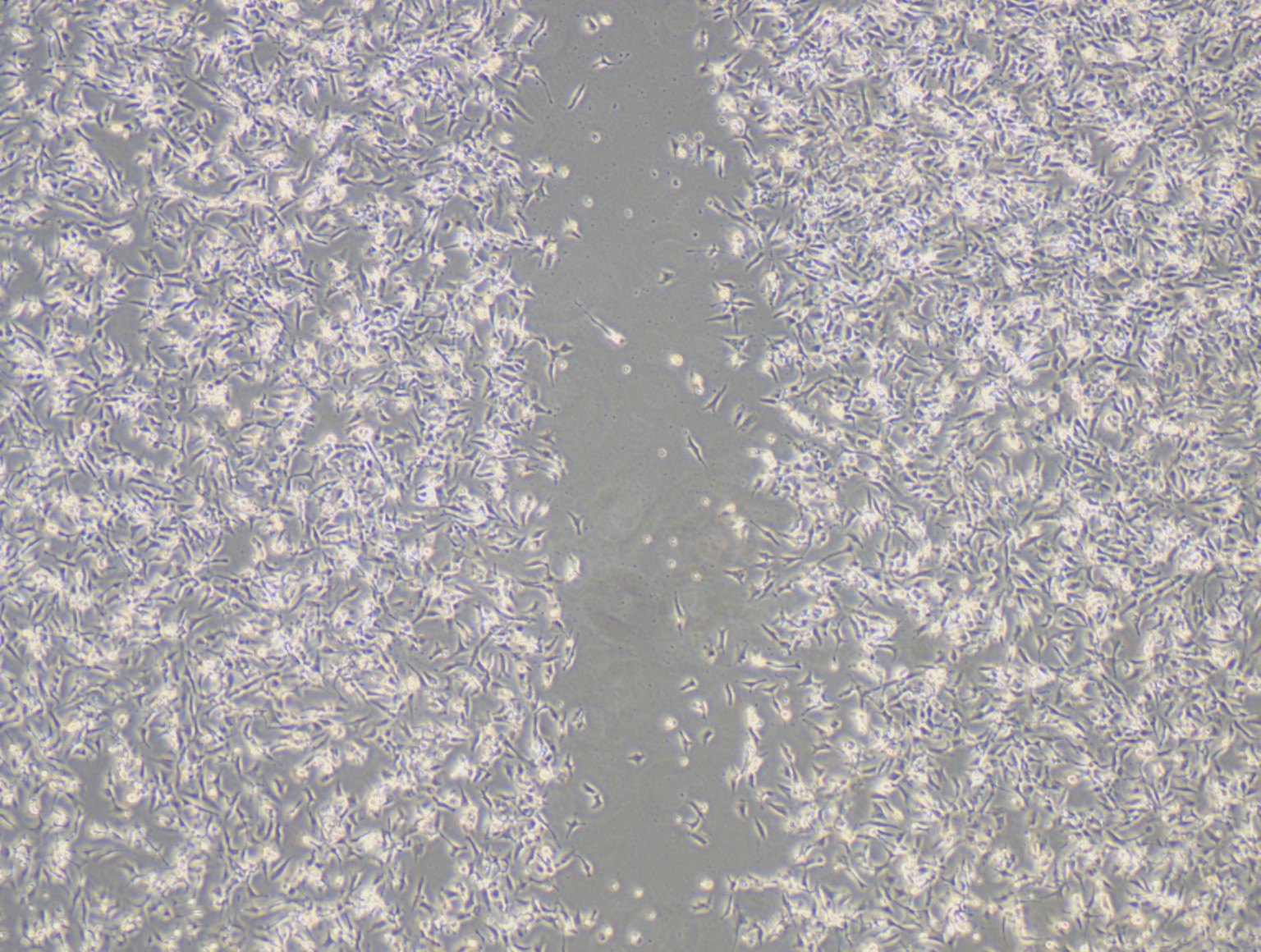

Supplement: Supplementary file 8 [file DataSheet8.zip › wound healing assay-si-COL1A2/2-NC-48h.jpg]

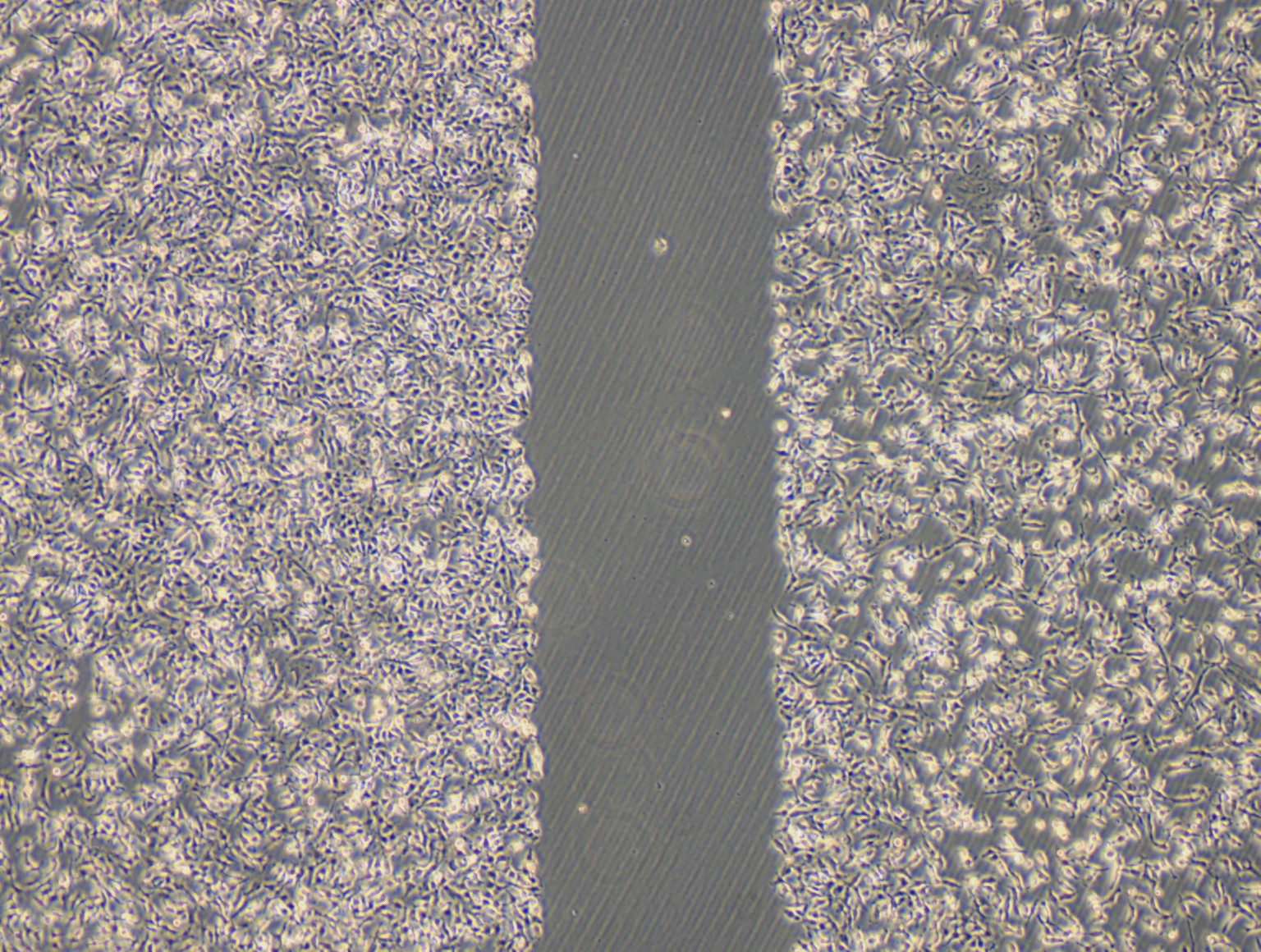

Supplement: Supplementary file 8 [file DataSheet8.zip › wound healing assay-si-COL1A2/2-si-0h.jpg]

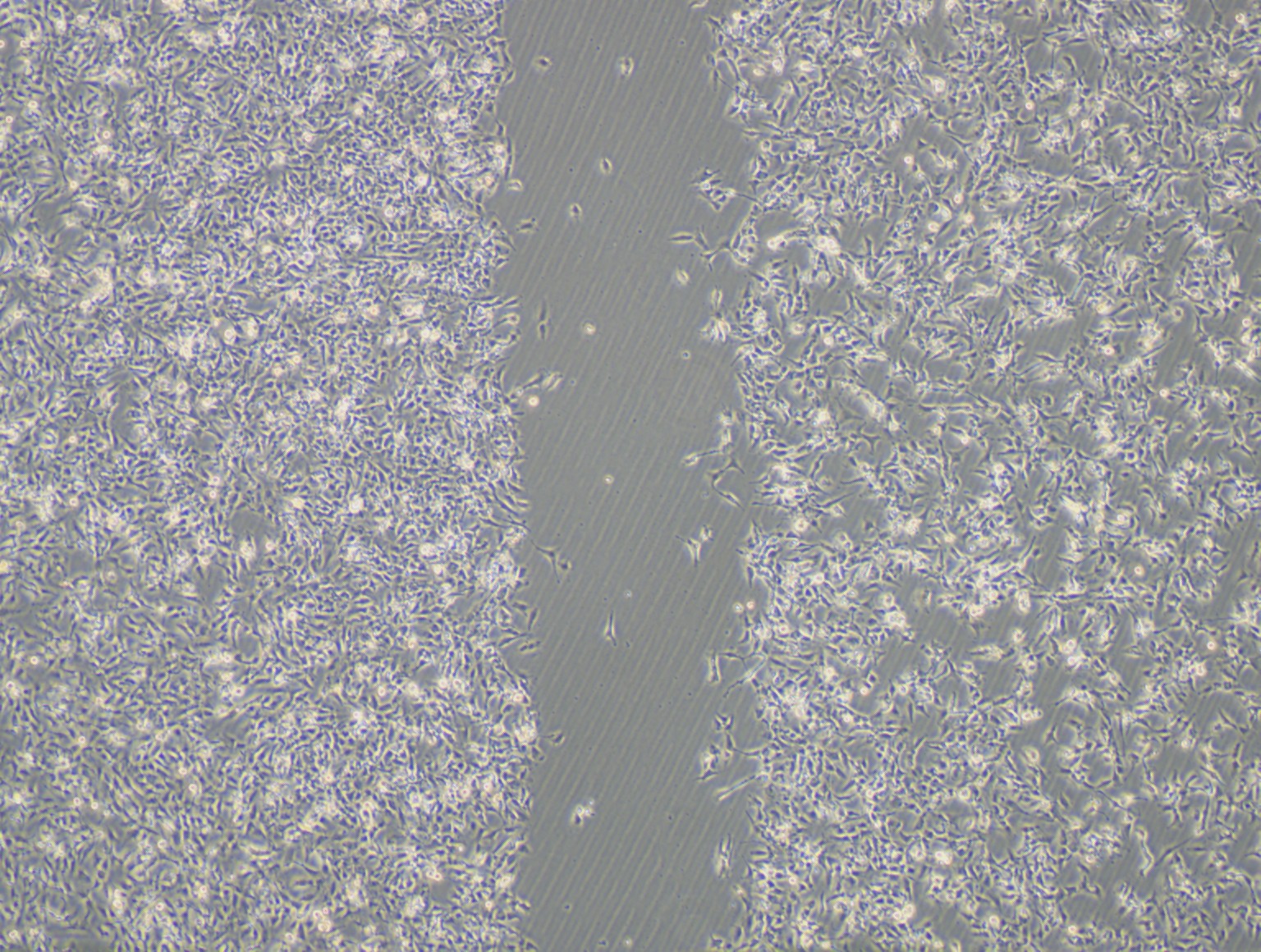

Supplement: Supplementary file 8 [file DataSheet8.zip › wound healing assay-si-COL1A2/2-si-24h.jpg]

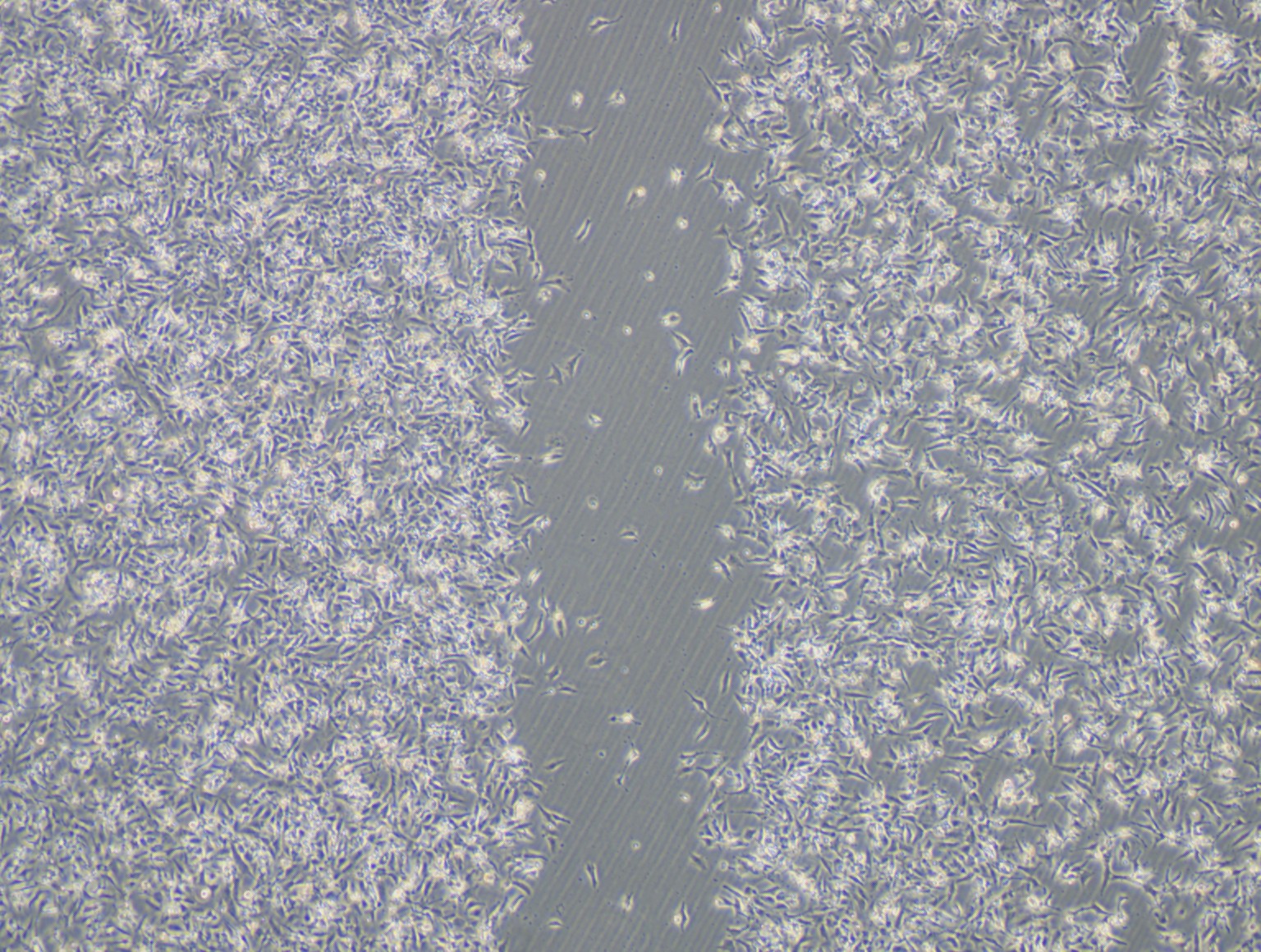

Supplement: Supplementary file 8 [file DataSheet8.zip › wound healing assay-si-COL1A2/2-si-48h.jpg]

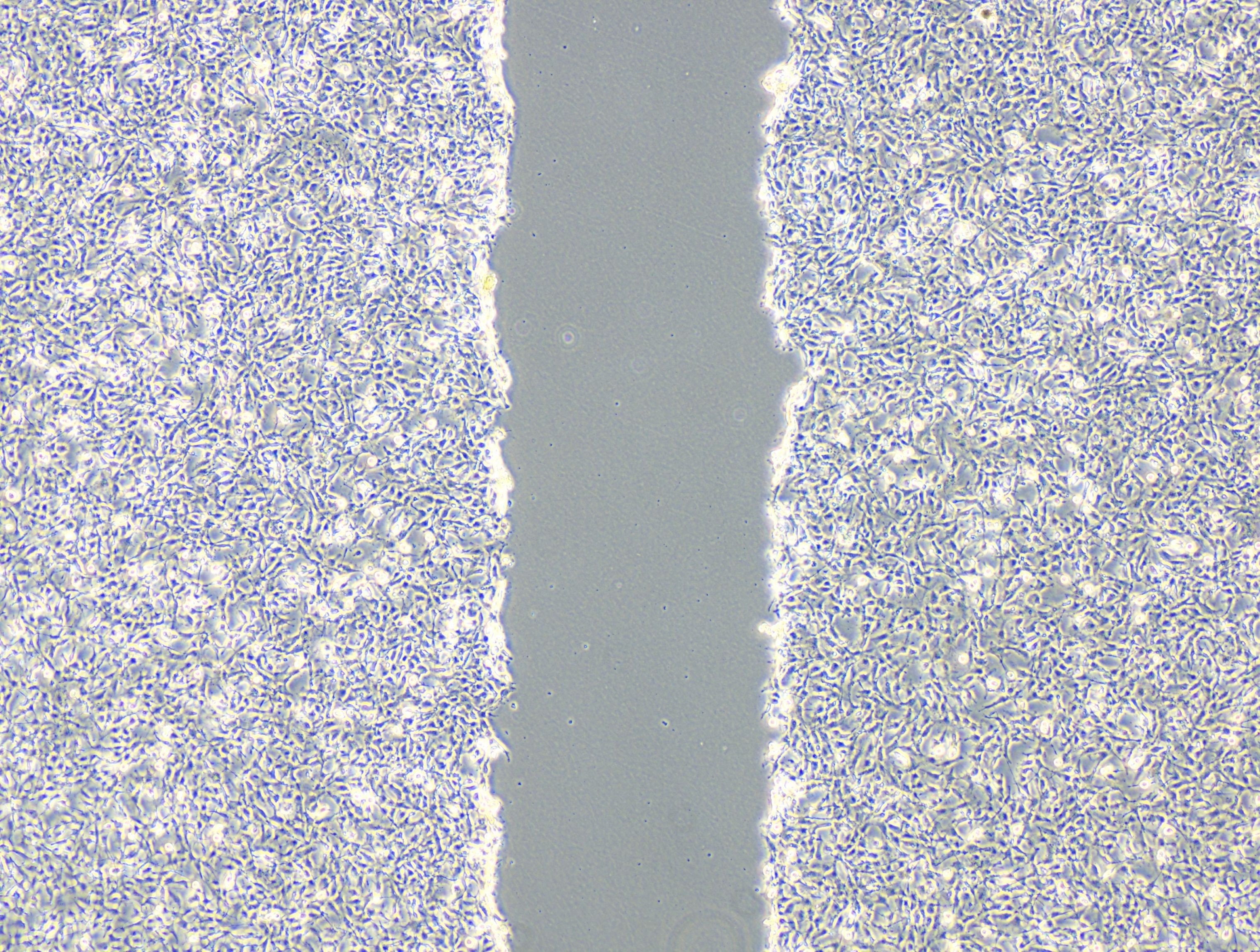

Supplement: Supplementary file 8 [file DataSheet8.zip › wound healing assay-si-COL1A2/3-NC-0h.jpg]

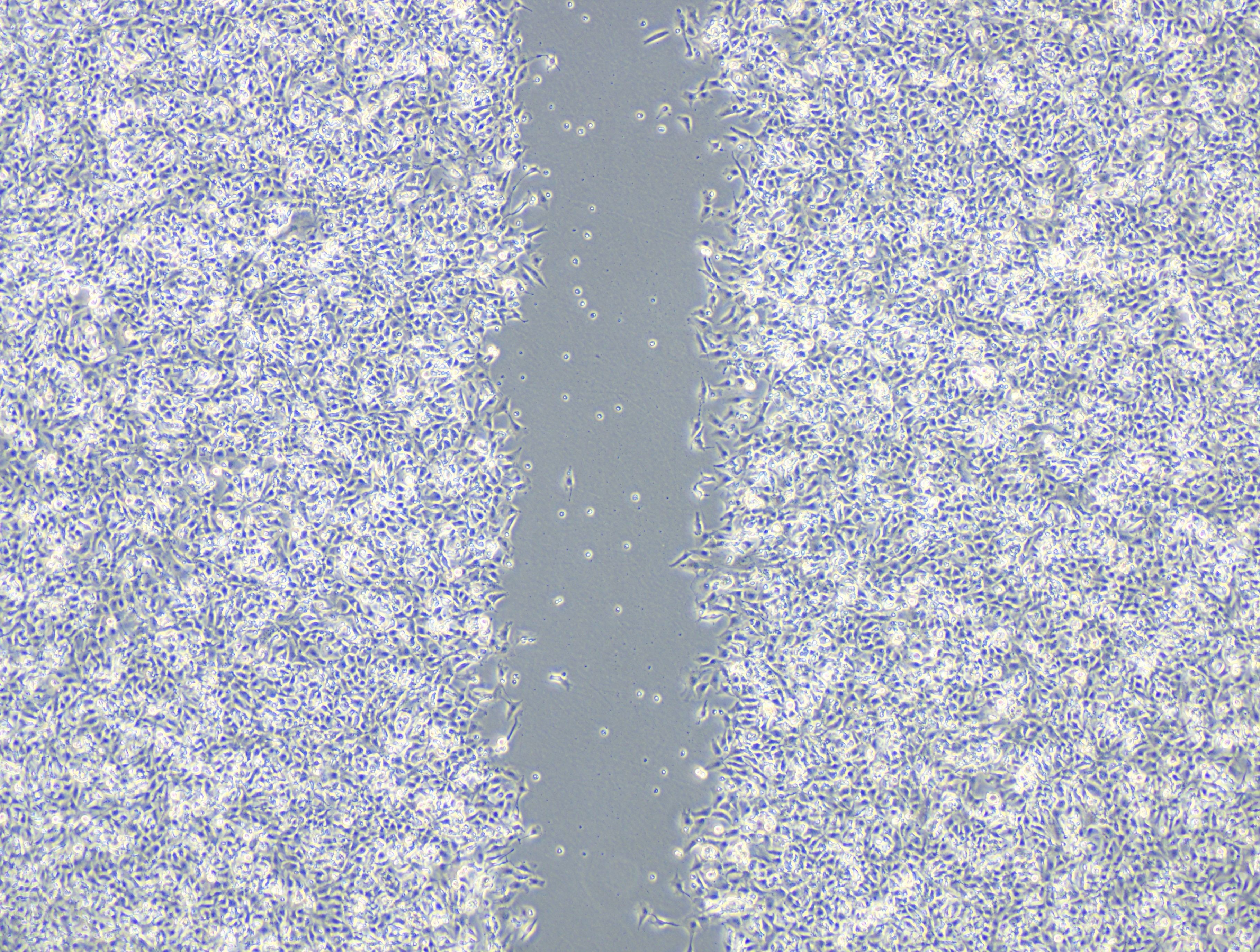

Supplement: Supplementary file 8 [file DataSheet8.zip › wound healing assay-si-COL1A2/3-NC-24h.jpg]

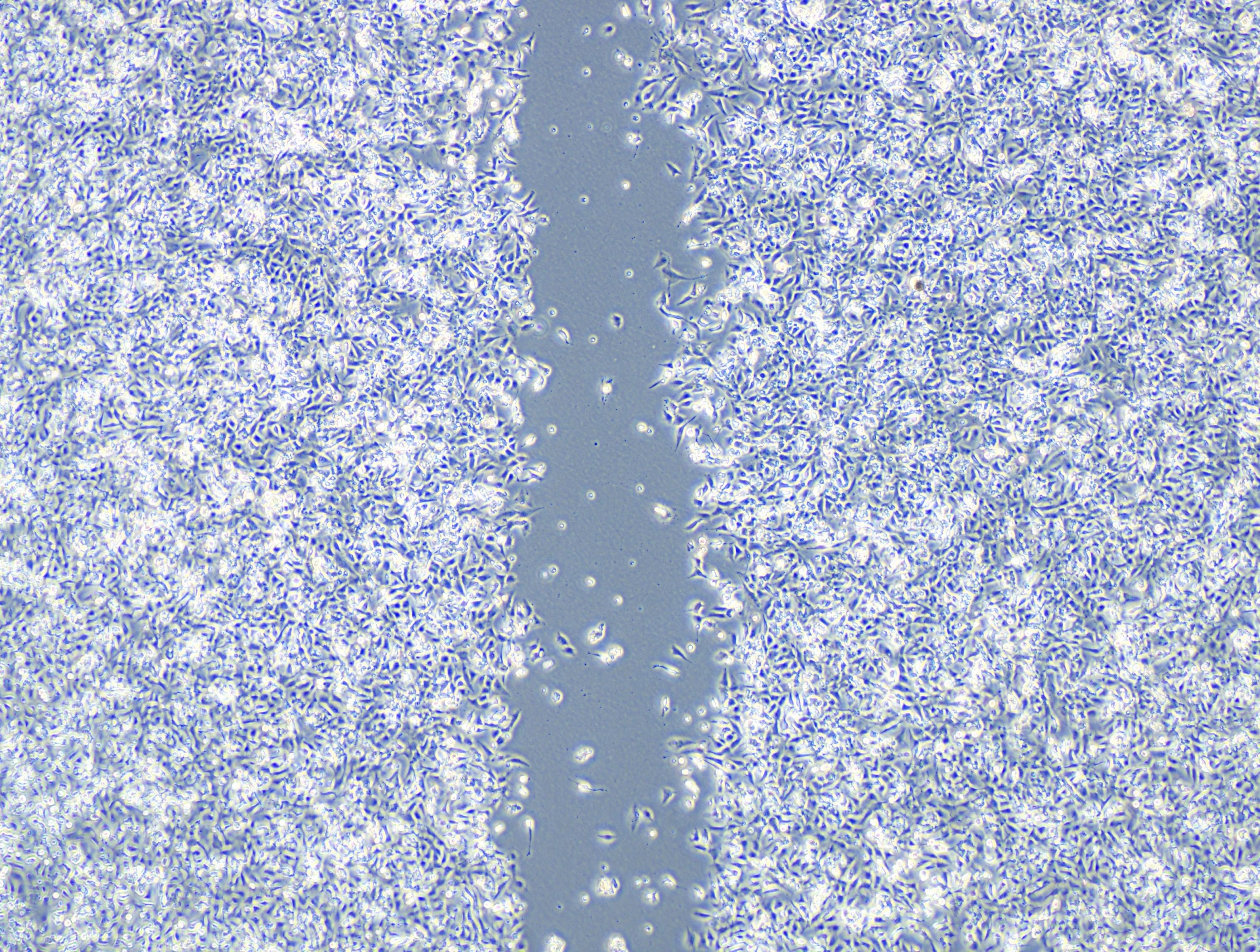

Supplement: Supplementary file 8 [file DataSheet8.zip › wound healing assay-si-COL1A2/3-NC-48h.jpg]

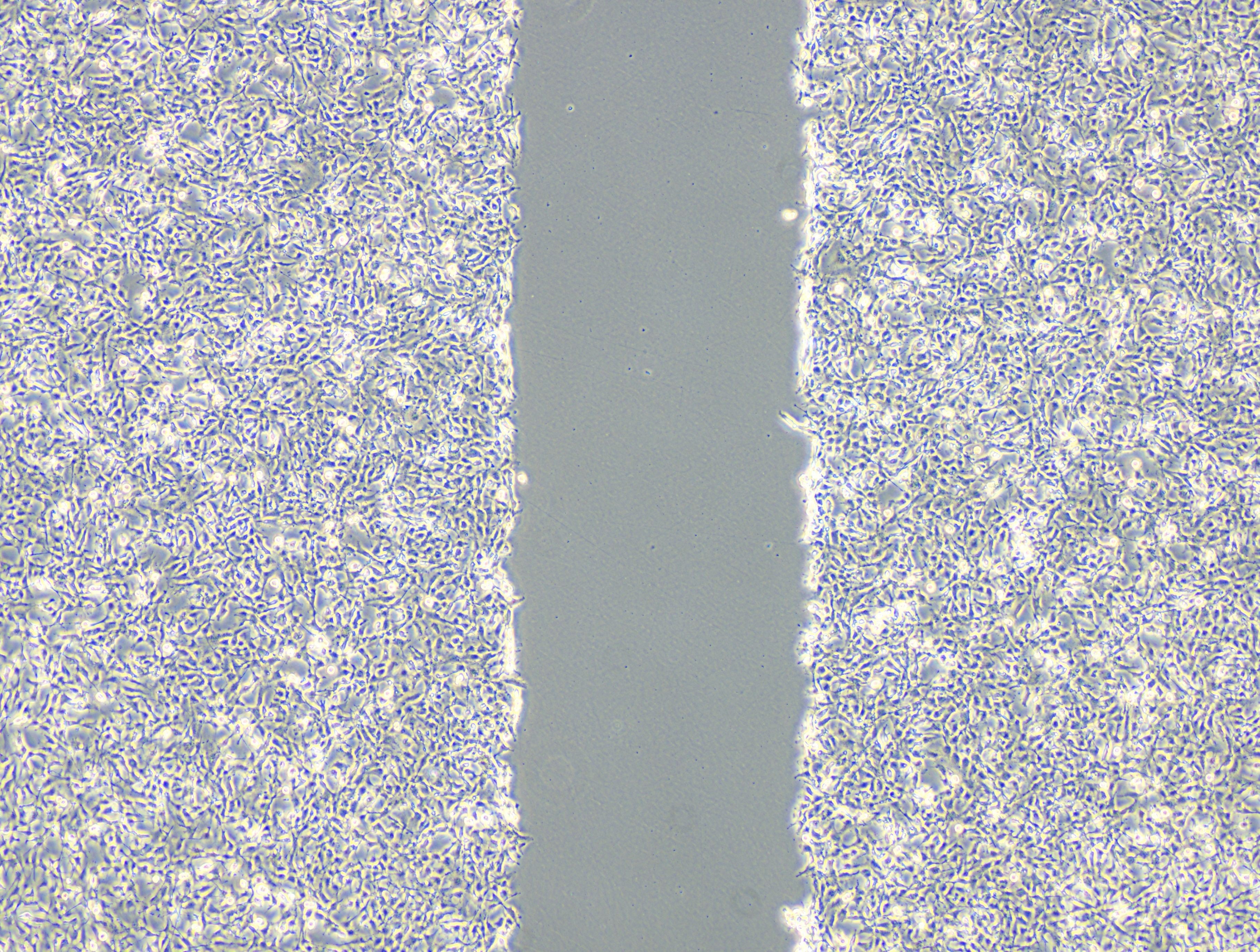

Supplement: Supplementary file 8 [file DataSheet8.zip › wound healing assay-si-COL1A2/3-si-0h.jpg]

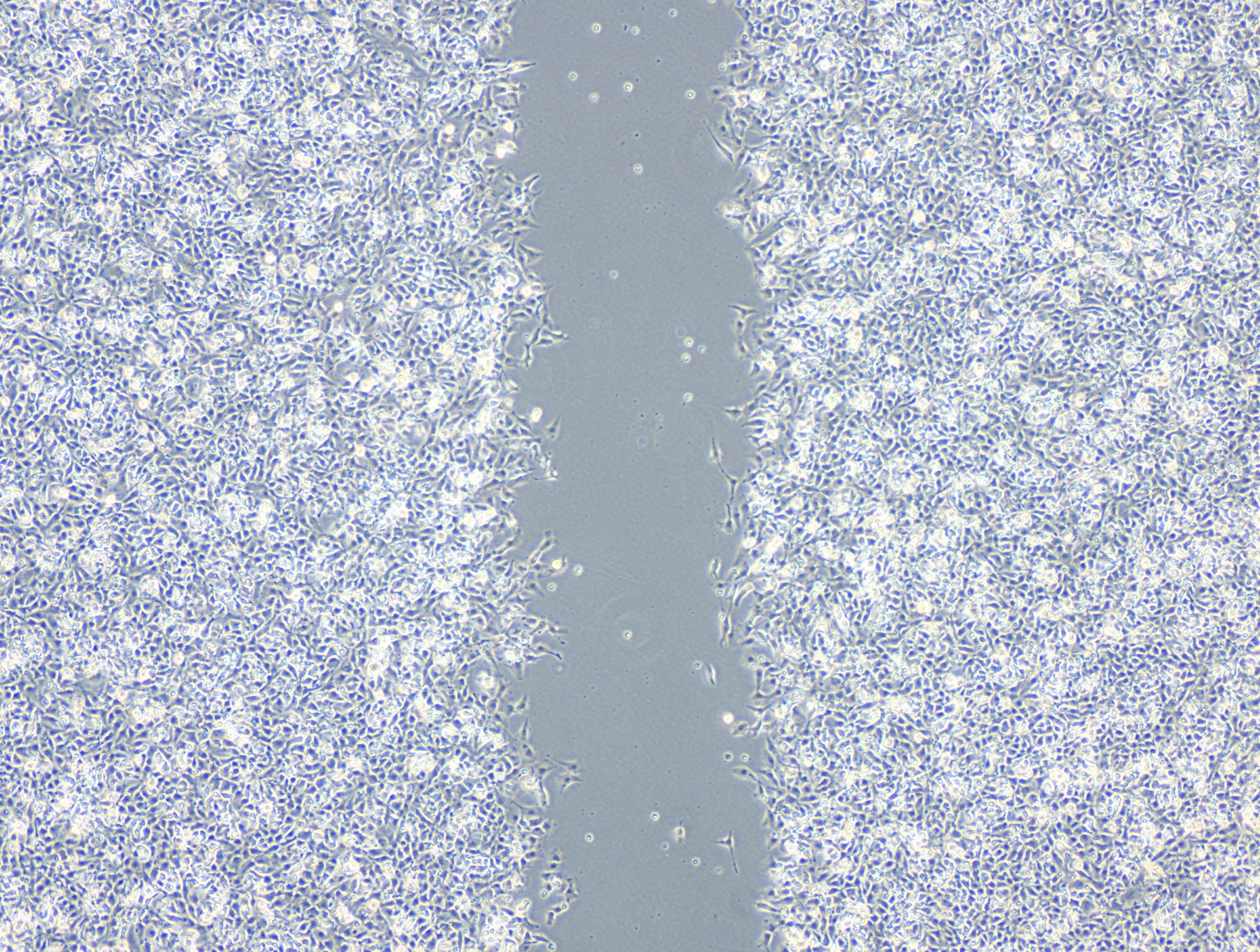

Supplement: Supplementary file 8 [file DataSheet8.zip › wound healing assay-si-COL1A2/3-si-24h.jpg]

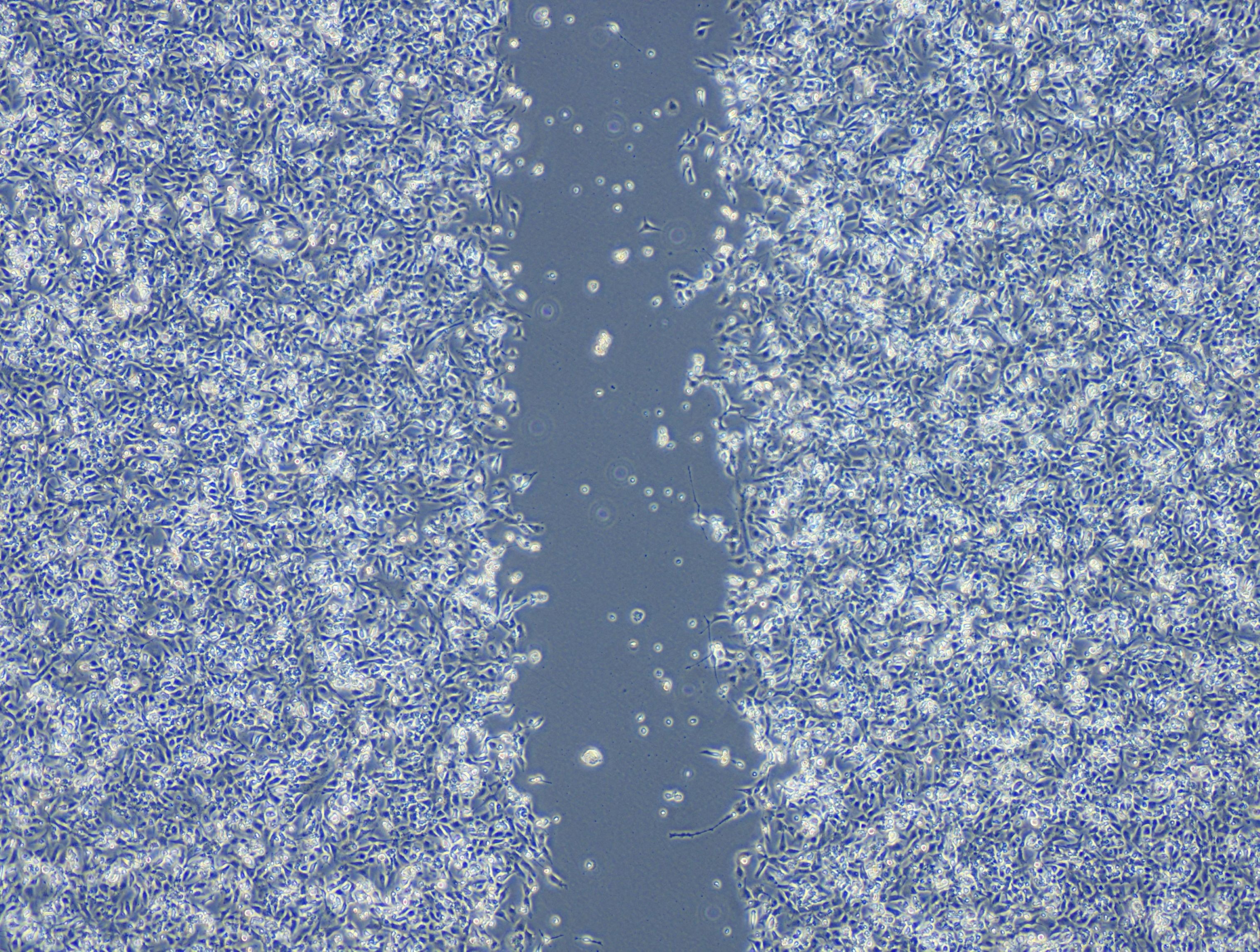

Supplement: Supplementary file 8 [file DataSheet8.zip › wound healing assay-si-COL1A2/3-si-48h.jpg]

# Time-dependent ROC Curves for COL1A2 in Bladder Cancer

N = 908 (Events: 197, Censored: 711)

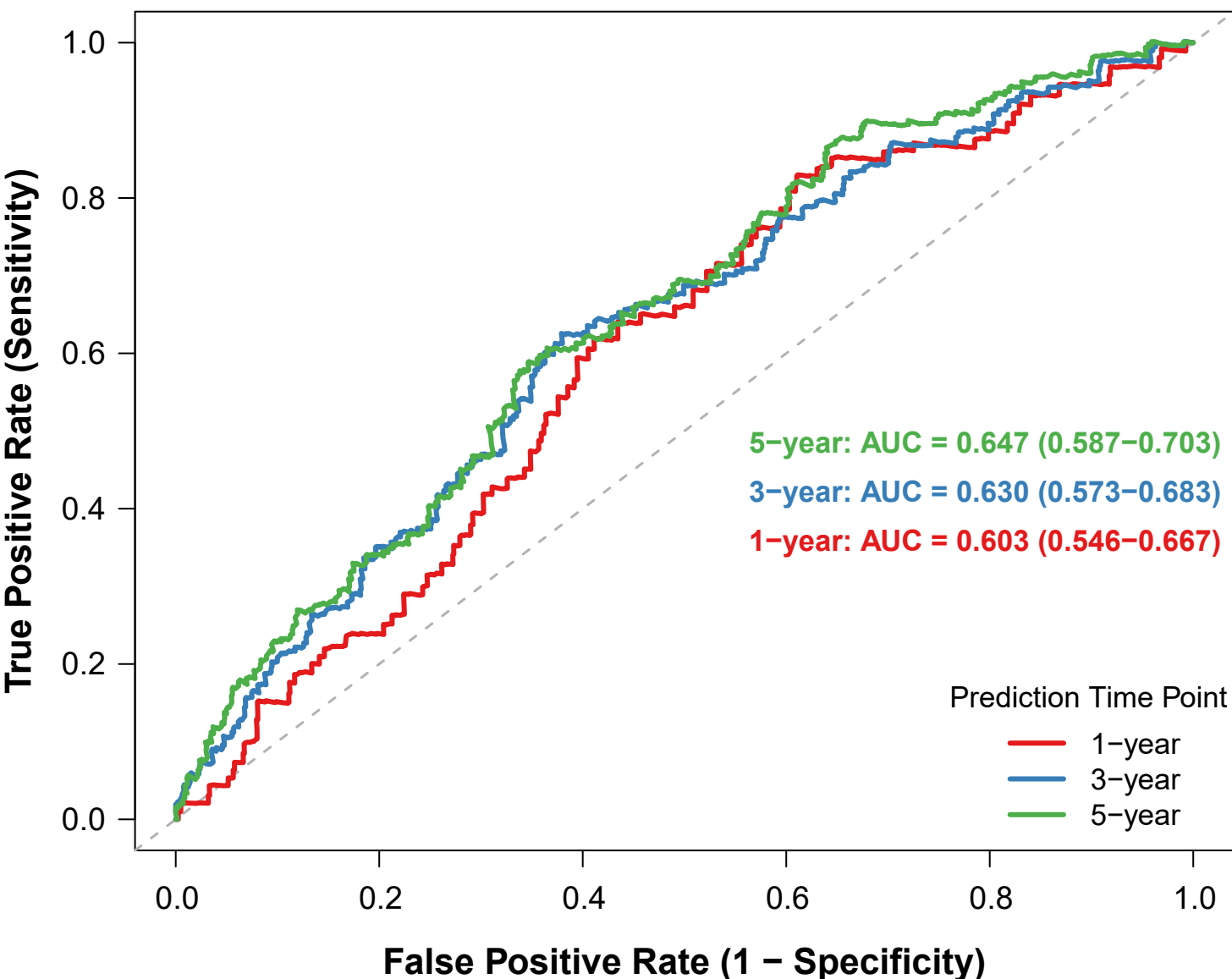

Supplement: Supplementary file 9 [file DataSheet9.pdf]

Cross-validation AUC Distribution

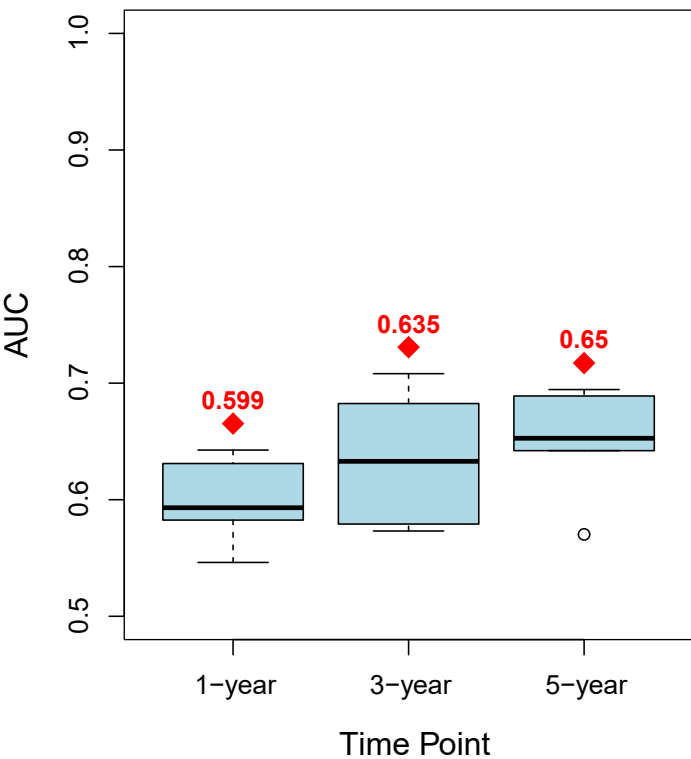

AUC: Full Dataset vs Cross-validation

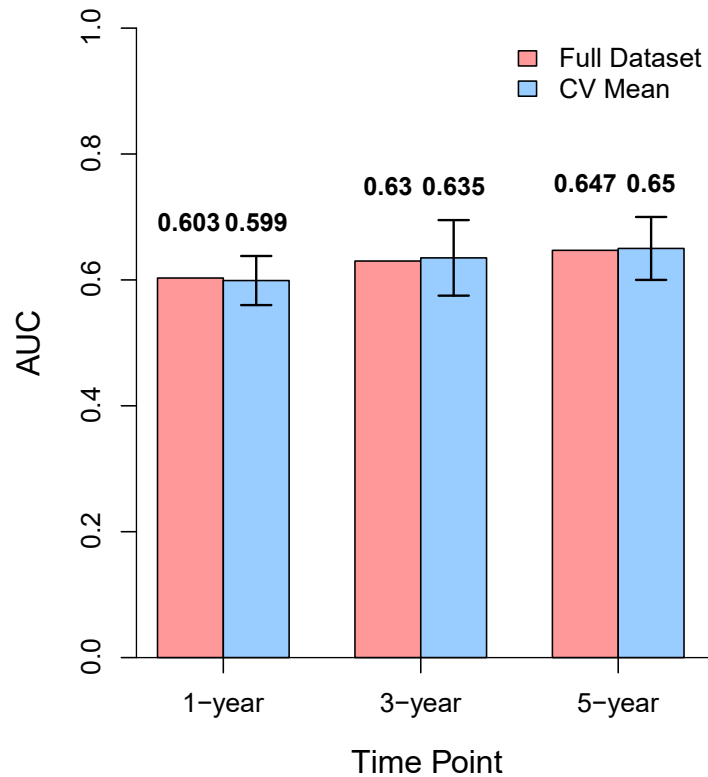

Supplement: Supplementary file 10 [file DataSheet10.pdf]
